# Catalytic Asymmetric Construction of α,α‐Diaryl Aldehydes via Oxo‐Hydroarylation of Terminal Alkynes

**DOI:** 10.1002/advs.202309645

**Published:** 2024-04-22

**Authors:** Xueting Zhou, Qingqin Huang, Jiami Guo, Lei Dai, Yixin Lu

**Affiliations:** ^1^ Joint School of National University of Singapore and Tianjin University International Campus of Tianjin University Binhai New City Fuzhou 350207 China; ^2^ Department of Chemistry National University of Singapore 3 Science Drive 3 Singapore 117543 Singapore

**Keywords:** α,α‐diaryl aldehydes, chiral aldehydes, chiral phosphoric acid, oxo‐hydroarylation of alkynes, tertiary stereocenters

## Abstract

Chiral aldehydes containing a tertiary stereogenic center are versatile building blocks in organic chemistry. In particular, such structural motifs bearing an α,α‐diaryl moiety are very challenging scaffolds and their efficient asymmetric synthesis is not reported. In this work, a phosphoric acid‐catalyzed enantioselective synthesis of α,α‐diaryl aldehydes from simple terminal alkynes is presented. This approach yields a wide range of highly enolizable α,α‐diaryl aldehydes in good yields with excellent enantioselectivities. Facile transformations of the products, as well as an efficient synthesis of bioactive molecules, including an effective anti‐smallpox agent and an FDA‐approved antidepressant drug (+)‐sertraline, are demonstrated.

## Graphical abstract

Chiral α,α‐diaryl aldehydes serve as crucial and versatile building blocks in organic chemistry. However, catalytic asymmetric synthesis of such molecules containing a tertiary stereogenic center is not documented before. Herein, a catalytic enantioselective synthesis of α,α‐diaryl aldehydes from simple terminal alkynes via chiral phosphoric acid catalysis is disclosed.

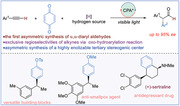

## 1 Introduction

**Figure 1:**
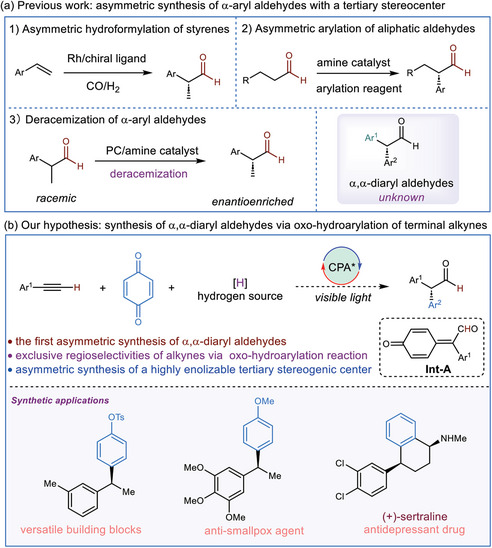
Background and our working hypothesis. a) Previous work: asymmetric synthesis of α‐aryl aldehydes with a tertiary stereocenter b) Our hypothesis: synthesis of α,α‐diaryl aldehydes via oxo‐hydroarylation of terminal alkynes. PC, photocatalyst; CPA, chiral phosphoric acid.

Aldehydes play a pivotal role as versatile building blocks in organic synthesis, and their transformations represent foundational chemical reactions found in textbooks. In this context, chiral α‐aryl aldehydes are especially valuable, as they can be easily converted to a variety of chiral products through facile functional group transformations.^[^ ^1^ ^]^ Consequently, the enantioselective synthesis of α‐aryl aldehydes has attracted much attention from the synthetic community. While most synthetic efforts are centered around the creation of chiral aldehydes bearing an α‐quaternary stereogenic center, the preparation of aldehydes containing an α‐tertiary chiral center remains a formidable challenge.^[^ ^2^ ^]^ This is due to the inherent susceptibility of post‐reaction racemization, resulting from product enolization under basic or acidic conditions, which is attributed to the presence of an acidic α‐hydrogen in the product.^[^ ^3^ ^]^ The literature examples are all on the enantioselective synthesis of α,α‐arylalkyl aldehydes (**Figure** **1**a). One strategy to access such scaffolds is asymmetric hydroformylation of styrenes, which has a somewhat limited substrate scope.^[^ ^4^ ^]^ Utilizing enamine catalysis, MacMillan and co‐workers developed an asymmetric α‐arylation of aliphatic aldehydes to prepare α,α‐arylalkyl aldehydes.^[^ ^5^ ^]^ In a recent study, Luo et al. disclosed a catalytic deracemization of α‐branched aldehydes to construct enantiopure α,α‐arylalkyl aldehydes, through photochemical *E/Z* isomerization of enamine intermediates.^[^ ^6^ ^]^ To the best of our knowledge, there is no report on the enantioselective synthesis of tertiary stereogenic center‐containing α,α‐diaryl aldehydes. The synthesis of such molecules is extremely difficult; the p*K*_a_ value of α,α‐diaryl aldehydes is at 10.4,^[^ ^7^ ^]^ and the presence of such an acidic proton requires a synthetic method that operates at very mild reaction conditions to avoid facile post‐reaction racemization of the products. [Supplementary-material advs7920-supitem-0001]

Alkynes represent a family of important chemical feedstocks that are widely used in agrochemical and pharmaceutical industry sectors.^[^ ^8^ ^]^ We recently became interested in developing synthetic methodologies for the multifunctionalization of alkynes, we, therefore wondered if we could devise an efficient synthetic route to access  α,α‐diaryl aldehydes from simple alkyne starting materials. We hypothesize that the employment of terminal alkynes, benzoquinone, and an appropriate hydrogen source under light‐induced chiral phosphoric acid (CPA) catalysis may lead to the formation of α,α‐diaryl aldehydes. Light induces Paternò–Büchi [2+2] reaction between an alkyne and the benzoquinone to generate an oxetane intermediate, which opens up in the presence of CPA to form the *p*‐quinone methide (*p*‐QM) intermediate (**Int**‐**A**).^[^ ^9^ ^]^ By employing a suitable hydrogen source^[^ ^10^ ^]^ under CPA catalysis, a stereoselective reduction may take place to yield optically enriched α,α‐diaryl aldehydes (Figure 1b). Herein, we document a catalytic asymmetric synthesis of α,α‐diaryl aldehydes from alkynes through light‐driven CPA catalysis.

## 2 Results and Discussion

### 2.1 Reaction Optimization

Our investigation commenced with examining the reaction involving terminal alkyne **1a**, benzoquinone **2a**, and Hantzsch ester **3** under 440 nm Kessil LED irradiation and in the presence of CPA catalysts (**Table** **1**). To evaluate the solvent effects, we performed a set of reactions with the employment of CPA **4a** and Hantzsch ester **3a** (entries 1–5). While polar solvents were less ideal, ether was shown to be the best solvent, albeit the chemical yield was low (entry 5). The evaluation of different CPAs was followed. The more hindered CPAs led to generally better yields, however, the enantioselectivities were decreased (entries 6–9). We then turned to the utilization of different Hantzsch esters, hoping to simultaneously improve the enantioselectivity and the yield (entries 10–15). Gratifyingly, this approach turned out to be effective. Among different Hantzsch esters examined, Hantzsch ester **3 g** was found to be the optimal reductant; the desired diaryl aldehyde **5a** was obtained in 92% yield with 95% ee (entry 15). In this sequential procedure, it is crucial to carry out step II at −80 °C, running this step at 0 °C or room temperature led to less ideal results (entries 16 and 17).

**Table 1:** Optimization of the reaction conditions.

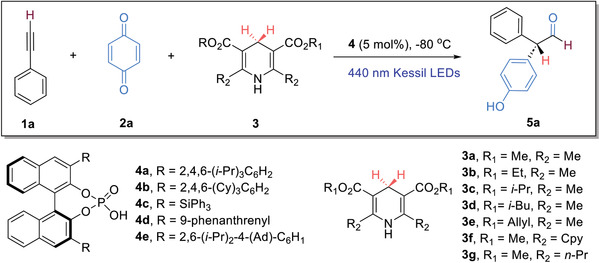					
Entry^a)^	CPA	solvent^b)^	3	yield^c)^	ee^d)^
1	4a	acetone	3a	74%	78%
2	4a	CH_3_CN	3a	67%	72%
3	4a	THF	3a	80%	80%
4	4a	CH_2_Cl_2_	3a	53%	82%
5	4a	ether	3a	41%	88%
6	4b	ether	3a	60%	67%
7	4c	ether	3a	72%	79%
8	4d	ether	3a	81%	67%
9	4e	ether	3a	74%	78%
10	4a	ether	3b	49%	88%
11	4a	ether	3c	40%	83%
12	4a	ether	3d	43%	27%
13	4a	ether	3e	74%	92%
14	4a	ether	3f	25%	20%
15	4a	ether	3g	92%	95%
16^e)^	4a	ether	3g	87%	67%
17**^f^ **^)^	4a	ether	3g	84%	59%

^a)^ Reaction conditions; step I: terminal alkyne **1a** (0.1 mmol) and **2a** (0.05 mmol) in CH_2_Cl_2_ (0.5 mL) were irradiated using 440 nm Kessil LEDs at room temperature for 2 h; step II: at −80 °C, CPA **4** (5 mol%) in the solvent specified (4.5 mL) was introduced, followed by **3** (0.06 mmol), stirring for 12 h;

^b)^ For step II, mixed CH_2_Cl_2_ (2.5 mL)/ether (2.0 mL) were used;

^c)^ Isolated yields;

^d)^ Determined by chiral HPLC analysis of the corresponding alcohol;

^e)^ step II: reaction was performed at 0 °C;

^f)^ step II: reaction was performed at room temperature. ee, enantiomeric excess; Cpy, cyclopropyl; ether, diethyl ether.

### 2.2 Substrate Scope

**Figure 2:**
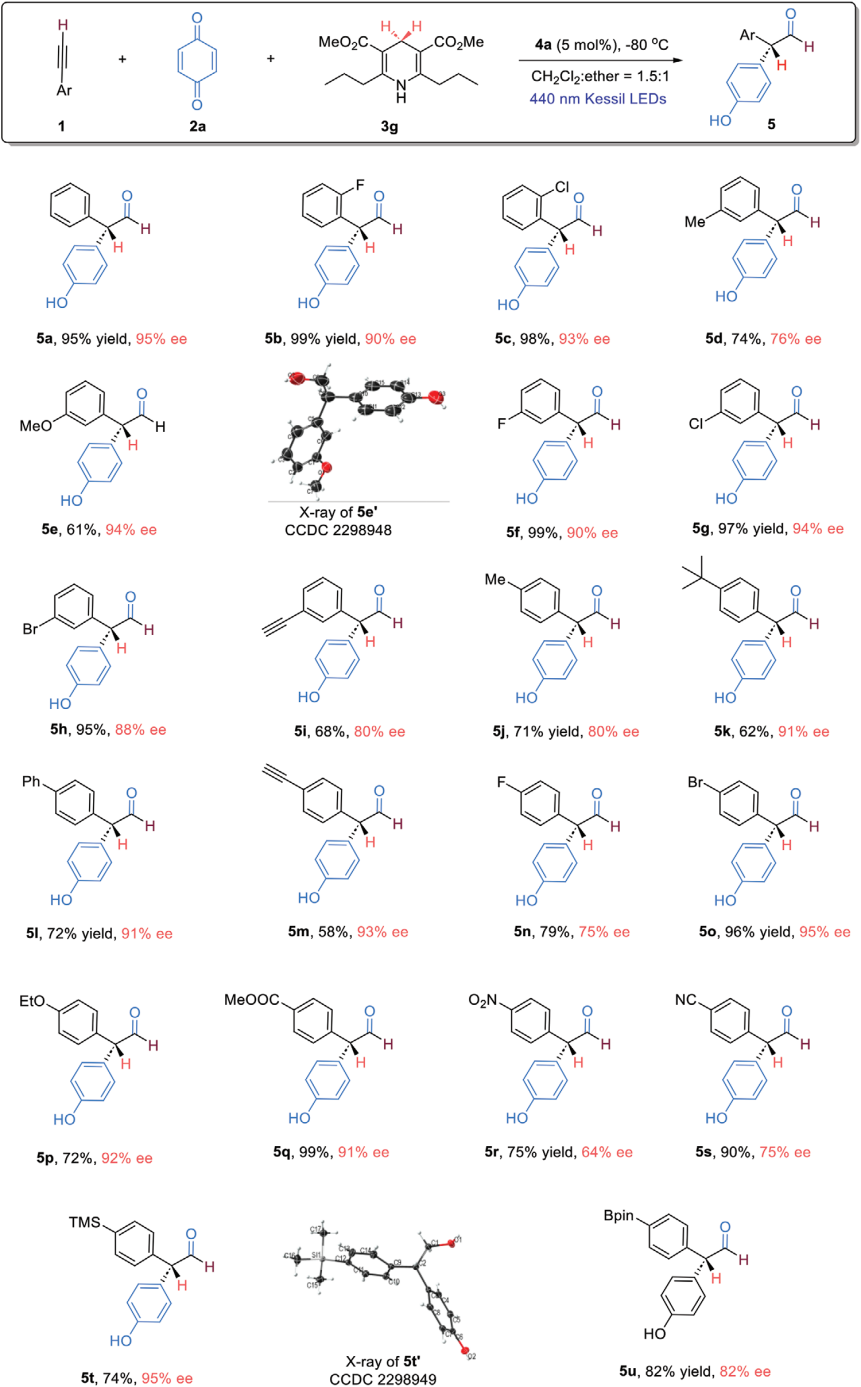
Reaction scope. Reaction conditions: step I: terminal alkyne **1** (0.1 mmol) and **2a** (0.05 mmol) in CH_2_Cl_2_ (0.5 mL) were irradiated using 440 nm Kessil LEDs at room temperature for 2  h; step II: at −80 °C, CPA **4a** (5 mol%) in the mixed CH_2_Cl_2_ (2.5 mL)/ether (2.0 mL) was introduced, followed by **3** **g** (0.06 mmol), stirring for 12 h.

The reaction scope was next investigated, and a diverse range of terminal alkynes bearing a mono‐substituted phenyl group were evaluated (**Figure** **2**). Alkynes containing an *ortho*‐substituted aryl were evaluated first, both fluorinated and chlorinated substrates were found to be suitable (**5b** and **5c**). Different *meta*‐substituted phenyl moieties in alkyne structures, regardless of their electronic nature, were well‐tolerated, and the corresponding aldehyde products were obtained in generally high yields and excellent ee values (**5d–5 h**). It is interesting to note that when 1,3‐diethynylbenzene was utilized, one triple bond reacted to form the aldehyde product, whereas the other triple bond remained intact (**5i**). We next examined a broad range of *para*‐substituted phenyl alkynes. The alkyl groups, e.g. methyl‐ (**5j**), *tert*‐butyl‐ (**5k**), and phenyl substituent (**5l**), were found to be compatible, and satisfactory yields and good enantioselectivities were constantly attainable. When 1,4‐diethynylbenzene was employed, only one triple bond reacted, in a fashion similar to that of 1,3‐diethynylbenzene (**5m** versus **5i**). Moreover, alkynes with a fluorinated or chlorinated phenyl ring were well‐tolerated (**5n** & **5o**), so were the alkynes bearing different functional groups, from ether (**5p**), ester (**5q**), nitro (**5r**), to cyano (**5s**). Notably, the aldehyde products containing a strong electron‐withdrawing group (**5r** & **5s**) were formed with lower enantiomeric excesses, likely due to the presence of a more acidic α‐hydrogen in these structures. It is noteworthy that alkynes containing a trimethylsilyl group (**5t**) and a Bpin moiety (**5u**) turned out to be good substrates, and the corresponding diaryl aldehydes were obtained in high yields with good enantioselectivities. The synthetic utility of the latter is especially valuable, as the boronate group can be readily subjected to the cross‐coupling reactions for further structural elaboration. The absolute configurations of diaryl aldehyde products were assigned on the basis of the X‐ray crystallographic analysis of the corresponding alcohols **5e’** ^[^ ^11^ ^]^ and **5t’** (For full details of X‐ray crystallographic analysis, see the Supporting Information).^[^ ^12^ ^]^

**Figure 3:**
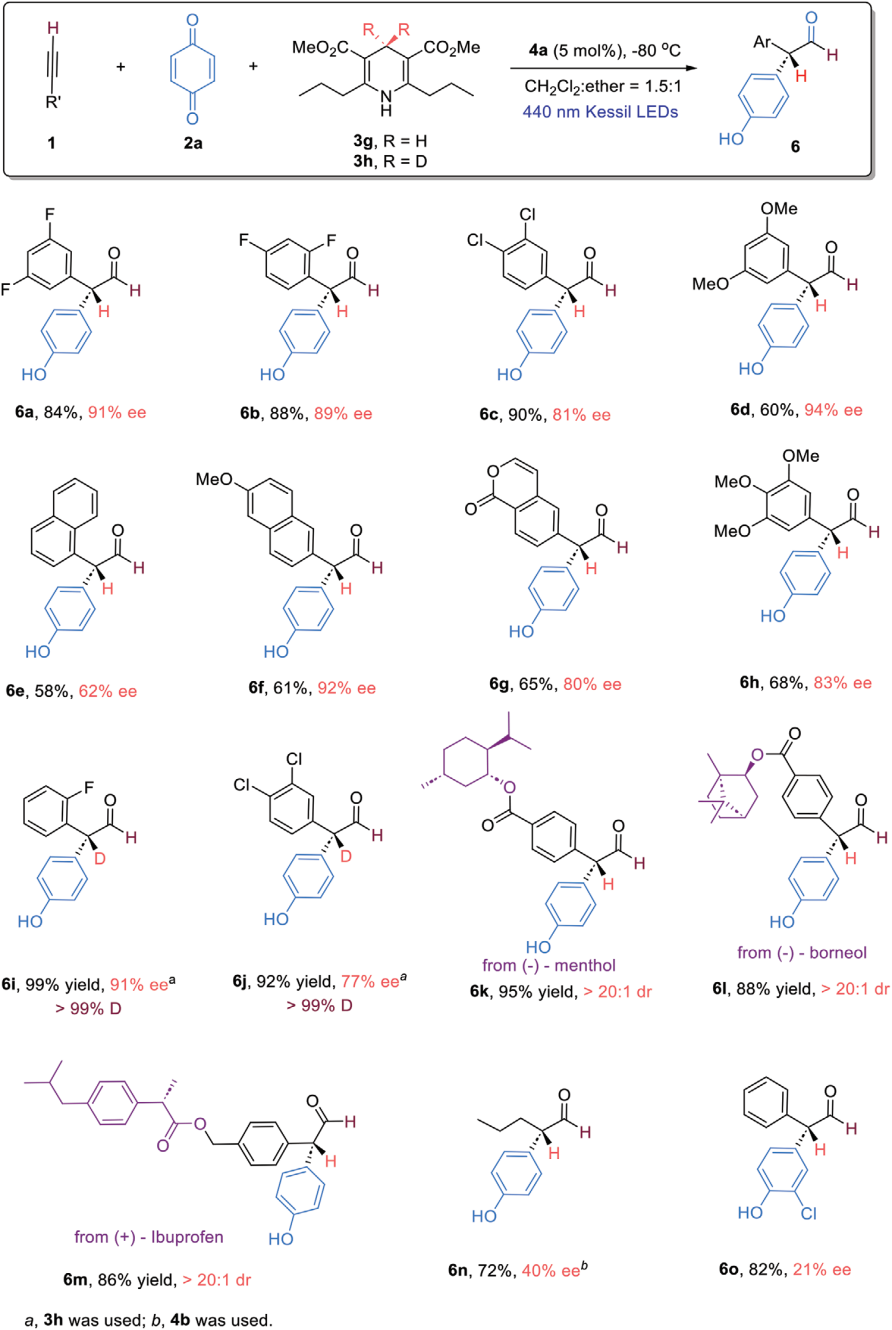
Further reaction scope. Reaction conditions: step I: terminal alkyne **1** (0.1 mmol) and **2a** (0.05 mmol) in CH_2_Cl_2_ (0.5 mL) were irradiated using 440 nm Kessil LEDs at room temperature for 2 h; step II: at −80 °C, CPA **4a** (5 mol%) in the mixed CH_2_Cl_2_ (2.5 mL)/ether (2.0 mL) was introduced, followed by **3** (0.06 mmol), stirring for 12 h.

To further evaluate the reaction scope, terminal alkynes bearing more complex aryl moieties were next examined (**Figure** **3**). Alkynes with a difluoro‐ or dichloro‐ substituted phenyl group were found to be good substrates, regardless of the substitution pattern (**6a**−**6c**). With the employment of dimethoxy‐substituted phenyl in the alkyne structure, the corresponding aldehyde with high ee value was obtained in somewhat lower yield (**6d**). Alkynes bearing naphthyl moieties could be utilized, although the enantioselectivities dropped when α‐naphthyl‐containing alkyne was used (**6e**), 2‐ethynyl‐7‐methoxynaphthalene, on the other hand, turned out to be an excellent substrate, and the desired product was obtained with 92% ee (**6f**). When an alkyne with a fused lactone moiety was used, good enantioselectivity was attainable (**6** **g**). Furthermore, the alkyne substrate bearing a trisubstituted phenyl group was also well‐tolerated (**6** **h**). Deuterium‐labelled compounds are useful tools in biology and medicinal chemistry, we next evaluated the suitability of our method for deuterium labeling. With the utilization of deuterated Hantzsch ester **3** **h**, the corresponding deuterated aldehydes were obtained in high yields, good enantioselectivities, and quantitative deuterium incorporation at the α‐position (**6i** & **6j**). The compatibility of our method for the late‐stage functionalization of natural products and drug molecules was next investigated, and the incorporation of (‐)‐menthol, (‐)‐borneol, and (+)‐ibuprofen took place smoothly, and the corresponding aldehydes were obtained in good yields with excellent diastereoselectivities (**6k–6m**). Additionally, we examined the suitability of alkyl‐substituted alkyne substrate; the employment of 1‐pentyne led to the formation of the corresponding aldehyde **6n** in good yield, but with poor enantioselectivity. Furthermore, substituted benzoquinone was also investigated, which was found to be less ideal, furnishing the desired product in good yield with low enantioselectivity (**6o**).

### 2.3 Mechanistic Studies

**Figure 4:**
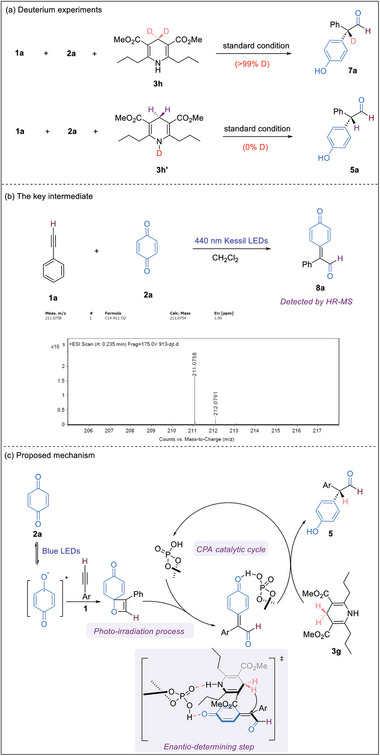
Mechanistic studies. a) Deuterium experiments; b) The key intermediate; c) Proposed mechanism.

To probe the reaction mechanism, a number of experiments were conducted. We first performed deuterium experiments to identify the source of the α‐H in the products. The utilization of 4‐*d_2_* Hantzsch ester **3** **h** led to quantitative deuterium incorporation, whereas the employment of *N*‐deuterated Hantzsch ester **3h’** did not yield any deuterated product (**Figure** **4**a). Under the irradiation of 440 nm Kessil LEDs, alkyne **1a** reacted with benzoquinone **2a** to form *p*‐QM **8a**, confirmed through high‐resolution mass spectrometry (HRMS) analysis. (Figure 4b). Based on these results, a plausible reaction mechanism is proposed. In the presence of 440 nm blue LEDs, the alkyne and benzoquinone undergo a [2+2] cycloaddition, resulting in the formation of an oxetene intermediate, which rapidly generates a *p*‐QM intermediate via the alkyne‐carbonyl metathesis reaction. Subsequent CPA‐promoted hydrogen atom transfer from Hantzsch ester to the *p*‐QM intermediate produces the α,α‐diaryl aldehyde product. Apparently, Hantzsch ester is important in this enantio‐determining step, which plays a crucial role in differentiating divergent reaction pathways through interactions with *p*‐QM and CPA, thus forming the observed major stereoisomer (Figure 4c).

### 2.4 Synthetic Applications

**Figure 5:**
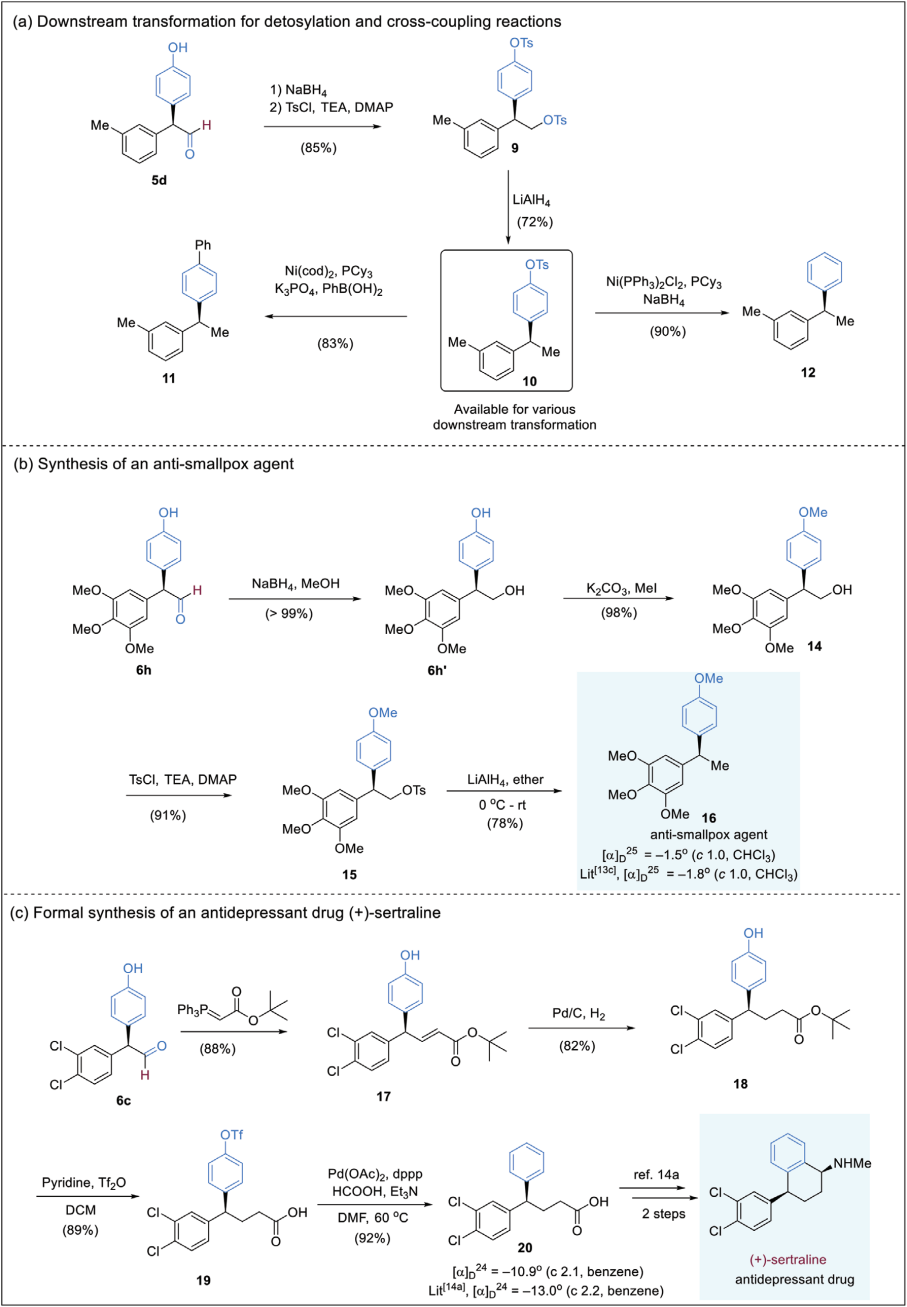
Synthetic applications. a) Downstream transformation for detosylation and cross‐coupling reactions; b) Synthesis of an anti‐smallpox agent; c) Formal synthesis of an antidepressant drug (+)‐sertraline.

The α,α‐diaryl aldehydes are useful chiral building blocks and synthetic intermediates, which can be readily subjected to further synthetic transformations to yield molecules of biological significance. The phenol moiety in the products can be easily synthetically manipulated, allowing access to other aryl moieties. Reduction of aldehyde **5d**, followed by tosylation, yielded bis‐tosylated product **9**. LAH reduction furnished compound **10**, which could be converted to biaryl **12** in a very good yield through a reductive detosylation process. Alternatively, a Suzuki–Miyama coupling reaction of **10**, furnished product **11** which contains multiple aryl moieties (**Figure** **5**a). In another synthetic illustration, aldehyde **6** **h** was reduced to yield alcohol **6h’**, subsequent selective methylation led to the formation of **14**. A tosyl group was then installed to afford intermediate **15**, which was treated with LiAlH_4_ to produce an anti‐smallpox agent **16**.^[^ ^13^ ^]^ The NMR spectra and specific rotation of our synthetic sample are fully consistent with literature reports^[^ ^13b,c^ ^]^ (Figure 5b). Moreover, we also developed a concise route toward the formal synthesis of an antidepressant drug, (+)‐sertraline.^[^ ^14^ ^]^ Aldehyde **6c** was converted to acrylate **17** via a Wittig reaction, subsequent hydrogenation and triflation furnished **19**. Palladium‐catalyzed removal of the triflate group proceeded smoothly to yield reported advanced intermediate **20**.^[^ ^14a^ ^]^ The NMR spectra and specific rotation of compound **20** were compared with literature reports^[^ ^14a^ ^]^ and found to be consistent (Figure 5c).

## 3 Conclusion

In summary, we have developed the first enantioselective synthesis of α,α‐diaryl aldehydes from terminal alkynes. It is noteworthy that the aldehyde products contain a highly enolizable tertiary chiral center. The method is straightforward and operationally simple, making use of readily accessible benzoquinone, Hantzsch esters, and CPAs as reaction partners. The α,α‐diaryl aldehydes prepared through our method are synthetically useful, which not only led to the synthesis of optically enriched α‐deuterated α,α‐diaryl aldehydes, but also served as a versatile intermediate leading to efficient synthesis of a number of bioactive and drug molecules. Current research in our laboratory continues on the development of enantioselective multifunctionalization of simple alkynes.

## Conflict of Interest

The authors declare no conflict of interest.

## Supporting information

Supporting Information

## Data Availability

The data that support the findings of this study are available from the corresponding author upon reasonable request.
