# Supplementary material for: Catalytic Asymmetric Construction of α,α‐Diaryl Aldehydes via Oxo‐Hydroarylation of Terminal Alkynes
Source: Adv Sci (Weinh). 2024 Apr 22;11(24):2309645. doi: 10.1002/advs.202309645 (PMC11199996; doi:10.1002/advs.202309645)
Supplement: Supplementary file 1 — Supporting Information [file ADVS-11-2309645-s001.pdf]

ADVANCED SCIENCE
Open Access

Supporting Information

for *Adv. Sci.*, DOI 10.1002/advs.202309645

Catalytic Asymmetric Construction of α,α-Diaryl Aldehydes via Oxo-Hydroarylation of Terminal Alkynes

Xueting Zhou, Qingqin Huang, Jiami Guo, Lei Dai and Yixin Lu**

Supporting Information

Catalytic asymmetric construction of α,α-diaryl aldehydes via oxo-hydroarylation of terminal alkynes

Xueting Zhou[1,2,+], Qingqin Huang[1,2,+], Jiami Guo[1,2], Lei Dai[2], and Yixin Lu[1,2]**

[1]X. Zhou, Q. Huang, J. Guo, Prof. Dr. Y. Lu

Joint School of National University of Singapore and Tianjin University

International Campus of Tianjin University

Binhai New City, Fuzhou 350207 (China)

[2]X. Zhou, Q. Huang, J. Guo, Dr. L. Dai and Prof. Dr. Y. Lu

Department of Chemistry

National University of Singapore

3 Science Drive 3, 117543 (Singapore)

[+]X. Zhou and Q. Huang contributed equally.

E-mail: L. Dai: chmdail@nus.edu.sg; Y. Lu: chmlyx@nus.edu.sg.

Content

1. Materials and Methods..3

2. General Procedures 4

3. Analytical Data of the Products 5

4. Mechanistic Sudies 23

5. Synthetic Applications 25

6. Single Crystal Structure X-ray Analysis of **5e’** and **5t’** 34

7. References..56

8. Copies of HPLC spectra 57

9. Copies of NMR spectra..123

1. Materials and Methods

All starting materials were obtained from commercial suppliers (Sigma Aldrich and TCI) and directly used without further purification unless otherwise stated. All reactions were carried out under argon atmosphere with magnetic stirring. Terminal alkynes[1] and Hantzsch esters[2] were synthesized according to literatures. Benzoquinone was purchased from Sigma-Aldrich and used without further purification. All chiral phosphoric acids were purchased from Daicel Chiral Technologies.

Analytical thin layer chromatography was carried out with silica gel pre-coated glass plates (TLC-Silica gel GF254, coating thickness: 0.25 mm) purchased from Merck. Visualization was accomplished with short wave UV light (254nm, 365nm) and/or 10% phosphomolybdic acid in ethanol or KMnO4 staining solutions followed by heating. Column chromatography was performed on silica gel 200~300 mesh. 440 nm Kessil LEDs was purchased from kessil.com. ^{1}H NMR and ^{13}C NMR spectra were recorded on a Bruker AV-III400 (400 MHZ) or AMX500 (500 MHz) spectrometer. Chemical shifts were calibrated using residual solvent as an internal reference ($CDCl_3$: 7.18 ppm ^{1}H NMR, 77.00 ppm ^{13}C NMR). ^{1}H NMR Spectroscopy splitting patterns were designated as singlet (s), doublet (d), triplet (t), quartet (q). Splitting patterns that could not be interpreted or easily visualized were designated as multiplet (m) or broad (br). All high-resolution mass spectra (HRMS) were obtained on a Finnigan/MAT 95XL-T spectrometer, the calculated values are based on the most abundant isotope. Absorption spectra were recorded in 1 cm path quartz cuvettes using an Edinburgh FS-5 spectrofluorometer. Chiral HPLC analyses were performed on an Agilent 1100 Series using a Daicel Chiralpak column (IE, IA) with hexanes/iPrOH as the eluent.

2. General Procedures

Figure S1. General procedure for the enantioselective synthesis of α,α-diaryl aldehydes.

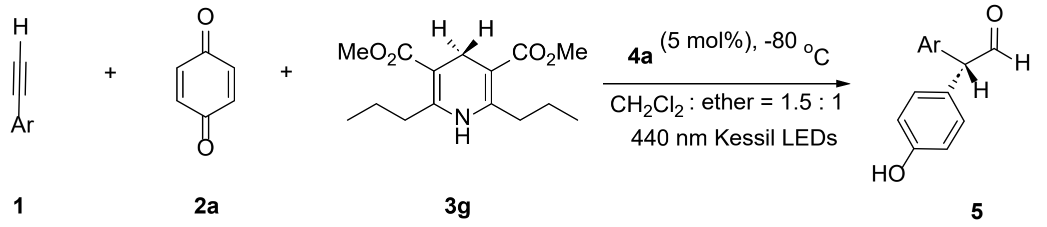

To a dried 1.5 mL screw-cap vial equipped with a magnetic stir bar were added alkyne **1** (0.1 mmol) and benzoquinone **2a** (5.4 mg, 0.05 mmol) in CH_2Cl_2 (0.5 mL). The mixture was then irradiated by 440 nm Kessil LEDs at room temperature for 2 h. At -80 °C, CPA **4a** (1.9 mg, 5 mol%) in CH_2Cl_2 (2.5 mL)/ether (2.0 mL) was added to the mixture in step I, then **3g** (16.9 mg, 0.06 mmol) was added and the resulting mixture was stirred for 12 h. For easy analysis, addition of $NaBH_4$ (1.5 equiv) in MeOH to the reaction mixture containing aldehyde **5'** gave the according alcohol product, which was obtained by silica gel column chromatography.

Figure S2. General procedure for alkynes with more complex structures and substitution patterns.

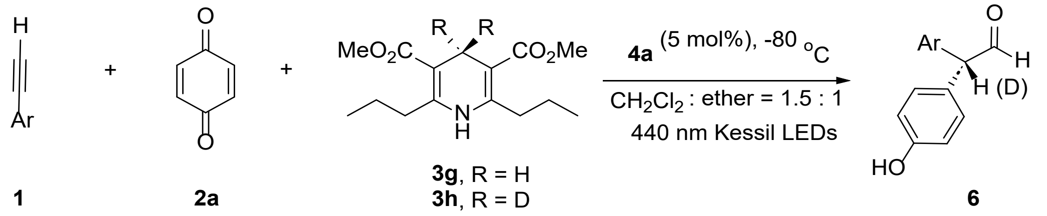

To a dried 1.5 mL screw-cap vial equipped with a magnetic stir bar were added alkyne **1** (0.1 mmol) and benzoquinone **2a** (5.4 mg, 0.05 mmol) in CH_2Cl_2 (0.5 mL). The mixture was then irradiated by 440 nm Kessil LEDs at room temperature for 2 h. At -80 °C, CPA **4a** (1.9 mg, 5 mol%) in CH_2Cl_2 (2.5 mL)/ether (2.0 mL) was added to the mixture in step I, then **3g** or **3h** (16.9 mg, 0.06 mmol) was added and the resulting mixture was stirred for 12 h. For easy analysis, addition of $NaBH_4$ (1.5 equiv) in MeOH to the reaction mixture containing aldehyde **6'** gave the according alcohol product, which was obtained by silica gel column chromatography.

3. Analytical Data and HPLC Chromatograms of the Products

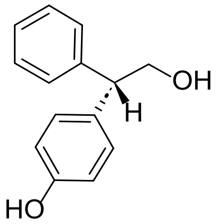

5a'

(*S*)-4-(2-Hydroxy-1-phenylethyl)phenol (5a')

95% yield, $[\alpha]_D^{25}$ = +3.0 (c 0.5, acetone), a light-yellow oil, ^{1}H NMR (400 MHz, CD_3OD) δ 7.34 – 7.13 (m, 5H), 7.11 – 7.01 (m, 2H), 6.77 – 6.64 (m, 2H), 4.04 (d, *J* = 3.4 Hz, 3H). ^{13}C NMR (101 MHz, CD_3OD) δ 156.9, 144.3, 134.6, 130.3, 129.3, 127.2, 116.2, 66.7, 54.3. HRMS (ESI) m/z calcd for $C_{14}H_{13}O_2$ $[M+H]^+$ = 213.0921, found = 213.0921; the ee value was 95%, t_R (major) = 5.8 min, t_R (minor) = 6.8 min (Chiralpak IE, λ = 220 nm, 20% *i*-PrOH/Hexane, flow rate = 1 mL/min).

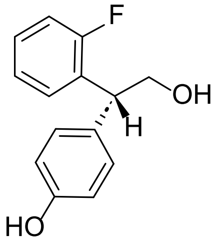

5b'

(*R*)-4-(1-(2-Fluorophenyl)-2-hydroxyethyl)phenol (5b')

99% yield, $[\alpha]_D^{25}$ = -5.2 (c 0.5, acetone), a light-yellow oil, ^{1}H NMR (400 MHz, Acetone-d_6) δ 7.40 (s, 1H), 6.46 (td, *J* = 7.6, 1.8 Hz, 1H), 6.26 (tdd, *J* = 7.3, 5.2, 1.8 Hz, 1H), 6.18 (dd, *J* = 7.6, 1.3 Hz, 1H), 6.16 – 6.11 (m, 2H), 6.07 (ddd, *J* = 10.7, 8.1, 1.3 Hz, 1H), 5.88 – 5.66 (m, 2H), 3.43 (t, *J* = 7.4 Hz, 1H), 3.20 – 3.04 (m, 2H), 3.00 (t, *J* = 5.6 Hz, 1H). ^{13}C NMR (101 MHz, Acetone-d_6) δ 163.1, 160.7, 156.9, 156.8, 133.2, 131.2, 131.0, 130.2, 130.2, 130.1, 128.7, 128.6, 124.9, 124.9, 116.0, 116.0, 115.9, 115.8, 65.4, 65.2, 46.7, 46.7. HRMS (ESI) m/z calcd for $C_{14}H_{12}FO_2$ $[M-H]^-$ = 231.0827, found = 231.0823; the ee value was 90%, t_R (major) = 5.5 min, t_R (minor) = 5.9 min (Chiralpak IE, λ = 220 nm, 20% *i*-PrOH/Hexane, flow rate = 1 mL/min).

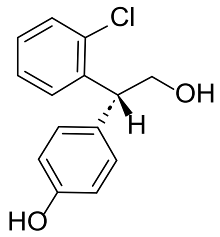

5c'

(*R*)-4-(1-(2-Chlorophenyl)-2-hydroxyethyl)phenol (5c')

98% yield, $[\alpha]_D^{25}$ = -2.4 (c 0.5, acetone), a light-yellow oil, ^{1}H NMR (400 MHz, Acetone-d_6) δ 8.38 (s, 1H), 7.49 (d, *J* = 7.8 Hz, 1H), 7.37 (d, *J* = 7.9 Hz, 1H), 7.30 (t, *J* = 7.5 Hz, 1H), 7.20 (t, *J* = 7.6 Hz, 1H), 7.09 (d, *J* = 8.6 Hz, 2H), 6.74 (d, *J* = 8.2 Hz, 2H), 4.57 (t, *J* = 7.2 Hz, 1H), 4.18 – 3.85 (m, 3H). ^{13}C NMR (101 MHz, Acetone-d_6) δ 156.9, 141.3, 135.2, 132.9, 130.4, 130.3, 130.1, 128.4, 127.7, 115.9, 115.9, 65.5, 50.0. HRMS (ESI) m/z calcd for $C_{14}H_{12}ClO_2$ $[M-H]^-$ = 247.0531, found = 247.0526; the ee value was 93%, t_R (major) = 12.4 min, t_R (minor) = 14.5 min (Chiralpak IE, λ = 220 nm, 10% *i*-PrOH/Hexane, flow rate = 0.8 mL/min).

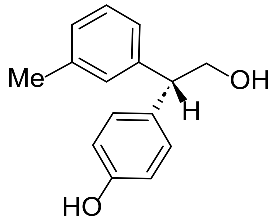

5d'

(*R*)-4-(2-Hydroxy-1-(m-tolyl)ethyl)phenol (5d')

74% yield, $[\alpha]_D^{25}$ = +1.8 (c 0.5, acetone), a light-yellow oil, ^{1}H NMR (400 MHz, Acetone-d_6) δ 8.33 (s, 1H), 7.21 – 7.03 (m, 5H), 6.97 (d, *J* = 7.4 Hz, 1H), 6.74 (d, *J* = 8.5 Hz, 2H), 4.03 (d, *J* = 3.9 Hz, 3H), 3.80 – 3.63 (m, 1H), 2.27 (s, 3H). ^{13}C NMR (101 MHz, Acetone-d_6) δ 156.70 144.34 138.3, 134.6, 130.1, 129.9, 128.9, 127.5, 126.2, 115.9, 115.8, 66.4, 54.1, 21.5. HRMS (ESI) m/z calcd for $C_{15}H_{15}O_2$ $[M-H]^-$ = 227.1078, found = 227.1076; the ee value was 76%, t_R (major) = 15.5 min, t_R (minor) = 18.6 min (Chiralpak IE, λ = 220 nm, 10% *i*-PrOH/Hexane, flow rate = 0.8 mL/min).

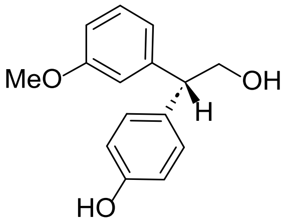

5e'

(*R*)-4-(2-Hydroxy-1-(3-methoxyphenyl)ethyl)phenol (5e')

61% yield, $[\alpha]_D^{25}$ = +2.2 (c 0.5, acetone), a light-yellow oil, ^{1}H NMR (400 MHz, Acetone-d_6) δ 8.14 (s, 1H), 7.18 (t, *J* = 8.1 Hz, 1H), 7.14 – 7.09 (m, 2H), 6.88 – 6.82 (m, 2H), 6.77 – 6.70 (m, 3H), 4.05 (d, *J* = 2.3 Hz, 3H), 3.75 (s, 3H), 3.66 (s, 1H). ^{13}C NMR (101 MHz, Acetone-d_6) δ 160.7, 156.7, 146.0, 134.5, 130.1, 130.0, 121.5, 115.9, 115.2, 111.9, 66.4, 55.3, 54.1. HRMS (ESI) m/z calcd for $C_{15}H_{15}O_3$ $[M-H]^-$ = 243.1027, found = 243.1027; the ee value was 94%, t_R (major) = 9.5 min, t_R (minor) = 27.1 min (Chiralpak IE, λ = 220 nm, 20% *i*-PrOH/Hexane, flow rate = 1 mL/min).

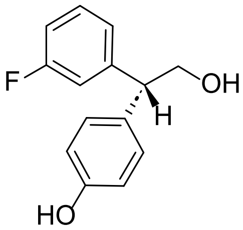

5f'

(*R*)-4-(1-(3-Hluorophenyl)-2-hydroxyethyl)phenol (5f')

99% yield, $[\alpha]_D^{25}$ = +3.8 (c 0.5, acetone), a light-yellow oil, ^{1}H NMR (400 MHz, Acetone-d_6) δ 8.23 (s, 1H), 7.30 (td, *J* = 8.0, 6.2 Hz, 1H), 7.12 (dd, *J* = 8.5, 2.2 Hz, 3H), 7.09 – 7.03 (m, 1H), 6.99 – 6.86 (m, 1H), 6.82 – 6.65 (m, 2H), 4.08 (dt, *J* = 19.0, 6.2 Hz, 3H), 3.84 (d, *J* = 6.3 Hz, 1H). ^{13}C NMR (101 MHz, Acetone-d_6) δ 164.9, 162.5, 156.9, 147.6, 147.5, 134.0, 130.7, 130.6, 130.1, 125.3, 125.3, 116.1, 115.9, 115.7, 113.5, 113.3, 66.2, 53.7, 53.7. HRMS (ESI) m/z calcd for $C_{14}H_{12}FO_2$ $[M-H]^-$ = 231.0827, found = 231.0826; the ee value was 90%, t_R (major) = 5.8 min, t_R (minor) = 7.2 min (Chiralpak IE, λ = 220 nm, 20% *i*-PrOH/Hexane, flow rate = 1 mL/min).

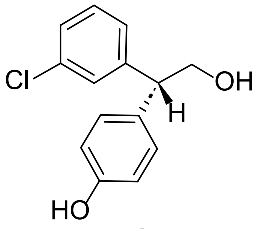

5g'

(*R*)-4-(1-(3-Chlorophenyl)-2-hydroxyethyl)phenol (5g')

97% yield, $[\alpha]_D^{25}$ = +0.8 (c 0.5, acetone), a light-yellow oil, ^{1}H NMR (400 MHz, Acetone-d_6) δ 8.19 (s, 1H), 7.35 – 7.23 (m, 3H), 7.20 (dt, *J* = 7.3, 2.0 Hz, 1H), 7.16 – 7.07 (m, 2H), 6.81 – 6.71 (m, 2H), 4.15 – 3.98 (m, 3H), 3.84 (t, *J* = 4.7 Hz, 1H). ^{13}C NMR (101 MHz, Acetone-d_6) δ 156.9, 147.1, 134.4, 133.9, 130.6, 130.2, 129.2, 127.9, 126.9, 116.1, 66.1, 53.7. HRMS (ESI) m/z calcd for $C_{14}H_{12}ClO_2$ $[M-H]^-$ = 247.0531, found = 247.0527; the ee value was 94%, t_R (major) = 5.3 min, t_R (minor) = 6.1 min (Chiralpak IE, λ = 220 nm, 20% *i*-PrOH/Hexane, flow rate = 1 mL/min).

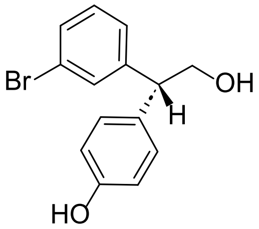

5h'

(*R*)-4-(1-(3-Bromophenyl)-2-hydroxyethyl)phenol (5h')

95% yield, $[\alpha]_D^{25}$ = +3.6 (c 0.5, acetone), a light-yellow oil, ^{1}H NMR (400 MHz, Acetone-d_6) δ 8.21 (s, 1H), 7.48 (t, *J* = 1.9 Hz, 1H), 7.33 (ddt, *J* = 15.3, 7.8, 1.4 Hz, 2H), 7.23 (t, *J* = 7.8 Hz, 1H), 7.18 – 7.03 (m, 2H), 6.85 – 6.69 (m, 2H), 4.19 – 3.96 (m, 3H), 3.86 (q, *J* = 4.4, 3.5 Hz, 1H). ^{13}C NMR (101 MHz, Acetone-d_6) δ 156.9, 147.4, 133.8, 132.2, 131.0, 130.1, 129.8, 128.3, 122.7, 116.1, 66.1, 53.6. HRMS (ESI) m/z calcd for $C_{14}H_{12}BrO_2$ $[M-H]^-$ = 291.0026, found = 291.0024; the ee value was 88%, t_R (major) = 10.4 min, t_R (minor) = 13.5 min (Chiralpak IE, λ = 220 nm, 10% *i*-PrOH/Hexane, flow rate = 1 mL/min).

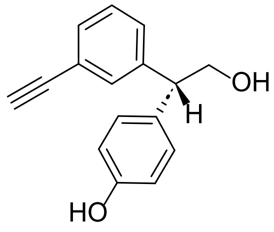

5i'

(*R*)-4-(1-(3-Ethynylphenyl)-2-hydroxyethyl)phenol (5i')

68% yield, $[\alpha]_D^{25}$ = +0.2 (c 0.5, acetone), a light-yellow oil, ^{1}H NMR (400 MHz, Acetone-*d*$_6$) δ 8.41 (s, 1H), 7.42 (s, 1H), 7.38 – 7.22 (m, 3H), 7.11 (d, *J* = 8.4 Hz, 2H), 6.76 (d, *J* = 8.6 Hz, 2H), 4.07 (td, *J* = 9.6, 3.4 Hz, 3H), 3.59 (s, 1H). ^{13}C NMR (101 MHz, Acetone-*d*$_6$) δ 156.9, 145.1, 134.0, 132.7, 130.4, 130.1, 130.0, 129.2, 123.0, 116.0, 84.5, 78.8, 66.1, 53.7. HRMS (ESI) m/z calcd for $C_{16}H_{13}O_2$ $[M-H]^-$ = 237.0921, found = 237.0917; the ee value was 80%, t_R (major) = 5.3 min, t_R (minor) = 6.3 min (Chiralpak IE, λ = 220 nm, 20% *i*-PrOH/Hexane, flow rate = 1 mL/min).

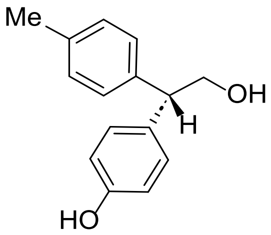

5j'

(*S*)-4-(2-Hydroxy-1-(p-tolyl)ethyl)phenol (5j')

71% yield, $[\alpha]_D^{25}$ = +0.8 (c 0.5, acetone), a light-yellow oil, ^{1}H NMR (400 MHz, Acetone-*d*$_6$) δ 8.33 (s, 1H), 7.15 (d, *J* = 8.1 Hz, 2H), 7.11 – 6.99 (m, 4H), 6.80 – 6.62 (m, 2H), 4.02 (s, 3H), 3.70 (s, 1H), 2.25 (s, 3H). ^{13}C NMR (101 MHz, Acetone-*d*$_6$) δ 156.7, 141.4, 136.1, 134.7, 130.1, 129.6, 129.1, 115.9, 115.8, 66.5, 53.7, 20.9; the ee value was 80%, t_R (major) = 6.5 min, t_R (major) = 9.3 min (Chiralpak IE, λ = 220 nm, 20% *i*-PrOH/Hexane, flow rate = 1 mL/min).

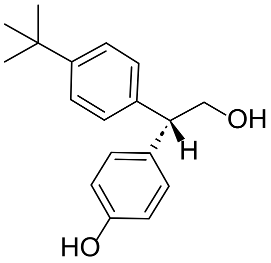

5k'

(*S*)-4-(1-(4-(Tert-butyl)phenyl)-2-hydroxyethyl)phenol (5k')

62% yield, $[\alpha]_D^{25}$ = +2.2 (c 0.5, acetone), a light-yellow oil, ^{1}H NMR (400 MHz, Acetone-d_6) δ 8.32 (s, 1H), 7.30 (d, *J* = 8.3 Hz, 2H), 7.20 (d, *J* = 8.0 Hz, 2H), 7.10 (d, *J* = 8.1 Hz, 2H), 6.74 (d, *J* = 8.6 Hz, 2H), 4.03 (s, 3H), 3.74 (s, 1H), 1.27 (s, 9H). ^{13}C NMR (101 MHz, Acetone-d_6) δ 156.7, 149.3, 141.4, 134.7, 130.1, 128.8, 125.8, 115.9, 115.8, 66.5, 53.7, 34.8, 31.7. HRMS (ESI) m/z calcd for $C_{18}H_{21}O_2$ $[M-H]^-$ = 269.1547, found = 269.1543; the ee value was 91%, t_R (major) = 11.0 min, t_R (minor) = 13.7 min (Chiralpak IE, λ = 220 nm, 10% *i*-PrOH/Hexane, flow rate = 0.8 mL/min).

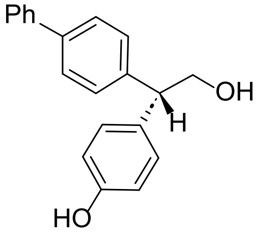

5l'

(*S*)-4-(1-([1,1'-Biphenyl]-4-yl)-2-hydroxyethyl)phenol (5l')

72% yield, $[\alpha]_D^{25}$ = +2.8 (c 0.5, acetone), a light-yellow oil, ^{1}H NMR (400 MHz, CD_3OD) δ 7.61 – 7.56 (m, 2H), 7.56 – 7.49 (m, 2H), 7.45 – 7.36 (m, 2H), 7.35 – 7.31 (m, 2H), 7.31 – 7.26 (m, 1H), 7.16 – 7.04 (m, 2H), 6.79 – 6.65 (m, 2H), 4.16 – 3.98 (m, 3H). ^{13}C NMR (101 MHz, CD_3OD) δ 157.0, 143.5, 142.3, 140.5, 134.6, 130.3, 129.8, 129.8, 128.1, 127.9, 127.9, 116.2, 66.7, 54.0. HRMS (ESI) m/z calcd for $C_{20}H_{17}O_2$ $[M-H]^-$ = 289.1234, found = 289.1231; the ee value was 91%, t_R (minor) = 8.7 min, t_R (major) = 10.2 min (Chiralpak IA, λ = 220 nm, 20% *i*-PrOH/Hexane, flow rate = 1 mL/min).

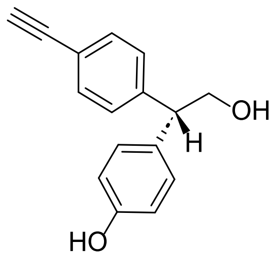

5m'

(*S*)-4-(1-(4-Ethynylphenyl)-2-hydroxyethyl)phenol (5m')

58% yield, $[\alpha]_D^{25}$ = -1.0 (c 0.5, acetone), a light-yellow oil, ^{1}H NMR (400 MHz, Acetone-d_6) δ 8.17 (s, 1H), 7.40 (d, *J* = 8.3 Hz, 2H), 7.31 (d, *J* = 8.3 Hz, 2H), 7.10 (d, *J* = 8.5 Hz, 2H), 6.76 (d, *J* = 8.6 Hz, 2H), 4.08 (dt, *J* = 13.7, 5.7 Hz, 3H), 3.77 (s, 1H), 3.57 (s, 1H). ^{13}C NMR (101 MHz, Acetone-d_6) δ 156.8, 145.6, 134.1, 132.6, 130.2, 129.5, 120.8, 116.0, 84.3, 78.6, 66.2, 53.9. HRMS (ESI) m/z calcd for $C_{16}H_{13}O_2$ $[M-H]^-$ = 237.0921, found = 237.0922; the ee value was 93%, t_R (major) = 16.7 min, t_R (minor) = 18.9 min (Chiralpak IE, λ = 220 nm, 10% *i*-PrOH/Hexane, flow rate = 1 mL/min).

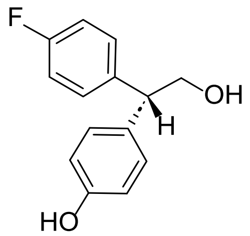

5n'

(*R*)-4-(1-(4-Fluorophenyl)-2-hydroxyethyl)phenol (5n')

79% yield, $[\alpha]_D^{25}$ = +3.0 (c 0.5, acetone), a light-yellow oil, ^{1}H NMR (400 MHz, Acetone-d_6) δ 8.37 (s, 1H), 7.31 (dd, *J* = 8.4, 5.6 Hz, 2H), 7.09 (d, *J* = 8.2 Hz, 2H), 7.02 (t, *J* = 8.7 Hz, 2H), 6.75 (d, *J* = 8.2 Hz, 2H), 4.20 – 3.99 (m, 3H), 3.87 (d, *J* = 5.5 Hz, 1H). ^{13}C NMR (101 MHz, Acetone-d_6) δ 163.3, 160.9, 156.8, 140.6, 140.5, 134.3, 130.9, 130.8, 130.0, 116.0, 115.9, 115.6, 115.4, 66.4, 53.2. HRMS (ESI) m/z calcd for $C_{14}H_{12}FO_2$ $[M-H]^-$ = 231.0827, found = 231.0825; the ee value was 75%, t_R (major) = 12.9 min, t_R (minor) = 14.4 min (Chiralpak IE, λ = 220 nm, 10% *i*-PrOH/Hexane, flow rate = 0.8 mL/min).

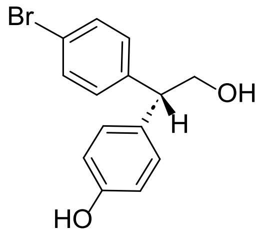

5o'

(*R*)-4-(1-(4-Bromophenyl)-2-hydroxyethyl)phenol (5o')

96% yield, $[\alpha]_D^{25}$ = -0.8 (c 0.5, acetone), a light-yellow oil, ^{1}H NMR (400 MHz, Acetone-*d*$_6$) δ 8.40 (s, 1H), 7.43 (d, *J* = 8.4 Hz, 2H), 7.25 (d, *J* = 8.4 Hz, 2H), 7.09 (d, *J* = 8.5 Hz, 2H), 6.75 (d, *J* = 8.6 Hz, 2H), 4.12 – 3.87 (m, 4H). ^{13}C NMR (101 MHz, Acetone-*d*$_6$) δ 156.9, 144.0, 133.9, 131.9, 131.4, 130.1 120.1, 116.1, 116.0, 66.2, 66.0, 53.4, 53.3. HRMS (ESI) m/z calcd for $C_{14}H_{12}BrO_2$ [M-H]$^-$ = 291.0026, found = 291.0022; the ee value was 95%, t_R (minor) = 4.9 min, t_R (major) = 5.9 min (Chiralpak IE, λ = 220 nm, 20% *i*-PrOH/Hexane, flow rate = 1 mL/min).

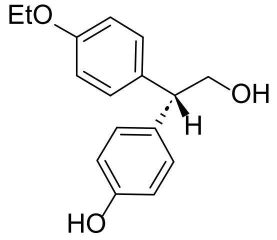

5p'

(*R*)-4-(1-(4-Ethoxyphenyl)-2-hydroxyethyl)phenol (5p')

72% yield, $[\alpha]_D^{25}$ = -17.8 (c 0.5, acetone), a light-yellow oil, ^{1}H NMR (400 MHz, Acetone-*d*$_6$) δ 8.14 (s, 1H), 7.17 (d, *J* = 8.7 Hz, 2H), 7.09 (d, *J* = 8.6 Hz, 2H), 6.81 (d, *J* = 8.7 Hz, 2H), 6.74 (d, *J* = 8.6 Hz, 2H), 4.25 – 3.86 (m, 6H), 1.33 (s, 3H). ^{13}C NMR (101 MHz, Acetone-*d*$_6$) δ 158.3, 136.3, 135.0, 130.1, 130.1, 129.9, 115.9, 115.0, 66.7, 63.8, 53.3, 15.2. HRMS (ESI) m/z calcd for $C_{16}H_{17}O_3$ [M-H]$^-$ = 257.1183, found = 257.1184; the ee value was 92%, t_R (major) = 8.1 min, t_R (minor) = 14.2 min (Chiralpak IE, λ = 220 nm, 20% *i*-PrOH/Hexane, flow rate = 1 mL/min).

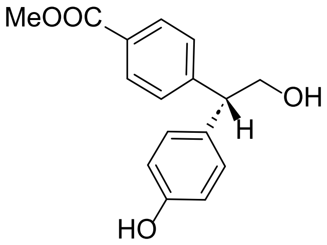

5q'

Methyl (*S*)-4-(2-hydroxy-1-(4-hydroxyphenyl)ethyl)benzoate (5q')

99% yield, $[\alpha]_D^{25}$ = +0.6 (c 0.5, acetone), a light-yellow oil, ^{1}H NMR (400 MHz, Acetone-d_6) δ 8.44 (s, 1H), 7.91 (d, *J* = 8.2 Hz, 2H), 7.43 (d, *J* = 8.1 Hz, 2H), 7.10 (d, *J* = 8.4 Hz, 2H), 6.75 (d, *J* = 8.5 Hz, 2H), 4.22 – 3.88 (m, 4H), 3.85 (s, 3H). ^{13}C NMR (101 MHz, Acetone-d_6) δ 167.3, 156.9, 150.1, 133.7, 130.1, 130.1, 129.5, 128.9, 116.1, 66.1, 54.0, 52.2. HRMS (ESI) m/z calcd for $C_{16}H_{15}O_4$ $[M-H]^-$ = 271.0976, found = 271.0972; the ee value was 91%, t_R (major) = 16.6 min, t_R (minor) = 30.0 min (Chiralpak IE, λ = 220 nm, 20% *i*-PrOH/Hexane, flow rate = 1 mL/min).

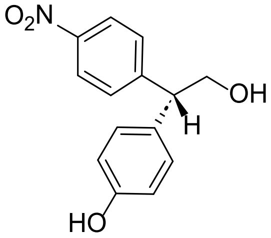

5r'

(*S*)-4-(2-Hydroxy-1-(4-nitrophenyl)ethyl)phenol (5r')

75% yield, $[\alpha]_D^{25}$ = +18.0 (c 0.5, acetone), a light-yellow oil, ^{1}H NMR (400 MHz, CD_3OD) δ 8.15 (d, *J* = 8.7 Hz, 2H), 7.51 (d, *J* = 8.7 Hz, 2H), 7.07 (d, *J* = 8.6 Hz, 2H), 6.73 (d, *J* = 8.5 Hz, 2H), 4.20 (t, *J* = 7.3 Hz, 1H), 4.08 (qd, *J* = 10.9, 7.3 Hz, 2H). ^{13}C NMR (101 MHz, CD_3OD) δ 157.4, 152.5, 147.9, 133.2, 130.5, 130.3, 124.4, 116.5, 66.1, 54.1. HRMS (ESI) m/z calcd for $C_{14}H_{12}NO_4$ $[M-H]^-$ = 258.0772, found = 258.0768; the ee value was 64%, t_R (major) = 12.3 min, t_R (minor) = 13.7 min (Chiralpak IA, λ = 220 nm, 20% *i*-PrOH/Hexane, flow rate = 1 mL/min).

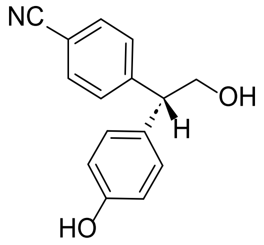

5s'

(*S*)-4-(2-Hydroxy-1-(4-hydroxyphenyl)ethyl)benzonitrile (5s')

90% yield, $[\alpha]_D^{25}$ = +6.4 (c 0.5, acetone), a light-yellow oil, ^{1}H NMR (400 MHz, Acetone-*d*$_6$) δ 7.74 – 7.59 (m, 1H), 7.59 – 7.41 (m, 1H), 7.17 – 6.94 (m, 1H), 6.87 – 6.61 (m, 1H), 4.22 – 3.96 (m, 2H), 3.35 – 2.60 (m, 2H). ^{13}C NMR (101 MHz, Acetone-*d*$_6$) δ 157.1, 150.4, 133.3, 132.8, 130.4, 130.1, 119.5, 116.2, 110.6, 65.9, 54.0. HRMS (ESI) m/z calcd for $C_{15}H_{12}NO_2$ $[M-H]^-$ = 238.0874, found = 238.0876; the ee value was 75%, t_R (major) = 26.6 min, t_R (minor) = 28.5 min (Chiralpak IE, λ = 220 nm, 10% *i*-PrOH/Hexane, flow rate = 1 mL/min).

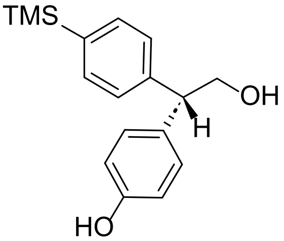

5t'

(*R*)-4-(2-Hydroxy-1-(4-(trimethylsilyl)phenyl)ethyl)phenol (5t')

74% yield, $[\alpha]_D^{25}$ = +3.8 (c 0.5, acetone), a light-yellow oil, ^{1}H NMR (400 MHz, Acetone-*d*$_6$) δ 8.14 (s, 1H), 7.44 (d, *J* = 8.2 Hz, 2H), 7.28 (d, *J* = 8.1 Hz, 2H), 7.12 (d, *J* = 8.6 Hz, 2H), 6.75 (d, *J* = 8.6 Hz, 2H), 4.19 – 3.99 (m, 3H), 3.69 (q, *J* = 4.3, 3.8 Hz, 1H), 0.23 (s, 9H). ^{13}C NMR (101 MHz, Acetone-*d*$_6$) δ 156.7, 145.2, 138.1, 134.5, 134.1, 130.1, 128.7, 115.9, 66.4, 54.2, 1.0. HRMS (ESI) m/z calcd for $C_{17}H_{21}O_2Si$ $[M-H]^-$ = 285.1316, found = 285.1314; the ee value was 95%, t_R (major) = 7.4 min, t_R (minor) = 8.6 min (Chiralpak IE, λ = 220 nm, 10% *i*-PrOH/Hexane, flow rate = 1 mL/min).

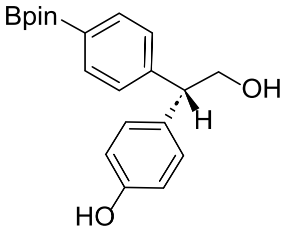

5u'

(*S*)-4-(2-Hydroxy-1-(4-(4,4,5,5-tetramethyl-1,3,2-dioxaborolan-2-yl)phenyl)ethyl)phenol (5u')

82% yield, $[\alpha]_D^{25}$ = -1.4 (c 0.5, acetone), a light-yellow oil, ^{1}H NMR (400 MHz, CD_3OD) δ 7.76 – 7.60 (m, 2H), 7.26 (d, *J* = 8.0 Hz, 2H), 7.15 – 6.96 (m, 2H), 6.71 (d, *J* = 8.6 Hz, 2H), 4.19 – 3.92 (m, 3H), 1.33 (s, 13H). ^{13}C NMR (101 MHz, CD_3OD) δ 157.0, 147.8, 135.8, 134.3, 130.3, 128.8, 116.2, 85.0, 66.5, 54.5, 25.2; the ee value was 82%, t_R (major) = 7.9 min, t_R (minor) = 9.6 min (Chiralpak IE, λ = 220 nm, 20% *i*-PrOH/Hexane, flow rate = 1 mL/min).

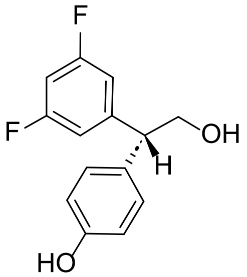

6a'

(*R*)-4-(1-(3,5-Difluorophenyl)-2-hydroxyethyl)phenol (6a')

84% yield, $[\alpha]_D^{25}$ = -12.6 (c 0.5, acetone), a light-yellow oil, ^{1}H NMR (400 MHz, Acetone-d_6) δ 8.21 (s, 1H), 7.53 – 7.40 (m, 1H), 7.10 (d, *J* = 8.6 Hz, 2H), 7.00 – 6.86 (m, 2H), 6.76 (d, *J* = 8.6 Hz, 2H), 4.36 (t, *J* = 7.3 Hz, 1H), 4.06 (p, *J* = 10.6, 10.1 Hz, 2H), 3.90 (s, 1H). ^{13}C NMR (101 MHz, Acetone-d_6) δ 163.6, 163.0, 161.1, 160.7, 156.9, 133.0, 131.4, 131.3, 131.3, 131.2, 130.1, 127.5, 127.4, 127.3, 116.0, 111.8, 111.8, 111.6, 111.6, 104.4, 104.2, 104.2, 103.9, 65.3, 46.2. HRMS (ESI) m/z calcd for $C_{14}H_{11}F_2O_2$ $[M-H]^-$ = 249.0733., found = 249.0733; the ee value was 91%, t_R (major) = 7.9 min, t_R (minor) = 10.8 min (Chiralpak IE, λ = 220 nm, 10% *i*-PrOH/Hexane, flow rate = 1 mL/min).

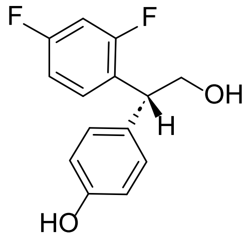

6b'

(*R*)-4-(1-(2,4-Difluorophenyl)-2-hydroxyethyl)phenol (6b')

88% yield, $[\alpha]_D^{25}$ = +3.0 (c 0.5, acetone), a light-yellow oil, ^{1}H NMR (400 MHz, Acetone-d_6) δ 8.23 (s, 1H), 7.13 (d, *J* = 8.6 Hz, 2H), 6.96 (d, *J* = 6.9 Hz, 2H), 6.78 (d, *J* = 8.5 Hz, 3H), 4.10 (dq, *J* = 25.3, 6.9, 6.3 Hz, 4H), 3.91 (s, 1H). ^{13}C NMR (101 MHz, Acetone-d_6) δ 165.1, 164.9, 162.6, 162.5, 157.1, 149.5, 133.3, 130.1, 116.2, 112.3, 112.2, 112.1, 112.0, 102.2, 101.9, 101.7, 65.9, 53.6, 53.6. HRMS (ESI) m/z calcd for $C_{14}H_{11}F_2O_2$ [M-H]$^-$ = 249.0733., found = 249.0732; the ee value was 89%, t_R (major) = 21.3 min, t_R (minor) = 23.4 min (Chiralpak IE, λ = 220 nm, 5% *i*-PrOH/Hexane, flow rate = 1 mL/min).

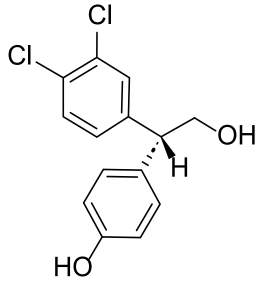

6c'

(*R*)-4-(1-(3,4-Dichlorophenyl)-2-hydroxyethyl)phenol (6c')

90% yield, $[\alpha]_D^{25}$ = +0.8 (c 0.5, acetone), a light-yellow oil, ^{1}H NMR (400 MHz, Acetone-d_6) δ 8.31 – 8.13 (m, 1H), 7.50 (d, *J* = 2.1 Hz, 1H), 7.46 (d, *J* = 8.3 Hz, 1H), 7.28 (dd, *J* = 8.3, 2.1 Hz, 1H), 7.18 – 7.03 (m, 2H), 6.84 – 6.67 (m, 2H), 4.21 – 3.98 (m, 3H), 3.93 (d, *J* = 6.6 Hz, 1H). ^{13}C NMR (101 MHz, Acetone-d_6) δ 157.0, 145.8, 133.5, 132.2, 131.3, 131.0, 130.1, 130.1, 129.5, 116.2, 66.0, 53.0. HRMS (ESI) m/z calcd for $C_{14}H_{11}F_2O_2$ [M-H]$^-$ = 281.0142., found = 281.0140; the ee value was 81%, t_R (major) = 9.7 min, t_R (minor) = 13.1 min (Chiralpak IE, λ = 220 nm, 10% *i*-PrOH/Hexane, flow rate = 1 mL/min).

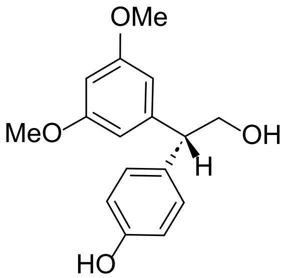

6d'

(*R*)-4-(1-(3,5-Dimethoxyphenyl)-2-hydroxyethyl)phenol (6d')

60% yield, $[\alpha]_D^{25}$ = +0.6 (c 0.5, acetone), a light-yellow oil, ^{1}H NMR (400 MHz, Acetone-d_6) δ 8.15 (s, 1H), 7.18 – 7.07 (m, 2H), 6.79 – 6.68 (m, 2H), 6.46 (d, *J* = 2.3 Hz, 2H), 6.31 (t, *J* = 2.3 Hz, 1H), 4.03 (dq, *J* = 9.6, 4.8, 4.3 Hz, 3H), 3.73 (s, 6H), 3.67 (d, *J* = 12.8 Hz, 1H). ^{13}C NMR (101 MHz, Acetone-d_6) δ 161.8, 156.7, 146.7, 134.4, 130.1, 115.9, 107.4, 98.4, 66.4, 55.4, 54.4. HRMS (ESI) m/z calcd for $C_{16}H_{17}O_4$ $[M-H]^-$ = 273.1132., found = 273.1131; the ee value was 94%, t_R (major) = 9.3 min, t_R (minor) = 11.7 min (Chiralpak IA, λ = 220 nm, 20% *i*-PrOH/Hexane, flow rate = 1 mL/min).

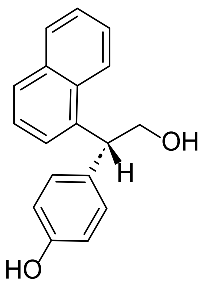

6e'

(*R*)-4-(2-Hydroxy-1-(naphthalen-1-yl)ethyl)phenol (6e')

58% yield, $[\alpha]_D^{25}$ = +2.6 (c 0.5, acetone), a light-yellow oil, ^{1}H NMR (400 MHz, Acetone-d_6) δ 8.21 – 8.11 (m, 1H), 7.88 (s, 1H), 7.78 (d, *J* = 8.2 Hz, 1H), 7.59 (d, *J* = 7.2 Hz, 1H), 7.50 (t, *J* = 7.7 Hz, 1H), 7.43 (dd, *J* = 6.5, 3.3 Hz, 2H), 7.14 (d, *J* = 8.4 Hz, 2H), 6.72 (d, *J* = 8.5 Hz, 2H), 4.90 (t, *J* = 7.1 Hz, 1H), 4.19 (ddd, *J* = 38.3, 11.0, 7.2 Hz, 2H). ^{13}C NMR (101 MHz, Acetone-d_6) δ 156.8, 139.4, 135.1, 134.4, 133.2, 130.3, 129.5, 127.6, 126.6, 126.2, 126.1, 125.5, 124.8, 115.9, 66.5, 49.2. HRMS (ESI) m/z calcd for $C_{18}H_{15}O_2$ $[M-H]^-$ = 263.1078., found = 263.1073; the ee value was 62%, t_R (minor) = 5.8 min, t_R (major) = 6.3 min (Chiralpak IE, λ = 220 nm, 20% *i*-PrOH/Hexane, flow rate = 1 mL/min).

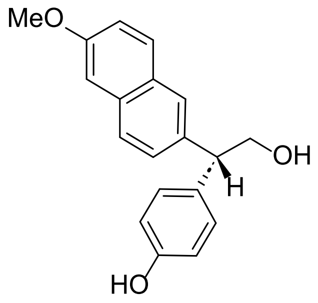

6f'

(*R*)-4-(2-Hydroxy-1-(6-methoxynaphthalen-2-yl)ethyl)phenol (6f')

61% yield, $[\alpha]_D^{25}$ = +16.0 (c 0.5, acetone), a light-yellow oil, ^{1}H NMR (400 MHz, Acetone-d_6) δ 7.80 – 7.64 (m, 2H), 7.35 (dd, J = 8.5, 1.8 Hz, 1H), 7.23 (d, J = 2.6 Hz, 1H), 7.17 – 7.07 (m, 2H), 6.83 – 6.69 (m, 1H), 4.25 – 4.06 (m, 2H), 3.88 (s, 3H). ^{13}C NMR (101 MHz, Acetone-d_6) δ 160.7, 160.0, 153.5, 146.0, 145.0, 141.6, 140.1, 130.2, 130.0, 128.6, 127.5, 127.1, 119.3, 114.6, 111.2, 106.4, 67.5, 58.9, 55.2. HRMS (ESI) m/z calcd for $C_{19}H_{17}O_3$ $[M-H]^-$ = 293.1183., found = 293.1179; the ee value was 92%, t_R (major) = 10.5 min, t_R (minor) = 17.3 min (Chiralpak IE, λ = 220 nm, 20% *i*-PrOH/Hexane, flow rate = 1 mL/min).

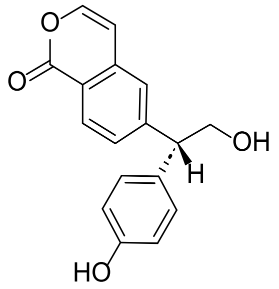

6g'

(*R*)-6-(2-Hydroxy-1-(4-hydroxyphenyl)ethyl)-1H-isochromen-1-one (6g')

65% yield, $[\alpha]_D^{25}$ = +1.6 (c 0.5, acetone), a light-yellow oil, ^{1}H NMR (400 MHz, Acetone-d_6) δ 8.23 (s, 1H), 7.92 (dd, J = 9.5, 0.6 Hz, 1H), 7.56 (d, J = 7.9 Hz, 1H), 7.34 – 7.23 (m, 2H), 7.22 – 7.07 (m, 2H), 6.82 – 6.69 (m, 2H), 6.34 (d, J = 9.5 Hz, 1H), 4.28 – 4.05 (m, 3H), 3.93 (s, 1H). ^{13}C NMR (101 MHz, Acetone-d_6) δ 160.8, 157.0, 155.1, 149.6, 144.4, 133.6, 130.2, 128.8, 125.8, 118.0, 116.8, 116.4, 116.2, 79.2, 66.0, 54.0. HRMS (ESI) m/z calcd for $C_{17}H_{13}O_4$ $[M-H]^-$ = 281.0819., found = 281.0817; the ee value was 80%, t_R (major) = 25.3 min, t_R (minor) = 34.5 min (Chiralpak IA, λ = 220 nm, 20% *i*-PrOH/Hexane, flow rate = 1 mL/min).

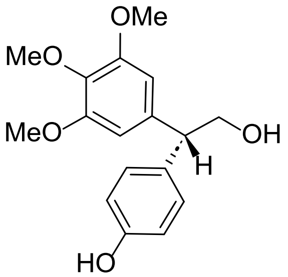

6h'

(*R*)-4-(2-Hydroxy-1-(3,4,5-trimethoxyphenyl)ethyl)phenol (6h')

68% yield, $[\alpha]_D^{25}$ = +2.0 (c 0.5, acetone), a light-yellow oil, ^{1}H NMR (400 MHz, Acetone-d_6) δ 8.35 (s, 1H), 7.13 (d, *J* = 8.5 Hz, 2H), 6.74 (d, *J* = 8.5 Hz, 2H), 6.60 (s, 2H), 4.09 – 3.99 (m, 3H), 3.77 (s, 6H), 3.67 (s, 3H). ^{13}C NMR (101 MHz, Acetone-d_6) δ 156.7, 154.1, 140.0, 137.6, 134.5, 130.0, 115.9, 115.8, 106.7, 66.4, 60.4, 56.4, 54.3. HRMS (ESI) m/z calcd for $C_{18}H_{23}O_4$ $[M-H]^-$ = 303.1591., found = 303.1596; the ee value was 83%, t_R (minor) = 5.9 min, t_R (major) = 6.5 min (Chiralpak IE, λ = 220 nm, 40% *i*-PrOH/Hexane, flow rate = 1 mL/min).

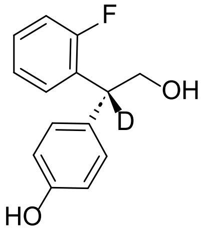

6i'

(*R*)-4-(1-(2-Fluorophenyl)-2-hydroxyethyl-1-d)phenol (6i')

99% yield, a light-yellow oil, ^{1}H NMR (400 MHz, Acetone-d_6) δ 8.17 (s, 1H), 7.43 (td, *J* = 7.6, 1.8 Hz, 1H), 7.23 (dddd, *J* = 8.1, 7.2, 5.2, 1.8 Hz, 1H), 7.18 – 7.08 (m, 3H), 7.04 (ddd, *J* = 10.7, 8.1, 1.4 Hz, 1H), 6.82 – 6.66 (m, 2H), 4.08 (qd, *J* = 10.8, 5.5 Hz, 2H), 3.83 (t, *J* = 5.6 Hz, 1H). ^{13}C NMR (101 MHz, Acetone-d_6) δ 163.1, 160.7, 156.9, 133.3, 131.1, 131.0, 130.3, 130.2, 130.1, 128.7, 128.6, 124.9, 124.9, 116.0, 116.0, 115.8, 65.4, 46.7, 46.3. HRMS (ESI) m/z calcd for $C_{14}H_{11}DFO_2$ $[M-H]^-$ = 232.0890., found = 232.0890; the ee value was 91%, t_R (major) = 10.5 min, t_R (minor) = 12.5 min (Chiralpak IE, λ = 220 nm, 10% *i*-PrOH/Hexane, flow rate = 1 mL/min).

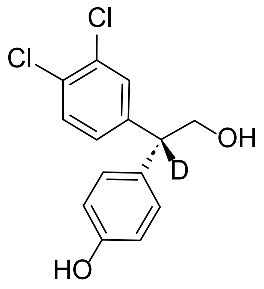

6j'

(*R*)-4-(1-(3,4-Dichlorophenyl)-2-hydroxyethyl-1-d)phenol (6j')

92% yield, a light-yellow oil; the ee value was 77%, t_R (major) = 9.8 min, t_R (minor) = 13.3 min (Chiralpak IE, λ = 220 nm, 10% *i*-PrOH/Hexane, flow rate = 1 mL/min).

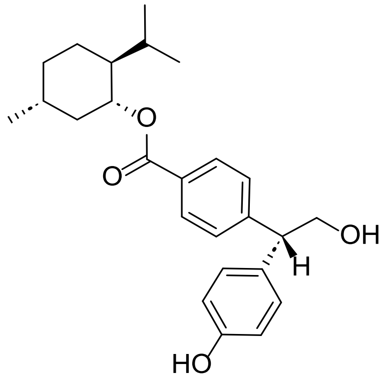

6k'

(1*R*,2*S*,5*R*)-2-Isopropyl-5-methylcyclohexyl 4-((S)-2-hydroxy-1-(4-hydroxyphenyl)ethyl)benzoate (6k')

95% yield, $[\alpha]_D^{25}$ = -64.6 (c 0.5, acetone), a light-yellow oil, ^1H NMR (400 MHz, Acetone-d_6) δ 8.19 (s, 1H), 7.99 – 7.86 (m, 2H), 7.44 (d, *J* = 8.3 Hz, 2H), 7.12 (d, *J* = 8.5 Hz, 2H), 6.81 – 6.71 (m, 2H), 4.90 (td, *J* = 10.9, 4.4 Hz, 1H), 4.23 – 4.00 (m, 3H), 3.83 (t, *J* = 5.4 Hz, 1H), 1.95 (tdd, *J* = 9.8, 7.0, 2.7 Hz, 1H), 1.74 (dt, *J* = 11.4, 2.9 Hz, 2H), 1.57 (ddt, *J* = 12.6, 11.2, 3.3 Hz, 2H), 1.24 – 1.03 (m, 2H), 1.02 – 0.94 (m, 1H), 0.92 (dd, *J* = 8.3, 6.8 Hz, 6H), 0.78 (d, *J* = 7.0 Hz, 3H). ^{13}C NMR (101 MHz, Acetone-d_6) δ 166.2, 156.9, 150.0, 133.8, 130.2, 130.1, 129.5, 129.5, 116.6, 116.1, 74.9, 66.1, 54.0, 48.1, 41.8, 35.0, 32.2, 27.3, 24.3, 22.3, 21.0, 16.8. HRMS (ESI) m/z calcd for $C_{25}H_{31}O_4$ $[M-H]^-$ = 395.2228., found = 395.2223.

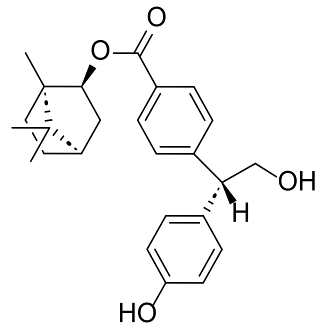

6l'

(1*R*,2*S*,4*R*)-1,7,7-Trimethylbicyclo[2.2.1]heptan-2-yl 4-((S)-2-hydroxy-1-(4-hydroxyphenyl)ethyl)benzoate (6l')

88% yield, $[\alpha]_D^{25}$ = -28.6 (c 0.5, acetone), a light-yellow oil, ^{1}H NMR (400 MHz, Acetone-*d6*) δ 8.21 (s, 1H), 7.96 (d, *J* = 8.4 Hz, 2H), 7.45 (d, *J* = 8.3 Hz, 2H), 7.12 (d, *J* = 8.5 Hz, 2H), 6.76 (d, *J* = 8.6 Hz, 2H), 5.09 (ddd, *J* = 9.9, 3.5, 2.2 Hz, 1H), 4.24 – 4.00 (m, 3H), 3.84 (s, 1H), 2.50 – 2.35 (m, 1H), 2.16 (ddd, *J* = 12.7, 9.5, 4.5 Hz, 1H), 1.82 (ddq, *J* = 11.1, 7.9, 3.8 Hz, 1H), 1.73 (t, *J* = 4.5 Hz, 1H), 1.51 – 1.37 (m, 1H), 1.37 – 1.25 (m, 1H), 1.10 (dd, *J* = 13.7, 3.5 Hz, 1H), 0.98 (s, 3H), 0.92 (d, *J* = 10.8 Hz, 6H). ^{13}C NMR (101 MHz, Acetone-*d6*) δ 166.9, 156.9, 150.0, 133.8, 130.2, 130.1, 129.6, 129.5, 116.1, 80.6, 66.2, 54.0, 49.7, 48.6, 45.8, 37.6, 28.6, 28.0, 20.0, 19.2, 13.9. HRMS (ESI) m/z calcd for $C_{25}H_{29}O_4$ $[M-H]^-$ = 393.2071., found = 393.2066.

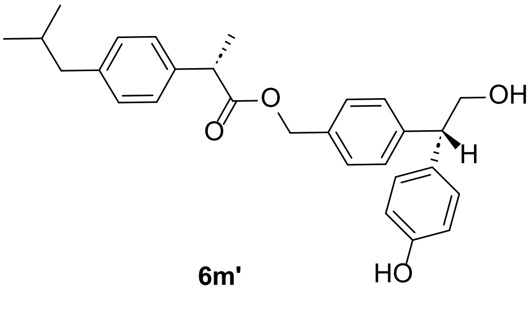

6m'

4-((*S*)-2-Hydroxy-1-(4-hydroxyphenyl)ethyl)benzyl (S)-2-(4-isobutylphenyl)propanoate (6m')

86% yield, $[\alpha]_D^{25}$ = +5.0 (c 0.5, acetone), a light-yellow oil, ^{1}H NMR (400 MHz, Acetone-*d6*) δ 8.16 (s, 1H), 7.27 – 7.13 (m, 6H), 7.10 (dd, *J* = 8.5, 2.4 Hz, 4H), 6.78 – 6.70 (m, 2H), 4.11 – 3.98 (m, 3H), 3.78 (q, *J* = 7.1 Hz, 1H), 3.73 – 3.66 (m, 1H), 2.44 (d, *J* = 7.2 Hz, 2H), 1.84 (dp, *J* = 13.5, 6.8 Hz, 1H), 1.43 (d, *J* = 7.1 Hz, 3H), 0.88 (d, *J* = 6.6 Hz, 6H). ^{13}C NMR (101 MHz, Acetone-*d6*) δ 174.7, 156.8, 144.3, 141.2, 139.2, 135.1, 134.4, 130.1, 130.1, 129.3, 128.6, 128.1,

116.0, 66.5, 66.4, 53.8, 45.7, 45.5, 31.0, 22.6, 19.0. HRMS (ESI) m/z calcd for $C_{28}H_{31}O_4$ [M-H]$^-$ = 431.2228., found = 431.2222.

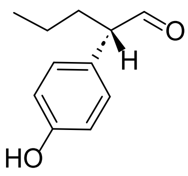

6n

(*S*)-2-(4-Hydroxyphenyl)pentanal (6n)

72% yield, a light-yellow oil, ^{1}H NMR (400 MHz, $CDCl_3$) δ 9.62 (d, *J* = 2.2 Hz, 1H), 7.05 (d, *J* = 8.5 Hz, 2H), 6.84 (d, *J* = 8.6 Hz, 2H), 5.33 (s, 1H), 3.45 (ddd, *J* = 8.5, 6.6, 2.2 Hz, 1H), 2.00 (dtd, *J* = 14.3, 8.0, 6.7 Hz, 1H), 1.67 (dq, *J* = 13.6, 8.0 Hz, 1H), 1.31 – 1.24 (m, 2H), 0.91 (t, *J* = 7.3 Hz, 3H). ^{13}C NMR (101 MHz, $CDCl_3$) δ 201.5, 155.1, 130.0, 128.3, 115.9, 58.0, 31.7, 20.2, 13.9； the ee value was 40%, t_R (major) = 29.1 min, t_R (minor) = 31.0 min (Chiralpak IA, λ = 220 nm, 5% *i*-PrOH/Hexane, flow rate = 1 mL/min).

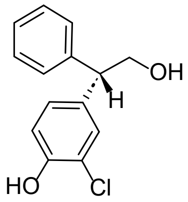

6o'

(*S*)-2-Chloro-4-(2-hydroxy-1-phenylethyl)phenol (6o')

82% yield, a colorless oil, ^{1}H NMR (400 MHz, $CDCl_3$) δ 7.30 – 7.24 (m, 2H), 7.18 – 7.13 (m, 4H), 7.01 (dd, *J* = 8.4, 2.2 Hz, 1H), 6.89 (d, *J* = 8.4 Hz, 1H), 4.05 (t, *J* = 1.4 Hz, 3H). ^{13}C NMR (126 MHz, $CDCl_3$) δ 150.2, 141.0, 128.9, 128.7, 128.3, 128.2, 127.1, 119.8, 116.4, 66.1, 52.6; the ee value was 21%, t_R (major) = 7.4 min, t_R (minor) = 7.9 min (Chiralpak IA, λ = 220 nm, 20% *i*-PrOH/Hexane, flow rate = 1 mL/min).

4. Mechanistic Sudies

(a) Deuterium experiments

Figure S3. Procedure for the synthesis of **7a**.

i)

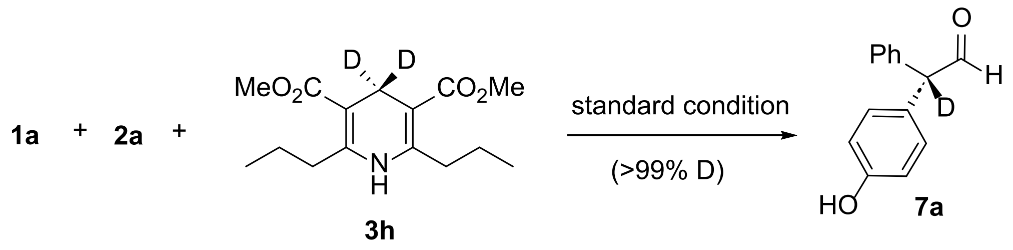

To a dried 1.5 mL screw-cap vial equipped with a magnetic stir bar were added alkyne **1a** (0.1 mmol) and benzoquinone **2a** (5.4 mg, 0.05 mmol) in CH_2Cl_2 (0.5 mL). The mixture was then irradiated by 440 nm Kessil LEDs at room temperature for 2 h. At -80 °C, CPA **4a** (1.9 mg, 5 mol%) in CH_2Cl_2 (2.5 mL)/ether (2.0 mL) was added to the mixture in step I, then **3h** (16.9mg, 0.06 mmol) was added and the resulting mixture was stirred for 12 h. Addition of $NaBH_4$ (1.5 equiv) in MeOH to the reaction mixture containing aldehyde **7a** gave the according alcohol product, which was obtained by silica gel column chromatography, giving the according deuterated product in >99% deuteration ratio determined by NMR spectrum.

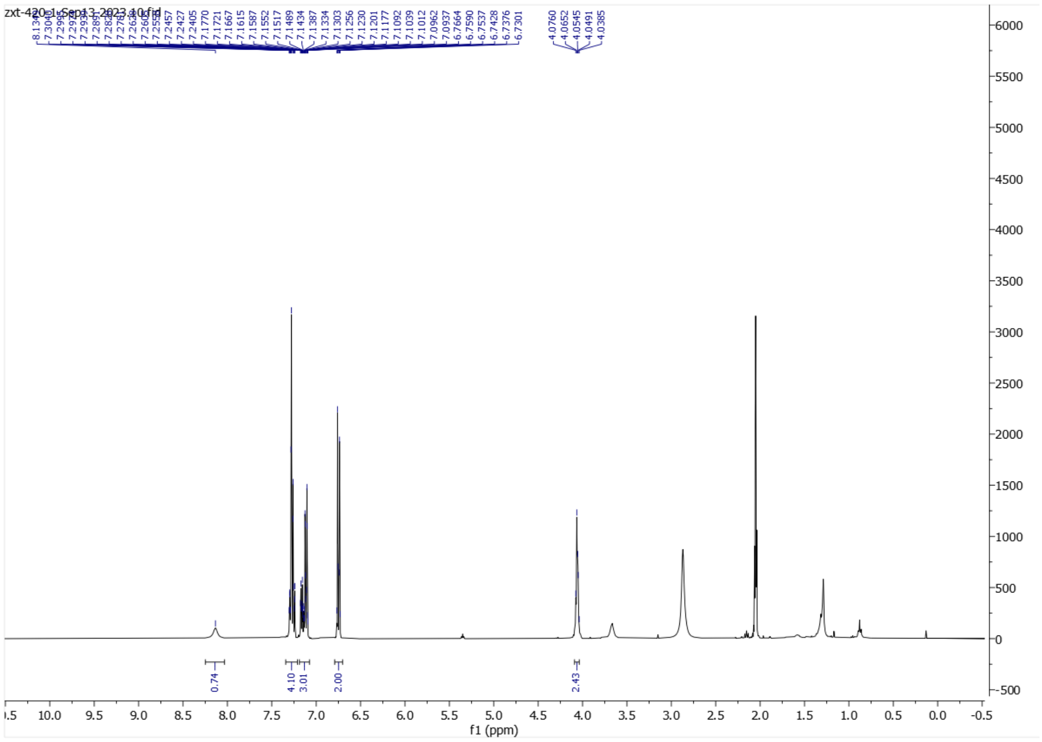

Figure S4. Procedure for the synthesis of **5a**.

ii)

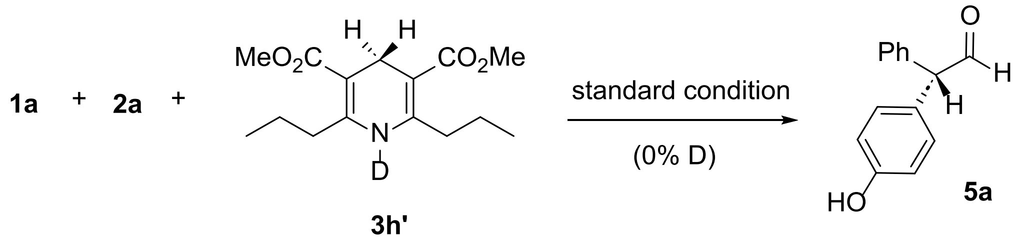

To a dried 1.5 mL screw-cap vial equipped with a magnetic stir bar were added alkyne 1a (0.1 mmol) and benzoquinone 2a (5.4 mg, 0.05 mmol) in CH_2Cl_2 (0.5 mL). The mixture was then irradiated by 440 nm Kessil LEDs at room temperature for 2 h. At -80 °C, CPA **4a** (1.9 mg, 5 mol%) in CH_2Cl_2 (2.5 mL)/ether (2.0 mL) was added to the mixture in step I, then **3h'** (16.9mg, 0.06 mmol) was added and the resulting mixture was stirred for 12 h. Addition of $NaBH_4$ (1.5 equiv) in MeOH to the reaction mixture containing aldehyde **5a** gave the according alcohol product, which was obtained by silica gel column chromatography, giving the according deuterated product in 0% deuteration ratio determined by NMR spectrum.

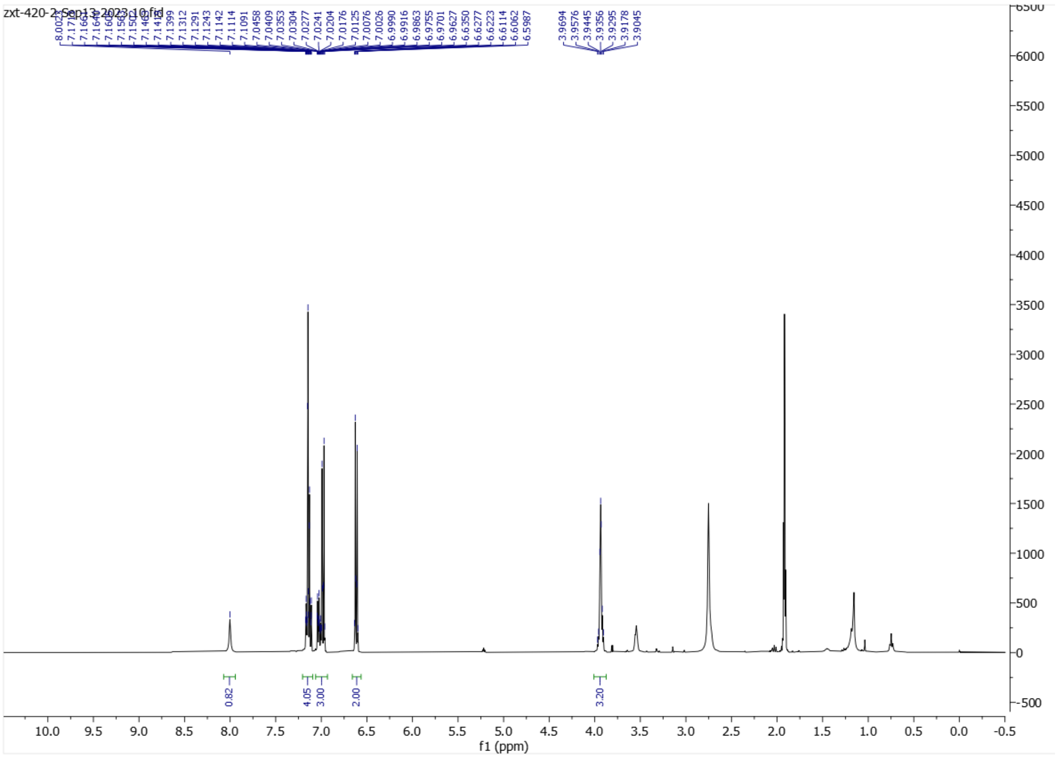

(b) The key intermediate

Figure S5. Procedure for the detection of **8a**.

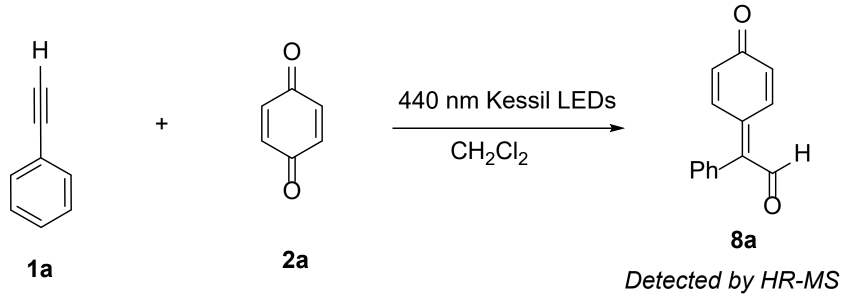

Meas. m/z	#	Formula	Calc. Mass	Err [ppm]
211.0758	1	C14 H11 O2	211.0754	1.90

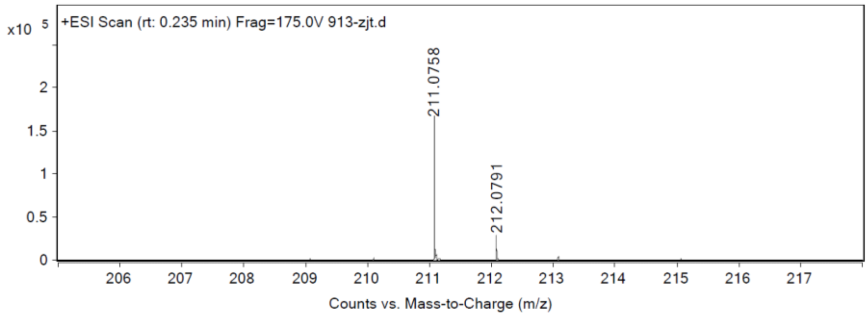

Using the irradiation of 440 nm Kessil LEDs, alkyne **1a** and benzoquinone **2a** generated *p*-QM **8a** which was detected by high-resolution mass spectrometry (HRMS); HRMS (ESI) m/z calcd for $C_{14}H_{11}O_2$ $[M+H]^+$ = 211.0754, found = 211.0758.

5. Synthetic Applications

(a) *Figure S6.* Downstream transformation for detosylation and cross-coupling reactions.

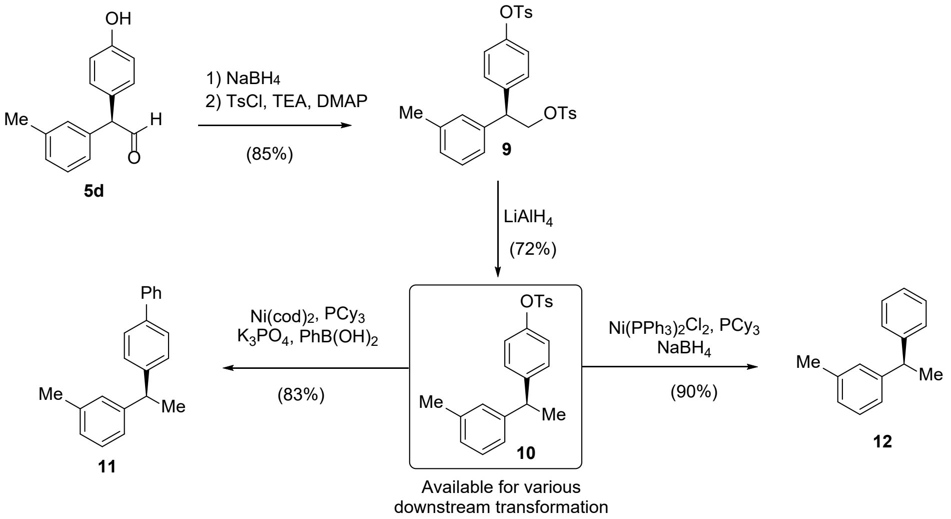

Synthesis of **9**: In-situ addition of $NaBH_4$ (1.5 equiv) in MeOH to the reaction mixture containing aldehyde **5d** gave the according alcohol product (quant.), which was isolated and then dissolved in dichloromethane. The alcohol in DCM was subsequently treated with TsCl

(2.5 equiv), TEA (4.0 equiv), DMAP (0.2 equiv) and stirred for overnight. The reaction mixture was diluted by water and extracted by dichloromethane. After the solvent was removed under vacuum, ditosylated product **9** was purified and obtained by silica gel column chromatography (PE/EA = 4:1) in 85% yield.

Synthesis of **10**: Dissolved **9** in anhydrous ether at -30 ºC, and added $LiAlH_4$ (5.0 equiv) in portion to the solution and the mixture was stirred for overnight. The reaction was quenched by adding saturated NH_4Cl solution and extracted by ether. The solvent of the combined organic phase was removed under rotary evaporator and the hydrolyzed product **10** was obtained by silica gel column chromatography (PE/EA = 6:1) in 72% yield.

Synthesis of **11**: $Ni(cod)_2$ (0.1 eq, 1.4 mg), PCy_3 (0.4 equiv, 5.6 mg), $PhB(OH)_2$ (1.5 equiv, 9 mg), K_3PO_4 (3.0 equiv, 32 mg) was added to **10** in THF (2 ml) solution. Water was added to the mixture and the solution was extracted by ethyl acetate. The solvent of combined organic phase was removed by rotary evaporator and the resulting mixture was subjected to the silica gel column chromatography (PE/EA = 12:1). **11** was obtained in 83% yield.

Synthesis of **12**: $Ni(PPh_3)_2Cl_2$ (0.1 eq, 3 mg), PCy_3 (0.40 equiv, 3 mg) and $NaBH_4$ (5.0 equiv, 10 mg) was added to **10** in DMF (2 ml) solution, and the mixture was stirred un 60 ºC for overnight. Water was added to the mixture and the solution was extracted by ethyl acetate. The solvent of combined organic phase was removed by rotary evaporator and the resulting mixture was subjected to the silica gel column chromatography (PE/EA = 10:1). 12 was obtained in 90% yield.

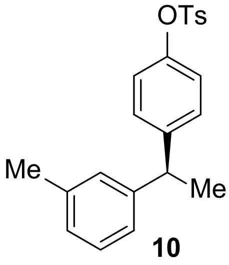

10

(*R*)-4-(1-(m-Tolyl)ethyl)phenyl 4-methylbenzenesulfonate (10)

72% yield, a light-yellow oil, ^{1}H NMR (400 MHz, $CDCl_3$) δ 7.71 (d, *J* = 8.4 Hz, 2H), 7.30 (d, *J* = 7.8 Hz, 2H), 7.20 – 7.07 (m, 3H), 7.06 – 6.92 (m, 3H), 6.92 – 6.84 (m, 2H), 4.07 (q, *J* = 7.2 Hz, 1H), 2.44 (s, 3H), 2.31 (s, 3H), 1.57 (d, *J* = 7.2 Hz, 3H).

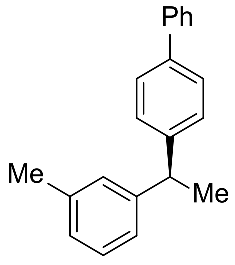

11

(*R*)-4-(1-(m-Tolyl)ethyl)-1,1'-biphenyl (11)

83% yield, a light-yellow oil, ^{1}H NMR (400 MHz, $CDCl_3$) δ 7.62 – 7.55 (m, 2H), 7.55 – 7.49 (m, 2H), 7.47 – 7.39 (m, 2H), 7.37 – 7.28 (m, 3H), 7.24 – 7.17 (m, 1H), 7.08 (d, *J* = 7.4 Hz, 2H), 7.03 (d, *J* = 7.7 Hz, 1H), 4.17 (q, *J* = 7.2 Hz, 1H), 2.34 (s, 3H), 1.68 (d, *J* = 7.2 Hz, 3H).

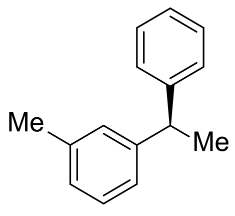

12

(*R*)-1-Methyl-3-(1-phenylethyl)benzene (12)

90% yield, a light-yellow oil, ^{1}H NMR (400 MHz, $CDCl_3$) δ 7.32 – 7.26 (m, 2H), 7.25 – 7.13 (m, 4H), 7.07 – 6.96 (m, 3H), 4.11 (q, *J* = 7.2 Hz, 1H), 2.31 (d, *J* = 0.7 Hz, 3H), 1.63 (d, *J* = 7.2 Hz, 4H). (The ^{1}H-NMR of **12** is the same as the previous study[3]).

(b) *Figure S7.* Synthesis of an anti-smallpox agent.

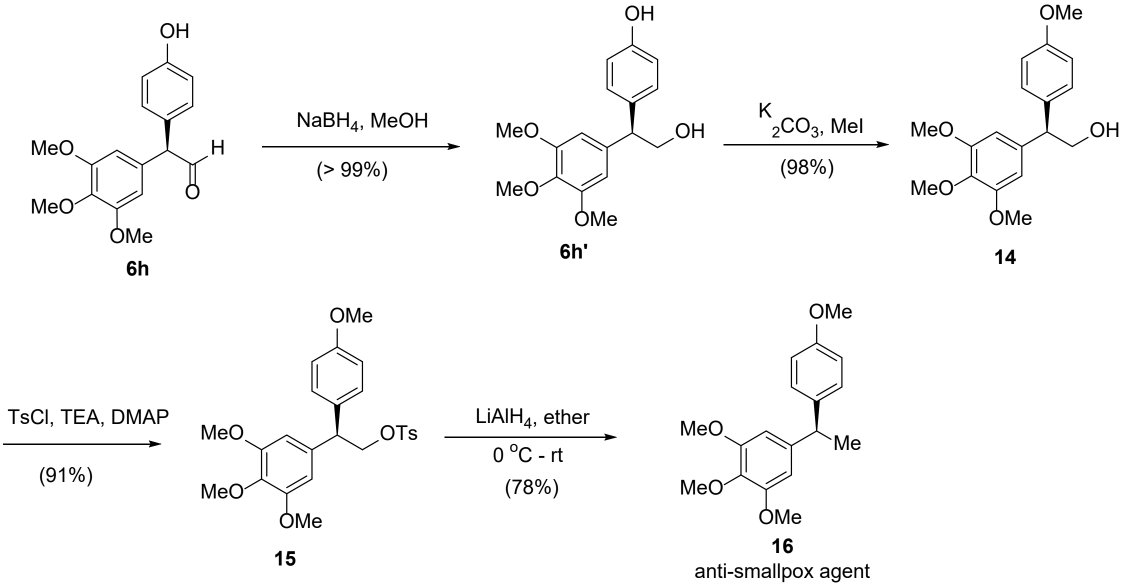

Synthesis of **6h'**: compound **6h'** was isolated with yield of exceed 99% after $NaBH_4$ in (1.5 equiv) in MeOH was added to **6h.**

Synthesis of **14**: To the solution of **6h'** in THF was added K_2CO_3 (2 equiv) and MeI (2.5 equiv), and the reaction was stired at room temperature until the starting compounds were disappeared. Then water was added to the solution and extracted with EA. And the extracts were evaporated, then the residue was purified by silica gel column chromatography (PE/EA = 3:1) to afford **14** in 98% yield.

Synthesis of **15**: To the solution of **14** in anhydrous DCM was added TEA (1.5 equiv), DMAP (0.1 equiv) and TsCl (1.2 equiv) sequentially and was stired at room temperature for overnight. The reaction mixture was diluted by water and extracted by dichloromethane. After the solvent was removed under vacuum, product **15** was purified and obtained by silica gel column chromatography (PE/EA = 5:1) in 91% yield.

Synthesis of **16**: Dissolved **15** in anhydrous ether at 0 °C, and added $LiAlH_4$ (5.0 equiv) in portion to the solution and the mixture was stirred for overnight at room temperature. The reaction was quenched by adding saturated NH_4Cl solution and extracted by ether. The solvent of the combined organic phase was removed under rotary evaporator and the anti-smallpox agent **16** was obtained by silica gel column chromatography (PE/EA = 8:1) in 78% yield.

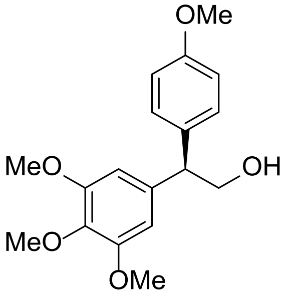

14

(*R*)-2-(4-Methoxyphenyl)-2-(3,4,5-trimethoxyphenyl)ethan-1-ol (14)

98% yield, a light-yellow oil, ^{1}H NMR (400 MHz, $CDCl_3$) δ 7.18 (d, *J* = 8.7 Hz, 2H), 6.87 (d, *J* = 8.6 Hz, 2H), 6.47 (s, 2H), 4.10 (s, 3H), 3.82 (s, 9H), 3.78 (s, 3H). ^{13}C NMR (101 MHz, $CDCl_3$) δ 158.4, 153.3, 139.1, 137.3, 136.8, 133.2, 129.1, 114.1, 105.3, 66.3, 60.8, 56.1, 55.2, 53.0.

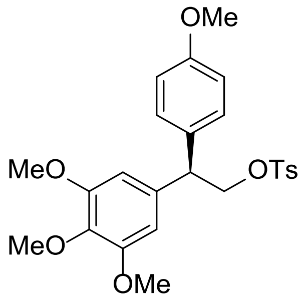

15

(*R*)-2-(4-Methoxyphenyl)-2-(3,4,5-trimethoxyphenyl)ethyl 4-methylbenzenesulfonate (15)

91% yield, a light-yellow oil, ^{1}H NMR (400 MHz, $CDCl_3$) δ 7.63 (d, *J* = 8.3 Hz, 2H), 7.27 (d, *J* = 7.4 Hz, 2H), 7.04 (d, *J* = 8.6 Hz, 2H), 6.80 (d, *J* = 8.7 Hz, 2H), 6.31 (s, 2H), 4.53 – 4.38 (m, 2H), 4.21 (t, *J* = 7.4 Hz, 1H), 3.81 (s, 3H), 3.77 (d, *J* = 7.7 Hz, 9H), 2.43 (s, 3H). ^{13}C NMR (101 MHz, $CDCl_3$) δ 158.6, 153.2, 144.7, 136.9, 135.6, 132.8, 131.6, 129.7, 129.7, 129.0, 128.9, 127.8, 127.8, 114.0, 114.0, 105.2, 72.0, 60.8, 56.0, 55.2, 49.3, 21.6.

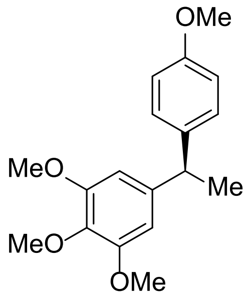

16

(*R*)-1,2,3-Trimethoxy-5-(1-(4-methoxyphenyl)ethyl)benzene (16)

78% yield, a light-yellow oil, ^{1}H NMR (400 MHz, $CDCl_3$) δ 7.14 (d, *J* = 8.6 Hz, 2H), 6.84 (d, *J* = 8.8 Hz, 2H), 6.42 (s, 2H), 4.04 (q, *J* = 7.2 Hz, 1H), 3.81 (d, *J* = 1.7 Hz, 9H), 3.79 (d, *J* = 1.8 Hz, 3H), 1.59 (d, *J* = 7.2 Hz, 3H). ^{13}C NMR (101 MHz, $CDCl_3$) δ 157.9, 153.0, 142.5, 138.3, 136.2, 128.4, 113.7, 104.6, 60.8, 56.1, 55.2, 44.2, 22.2. HRMS (ESI) m/z calcd for $C_{18}H_{23}O_4$ $[M+H]^+$ = 303.1591, found = 303.1596. The NMR spectra of our synthetic sample were consistent with previous study[4a]. $[\alpha]_D^{25}$ = -1.5° (*c* 1.0, $CHCl_3$), {Lit[4b], $[\alpha]_D^{25}$ = –1.8° (*c* 1.0, $CHCl_3$)}.

^{1}H-NMR spectrum (our synthetic sample)

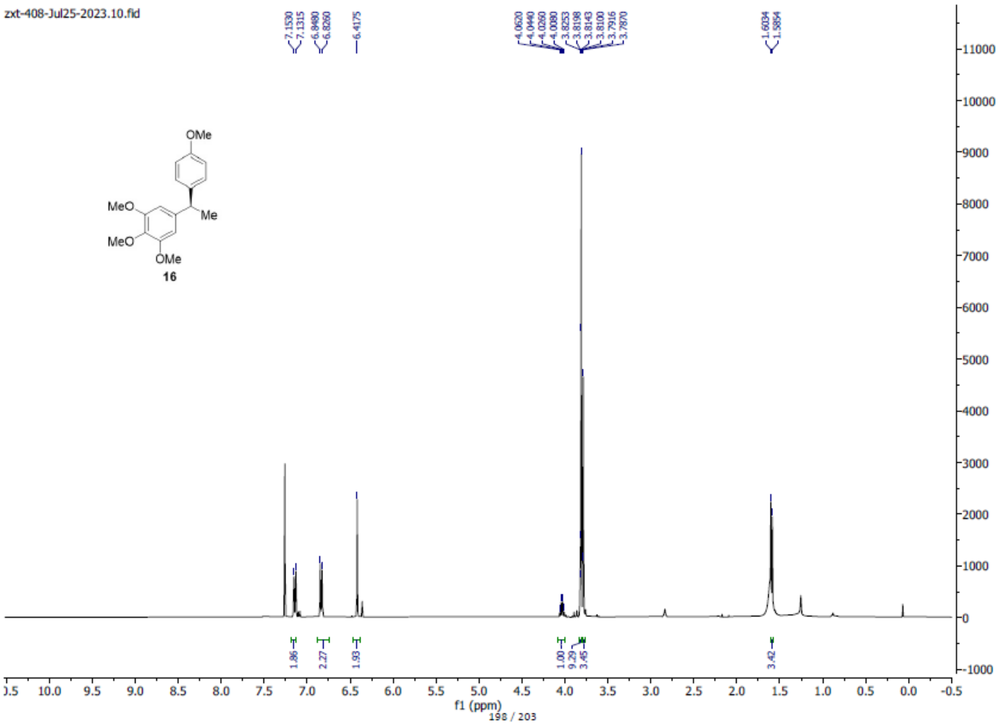

^{1}H-NMR spectrum (reported by Prof. Yin[4a])

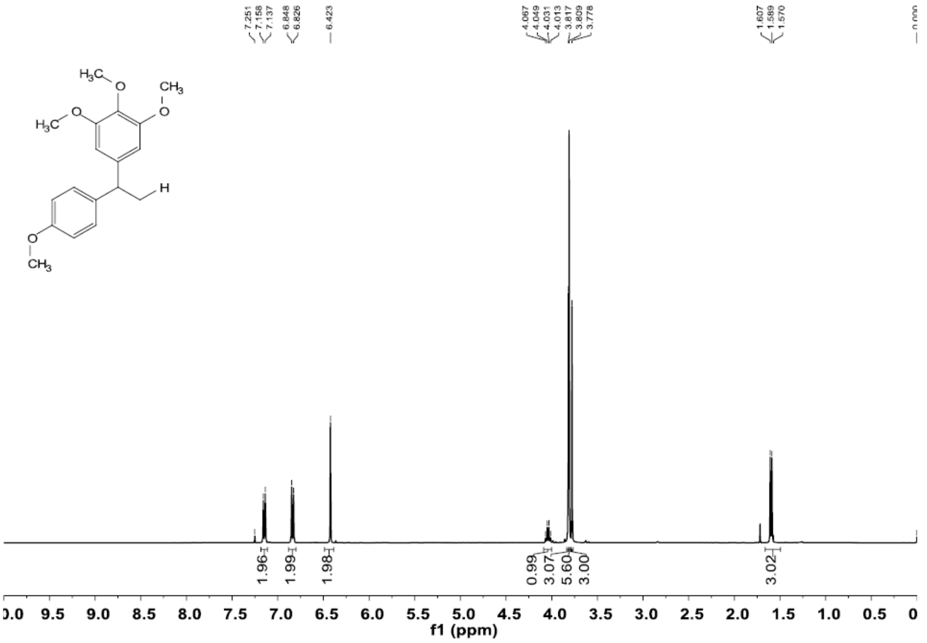

(c) *Figure S8.* Formal synthesis of an antidepressant drug (+)-sertraline.

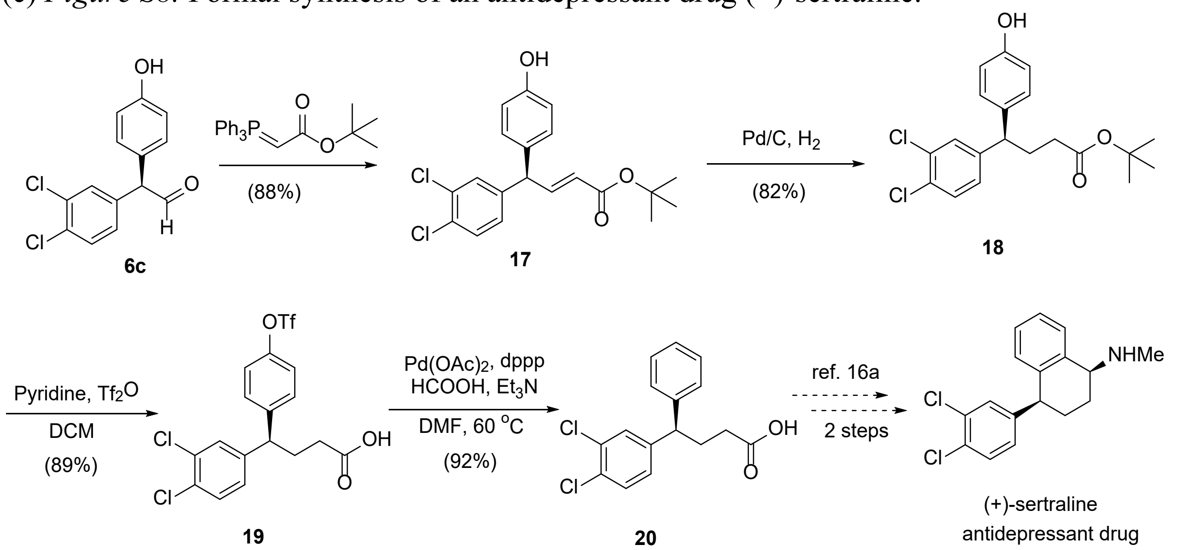

Synthesis of **17**: directly added *tert*-butyl(triphenylphosphoranylidene)acetate (1.5 equiv) to the reaction mixture under -80 °C and the mixture was stirred for 4 hours. After removal of the solvent, the mixture was purified by silica gel column chromatography (PE/EA = 3:1) and 17 was obtained in 88% yield.

Synthesis of **18**: Dissolved **17** in methanol and added Pd/C (0.1 equiv), and the atmosphere in the flash was replaced by hydrogen (using hydrogen balloon). The mixture was stirred for 12 hours, and the mixture was filtered and the solvent was removed by vacuum. 18 was obtained after silica gel column chromatography (PE/EA = 4:1) in 82% yield.

Synthesis of **19**: **18** was dissolved in dichloromethane, and pyridine (1.2 equiv) and Tf_2O (1.2 equiv) was added subsequently under 0 °C. After the reaction was complete, solvent was removed under rotavap and the mixture was subjected to silica gel column chromatography (PE/EA = 1:1) to afford acid **19** in 89% yield.

Synthgsis of **20**: $Pd(OAc)_2$ (0.1 equiv, 1.1 mg), dppp (0.15 equiv, 3 mg), HCOOH (13.5 equiv), TEA (13.0 equiv) was added to **19** in DMF and the mixture was stirred under 60 °C for 12 hours. Water was added to the mixture and the solution was extracted by ethyl acetate. The solvent of combined organic phase was removed by rotary evaporator and the resulting mixture was subjected to the silica gel column chromatography (PE/EA = 1:1). **20** was obtained in 92% yield.

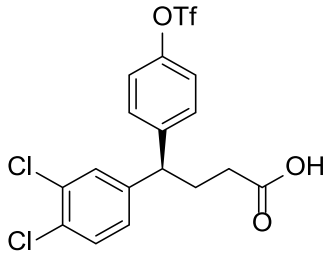

19

(*R*)-4-(3,4-Dichlorophenyl)-4-(4-(((trifluoromethyl)sulfonyl)oxy)phenyl)butanoic acid (19)

89% yield, a light-yellow oil, ^{1}H NMR (400 MHz, $CDCl_3$) δ 7.38 (d, *J* = 8.3 Hz, 1H), 7.33 – 7.13 (m, 5H), 7.04 (dd, *J* = 8.3, 2.1 Hz, 1H), 4.03 – 3.88 (m, 1H), 2.44 – 2.19 (m, 4H). ^{19}F NMR (377 MHz, $CDCl_3$) δ -72.85.

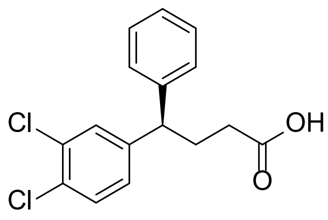

20

(*R*)-4-(3,4-Dichlorophenyl)-4-phenylbutanoic acid (20)

92% yield, a light-yellow oil, ^{1}H NMR (400 MHz, $CDCl_3$) δ 7.38 – 7.26 (m, 4H), 7.25 – 7.14 (m, 3H), 7.07 (dd, *J* = 8.3, 2.2 Hz, 1H), 4.76 (s, 1H), 3.92 (t, *J* = 7.6 Hz, 1H), 2.42 – 2.28 (m, 4H). ^{13}C NMR (101 MHz, $CDCl_3$) δ 177.7, 148.3, 143.3, 143.0, 132.9, 131.1, 130.8, 129.7, 129.5, 127.2, 121.7, 48.8, 31.8, 29.9. The NMR spectra of our synthetic sample were consistent with previous study [5]. $[\alpha]_D^{24}$ = -10.9° (*c* 2.1, benzene), {Lit[5] $[\alpha]_D^{24}$ = –13.0° (*c* 2.2, benzene)}.

^{1}H-NMR spectrum of **20** (our synthetic sample)

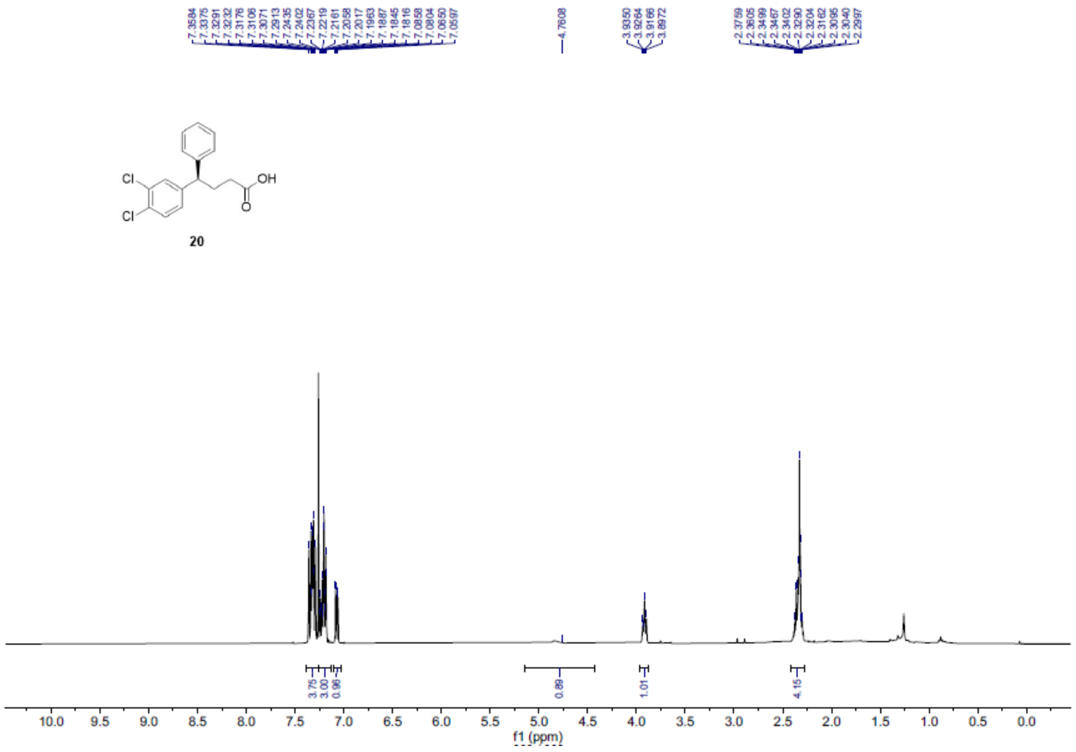

^{1}H-NMR spectrum (reported by Prof. Sawamura[5])

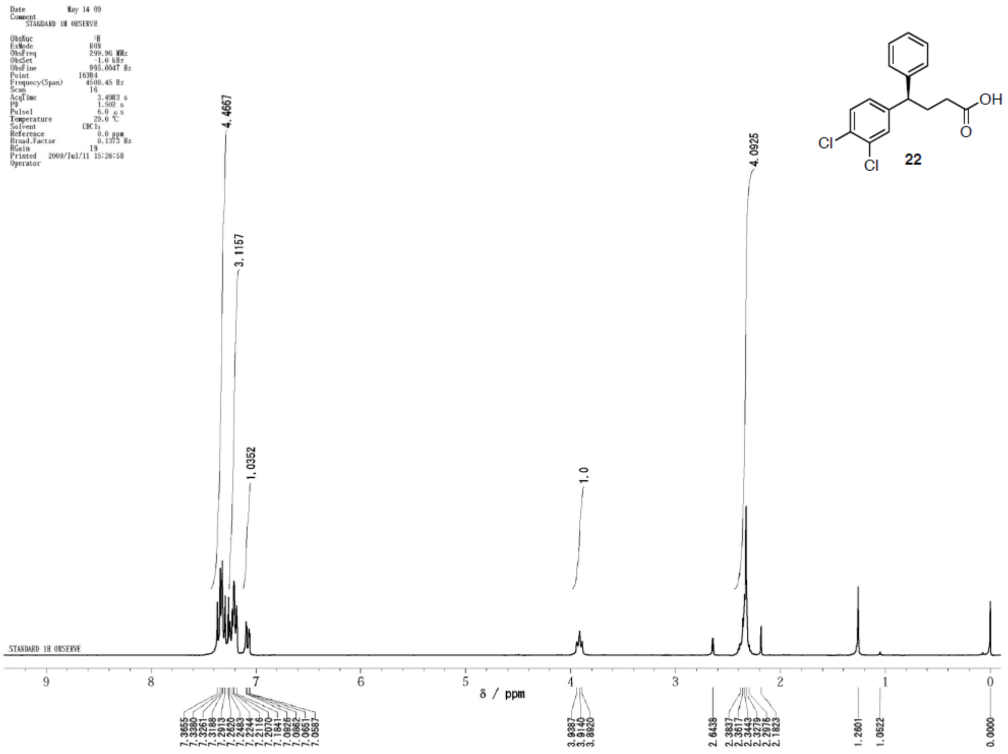

6. Single Crystal Structure X-ray Analysis of 5e' and 5t'

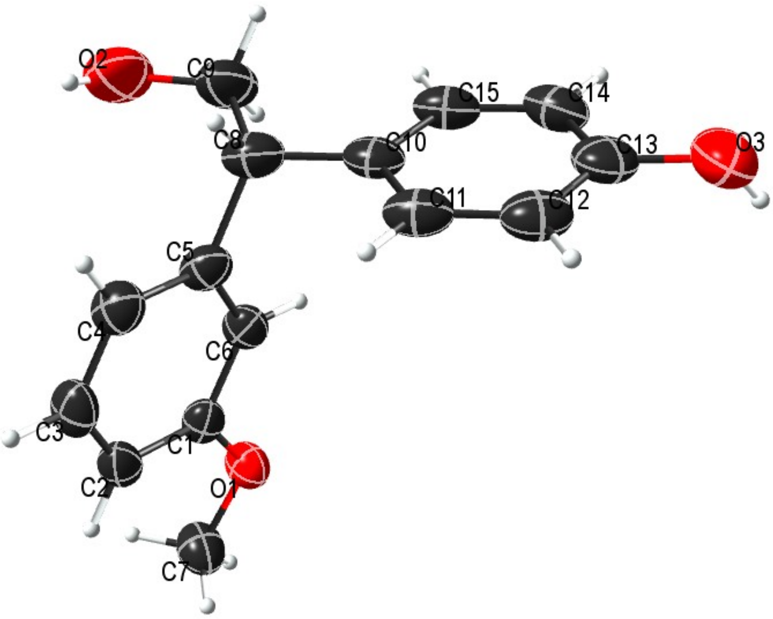

<u>Single Crystal Structure X-ray Analysis of 5e'</u>

Sample Code: N336

CCDC: 2298948

Date: 8-08-2023

Note: The crystal is hexagonal, space group P6(3). The asymmetric unit contains one molecule of the compound C15H16O3 and two quarterly occupied water molecules. H atoms of the water molecules could not be located. Hence, they were not included in the final refinement. Restraints in thermal parameters were applied to the water molecules. H atoms of the OH groups were located form different map and refined with restraints in bond lengths and thermal parameters.

As Flack x = 0.034(538) by classical fit to all intensities and = 0.052(48) from 1142 selected quotients by Parsons' method, the reported structure is the correct hand.

Final R values are R1=0.0561 and wR2=0.1553 for 2- theta up to 144°.

Table S1. Crystal data and structure refinement for N336.

Identification code	N336
Empirical formula	C15 H17 O3.50
Formula weight	253.28
Temperature	100(2) K
Wavelength	1.54178 Å
Crystal system	Hexagonal
Space group	$P6_3$
Unit cell dimensions	a = 19.8013(3) Å a= 90°.
	b = 19.8013(3) Å b= 90°.
	c = 5.80190(10) Å g = 120°.
Volume	1970.10(7) $Å^3$
Z	6
Density (calculated)	1.281 Mg/m^3
Absorption coefficient	0.738 mm^{-1}
F(000)	810
Crystal size	0.234 x 0.127 x 0.088 mm^3
Theta range for data collection	4.466 to 72.292°.
Index ranges	-24<=h<=24, -24<=k<=24, -7<=l<=7
Reflections collected	57481
Independent reflections	2611 [R(int) = 0.0250]
Completeness to theta = 67.679°	99.9 %
Absorption correction	Semi-empirical from equivalents
Max. and min. transmission	0.7536 and 0.6907
Refinement method	Full-matrix least-squares on F^2
Data / restraints / parameters	2611 / 3 / 183
Goodness-of-fit on F^2	1.055
Final R indices [I>2sigma(I)]	R1 = 0.0561, wR2 = 0.1548
R indices (all data)	R1 = 0.0565, wR2 = 0.1553
Absolute structure parameter	0.05(5)
Extinction coefficient	n/a
Largest diff. peak and hole	0.629 and -0.292 e.$Å^{-3}$

Table S2. Atomic coordinates (x 10^4) and equivalent isotropic displacement parameters ($Å^2$x 10^3)
for N336. U(eq) is defined as one third of the trace of the orthogonalized U^{ij} tensor.

	x	y	z	U(eq)
O(1)	7852(1)	8949(1)	7602(4)	42(1)
O(2)	4642(2)	7115(2)	5642(10)	89(1)
O(3)	6666(2)	4625(2)	3993(9)	79(1)
C(1)	7345(2)	8721(2)	5782(6)	36(1)
C(2)	7397(2)	9210(2)	3997(6)	39(1)
C(3)	6852(2)	8903(2)	2219(6)	46(1)
C(4)	6273(2)	8132(2)	2232(7)	47(1)
C(5)	6223(2)	7643(2)	4000(6)	42(1)
C(6)	6759(2)	7940(2)	5778(6)	38(1)
C(7)	8391(2)	9760(2)	7793(8)	52(1)
C(8)	5565(2)	6785(2)	4028(8)	52(1)
C(9)	4982(2)	6638(2)	5941(10)	65(1)
C(10)	5868(2)	6223(2)	4120(7)	44(1)
C(11)	6323(2)	6213(2)	2290(6)	48(1)
C(12)	6597(2)	5695(2)	2228(7)	53(1)
C(13)	6411(2)	5163(2)	3998(8)	56(1)
C(14)	5968(2)	5162(2)	5815(8)	55(1)
C(15)	5699(2)	5681(2)	5886(7)	50(1)
O(1W)	5961(8)	3187(8)	6270(30)	77(3)
O(2W)	3356(8)	6176(8)	2740(30)	77(3)

Table S3. Bond lengths [Å] and angles [°] for N336.

O(1)-C(1)	1.369(4)
O(1)-C(7)	1.420(4)
O(2)-C(9)	1.418(6)
O(2)-H(2A)	0.85(3)
O(3)-C(13)	1.387(6)
O(3)-H(3A)	0.84(3)
C(1)-C(2)	1.385(5)
C(1)-C(6)	1.395(4)
C(2)-C(3)	1.393(5)
C(2)-H(2)	0.9500
C(3)-C(4)	1.377(5)
C(3)-H(3)	0.9500
C(4)-C(5)	1.380(5)
C(4)-H(4)	0.9500
C(5)-C(6)	1.383(5)
C(5)-C(8)	1.539(5)
C(6)-H(6)	0.9500
C(7)-H(7A)	0.9800
C(7)-H(7B)	0.9800
C(7)-H(7C)	0.9800
C(8)-C(10)	1.507(6)
C(8)-C(9)	1.521(6)
C(8)-H(8)	1.0000
C(9)-H(9A)	0.9900
C(9)-H(9B)	0.9900
C(10)-C(15)	1.397(6)
C(10)-C(11)	1.399(5)
C(11)-C(12)	1.381(6)
C(11)-H(11)	0.9500
C(12)-C(13)	1.383(6)
C(12)-H(12)	0.9500
C(13)-C(14)	1.371(6)
C(14)-C(15)	1.377(6)
C(14)-H(14)	0.9500
C(15)-H(15)	0.9500
O(2W)-O(2W)#1	1.72(3)

O(2W)-O(2W)#2	1.72(3)

C(1)-O(1)-C(7)	116.5(3)
C(9)-O(2)-H(2A)	125(7)
C(13)-O(3)-H(3A)	120(5)
O(1)-C(1)-C(2)	124.1(3)
O(1)-C(1)-C(6)	115.7(3)
C(2)-C(1)-C(6)	120.2(3)
C(1)-C(2)-C(3)	118.6(3)
C(1)-C(2)-H(2)	120.7
C(3)-C(2)-H(2)	120.7
C(4)-C(3)-C(2)	120.9(3)
C(4)-C(3)-H(3)	119.6
C(2)-C(3)-H(3)	119.6
C(3)-C(4)-C(5)	120.7(3)
C(3)-C(4)-H(4)	119.7
C(5)-C(4)-H(4)	119.7
C(4)-C(5)-C(6)	119.0(3)
C(4)-C(5)-C(8)	120.4(3)
C(6)-C(5)-C(8)	120.5(3)
C(5)-C(6)-C(1)	120.6(3)
C(5)-C(6)-H(6)	119.7
C(1)-C(6)-H(6)	119.7
O(1)-C(7)-H(7A)	109.5
O(1)-C(7)-H(7B)	109.5
H(7A)-C(7)-H(7B)	109.5
O(1)-C(7)-H(7C)	109.5
H(7A)-C(7)-H(7C)	109.5
H(7B)-C(7)-H(7C)	109.5
C(10)-C(8)-C(9)	112.1(3)
C(10)-C(8)-C(5)	112.7(3)
C(9)-C(8)-C(5)	111.2(3)
C(10)-C(8)-H(8)	106.8
C(9)-C(8)-H(8)	106.8
C(5)-C(8)-H(8)	106.8
O(2)-C(9)-C(8)	110.5(4)
O(2)-C(9)-H(9A)	109.6
C(8)-C(9)-H(9A)	109.6

O(2)-C(9)-H(9B)	109.6
C(8)-C(9)-H(9B)	109.6
H(9A)-C(9)-H(9B)	108.1
C(15)-C(10)-C(11)	117.1(4)
C(15)-C(10)-C(8)	124.2(4)
C(11)-C(10)-C(8)	118.7(3)
C(12)-C(11)-C(10)	121.7(4)
C(12)-C(11)-H(11)	119.2
C(10)-C(11)-H(11)	119.2
C(11)-C(12)-C(13)	119.5(4)
C(11)-C(12)-H(12)	120.2
C(13)-C(12)-H(12)	120.2
C(14)-C(13)-C(12)	119.9(4)
C(14)-C(13)-O(3)	118.6(4)
C(12)-C(13)-O(3)	121.5(4)
C(13)-C(14)-C(15)	120.6(4)
C(13)-C(14)-H(14)	119.7
C(15)-C(14)-H(14)	119.7
C(14)-C(15)-C(10)	121.2(4)
C(14)-C(15)-H(15)	119.4
C(10)-C(15)-H(15)	119.4
O(2W)#1-O(2W)-O(2W)#260.000(6)	

Symmetry transformations used to generate equivalent atoms:

#1 -x+y,-x+1,z #2 -y+1,x-y+1,z

Table S4. Anisotropic displacement parameters ($Å^2 x 10^3$) for N336. The anisotropic displacement factor exponent takes the form: $-2p^2[h^2 a^{*2}U^{11} + ... + 2 h k a^* b^* U^{12}]$

	U^{11}	U^{22}	U^{33}	U^{23}	U^{13}	U^{12}
O(1)	47(1)	39(1)	39(1)	3(1)	-5(1)	20(1)
O(2)	54(2)	72(2)	136(4)	3(3)	25(3)	28(2)
O(3)	63(2)	47(2)	117(3)	-7(2)	-13(2)	20(2)
C(1)	37(2)	43(2)	30(1)	5(1)	6(1)	23(1)
C(2)	40(2)	40(2)	40(2)	8(2)	10(2)	22(1)
C(3)	56(2)	54(2)	34(2)	13(2)	3(2)	32(2)
C(4)	46(2)	56(2)	39(2)	4(2)	-3(2)	25(2)
C(5)	34(2)	50(2)	38(2)	4(2)	3(1)	19(1)
C(6)	39(2)	42(2)	33(2)	9(1)	5(1)	20(1)
C(7)	57(2)	43(2)	54(2)	-6(2)	-14(2)	22(2)
C(8)	38(2)	53(2)	51(2)	3(2)	0(2)	12(2)
C(9)	48(2)	52(2)	83(3)	6(2)	17(2)	17(2)
C(10)	34(2)	40(2)	39(2)	-3(2)	-6(1)	5(1)
C(11)	39(2)	53(2)	33(2)	1(2)	-3(1)	8(2)
C(12)	41(2)	54(2)	45(2)	-12(2)	-2(2)	10(2)
C(13)	42(2)	42(2)	64(2)	-11(2)	-7(2)	7(2)
C(14)	50(2)	41(2)	53(2)	6(2)	-7(2)	6(2)
C(15)	41(2)	49(2)	36(2)	-1(2)	0(2)	5(2)
O(1W)	69(6)	74(5)	78(7)	-14(5)	23(5)	28(4)
O(2W)	69(6)	74(5)	78(7)	-14(5)	23(5)	28(4)

Table S5. Hydrogen coordinates (x 10^4) and isotropic displacement parameters (Å^{2}x 10^3) for N336.

	x	y	z	U(eq)
H(2A)	4600(50)	7310(50)	4360(90)	133
H(3A)	7040(30)	4690(40)	4850(120)	119
H(2)	7796	9742	3986	47
H(3)	6881	9231	982	55
H(4)	5905	7934	1009	56
H(6)	6728	7609	7007	46
H(7A)	8658	9867	9284	78
H(7B)	8112	10051	7679	78
H(7C)	8776	9921	6547	78
H(8)	5277	6688	2537	63
H(9A)	4568	6082	5928	77
H(9B)	5249	6751	7452	77
H(11)	6446	6572	1058	58
H(12)	6912	5704	978	63
H(14)	5847	4799	7037	66
H(15)	5392	5672	7158	59

Table S6. Torsion angles [°] for N336.

C(7)-O(1)-C(1)-C(2)	9.0(4)
C(7)-O(1)-C(1)-C(6)	-172.4(3)
O(1)-C(1)-C(2)-C(3)	178.8(3)
C(6)-C(1)-C(2)-C(3)	0.2(5)
C(1)-C(2)-C(3)-C(4)	0.1(5)
C(2)-C(3)-C(4)-C(5)	-0.4(6)
C(3)-C(4)-C(5)-C(6)	0.5(5)
C(3)-C(4)-C(5)-C(8)	178.8(3)
C(4)-C(5)-C(6)-C(1)	-0.3(5)
C(8)-C(5)-C(6)-C(1)	-178.6(3)
O(1)-C(1)-C(6)-C(5)	-178.8(3)
C(2)-C(1)-C(6)-C(5)	-0.1(5)
C(4)-C(5)-C(8)-C(10)	123.0(4)
C(6)-C(5)-C(8)-C(10)	-58.7(5)
C(4)-C(5)-C(8)-C(9)	-110.1(4)
C(6)-C(5)-C(8)-C(9)	68.1(5)
C(10)-C(8)-C(9)-O(2)	-173.2(4)
C(5)-C(8)-C(9)-O(2)	59.6(5)
C(9)-C(8)-C(10)-C(15)	-6.2(5)
C(5)-C(8)-C(10)-C(15)	120.2(4)
C(9)-C(8)-C(10)-C(11)	171.1(3)
C(5)-C(8)-C(10)-C(11)	-62.6(4)
C(15)-C(10)-C(11)-C(12)	-0.3(5)
C(8)-C(10)-C(11)-C(12)	-177.8(3)
C(10)-C(11)-C(12)-C(13)	0.9(5)
C(11)-C(12)-C(13)-C(14)	-1.1(5)
C(11)-C(12)-C(13)-O(3)	179.2(3)
C(12)-C(13)-C(14)-C(15)	0.7(6)
O(3)-C(13)-C(14)-C(15)	-179.6(3)
C(13)-C(14)-C(15)-C(10)	-0.1(6)
C(11)-C(10)-C(15)-C(14)	-0.1(5)
C(8)-C(10)-C(15)-C(14)	177.2(3)

Symmetry transformations used to generate equivalent atoms:

#1 -x+y,-x+1,z #2 -y+1,x-y+1,z

Table S7. Hydrogen bonds for N336 [Å and °].

D-H...A	d(D-H)	d(H...A)	d(D...A)	<(DHA)
O(2)-H(2A)...O(1W^a)#3	0.85(3)	2.08(7)	2.740(15)	133(8)
O(2)-H(2A)...O(2W^b)#2	0.85(3)	1.72(7)	2.385(15)	133(8)
O(3)-H(3A)...O(1W^a)#4	0.84(3)	1.72(6)	2.368(14)	132(7)
O(3)-H(3A)...O(2W^b)#5	0.84(3)	2.24(7)	2.677(14)	112(6)

Symmetry transformations used to generate equivalent atoms:
#1 -x+y,-x+1,z #2 -y+1,x-y+1,z #3 -x+1,-y+1,z-1/2
#4 -x+y+1,-x+1,z #5 -x+1,-y+1,z+1/2

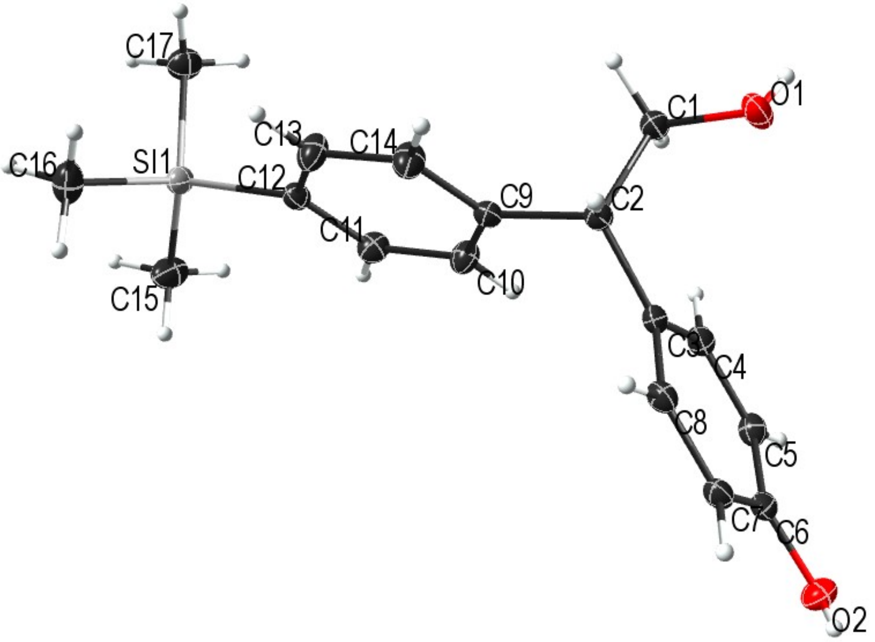

Single Crystal Structure X-ray Analysis of 5t'

Sample Code: N337

CCDC: 2298949

Date: 8-08-2023

Note: The crystal is monoclinic, space group P2(1). The asymmetric unit contains one molecule of the compound C17H22O2Si.

H atoms of the OH groups were located form different map.

As Flack x = 0.010(22) by classical fit to all intensities and = 0.008(6) from 1366 selected quotients by Parsons' method, the reported structure is the correct hand.

Final R values are R1=0.0222 and wR2=0.0629 for 2- theta up to 144º.

Table S8. Crystal data and structure refinement for N337.

Identification code	N337	
Empirical formula	C17 H22 O2 Si	
Formula weight	286.43	
Temperature	100(2) K	
Wavelength	1.54178 Å	
Crystal system	Monoclinic	
Space group	$P2_1$	
Unit cell dimensions	a = 6.1419(6) Å	a= 90°.
	b = 6.7997(7) Å	b= 94.680(3)°.
	c = 18.9762(18) Å	g = 90°.
Volume	789.86(13) $Å^3$	
Z	2	
Density (calculated)	1.204 Mg/m^3	
Absorption coefficient	1.297 mm^{-1}	
F(000)	308	
Crystal size	0.163 x 0.124 x 0.072 mm^3	
Theta range for data collection	4.676 to 72.223°.	
Index ranges	-7<=h<=7, -8<=k<=8, -23<=l<=23	
Reflections collected	23781	
Independent reflections	3082 [R(int) = 0.0224]	
Completeness to theta = 67.679°	99.9 %	
Absorption correction	Semi-empirical from equivalents	
Max. and min. transmission	0.7536 and 0.6795	
Refinement method	Full-matrix least-squares on F^2	
Data / restraints / parameters	3082 / 1 / 192	
Goodness-of-fit on F^2	1.063	
Final R indices [I>2sigma(I)]	R1 = 0.0222, wR2 = 0.0628	
R indices (all data)	R1 = 0.0223, wR2 = 0.0629	
Absolute structure parameter	0.008(6)	
Extinction coefficient	n/a	
Largest diff. peak and hole	0.187 and -0.154 e.$Å^{-3}$	

Table S9. Atomic coordinates (x 10^4) and equivalent isotropic displacement parameters ($Å^2$x 10^3)
for N337. U(eq) is defined as one third of the trace of the orthogonalized U^{ij} tensor.

	x	y	z	U(eq)
Si(1)	7509(1)	9018(1)	8847(1)	17(1)
O(1)	2413(2)	-632(2)	6734(1)	25(1)
O(2)	1849(2)	4079(2)	3860(1)	22(1)
C(1)	3705(3)	926(2)	7056(1)	18(1)
C(2)	2622(2)	2888(2)	6842(1)	15(1)
C(3)	2490(3)	3262(2)	6049(1)	15(1)
C(4)	4166(3)	2767(2)	5628(1)	17(1)
C(5)	3945(3)	3064(2)	4899(1)	18(1)
C(6)	2016(2)	3853(2)	4581(1)	17(1)
C(7)	343(2)	4384(2)	4991(1)	17(1)
C(8)	598(2)	4085(3)	5719(1)	17(1)
C(9)	3777(2)	4488(2)	7295(1)	16(1)
C(10)	5686(3)	5393(3)	7112(1)	20(1)
C(11)	6781(3)	6754(3)	7565(1)	20(1)
C(12)	6015(3)	7262(2)	8214(1)	16(1)
C(13)	4073(3)	6368(3)	8381(1)	24(1)
C(14)	2979(3)	5011(3)	7934(1)	22(1)
C(15)	9316(3)	10647(3)	8364(1)	28(1)
C(16)	5510(3)	10542(3)	9298(1)	27(1)
C(17)	9144(3)	7530(3)	9530(1)	25(1)

Table S10. Bond lengths [Å] and angles [°] for N337.

Si(1)-C(15)	1.8612(19)
Si(1)-C(16)	1.8673(19)
Si(1)-C(17)	1.8707(19)
Si(1)-C(12)	1.8784(17)
O(1)-C(1)	1.431(2)
O(1)-H(1)	0.75(3)
O(2)-C(6)	1.3729(18)
O(2)-H(2)	0.79(3)
C(1)-C(2)	1.531(2)
C(1)-H(1A)	0.9900
C(1)-H(1B)	0.9900
C(2)-C(3)	1.522(2)
C(2)-C(9)	1.525(2)
C(2)-H(2A)	1.0000
C(3)-C(8)	1.392(2)
C(3)-C(4)	1.395(2)
C(4)-C(5)	1.393(2)
C(4)-H(4)	0.9500
C(5)-C(6)	1.392(2)
C(5)-H(5)	0.9500
C(6)-C(7)	1.386(2)
C(7)-C(8)	1.392(2)
C(7)-H(7)	0.9500
C(8)-H(8)	0.9500
C(9)-C(14)	1.391(2)
C(9)-C(10)	1.393(2)
C(10)-C(11)	1.398(2)
C(10)-H(10)	0.9500
C(11)-C(12)	1.397(2)
C(11)-H(11)	0.9500
C(12)-C(13)	1.398(2)
C(13)-C(14)	1.390(2)
C(13)-H(13)	0.9500
C(14)-H(14)	0.9500
C(15)-H(15A)	0.9800
C(15)-H(15B)	0.9800

C(15)-H(15C)	0.9800
C(16)-H(16A)	0.9800
C(16)-H(16B)	0.9800
C(16)-H(16C)	0.9800
C(17)-H(17A)	0.9800
C(17)-H(17B)	0.9800
C(17)-H(17C)	0.9800
C(15)-Si(1)-C(16)	109.63(10)
C(15)-Si(1)-C(17)	110.79(9)
C(16)-Si(1)-C(17)	108.56(9)
C(15)-Si(1)-C(12)	110.09(8)
C(16)-Si(1)-C(12)	109.91(8)
C(17)-Si(1)-C(12)	107.82(8)
C(1)-O(1)-H(1)	106(2)
C(6)-O(2)-H(2)	107.0(17)
O(1)-C(1)-C(2)	108.56(13)
O(1)-C(1)-H(1A)	110.0
C(2)-C(1)-H(1A)	110.0
O(1)-C(1)-H(1B)	110.0
C(2)-C(1)-H(1B)	110.0
H(1A)-C(1)-H(1B)	108.4
C(3)-C(2)-C(9)	115.00(13)
C(3)-C(2)-C(1)	113.23(13)
C(9)-C(2)-C(1)	107.49(12)
C(3)-C(2)-H(2A)	106.9
C(9)-C(2)-H(2A)	106.9
C(1)-C(2)-H(2A)	106.9
C(8)-C(3)-C(4)	117.84(14)
C(8)-C(3)-C(2)	119.17(13)
C(4)-C(3)-C(2)	122.97(14)
C(5)-C(4)-C(3)	121.15(15)
C(5)-C(4)-H(4)	119.4
C(3)-C(4)-H(4)	119.4
C(6)-C(5)-C(4)	119.76(14)
C(6)-C(5)-H(5)	120.1
C(4)-C(5)-H(5)	120.1
O(2)-C(6)-C(7)	122.10(14)

O(2)-C(6)-C(5)	117.85(14)
C(7)-C(6)-C(5)	120.05(14)
C(6)-C(7)-C(8)	119.34(14)
C(6)-C(7)-H(7)	120.3
C(8)-C(7)-H(7)	120.3
C(7)-C(8)-C(3)	121.83(14)
C(7)-C(8)-H(8)	119.1
C(3)-C(8)-H(8)	119.1
C(14)-C(9)-C(10)	117.91(14)
C(14)-C(9)-C(2)	119.50(14)
C(10)-C(9)-C(2)	122.51(14)
C(9)-C(10)-C(11)	120.77(15)
C(9)-C(10)-H(10)	119.6
C(11)-C(10)-H(10)	119.6
C(12)-C(11)-C(10)	121.77(15)
C(12)-C(11)-H(11)	119.1
C(10)-C(11)-H(11)	119.1
C(11)-C(12)-C(13)	116.57(15)
C(11)-C(12)-Si(1)	122.58(12)
C(13)-C(12)-Si(1)	120.84(12)
C(14)-C(13)-C(12)	121.93(15)
C(14)-C(13)-H(13)	119.0
C(12)-C(13)-H(13)	119.0
C(13)-C(14)-C(9)	121.03(15)
C(13)-C(14)-H(14)	119.5
C(9)-C(14)-H(14)	119.5
Si(1)-C(15)-H(15A)	109.5
Si(1)-C(15)-H(15B)	109.5
H(15A)-C(15)-H(15B)	109.5
Si(1)-C(15)-H(15C)	109.5
H(15A)-C(15)-H(15C)	109.5
H(15B)-C(15)-H(15C)	109.5
Si(1)-C(16)-H(16A)	109.5
Si(1)-C(16)-H(16B)	109.5
H(16A)-C(16)-H(16B)	109.5
Si(1)-C(16)-H(16C)	109.5
H(16A)-C(16)-H(16C)	109.5
H(16B)-C(16)-H(16C)	109.5

Si(1)-C(17)-H(17A)	109.5
Si(1)-C(17)-H(17B)	109.5
H(17A)-C(17)-H(17B)	109.5
Si(1)-C(17)-H(17C)	109.5
H(17A)-C(17)-H(17C)	109.5
H(17B)-C(17)-H(17C)	109.5

Symmetry transformations used to generate equivalent atoms:

Table S11. Anisotropic displacement parameters ($Å^2$x 10^3) for N337. The anisotropic displacement factor exponent takes the form: $-2p^2[h^2 a^{*2}U^{11} + ... + 2 h k a^* b^* U^{12}]$

	U^{11}	U^{22}	U^{33}	U^{23}	U^{13}	U^{12}
Si(1)	18(1)	17(1)	16(1)	0(1)	0(1)	-2(1)
O(1)	31(1)	12(1)	29(1)	0(1)	-9(1)	1(1)
O(2)	23(1)	27(1)	15(1)	2(1)	1(1)	-1(1)
C(1)	22(1)	16(1)	17(1)	0(1)	-2(1)	-1(1)
C(2)	16(1)	14(1)	16(1)	0(1)	1(1)	0(1)
C(3)	19(1)	12(1)	16(1)	-1(1)	0(1)	-2(1)
C(4)	16(1)	15(1)	20(1)	-1(1)	-1(1)	0(1)
C(5)	17(1)	17(1)	19(1)	-3(1)	4(1)	-2(1)
C(6)	22(1)	13(1)	15(1)	0(1)	1(1)	-3(1)
C(7)	18(1)	13(1)	20(1)	0(1)	-1(1)	2(1)
C(8)	18(1)	14(1)	19(1)	0(1)	3(1)	1(1)
C(9)	18(1)	14(1)	16(1)	1(1)	0(1)	1(1)
C(10)	20(1)	24(1)	17(1)	-3(1)	5(1)	-1(1)
C(11)	17(1)	23(1)	20(1)	1(1)	4(1)	-3(1)
C(12)	18(1)	14(1)	18(1)	1(1)	0(1)	-1(1)
C(13)	26(1)	26(1)	19(1)	-6(1)	8(1)	-7(1)
C(14)	24(1)	22(1)	21(1)	-2(1)	7(1)	-9(1)
C(15)	30(1)	28(1)	25(1)	3(1)	-1(1)	-12(1)
C(16)	24(1)	28(1)	28(1)	-9(1)	-1(1)	3(1)
C(17)	24(1)	27(1)	23(1)	2(1)	-1(1)	-1(1)

Table S12. Hydrogen coordinates (x 10^4) and isotropic displacement parameters ($Å^2$ x 10^3) for N337.

	x	y	z	U(eq)
H(1)	3100(40)	-1550(40)	6772(13)	34(7)
H(2)	610(40)	4260(50)	3743(12)	35(6)
H(1A)	5200	878	6897	22
H(1B)	3809	782	7577	22
H(2A)	1086	2822	6980	19
H(4)	5480	2219	5842	20
H(5)	5106	2729	4620	21
H(7)	-965	4945	4777	20
H(8)	-551	4454	5998	20
H(10)	6251	5082	6674	24
H(11)	8081	7349	7428	24
H(13)	3484	6698	8814	28
H(14)	1665	4432	8066	27
H(15A)	10142	11512	8702	42
H(15B)	8424	11446	8021	42
H(15C)	10332	9842	8115	42
H(16A)	6291	11337	9668	40
H(16B)	4466	9679	9511	40
H(16C)	4724	11410	8952	40
H(17A)	10102	8403	9827	37
H(17B)	10033	6571	9296	37
H(17C)	8156	6838	9825	37

Table S13. Torsion angles [°] for N337.

O(1)-C(1)-C(2)-C(3)	-62.12(17)
O(1)-C(1)-C(2)-C(9)	169.75(13)
C(9)-C(2)-C(3)-C(8)	-96.79(18)
C(1)-C(2)-C(3)-C(8)	139.09(16)
C(9)-C(2)-C(3)-C(4)	84.98(19)
C(1)-C(2)-C(3)-C(4)	-39.1(2)
C(8)-C(3)-C(4)-C(5)	-0.8(2)
C(2)-C(3)-C(4)-C(5)	177.46(15)
C(3)-C(4)-C(5)-C(6)	-0.5(2)
C(4)-C(5)-C(6)-O(2)	-178.56(14)
C(4)-C(5)-C(6)-C(7)	1.5(2)
O(2)-C(6)-C(7)-C(8)	178.89(15)
C(5)-C(6)-C(7)-C(8)	-1.2(2)
C(6)-C(7)-C(8)-C(3)	-0.2(3)
C(4)-C(3)-C(8)-C(7)	1.1(3)
C(2)-C(3)-C(8)-C(7)	-177.19(15)
C(3)-C(2)-C(9)-C(14)	141.50(15)
C(1)-C(2)-C(9)-C(14)	-91.41(17)
C(3)-C(2)-C(9)-C(10)	-41.9(2)
C(1)-C(2)-C(9)-C(10)	85.22(18)
C(14)-C(9)-C(10)-C(11)	1.2(2)
C(2)-C(9)-C(10)-C(11)	-175.45(15)
C(9)-C(10)-C(11)-C(12)	-0.1(3)
C(10)-C(11)-C(12)-C(13)	-1.2(3)
C(10)-C(11)-C(12)-Si(1)	177.76(13)
C(15)-Si(1)-C(12)-C(11)	22.96(17)
C(16)-Si(1)-C(12)-C(11)	143.83(14)
C(17)-Si(1)-C(12)-C(11)	-98.00(15)
C(15)-Si(1)-C(12)-C(13)	-158.15(14)
C(16)-Si(1)-C(12)-C(13)	-37.28(17)
C(17)-Si(1)-C(12)-C(13)	80.88(15)
C(11)-C(12)-C(13)-C(14)	1.3(3)
Si(1)-C(12)-C(13)-C(14)	-177.65(14)
C(12)-C(13)-C(14)-C(9)	-0.2(3)
C(10)-C(9)-C(14)-C(13)	-1.1(3)
C(2)-C(9)-C(14)-C(13)	175.67(16)

Symmetry transformations used to generate equivalent atoms:

Table S14. Hydrogen bonds for N337 [Å and °].

D-H...A	d(D-H)	d(H...A)	d(D...A)	<(DHA)
O(2)-H(2)...O(1)#1	0.79(3)	2.00(3)	2.7709(18)	168(3)

Symmetry transformations used to generate equivalent atoms:

#1 -x,y+1/2,-z+1

7. References

[1] a) F. A. Cruz, V. M. Dong, *J. Am. Chem. Soc.* **2017**, *139*, 1029-1032; b) Y. Ping, K. Wang, Q. Pan, Z. Ding, Z. Zhou, Y. Guo, W. Kong, *ACS Catal.* **2019**, *9*, 7335-7342; c) R. T. Davison, P. D. Parker, X. Hou, C. P. Chung, S. A. Augustine, V. M. Dong, *Angew. Chem., Int. Ed.* **2021**, *60*, 4599-4603; d) Y. e. You, S. Ge, *Angew. Chem., Int. Ed.* **2021**, *60*, 20684-20688; e) J. Li, S. Ge, *Angew. Chem., Int. Ed.* **2022**, *61*, e202213057.

[2] a) T. M. Bräuer, Q. Zhang, K. Tiefenbacher, *J. Am. Chem. Soc.* **2017**, *139*, 17500-17507; b) C.-H. Fan, T. Xu, Z. Ke, Y.-Y. Yeung, *Org. Chem. Front.* **2022**, *9*, 4091-4096.

[3] X. Cheng, H. Lu, Z. Lu, *Nat. Commun.* **2019**, *10*, 3549.

[4] a) L. Peng, Y. Li, Y. Li, W. Wang, H. Pang, G. Yin, *ACS Catal.* **2018**, *8*, 310-313; b) Y. Li, K. Dong, Z. Wang, K. Ding, *Angew. Chem., Int. Ed.* **2013**, *52,* 6748-6752.

[5] H. Ohmiya, Y. Makida, D. Li, M. Tanabe, M. Sawamura, *J. Am. Chem. Soc.* **2010**, *132*, 879-889.

SHIMADZU LabSolutions

Analysis Report

<Sample Information>

Sample Name	:		
Sample ID	:		
Data Filename	: s3-17-8-rac-IE-20%-1ml.lcd		
Method Filename	: WMY-20%-220-60mins.lcm		
Batch Filename	: s3-17-8-rac.lcb		
Vial #	: 1-1	Sample Type	: Unknown
Injection Volume	: 10 uL		
Date Acquired	: 11/4/2023 3:58:38 PM	Acquired by	: System Administrator
Date Processed	: 11/4/2023 4:22:06 PM	Processed by	: System Administrator

<Chromatogram>

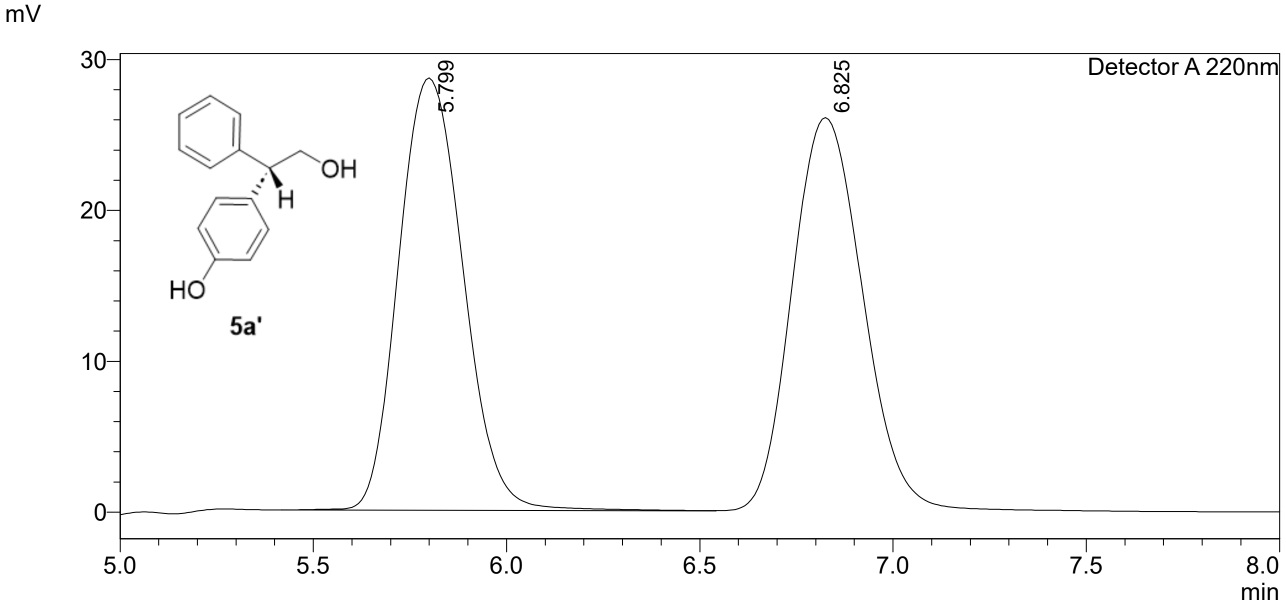

<Peak Table>

Detector A 220nm

Peak#	Ret. Time	Area	Height	Conc.	Unit	Mark	Name
1	5.799	332808	28654	49.934			
2	6.825	333682	26093	50.066		SV	
Total		666490	54747				

SHIMADZU LabSolutions

Analysis Report

<Sample Information>

Sample Name	:		
Sample ID	:		
Data Filename	: s3-17-7-asy-IE-20%-1ml001.lcd		
Method Filename	: WMY-20%-220-12mins.lcm		
Batch Filename	: 12.lcb		
Vial #	: 1-2	Sample Type	: Unknown
Injection Volume	: 10 uL		
Date Acquired	: 11/4/2023 5:35:13 PM	Acquired by	: System Administrator
Date Processed	: 11/4/2023 8:19:04 PM	Processed by	: System Administrator

<Chromatogram>

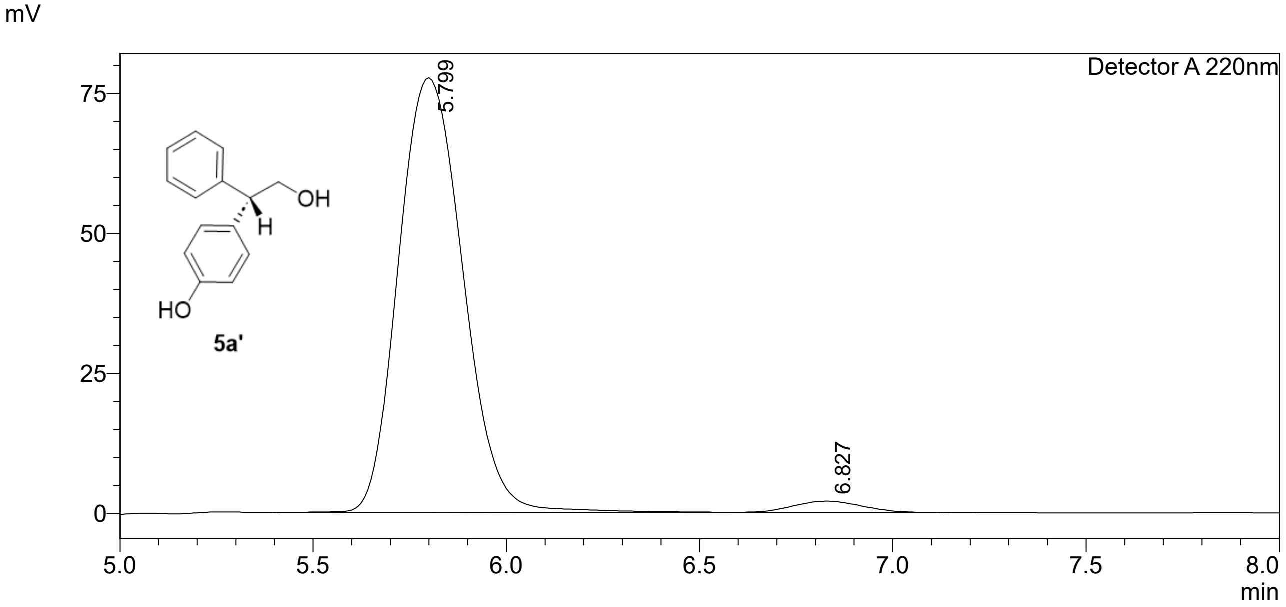

<Peak Table>

Detector A 220nm

Peak#	Ret. Time	Area	Height	Conc.	Unit	Mark	Name
1	5.799	902238	77594	97.437			
2	6.827	23729	1968	2.563		M	
Total		925967	79562				

SHIMADZU LabSolutions

Analysis Report

<Sample Information>

Sample Name	:		
Sample ID	:		
Data Filename	: S3-18-5-RAC-ie-20%-1ML.lcd		
Method Filename	: 20%-1ml-220nm-20min.lcm		
Batch Filename	: S3-18-4-6.lcb		
Vial #	: 1-16	Sample Type	: Unknown
Injection Volume	: 10 uL		
Date Acquired	: 4/13/2023 4:47:47 PM	Acquired by	: System Administrator
Date Processed	: 4/14/2023 9:22:07 AM	Processed by	: System Administrator

<Chromatogram>

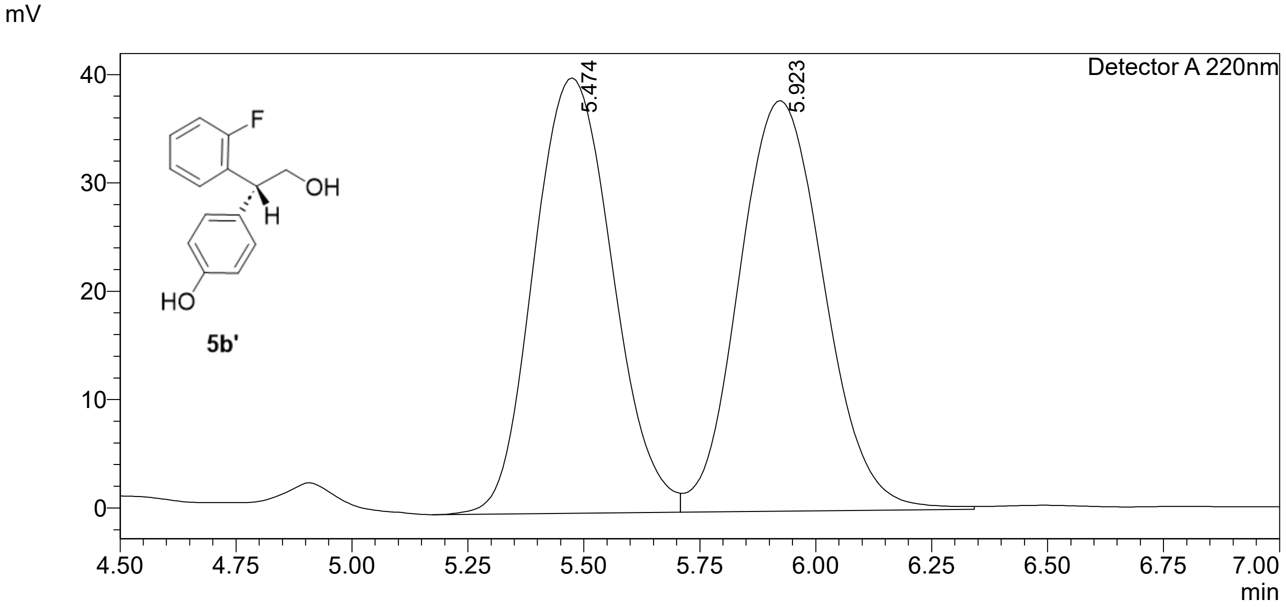

<Peak Table>

Detector A 220nm

Peak#	Ret. Time	Area	Height	Conc.	Unit	Mark	Name
1	5.474	475345	40176	49.520			
2	5.923	484560	37888	50.480		V	
Total		959904	78064				

SHIMADZU LabSolutions

Analysis Report

<Sample Information>

Sample Name	:		
Sample ID	:		
Data Filename	: S3-18-5-ASY-ie-20%-1ML.lcd		
Method Filename	: 20%-1ml-220nm-20min.lcm		
Batch Filename	: S3-18-4-6.lcb		
Vial #	: 1-17	Sample Type	: Unknown
Injection Volume	: 10 uL		
Date Acquired	: 4/13/2023 5:08:12 PM	Acquired by	: System Administrator
Date Processed	: 4/14/2023 9:22:46 AM	Processed by	: System Administrator

<Chromatogram>

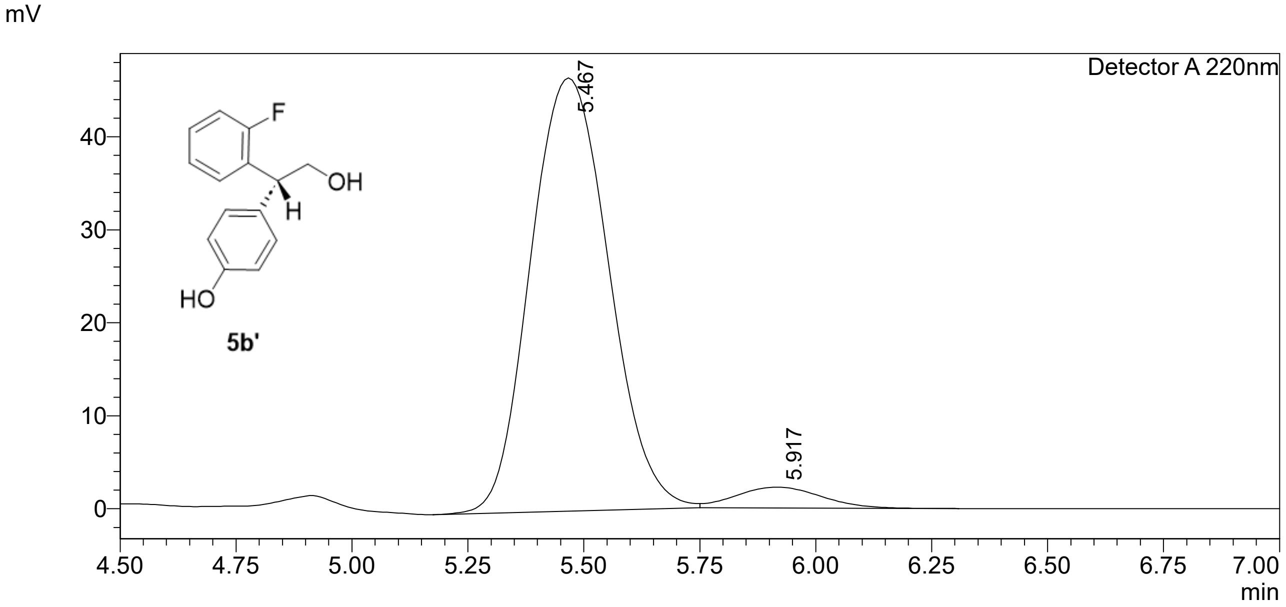

<Peak Table>

Detector A 220nm

Peak#	Ret. Time	Area	Height	Conc.	Unit	Mark	Name
1	5.467	547351	46567	95.056		M	
2	5.917	28469	2229	4.944		M	
Total		575820	48796				

SHIMADZU LabSolutions

Analysis Report

<Sample Information>

Sample Name	:		
Sample ID	:		
Data Filename	: 366-1-RAC-IE-10%.lcd		
Method Filename	: 10%-0.8ml-220nm-45min.lcm		
Batch Filename	: 366-1-RAC-IE-10%.lcd.lcb		
Vial #	: 1-1	Sample Type	: Unknown
Injection Volume	: 10 uL		
Date Acquired	: 4/17/2023 2:00:20 PM	Acquired by	: System Administrator
Date Processed	: 4/17/2023 3:26:43 PM	Processed by	: System Administrator

<Chromatogram>

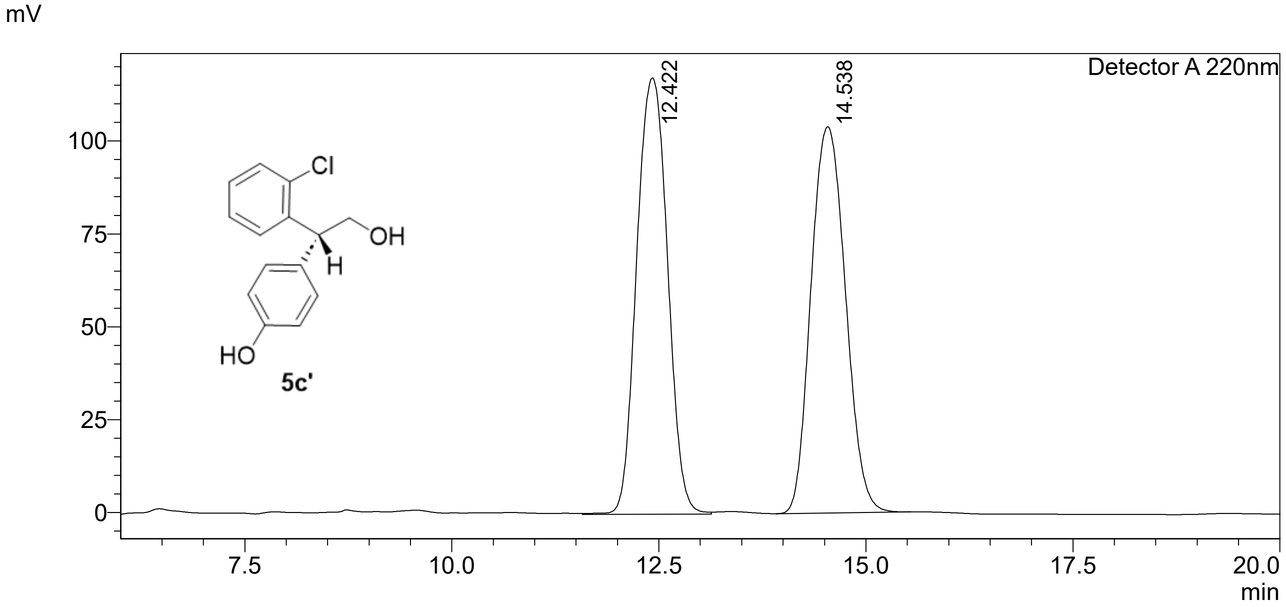

<Peak Table>

Detector A 220nm

Peak#	Ret. Time	Area	Height	Conc.	Unit	Mark	Name
1	12.422	3053415	117447	50.294			
2	14.538	3017658	103988	49.706			
Total		6071074	221435				

SHIMADZU LabSolutions

Analysis Report

<Sample Information>

Sample Name	:		
Sample ID	:		
Data Filename	: 366-1-CHIRAL-IE-10%.lcd		
Method Filename	: 10%-0.8ml-220nm-30min.lcm		
Batch Filename	: 366-1-CHIRAL-IE-10%.lcd.lcb		
Vial #	: 1-2	Sample Type	: Unknown
Injection Volume	: 10 uL		
Date Acquired	: 4/17/2023 2:45:45 PM	Acquired by	: System Administrator
Date Processed	: 4/17/2023 3:27:00 PM	Processed by	: System Administrator

<Chromatogram>

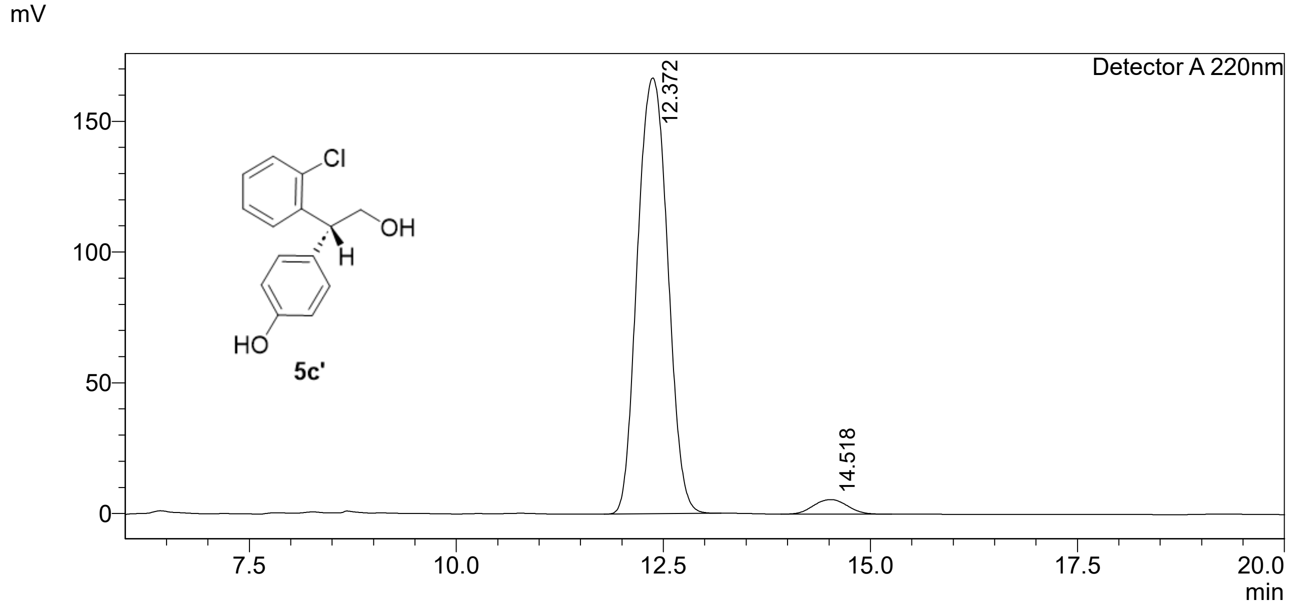

<Peak Table>

Detector A 220nm

Peak#	Ret. Time	Area	Height	Conc.	Unit	Mark	Name
1	12.372	4254321	166650	96.420			
2	14.518	157982	5557	3.580			
Total		4412304	172208				

SHIMADZU LabSolutions

Analysis Report

<Sample Information>

Sample Name	:		
Sample ID	:		
Data Filename	: 366-3-RAC-IE-10%.lcd		
Method Filename	: 10%-0.8ml-220nm-30min.lcm		
Batch Filename	: 366-3-RAC-IE-10%.lcd.lcb		
Vial #	: 1-2	Sample Type	: Unknown
Injection Volume	: 10 uL		
Date Acquired	: 4/17/2023 8:47:43 PM	Acquired by	: System Administrator
Date Processed	: 4/18/2023 10:00:15 AM	Processed by	: System Administrator

<Chromatogram>

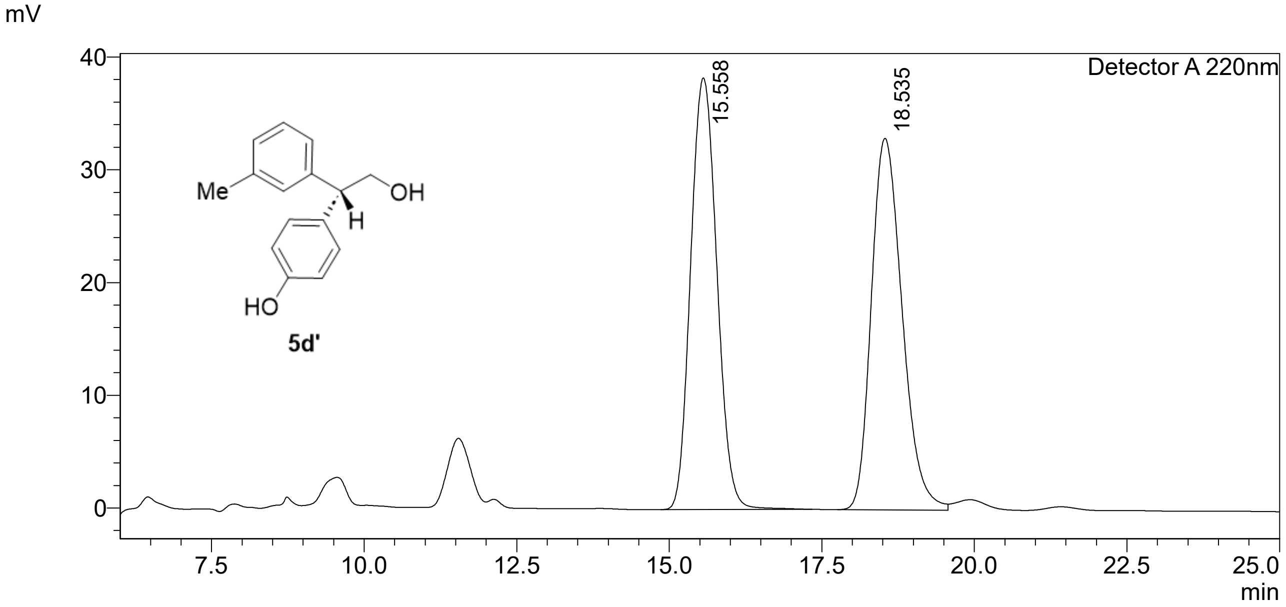

<Peak Table>

Detector A 220nm

Peak#	Ret. Time	Area	Height	Conc.	Unit	Mark	Name
1	15.558	1144074	38278	49.703		S	
2	18.535	1157755	32954	50.297			
Total		2301830	71231				

SHIMADZU LabSolutions

Analysis Report

<Sample Information>

Sample Name	:		
Sample ID	:		
Data Filename	: 366-3-CHIRAL-IE-10%.lcd		
Method Filename	: 10%-0.8ml-220nm-30min.lcm		
Batch Filename	: 366-3&366-6.lcb		
Vial #	: 1-3	Sample Type	: Unknown
Injection Volume	: 10 uL		
Date Acquired	: 4/17/2023 9:18:10 PM	Acquired by	: System Administrator
Date Processed	: 5/11/2023 3:32:24 PM	Processed by	: System Administrator

<Chromatogram>

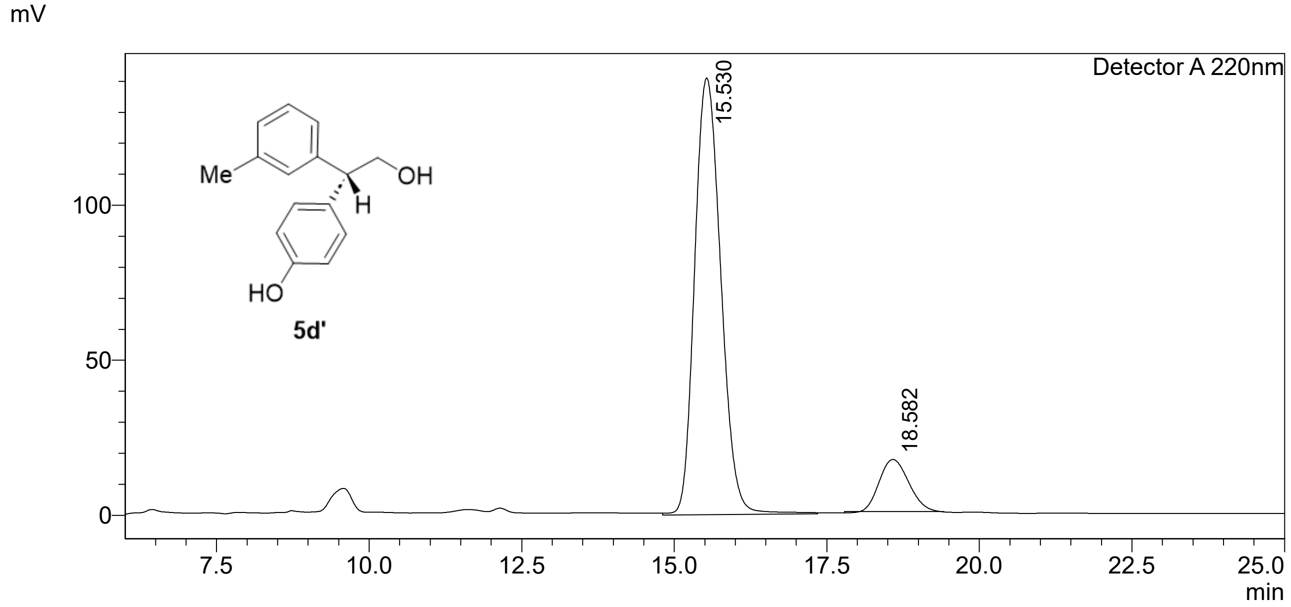

<Peak Table>

Detector A 220nm

Peak#	Ret. Time	Area	Height	Conc.	Unit	Mark	Name
1	15.530	4215030	140883	88.243		M	
2	18.582	561600	16830	11.757		M	
Total		4776629	157713				

SHIMADZU LabSolutions

Analysis Report

<Sample Information>

Sample Name	:		
Sample ID	:		
Data Filename	: 373-6-rac-ie-20%.lcd		
Method Filename	: WMY-20%-220-40mins.lcm		
Batch Filename	: 373-6.lcb		
Vial #	: 1-3	Sample Type	: Unknown
Injection Volume	: 10 uL		
Date Acquired	: 6/5/2023 2:01:26 PM	Acquired by	: System Administrator
Date Processed	: 6/5/2023 3:06:25 PM	Processed by	: System Administrator

<Chromatogram>

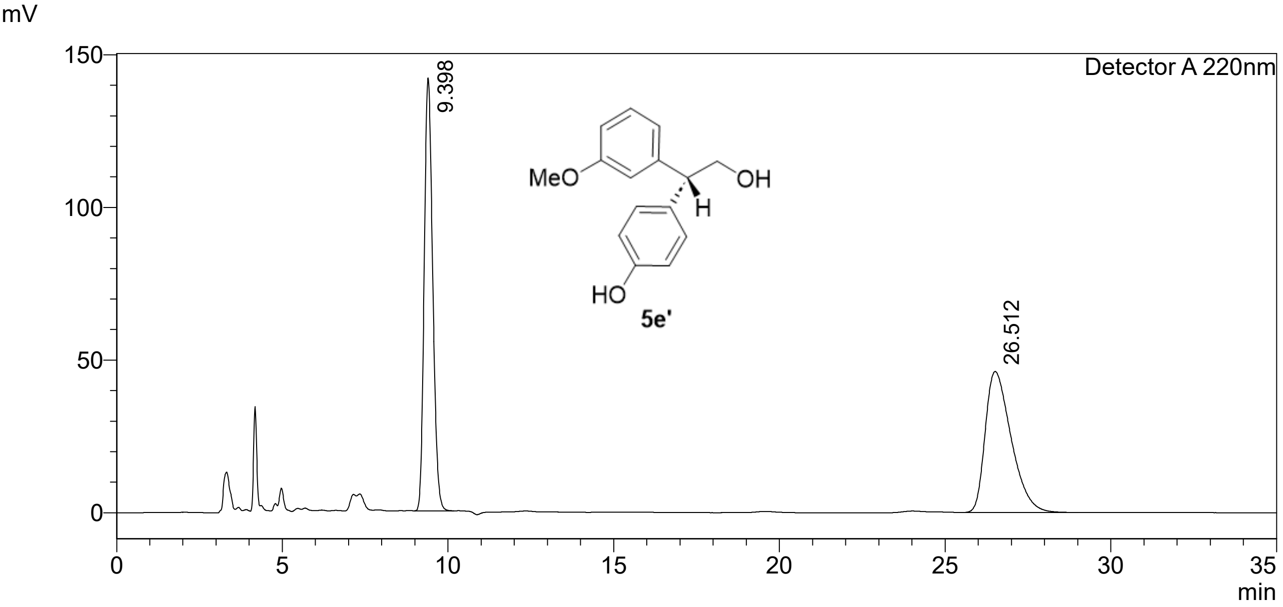

<Peak Table>

Detector A 220nm

Peak#	Ret. Time	Area	Height	Conc.	Unit	Mark	Name
1	9.398	2525365	141773	50.057			
2	26.512	2519629	46180	49.943			
Total		5044994	187954				

SHIMADZU LabSolutions

Analysis Report

<Sample Information>

Sample Name	:		
Sample ID	:		
Data Filename	: 373-6-chiral-ie-20%.lcd		
Method Filename	: WMY-20%-220-40mins.lcm		
Batch Filename	: 373-6.lcb		
Vial #	: 1-4	Sample Type	: Unknown
Injection Volume	: 10 uL		
Date Acquired	: 6/5/2023 2:41:50 PM	Acquired by	: System Administrator
Date Processed	: 6/5/2023 3:24:43 PM	Processed by	: System Administrator

<Chromatogram>

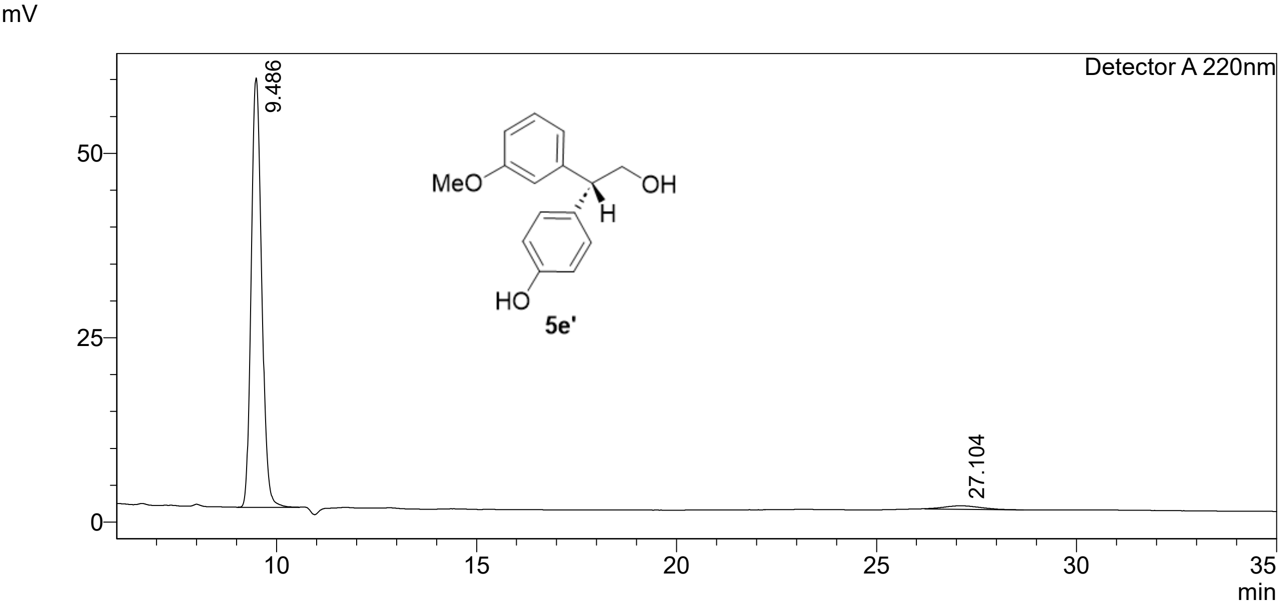

<Peak Table>

Detector A 220nm

Peak#	Ret. Time	Area	Height	Conc.	Unit	Mark	Name
1	9.486	1057603	58202	97.200			
2	27.104	30464	474	2.800		V	
Total		1088067	58676				

SHIMADZU LabSolutions

Analysis Report

<Sample Information>

Sample Name	:		
Sample ID	:		
Data Filename	: S3-21-2-RAC-ie-20%-1ML.lcd		
Method Filename	: 20%-1ml-220nm-40min.lcm		
Batch Filename	: S3-22-1-RAC-ie-20%-1ML.lcb		
Vial #	: 1-1	Sample Type	: Unknown
Injection Volume	: 10 uL		
Date Acquired	: 5/3/2023 6:59:56 PM	Acquired by	: System Administrator
Date Processed	: 5/3/2023 8:08:15 PM	Processed by	: System Administrator

<Chromatogram>

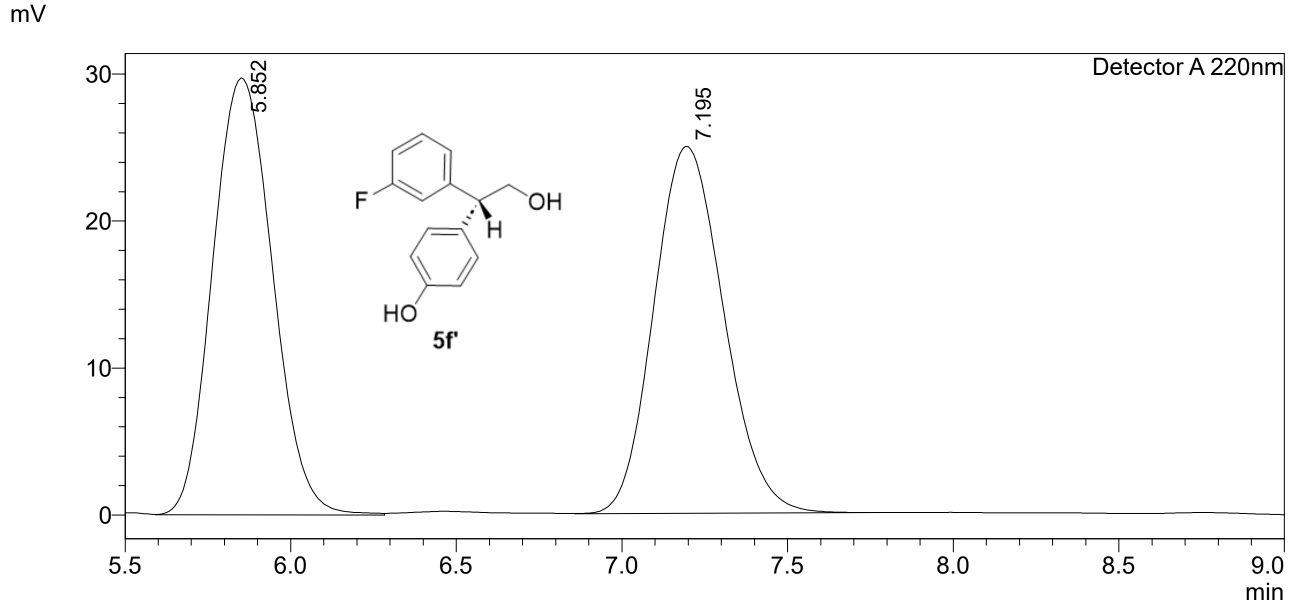

<Peak Table>

Detector A 220nm

Peak#	Ret. Time	Area	Height	Conc.	Unit	Mark	Name
1	5.852	374790	29724	50.531		M	
2	7.195	366916	24963	49.469		M	
Total		741706	54687				

SHIMADZU LabSolutions

Analysis Report

<Sample Information>

Sample Name	:		
Sample ID	:		
Data Filename	: S3-21-2-ASY-ie-20%-1ML.lcd		
Method Filename	: 20%-1ml-220nm-10min.lcm		
Batch Filename	: S3-22.lcb		
Vial #	: 1-1	Sample Type	: Unknown
Injection Volume	: 10 uL		
Date Acquired	: 5/3/2023 8:11:23 PM	Acquired by	: System Administrator
Date Processed	: 5/3/2023 8:22:05 PM	Processed by	: System Administrator

<Chromatogram>

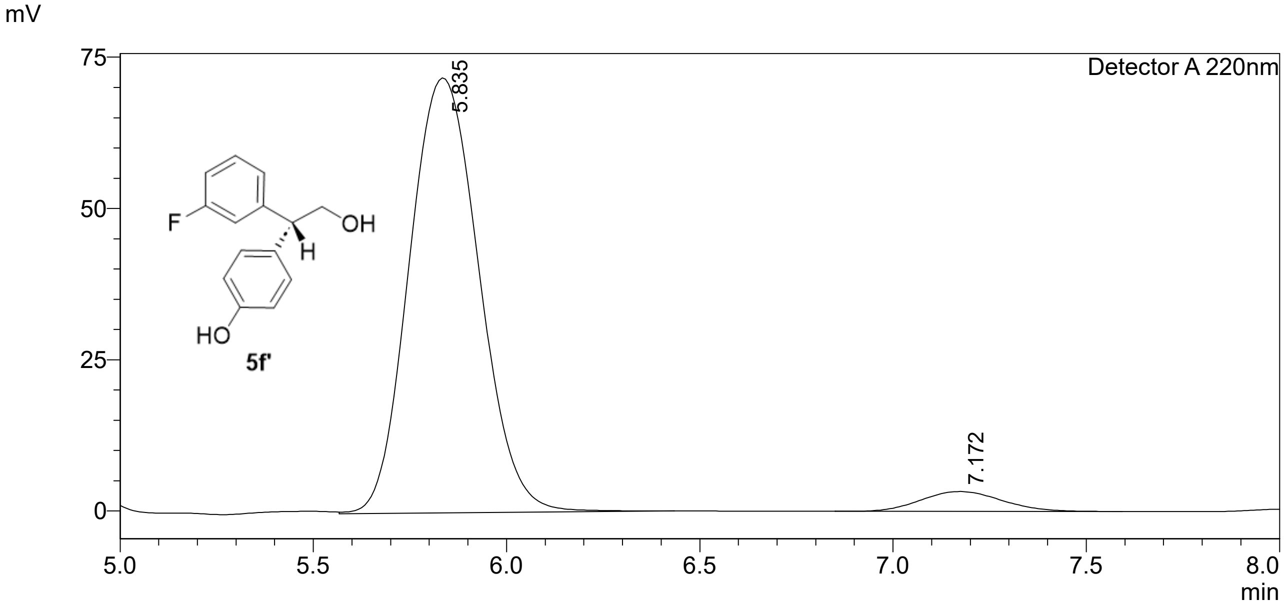

<Peak Table>

Detector A 220nm

Peak#	Ret. Time	Area	Height	Conc.	Unit	Mark	Name
1	5.835	903726	71886	94.961			
2	7.172	47952	3284	5.039			
Total		951678	75170				

SHIMADZU LabSolutions

Analysis Report

<Sample Information>

Sample Name	:		
Sample ID	:		
Data Filename	: S3-18-3-RAC-ie-20%-1ML.lcd		
Method Filename	: 20%-1ml-220nm-20min.lcm		
Batch Filename	: S3-18-3-RAC-ie-20%-1ML.lcb		
Vial #	: 1-1	Sample Type	: Unknown
Injection Volume	: 10 uL		
Date Acquired	: 4/13/2023 3:26:09 PM	Acquired by	: System Administrator
Date Processed	: 4/14/2023 9:20:45 AM	Processed by	: System Administrator

<Chromatogram>

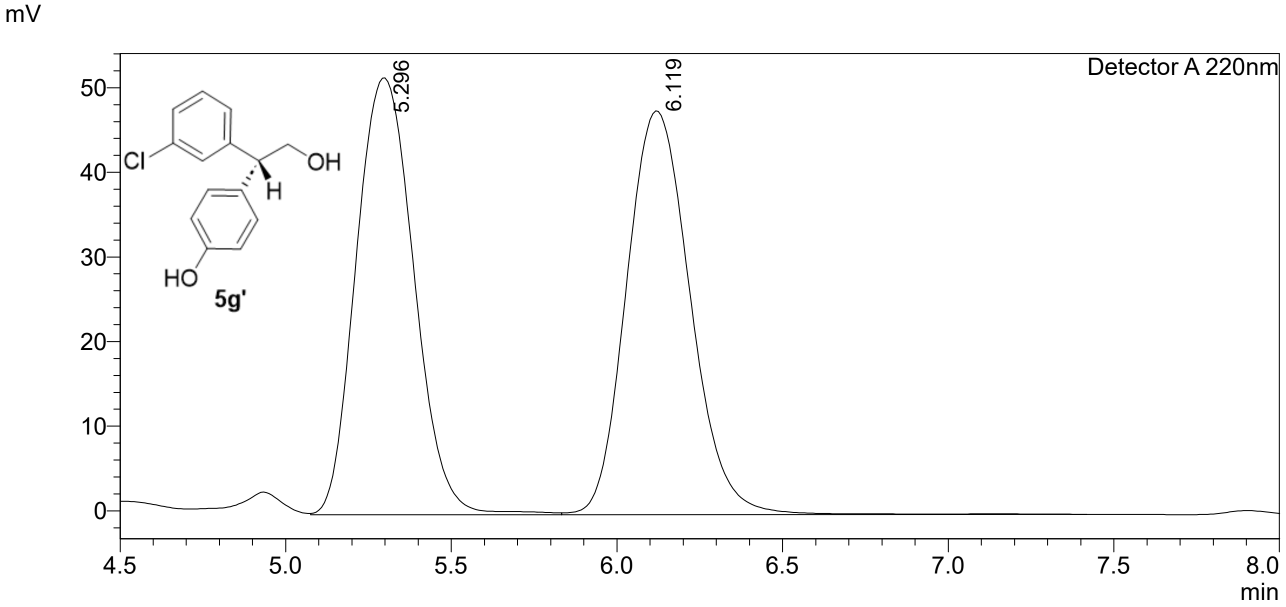

<Peak Table>

Detector A 220nm

Peak#	Ret. Time	Area	Height	Conc.	Unit	Mark	Name
1	5.296	628229	51623	49.580			
2	6.119	638878	47699	50.420		SV	
Total		1267108	99322				

SHIMADZU LabSolutions

Analysis Report

<Sample Information>

Sample Name	:		
Sample ID	:		
Data Filename	: S3-18-3-ASY-ie-20%-1ML.lcd		
Method Filename	: 20%-1ml-220nm-20min.lcm		
Batch Filename	: S3-18-3-ASY-ie-20%-1ML.lcb		
Vial #	: 1-2	Sample Type	: Unknown
Injection Volume	: 10 uL		
Date Acquired	: 4/13/2023 3:46:34 PM	Acquired by	: System Administrator
Date Processed	: 4/14/2023 9:20:32 AM	Processed by	: System Administrator

<Chromatogram>

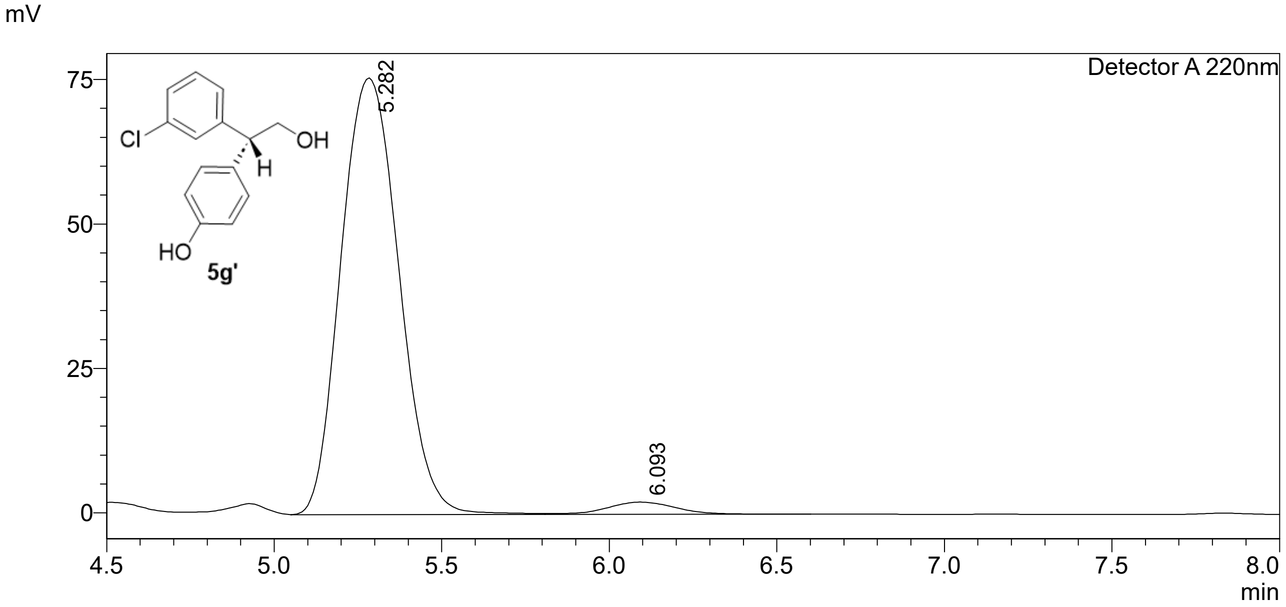

<Peak Table>

Detector A 220nm

Peak#	Ret. Time	Area	Height	Conc.	Unit	Mark	Name
1	5.282	908988	75558	96.763			
2	6.093	30412	2121	3.237		V	
Total		939400	77679				

SHIMADZU LabSolutions

Analysis Report

<Sample Information>

Sample Name	:		
Sample ID	:		
Data Filename	: s3-21-3-rac-IE-10-%-1ml.lcd		
Method Filename	: 10%1ml-220nm-30min.lcm		
Batch Filename	: s3-21-3-rac-IE-10-%-1ml.lcb		
Vial #	: 1-1	Sample Type	: Unknown
Injection Volume	: 10 uL		
Date Acquired	: 5/10/2023 1:46:16 PM	Acquired by	: System Administrator
Date Processed	: 5/10/2023 2:36:59 PM	Processed by	: System Administrator

<Chromatogram>

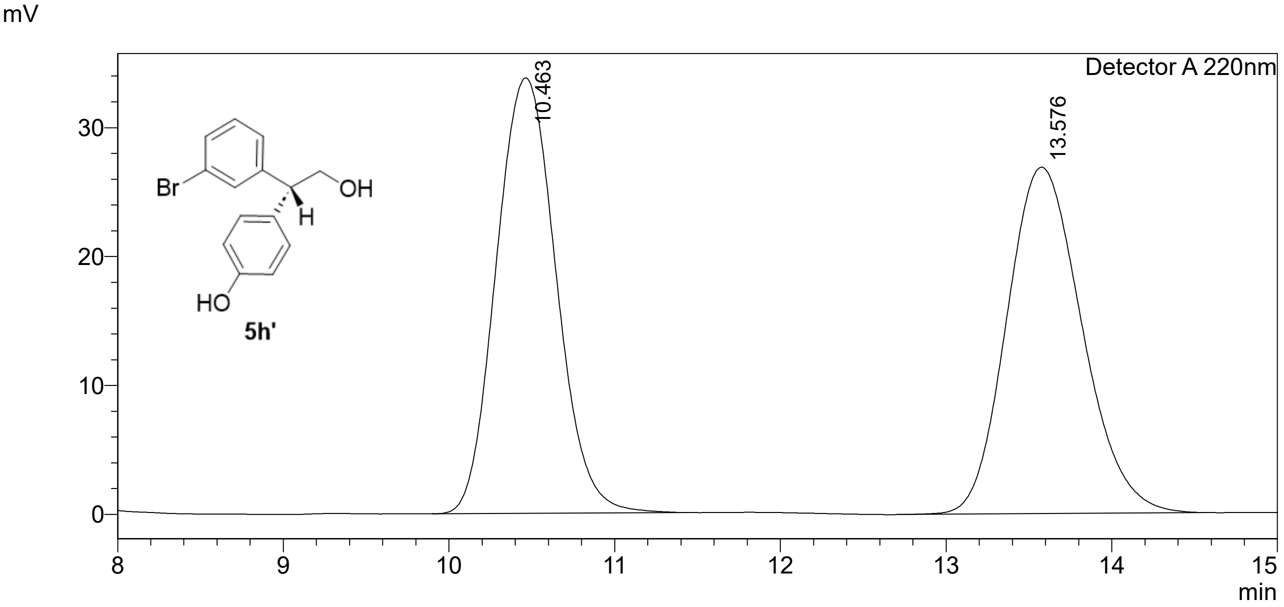

<Peak Table>

Detector A 220nm

Peak#	Ret. Time	Area	Height	Conc.	Unit	Mark	Name
1	10.463	849324	33778	50.050			
2	13.576	847627	26883	49.950			
Total		1696951	60660				

SHIMADZU LabSolutions

Analysis Report

<Sample Information>

Sample Name	:		
Sample ID	:		
Data Filename	: s3-23-3-asy-IE-10%-1ml.lcd		
Method Filename	: 10%1ml-220nm-20min.lcm		
Batch Filename	: s3-23-3-asy-IE-10%-1ml.lcb		
Vial #	: 1-2	Sample Type	: Unknown
Injection Volume	: 10 uL		
Date Acquired	: 5/14/2023 3:48:55 PM	Acquired by	: System Administrator
Date Processed	: 5/14/2023 4:12:12 PM	Processed by	: System Administrator

<Chromatogram>

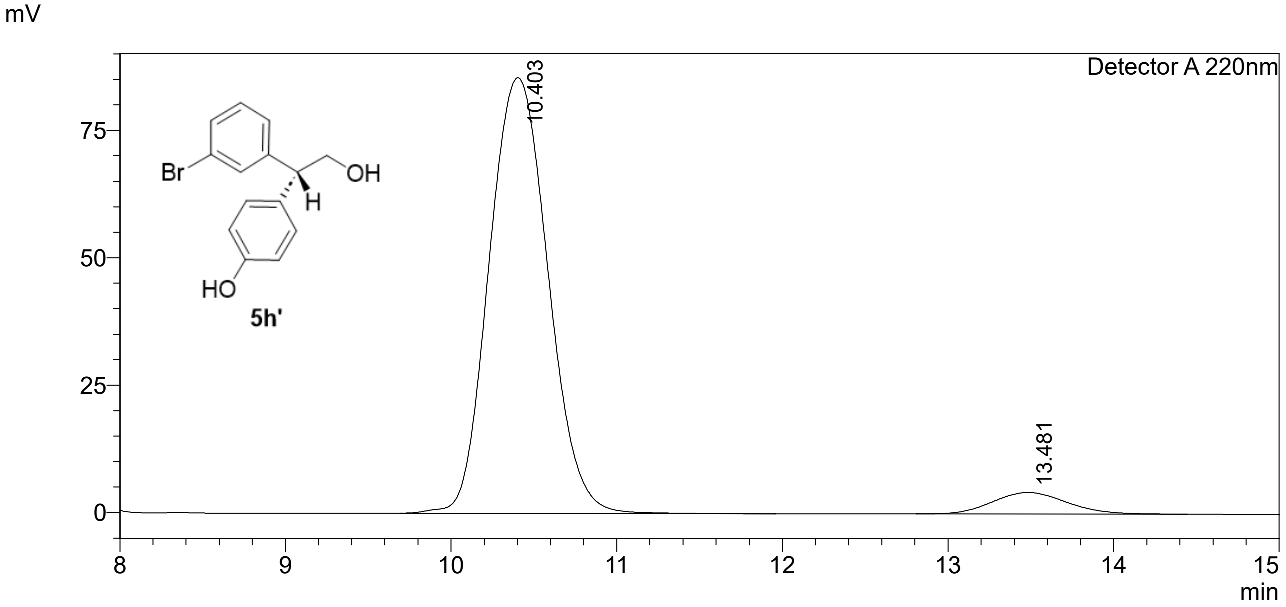

<Peak Table>

Detector A 220nm

Peak#	Ret. Time	Area	Height	Conc.	Unit	Mark	Name
1	10.403	2113578	85483	94.129			
2	13.481	131833	4210	5.871			
Total		2245411	89693				

SHIMADZU LabSolutions

Analysis Report

<Sample Information>

Sample Name	:		
Sample ID	:		
Data Filename	: 368-2-RAC-IE-20%.lcd		
Method Filename	: 20%1ml-220nm-60min.lcm		
Batch Filename	: 368-2-RAC-IE-20%.lcd.lcb		
Vial #	: 1-3	Sample Type	: Unknown
Injection Volume	: 10 uL		
Date Acquired	: 4/21/2023 2:40:45 PM	Acquired by	: System Administrator
Date Processed	: 4/21/2023 3:00:32 PM	Processed by	: System Administrator

<Chromatogram>

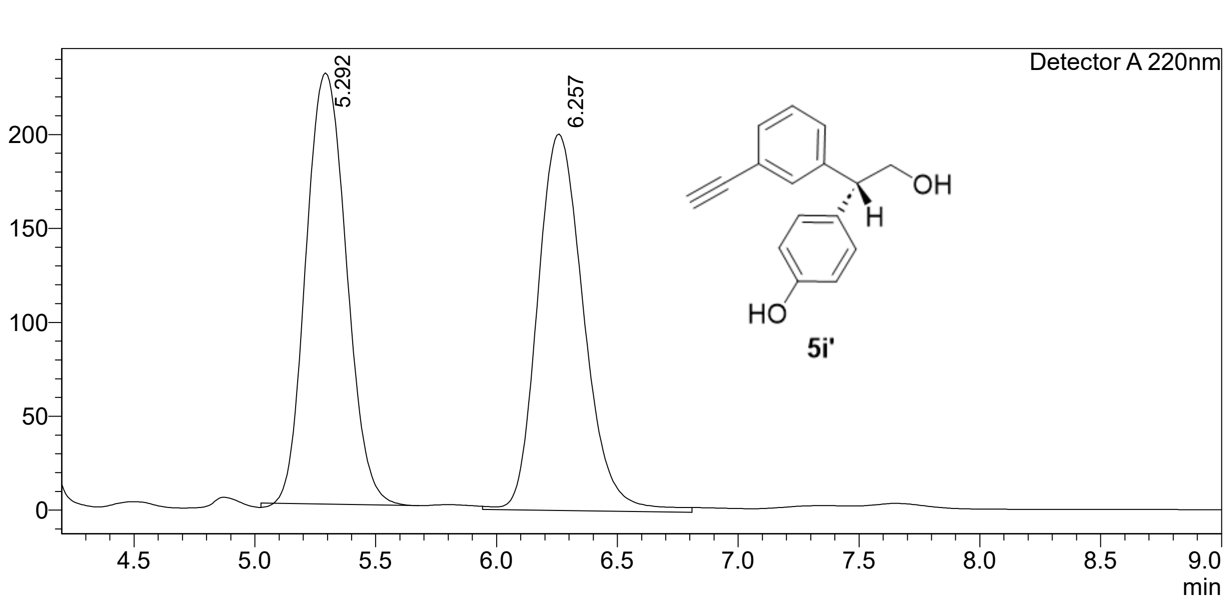

<Peak Table>

Detector A 220nm

Peak#	Ret. Time	Area	Height	Conc.	Unit	Mark	Name
1	5.292	2673790	229648	50.078		M	
2	6.257	2665493	200347	49.922		M	
Total		5339283	429995				

SHIMADZU LabSolutions Analysis Report

<Sample Information>

Sample Name	:		
Sample ID	:		
Data Filename	: 368-2-chiral-IE-20%.lcd		
Method Filename	: 20%-1ml-220nm-10min.lcm		
Batch Filename	: 368-2-chiral-IE-20%.lcd.lcb		
Vial #	: 1-4	Sample Type	: Unknown
Injection Volume	: 10 uL		
Date Acquired	: 4/21/2023 3:01:34 PM	Acquired by	: System Administrator
Date Processed	: 5/11/2023 3:39:21 PM	Processed by	: System Administrator

<Chromatogram>

mV

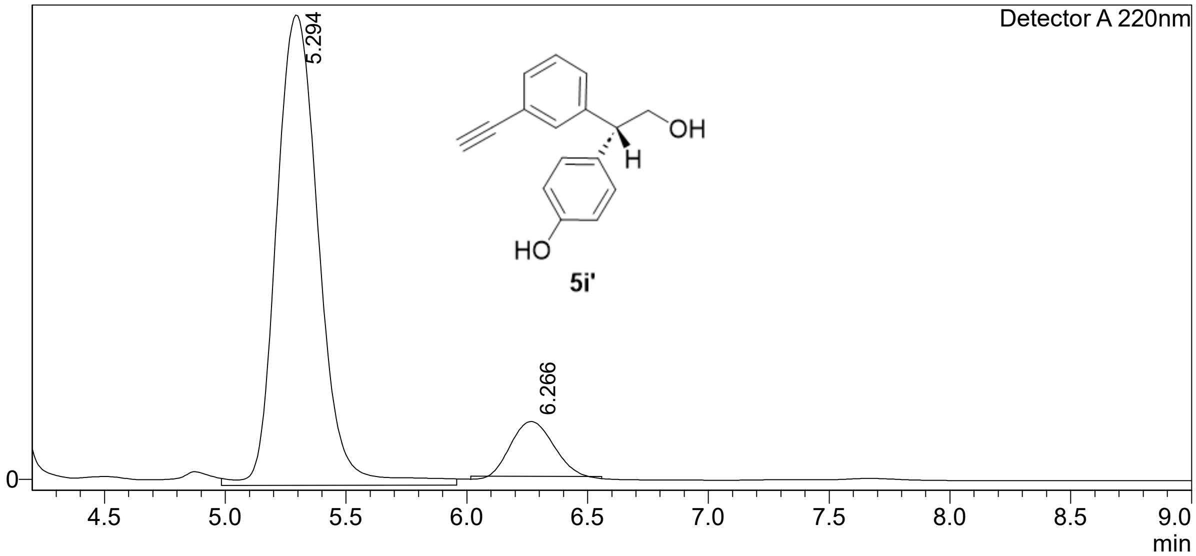

<Peak Table>

Detector A 220nm

Peak#	Ret. Time	Area	Height	Conc.	Unit	Mark	Name
1	5.294	2561411	208547	89.886		M	
2	6.266	288210	24291	10.114		M	
Total		2849621	232839				

SHIMADZU LabSolutions

Analysis Report

<Sample Information>

Sample Name	:		
Sample ID	:		
Data Filename	: S3-18-6-RAC-ie-20%-1ML.lcd		
Method Filename	: 20%-1ml-220nm-20min.lcm		
Batch Filename	: wasgf.lcb		
Vial #	: 1-2	Sample Type	: Unknown
Injection Volume	: 10 uL		
Date Acquired	: 4/14/2023 3:19:50 PM	Acquired by	: System Administrator
Date Processed	: 4/14/2023 3:31:45 PM	Processed by	: System Administrator

<Chromatogram>

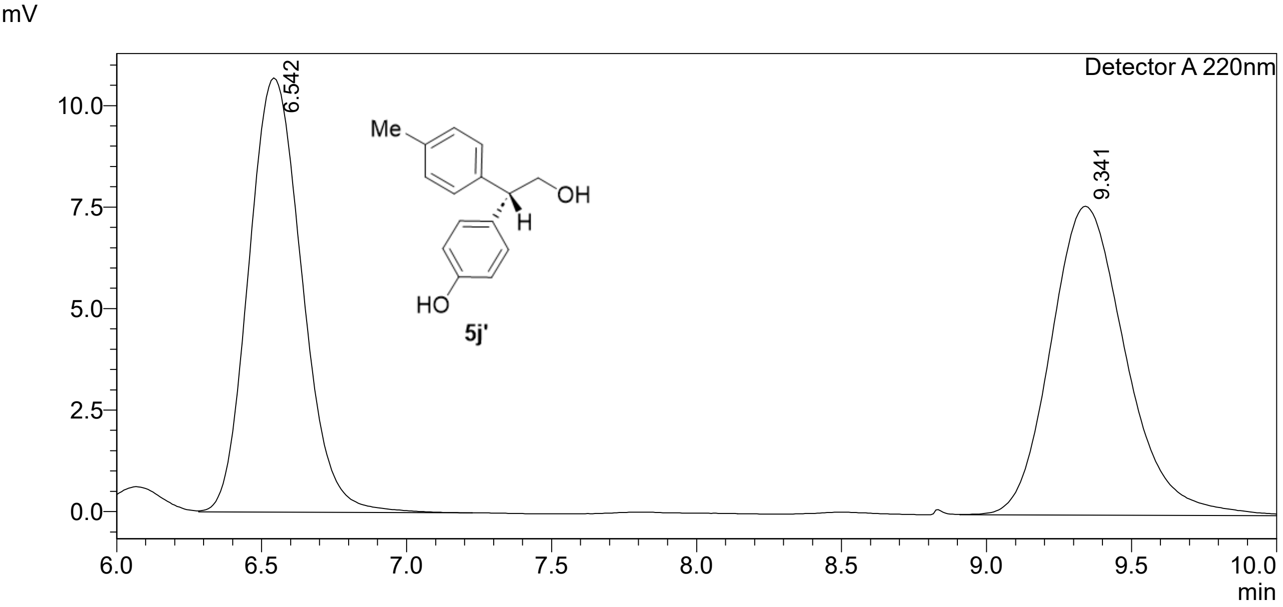

<Peak Table>

Detector A 220nm

Peak#	Ret. Time	Area	Height	Conc.	Unit	Mark	Name
1	6.542	137115	10693	50.034		M	
2	9.341	136931	7604	49.966		V	
Total		274046	18297				

SHIMADZU LabSolutions

Analysis Report

<Sample Information>

Sample Name	:		
Sample ID	:		
Data Filename	: S3-18-6-ASY-ie-20%-1ML-RE.lcd		
Method Filename	: 20%-1ml-220nm-20min.lcm		
Batch Filename	: 18-6.lcb		
Vial #	: 1-1	Sample Type	: Unknown
Injection Volume	: 10 uL		
Date Acquired	: 4/14/2023 3:33:33 PM	Acquired by	: System Administrator
Date Processed	: 4/14/2023 4:06:42 PM	Processed by	: System Administrator

<Chromatogram>

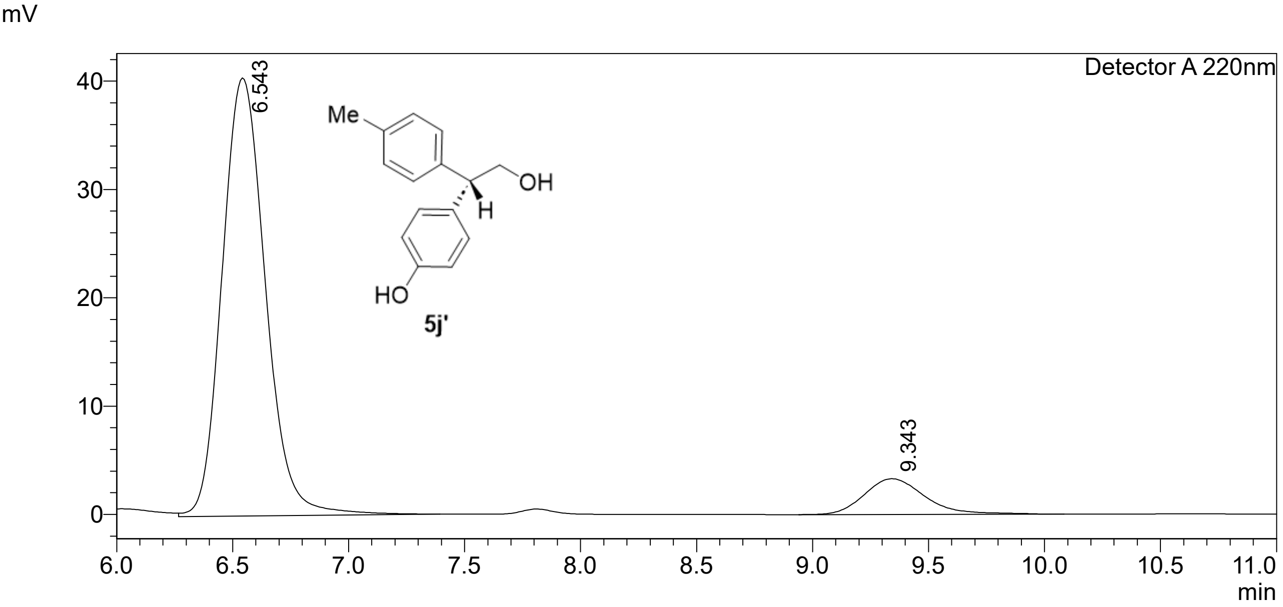

<Peak Table>

Detector A 220nm

Peak#	Ret. Time	Area	Height	Conc.	Unit	Mark	Name
1	6.543	526454	40458	89.775			
2	9.343	59959	3300	10.225			
Total		586413	43758				

SHIMADZU LabSolutions

Analysis Report

<Sample Information>

Sample Name	:		
Sample ID	:		
Data Filename	: 366-6-RAC-IE-10%.lcd		
Method Filename	: 10%-0.8ml-220nm-30min.lcm		
Batch Filename	: 366-3&366-6.lcb		
Vial #	: 1-4	Sample Type	: Unknown
Injection Volume	: 10 uL		
Date Acquired	: 4/17/2023 9:48:34 PM	Acquired by	: System Administrator
Date Processed	: 4/18/2023 10:02:20 AM	Processed by	: System Administrator

<Chromatogram>

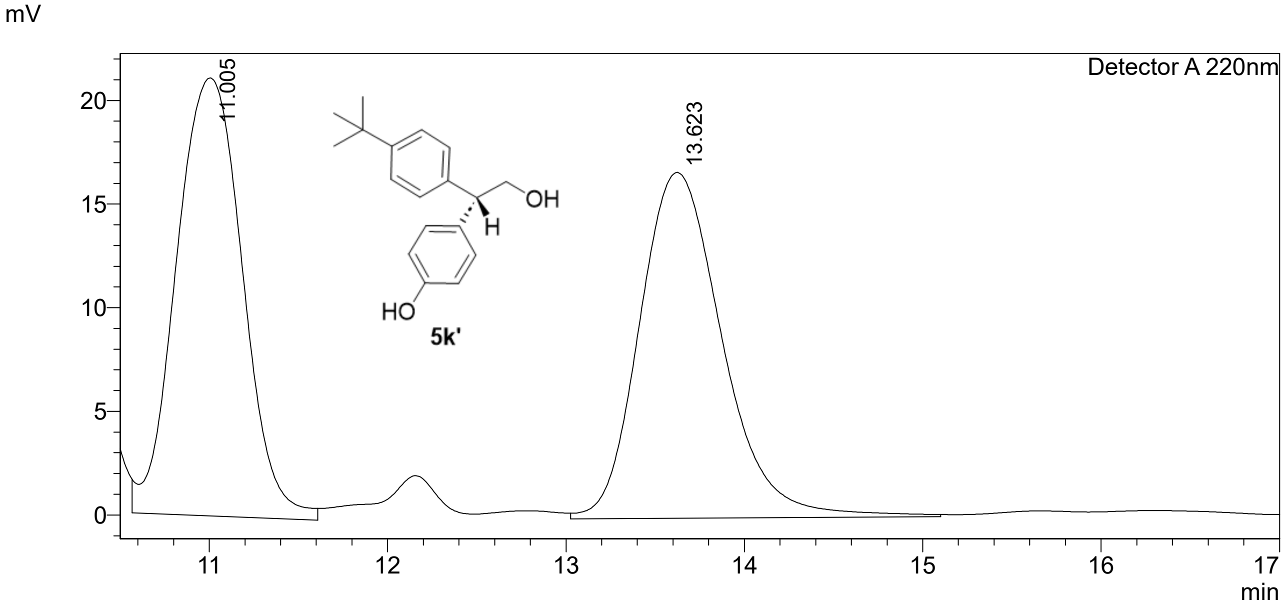

<Peak Table>

Detector A 220nm

Peak#	Ret. Time	Area	Height	Conc.	Unit	Mark	Name
1	11.005	545338	21133	50.293		M	
2	13.623	538993	16682	49.707			
Total		1084331	37816				

SHIMADZU LabSolutions

Analysis Report

<Sample Information>

Sample Name	:		
Sample ID	:		
Data Filename	: 366-6-CHIRAL-IE-10%.lcd		
Method Filename	: 10%-0.8ml-220nm-30min.lcm		
Batch Filename	: 366-3&366-6.lcb		
Vial #	: 1-5	Sample Type	: Unknown
Injection Volume	: 10 uL		
Date Acquired	: 4/17/2023 10:18:57 PM	Acquired by	: System Administrator
Date Processed	: 5/11/2023 3:34:42 PM	Processed by	: System Administrator

<Chromatogram>

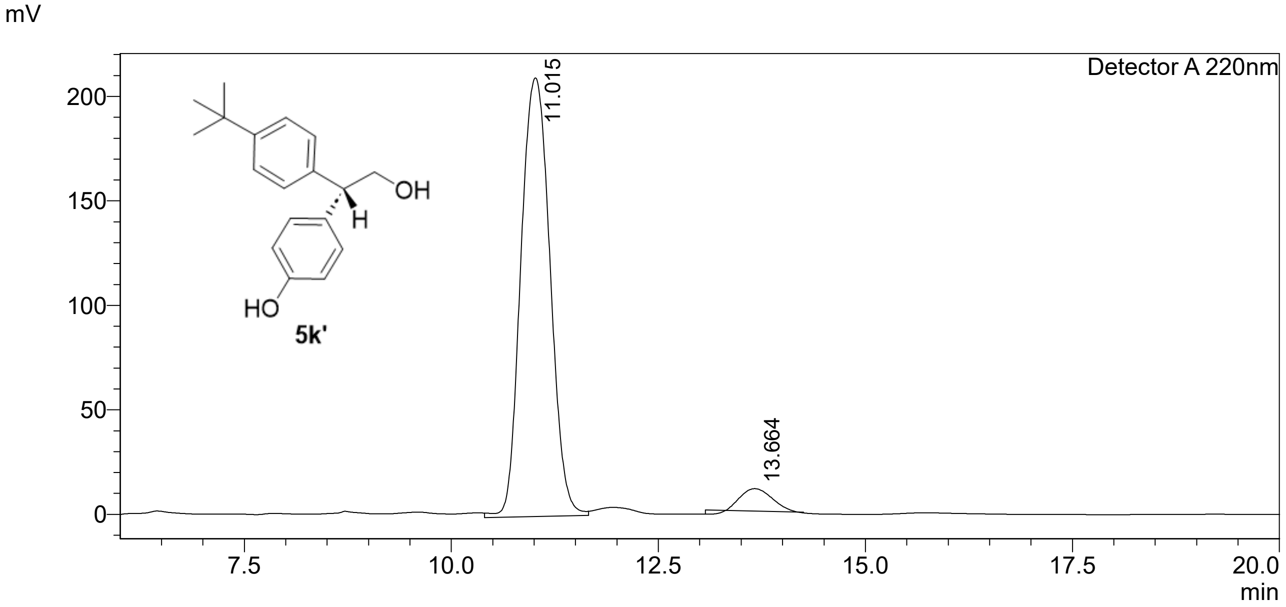

<Peak Table>

Detector A 220nm

Peak#	Ret. Time	Area	Height	Conc.	Unit	Mark	Name
1	11.015	5161645	209942	95.138		M	
2	13.664	263771	10712	4.862		M	
Total		5425416	220654				

SHIMADZU LabSolutions

Analysis Report

<Sample Information>

Sample Name	:		
Sample ID	:		
Data Filename	: s3-19-4--rac-oldIA-20%-1ml.lcd		
Method Filename	: 20%-1ml-220nm-20min.lcm		
Batch Filename	: s3-19-4--rac-oldIA-20%-1ml.lcb		
Vial #	: 1-2	Sample Type	: Unknown
Injection Volume	: 10 uL		
Date Acquired	: 4/19/2023 5:45:14 PM	Acquired by	: System Administrator
Date Processed	: 4/19/2023 6:24:04 PM	Processed by	: System Administrator

<Chromatogram>

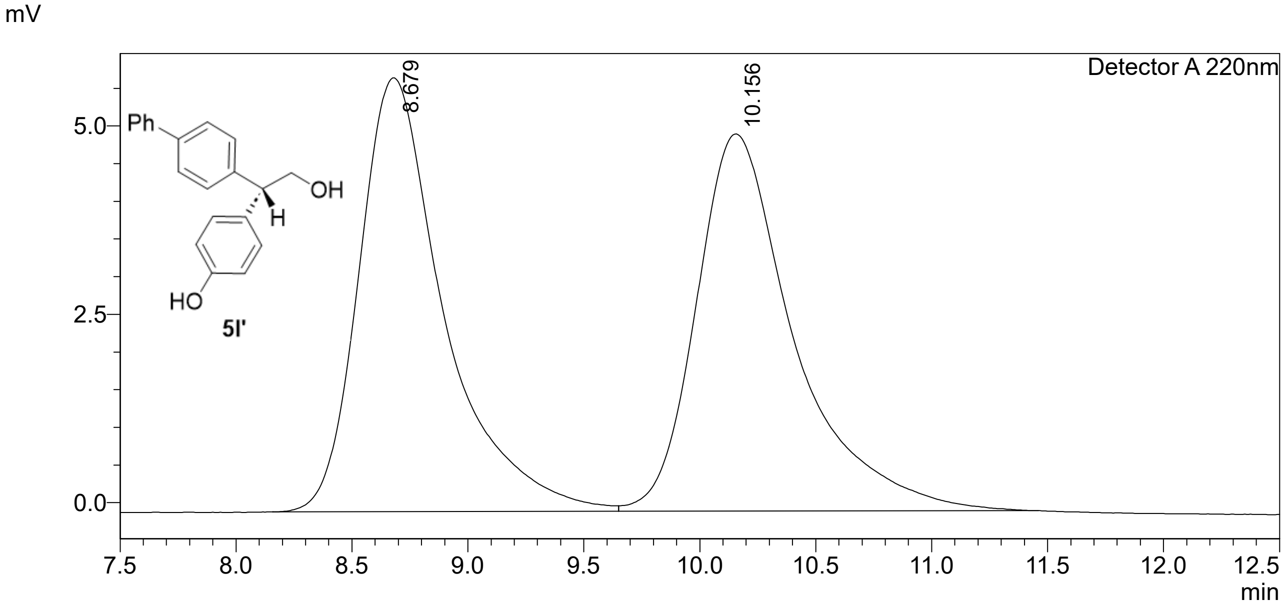

<Peak Table>

Detector A 220nm

Peak#	Ret. Time	Area	Height	Conc.	Unit	Mark	Name
1	8.679	146877	5756	49.997			
2	10.156	146897	5006	50.003		V	
Total		293774	10763				

SHIMADZU LabSolutions

Analysis Report

<Sample Information>

Sample Name	:		
Sample ID	:		
Data Filename	: s3-19-4--asy-oldIA-20%-1ml.lcd		
Method Filename	: 20%-1ml-220nm-20min.lcm		
Batch Filename	: s3-19-4--asy-oldIA-20%-1ml.lcb		
Vial #	: 1-3	Sample Type	: Unknown
Injection Volume	: 10 uL		
Date Acquired	: 4/19/2023 6:05:39 PM	Acquired by	: System Administrator
Date Processed	: 4/19/2023 6:24:31 PM	Processed by	: System Administrator

<Chromatogram>

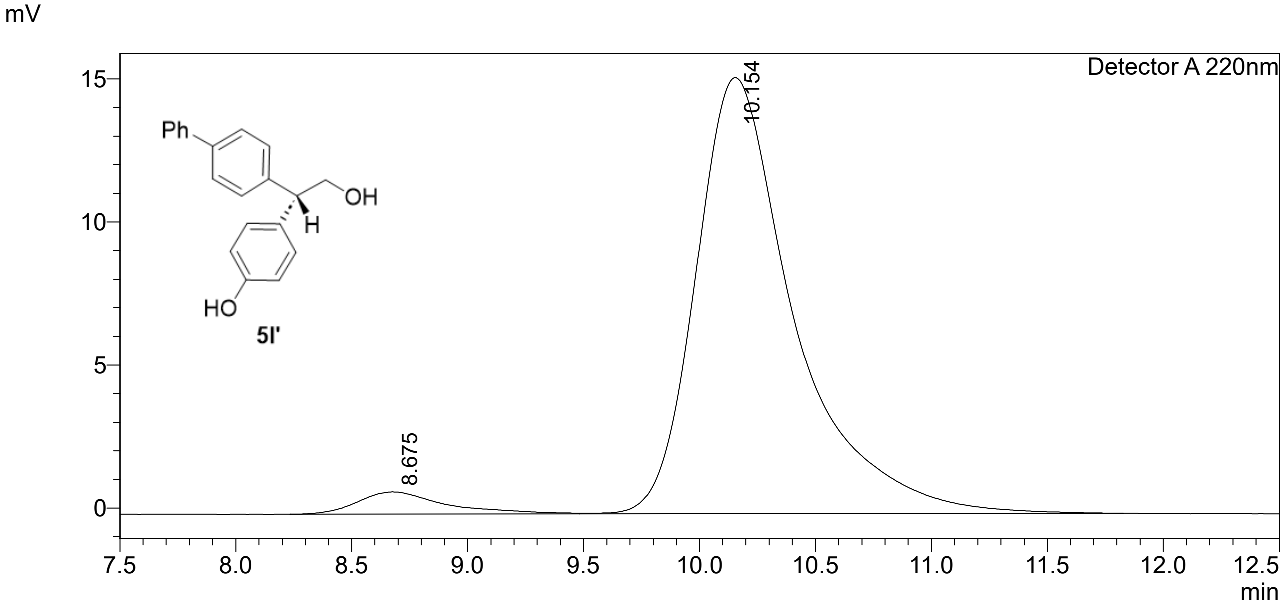

<Peak Table>

Detector A 220nm

Peak#	Ret. Time	Area	Height	Conc.	Unit	Mark	Name
1	8.675	20553	778	4.376			
2	10.154	449153	15249	95.624		V	
Total		469706	16027				

SHIMADZU LabSolutions

Analysis Report

<Sample Information>

Sample Name	:		
Sample ID	:		
Data Filename	: 375-1-RAC-IE-10%.lcd		
Method Filename	: 10%1ml-220nm-40min.lcm		
Batch Filename	: 375-1.lcb		
Vial #	: 1-1	Sample Type	: Unknown
Injection Volume	: 10 uL		
Date Acquired	: 5/8/2023 12:47:35 PM	Acquired by	: System Administrator
Date Processed	: 5/8/2023 3:47:22 PM	Processed by	: System Administrator

<Chromatogram>

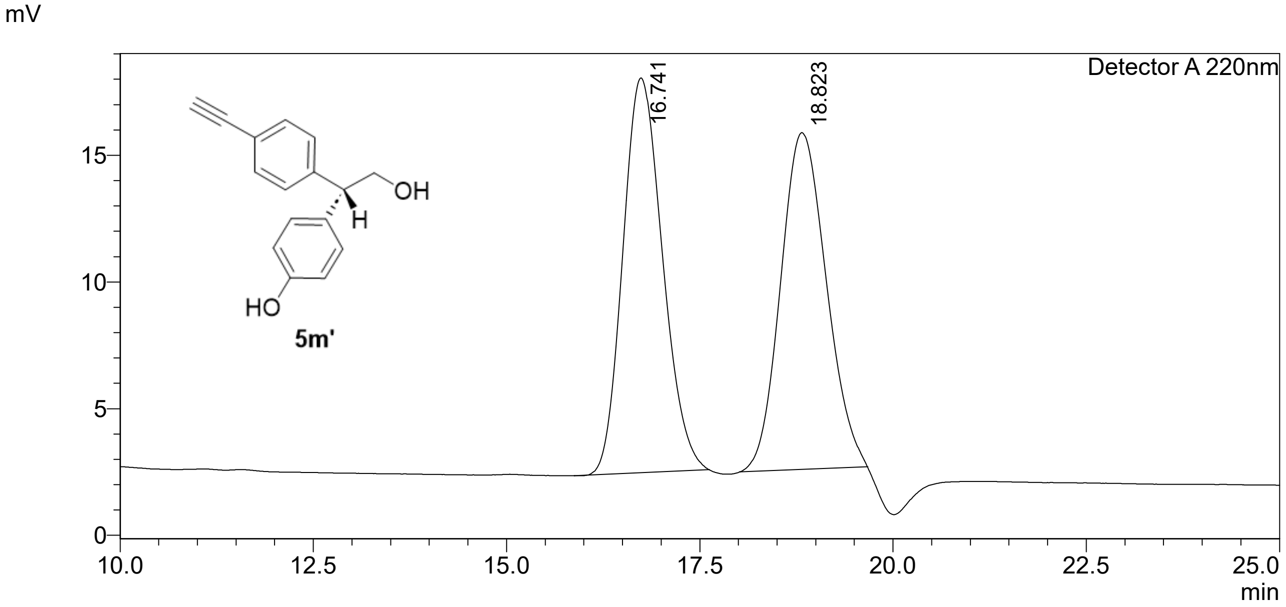

<Peak Table>

Detector A 220nm

Peak#	Ret. Time	Area	Height	Conc.	Unit	Mark	Name
1	16.741	555471	15580	50.402		M	
2	18.823	546616	13291	49.598		M	
Total		1102086	28871				

SHIMADZU LabSolutions

Analysis Report

<Sample Information>

Sample Name	:		
Sample ID	:		
Data Filename	: 375-1-CHIRAL-IE-10%.lcd		
Method Filename	: 10%1ml-220nm-40min.lcm		
Batch Filename	: 375-1.lcb		
Vial #	: 1-2	Sample Type	: Unknown
Injection Volume	: 10 uL		
Date Acquired	: 5/8/2023 1:28:00 PM	Acquired by	: System Administrator
Date Processed	: 5/8/2023 3:47:58 PM	Processed by	: System Administrator

<Chromatogram>

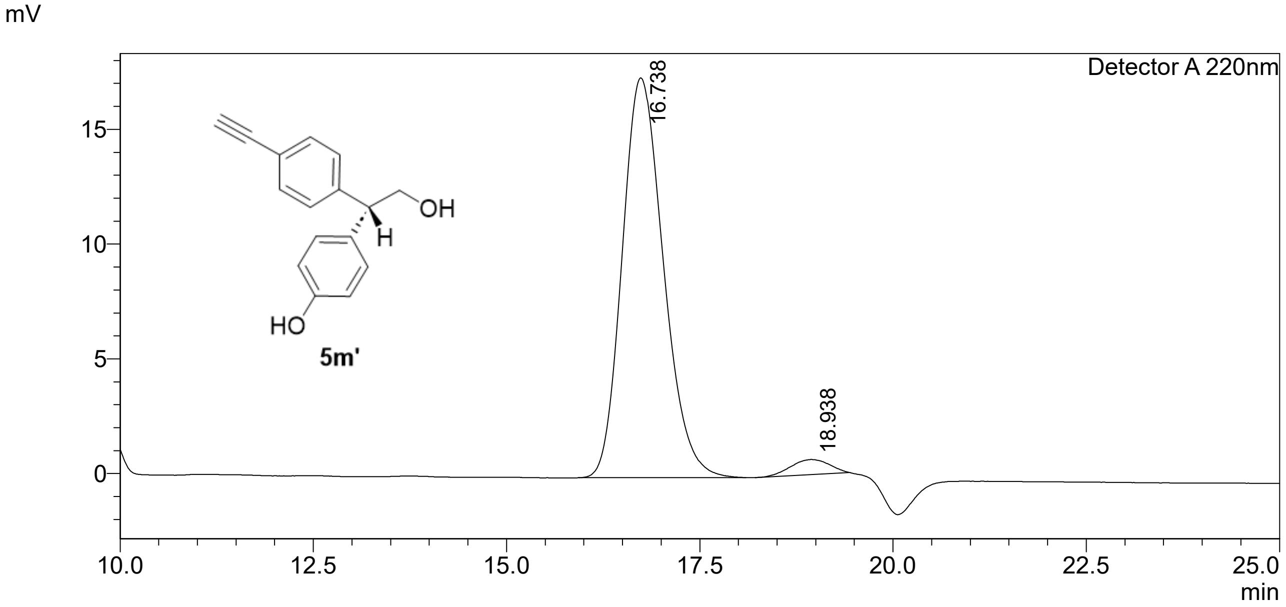

<Peak Table>

Detector A 220nm

Peak#	Ret. Time	Area	Height	Conc.	Unit	Mark	Name
1	16.738	646338	17416	96.556			
2	18.938	23051	659	3.444		M	
Total		669389	18075				

SHIMADZU LabSolutions

Analysis Report

<Sample Information>

Sample Name	:		
Sample ID	:		
Data Filename	: 366-2-RAC-IE-10%.lcd		
Method Filename	: 10%-0.8ml-220nm-45min.lcm		
Batch Filename	: 366-2-RAC-IE-10%.lcd.lcb		
Vial #	: 1-3	Sample Type	: Unknown
Injection Volume	: 10 uL		
Date Acquired	: 4/17/2023 7:36:09 PM	Acquired by	: System Administrator
Date Processed	: 4/17/2023 8:45:24 PM	Processed by	: System Administrator

<Chromatogram>

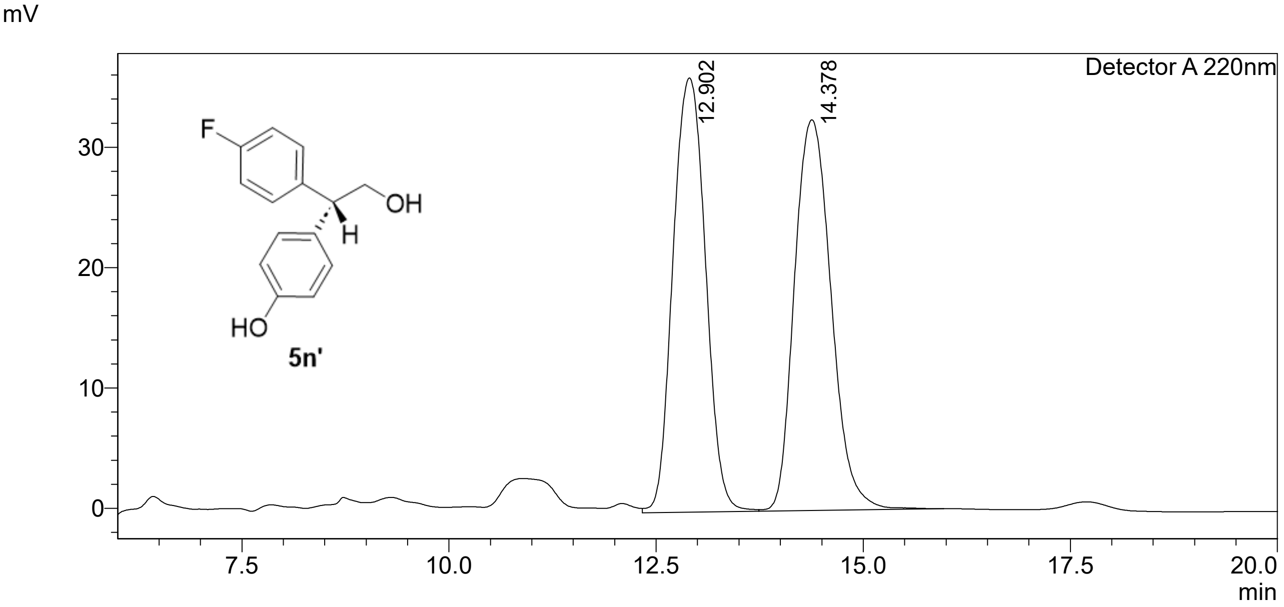

<Peak Table>

Detector A 220nm

Peak#	Ret. Time	Area	Height	Conc.	Unit	Mark	Name
1	12.902	986647	36072	49.602			
2	14.378	1002472	32458	50.398		SV	
Total		1989118	68530				

SHIMADZU LabSolutions

Analysis Report

<Sample Information>

Sample Name	:		
Sample ID	:		
Data Filename	: 366-2-CHIRAL-IE-10%.lcd		
Method Filename	: 10%-0.8ml-220nm-20min.lcm		
Batch Filename	: 366-2-CHIRAL-IE-10%.lcd.lcb		
Vial #	: 1-1	Sample Type	: Unknown
Injection Volume	: 10 uL		
Date Acquired	: 4/17/2023 8:27:17 PM	Acquired by	: System Administrator
Date Processed	: 5/11/2023 3:30:44 PM	Processed by	: System Administrator

<Chromatogram>

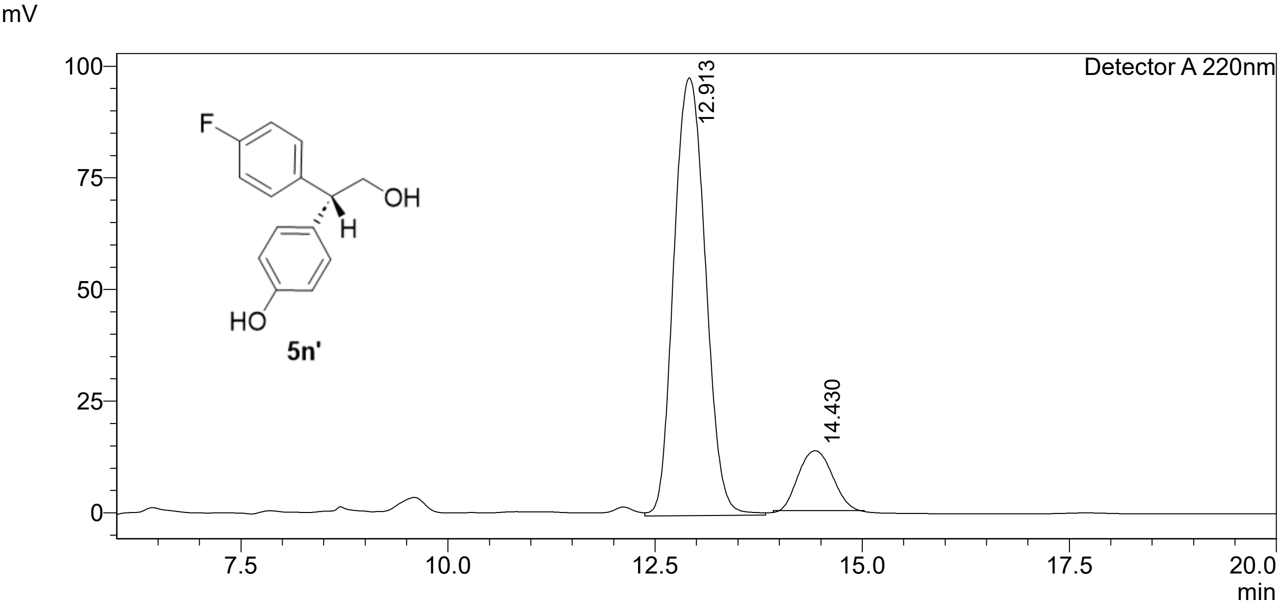

<Peak Table>

Detector A 220nm

Peak#	Ret. Time	Area	Height	Conc.	Unit	Mark	Name
1	12.913	2614742	98024	87.361		M	
2	14.430	378277	13402	12.639		M	
Total		2993019	111426				

SHIMADZU LabSolutions

Analysis Report

<Sample Information>

Sample Name	:		
Sample ID	:		
Data Filename	: S3-18-4-RAC-ie-20%-1ML.lcd		
Method Filename	: 20%-1ml-220nm-20min.lcm		
Batch Filename	: S3-18-4-6.lcb		
Vial #	: 1-3	Sample Type	: Unknown
Injection Volume	: 10 uL		
Date Acquired	: 4/13/2023 4:06:59 PM	Acquired by	: System Administrator
Date Processed	: 4/14/2023 9:21:04 AM	Processed by	: System Administrator

<Chromatogram>

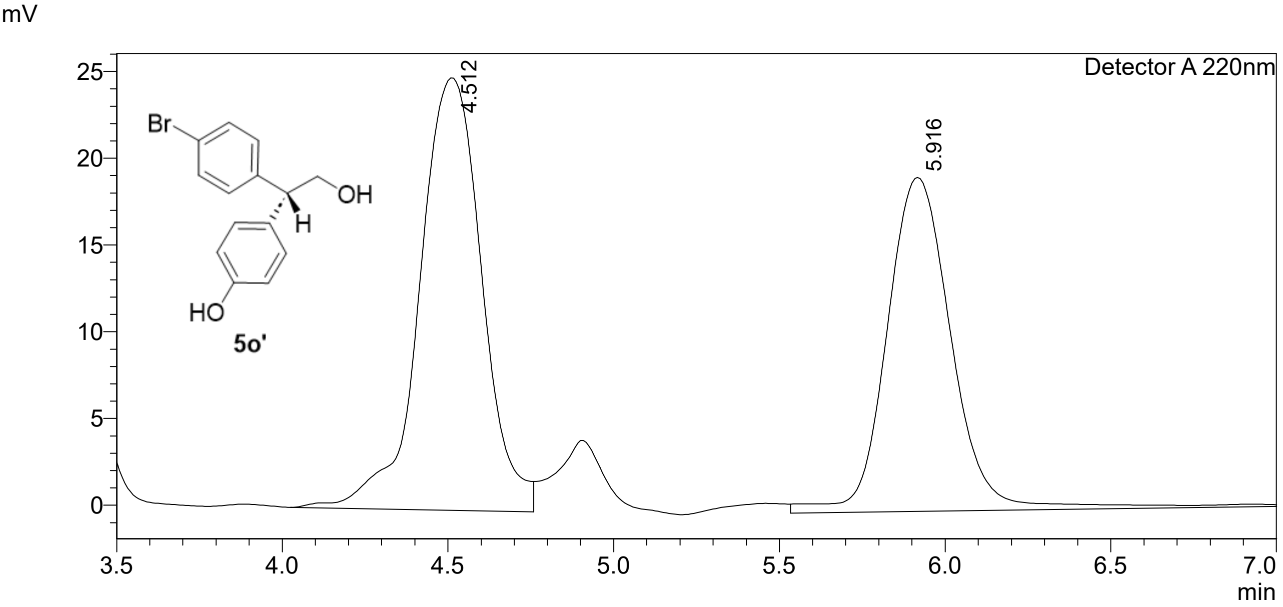

<Peak Table>

Detector A 220nm

Peak#	Ret. Time	Area	Height	Conc.	Unit	Mark	Name
1	4.512	327800	24925	55.306			
2	5.916	264903	19261	44.694		S	
Total		592703	44186				

SHIMADZU LabSolutions

Analysis Report

<Sample Information>

Sample Name	:		
Sample ID	:		
Data Filename	: S3-18-4-ASY-ie-20%-1ML.lcd		
Method Filename	: 20%-1ml-220nm-20min.lcm		
Batch Filename	: S3-18-4-6.lcb		
Vial #	: 1-4	Sample Type	: Unknown
Injection Volume	: 10 uL		
Date Acquired	: 4/13/2023 4:27:23 PM	Acquired by	: System Administrator
Date Processed	: 4/14/2023 9:21:55 AM	Processed by	: System Administrator

<Chromatogram>

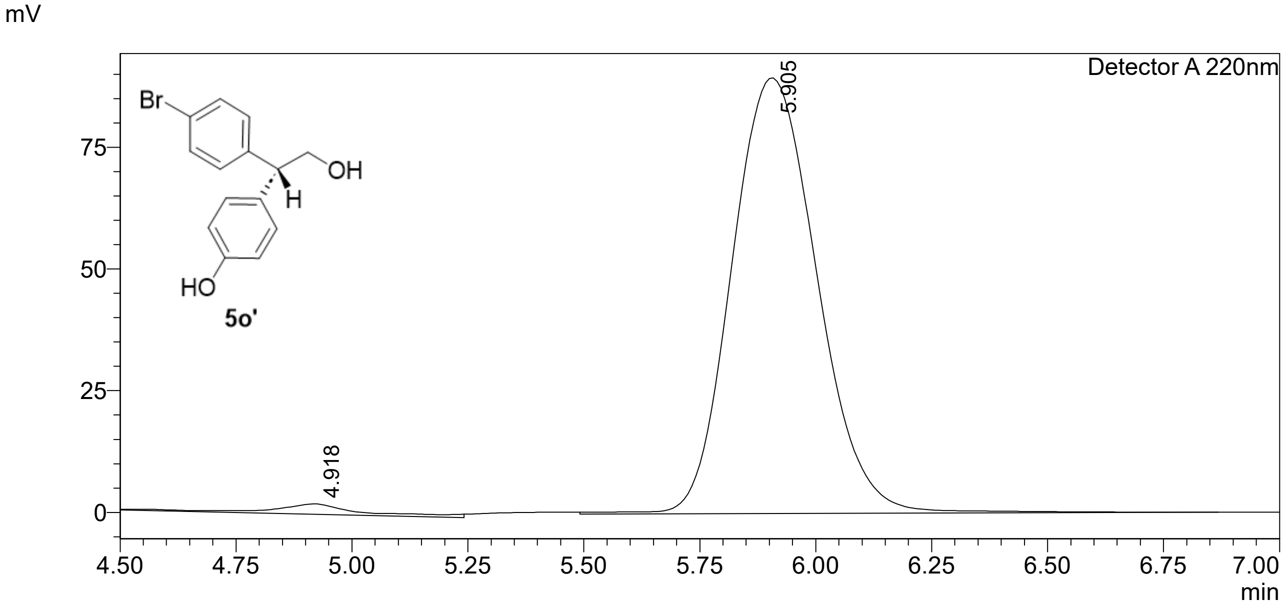

<Peak Table>

Detector A 220nm

Peak#	Ret. Time	Area	Height	Conc.	Unit	Mark	Name
1	4.918	28445	2123	2.419		M	
2	5.905	1147367	89507	97.581			
Total		1175813	91630				

SHIMADZU LabSolutions

Analysis Report

<Sample Information>

Sample Name	:		
Sample ID	:		
Data Filename	: 373-2(re)-RAC-IE-20%.lcd		
Method Filename	: 20%-1ml-220nm-20min.lcm		
Batch Filename	: 373-2-ie-20%.lcb		
Vial #	: 1-1	Sample Type	: Unknown
Injection Volume	: 10 uL		
Date Acquired	: 5/5/2023 7:13:01 PM	Acquired by	: System Administrator
Date Processed	: 5/5/2023 8:25:01 PM	Processed by	: System Administrator

<Chromatogram>

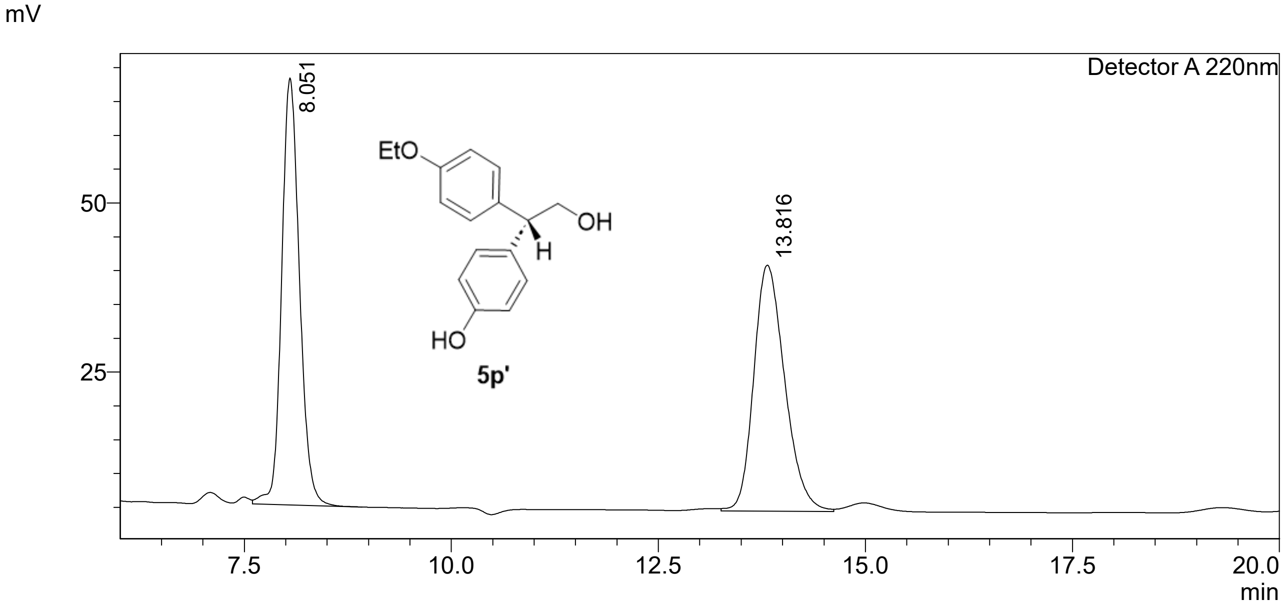

<Peak Table>

Detector A 220nm

Peak#	Ret. Time	Area	Height	Conc.	Unit	Mark	Name
1	8.051	947856	63136	49.992		M	
2	13.816	948146	36365	50.008			
Total		1896002	99501				

SHIMADZU LabSolutions

Analysis Report

<Sample Information>

Sample Name	:		
Sample ID	:		
Data Filename	: 373-2-chiral-IE-20%.lcd		
Method Filename	: 20%-1ml-220nm-20min.lcm		
Batch Filename	: 373-2-ie-20%.lcb		
Vial #	: 1-2	Sample Type	: Unknown
Injection Volume	: 10 uL		
Date Acquired	: 5/5/2023 7:33:24 PM	Acquired by	: System Administrator
Date Processed	: 5/11/2023 3:44:50 PM	Processed by	: System Administrator

<Chromatogram>

mV

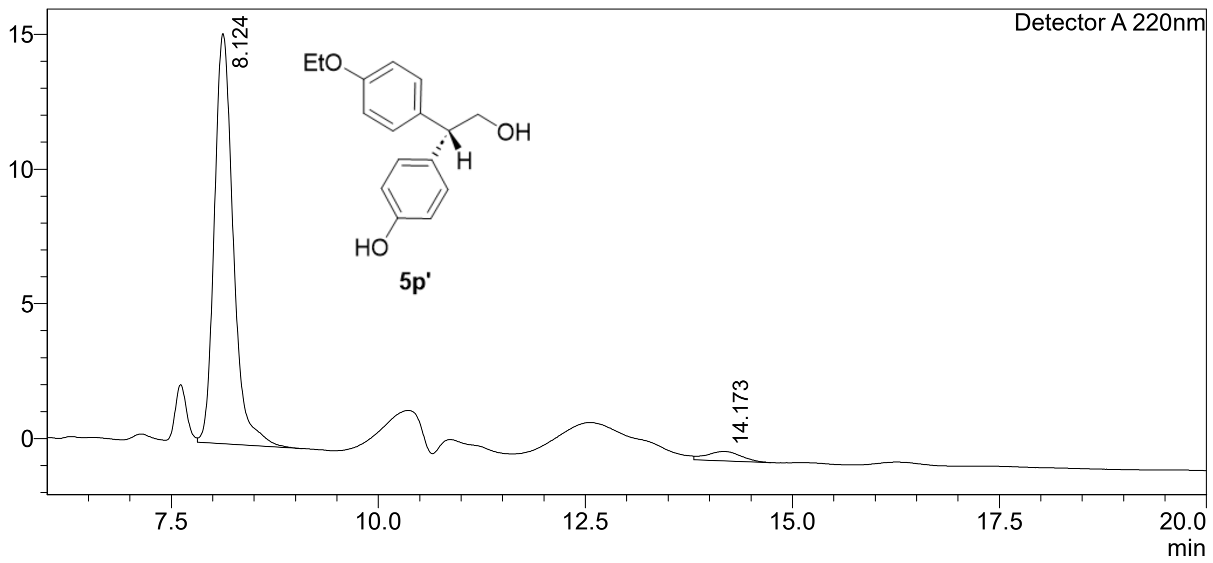

<Peak Table>

Detector A 220nm

Peak#	Ret. Time	Area	Height	Conc.	Unit	Mark	Name
1	8.124	241410	15220	95.973			
2	14.173	10130	350	4.027		M	
Total		251540	15570				

SHIMADZU LabSolutions

Analysis Report

<Sample Information>

Sample Name	:		
Sample ID	:		
Data Filename	: 368-1-RAC-IE-20%.lcd		
Method Filename	: 20%1ml-220nm-60min.lcm		
Batch Filename	: 368-1-RAC-IE-20%.lcd.lcb		
Vial #	: 1-1	Sample Type	: Unknown
Injection Volume	: 10 uL		
Date Acquired	: 4/21/2023 12:50:02 PM	Acquired by	: System Administrator
Date Processed	: 10/12/2023 10:09:03 AM	Processed by	: System Administrator

<Chromatogram>

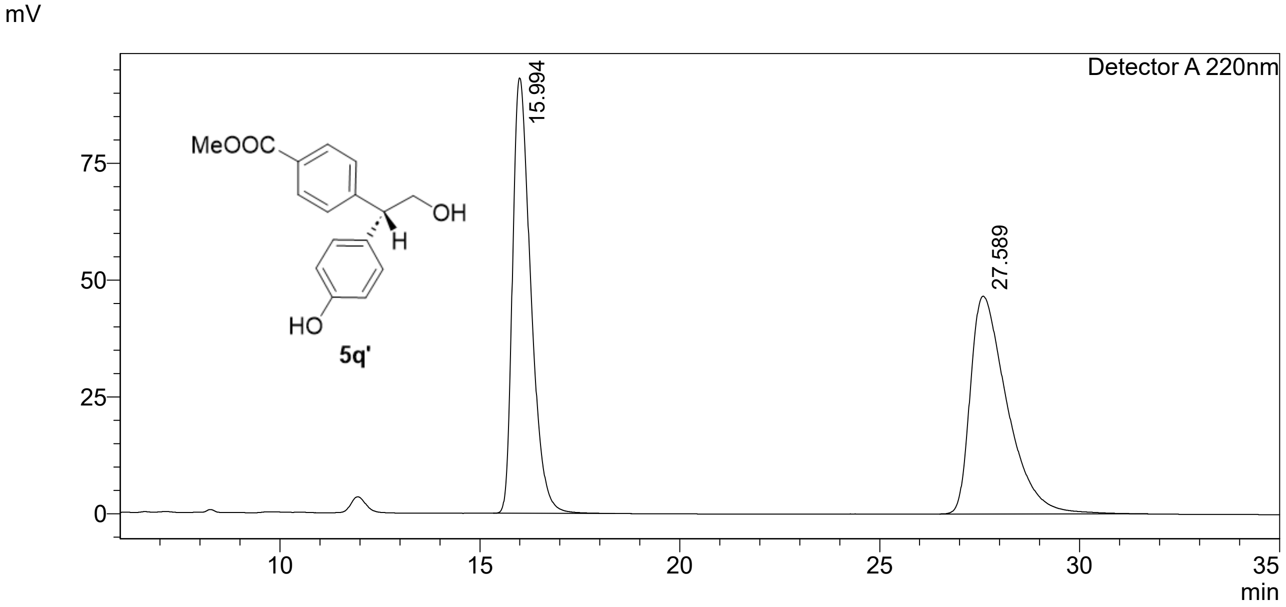

<Peak Table>

Detector A 220nm

Peak#	Ret. Time	Area	Height	Conc.	Unit	Mark	Name
1	15.994	2955155	93150	49.738			
2	27.589	2986251	46604	50.262			
Total		5941406	139755				

SHIMADZU LabSolutions

Analysis Report

<Sample Information>

Sample Name	:		
Sample ID	:		
Data Filename	: 368-1-CHIRAL-IE-20%.lcd		
Method Filename	: 20%-1ml-220nm-40min.lcm		
Batch Filename	: 368-1-CHIRAL-IE-20%.lcd.lcb		
Vial #	: 1-2	Sample Type	: Unknown
Injection Volume	: 10 uL		
Date Acquired	: 4/21/2023 1:57:20 PM	Acquired by	: System Administrator
Date Processed	: 5/11/2023 3:36:47 PM	Processed by	: System Administrator

<Chromatogram>

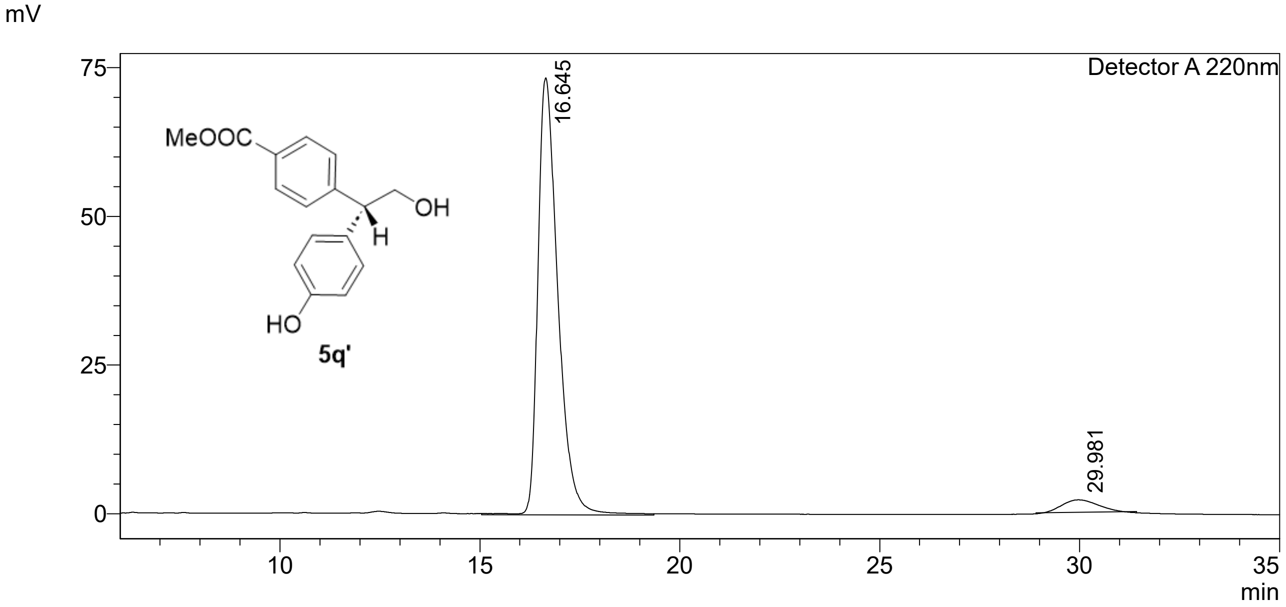

<Peak Table>

Detector A 220nm

Peak#	Ret. Time	Area	Height	Conc.	Unit	Mark	Name
1	16.645	2563298	73471	95.331		M	
2	29.981	125544	2087	4.669		M	
Total		2688842	75559				

SHIMADZU LabSolutions

Analysis Report

<Sample Information>

Sample Name	:		
Sample ID	:		
Data Filename	: s3-19-3-rac-oldIA-20%-1ml.lcd		
Method Filename	: 20%-1ml-220nm-20min.lcm		
Batch Filename	: s3-19-2-asy-IA-20%-1ml.lcb		
Vial #	: 1-1	Sample Type	: Unknown
Injection Volume	: 10 uL		
Date Acquired	: 4/19/2023 4:55:26 PM	Acquired by	: System Administrator
Date Processed	: 4/19/2023 5:16:06 PM	Processed by	: System Administrator

<Chromatogram>

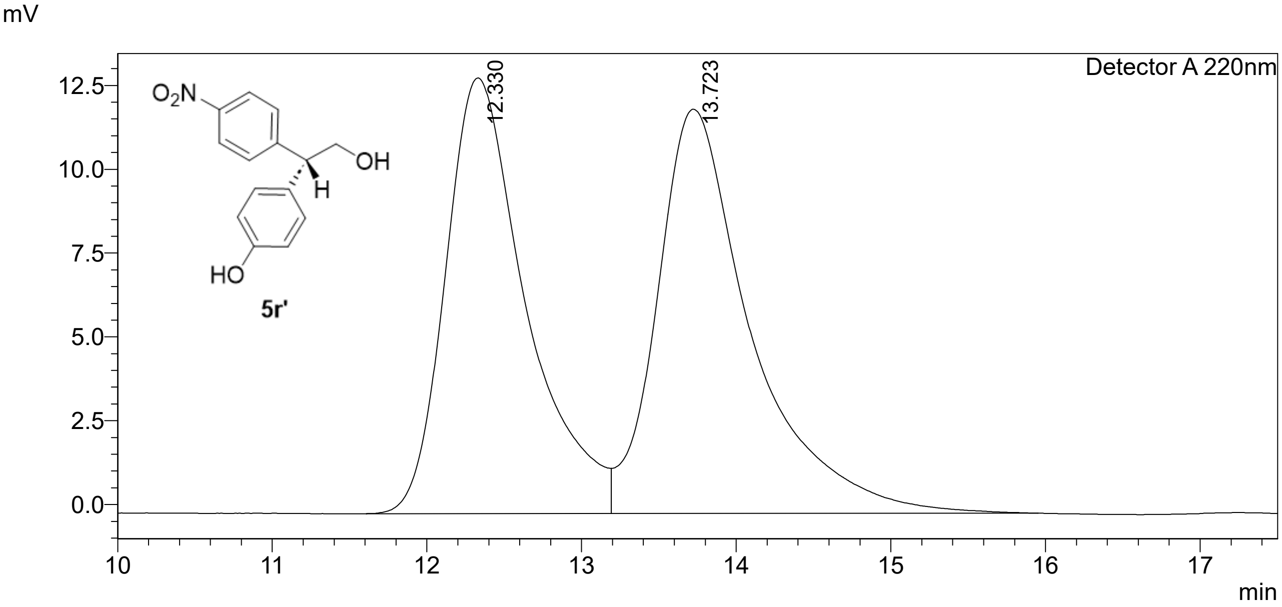

<Peak Table>

Detector A 220nm

Peak#	Ret. Time	Area	Height	Conc.	Unit	Mark	Name
1	12.330	469786	12993	48.005			
2	13.723	508823	12058	51.995		V	
Total		978609	25051				

SHIMADZU LabSolutions

Analysis Report

<Sample Information>

Sample Name	:		
Sample ID	:		
Data Filename	: s3-19-3-asy-oldIA-20%-1ml.lcd		
Method Filename	: 20%1ml-220nm-60min.lcm		
Batch Filename	: s3-19-3-rac-IA-20%-1ml.lcb		
Vial #	: 1-1	Sample Type	: Unknown
Injection Volume	: 10 uL		
Date Acquired	: 4/19/2023 5:15:35 PM	Acquired by	: System Administrator
Date Processed	: 4/19/2023 5:32:10 PM	Processed by	: System Administrator

<Chromatogram>

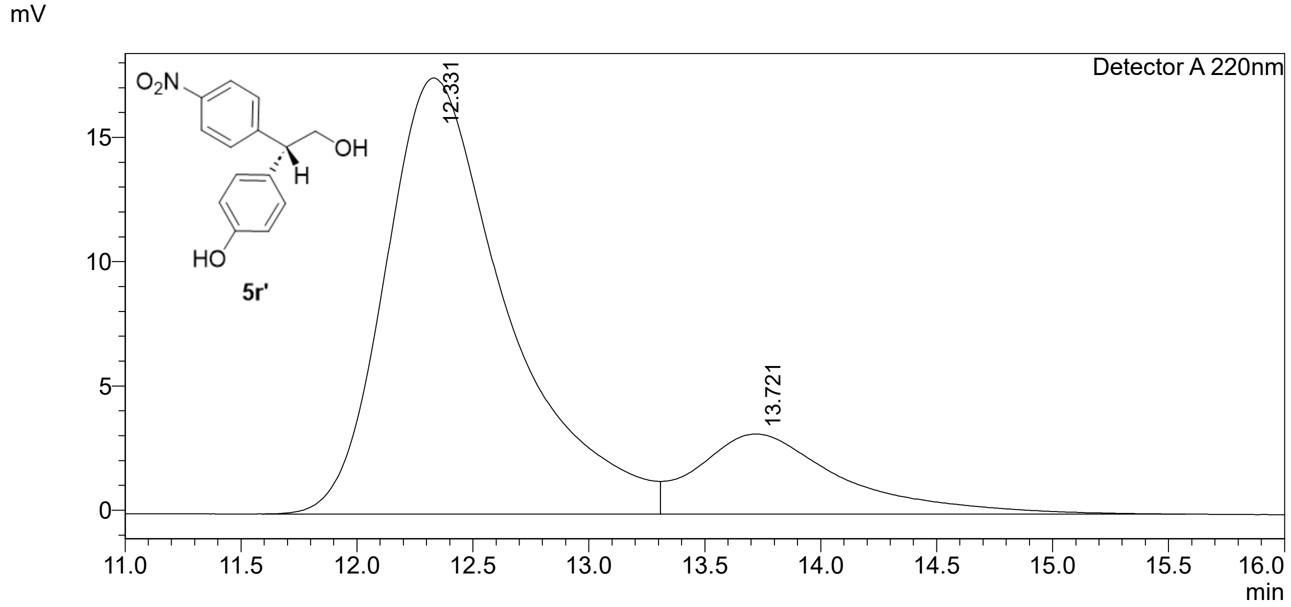

<Peak Table>

Detector A 220nm

Peak#	Ret. Time	Area	Height	Conc.	Unit	Mark	Name
1	12.331	642247	17538	82.118			
2	13.721	139854	3218	17.882		V	
Total		782101	20756				

SHIMADZU LabSolutions

Analysis Report

<Sample Information>

Sample Name	:		
Sample ID	:		
Data Filename	: s3-21-5-rac-IE-10%-1ml.lcd		
Method Filename	: 10%1ml-220nm-40min.lcm		
Batch Filename	: s3-21-5-rac-IE-10%-1ml.lcb		
Vial #	: 1-1	Sample Type	: Unknown
Injection Volume	: 10 uL		
Date Acquired	: 5/10/2023 11:29:20 AM	Acquired by	: System Administrator
Date Processed	: 5/10/2023 12:15:01 PM	Processed by	: System Administrator

<Chromatogram>

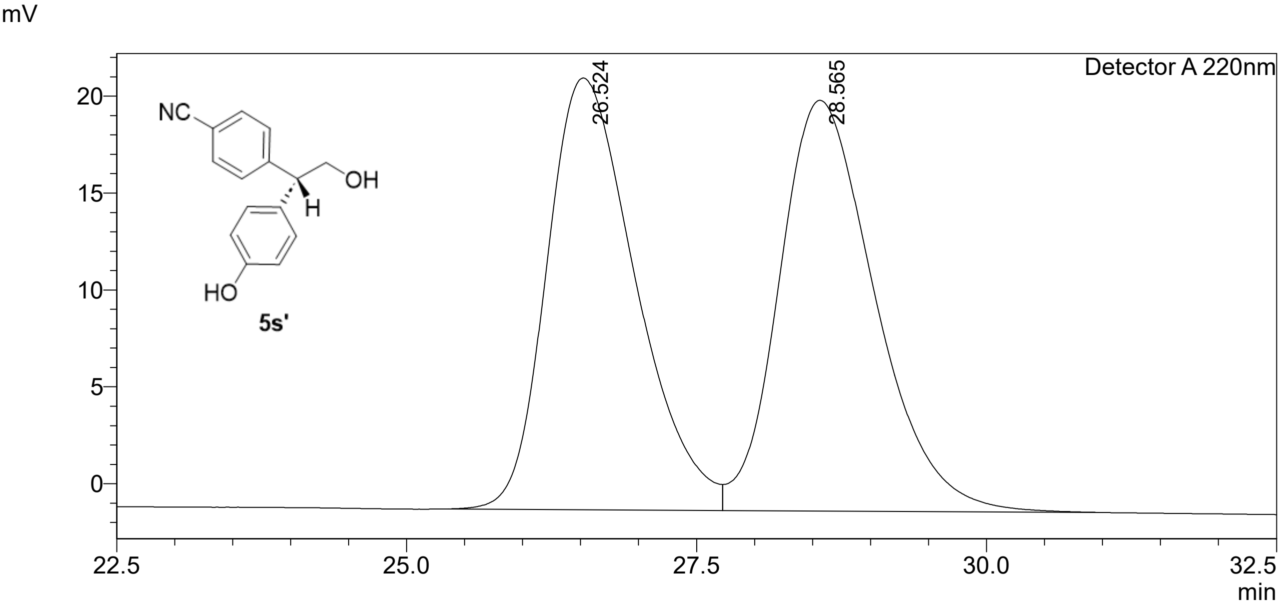

<Peak Table>

Detector A 220nm

Peak#	Ret. Time	Area	Height	Conc.	Unit	Mark	Name
1	26.524	1186519	22277	49.370			
2	28.565	1216811	21191	50.630		V	
Total		2403330	43468				

SHIMADZU LabSolutions

Analysis Report

<Sample Information>

Sample Name	:		
Sample ID	:		
Data Filename	: s3-21-5-asy-IE-10%-1ml.lcd		
Method Filename	: 10%1ml-220nm-40min.lcm		
Batch Filename	: s3-21-5-asy-IE-10%-1ml.lcb		
Vial #	: 1-2	Sample Type	: Unknown
Injection Volume	: 10 uL		
Date Acquired	: 5/10/2023 12:09:44 PM	Acquired by	: System Administrator
Date Processed	: 5/10/2023 1:14:36 PM	Processed by	: System Administrator

<Chromatogram>

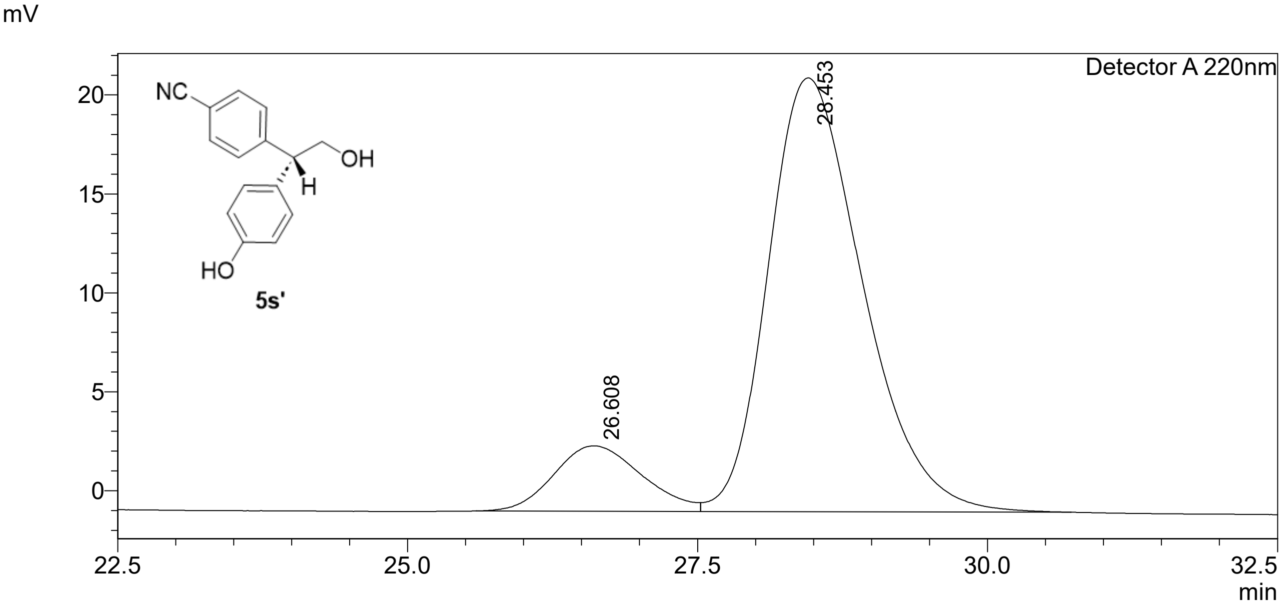

<Peak Table>

Detector A 220nm

Peak#	Ret. Time	Area	Height	Conc.	Unit	Mark	Name
1	26.608	174480	3306	12.330			
2	28.453	1240605	21921	87.670		V	
Total		1415085	25227				

SHIMADZU LabSolutions

Analysis Report

<Sample Information>

Sample Name	:		
Sample ID	:		
Data Filename	: 373-3(re)-RAC-IE-10%.lcd		
Method Filename	: 10%1ml-220nm-20min.lcm		
Batch Filename	: 373-3-ie-10%.lcb		
Vial #	: 1-3	Sample Type	: Unknown
Injection Volume	: 10 uL		
Date Acquired	: 5/5/2023 3:28:44 PM	Acquired by	: System Administrator
Date Processed	: 5/5/2023 3:57:45 PM	Processed by	: System Administrator

<Chromatogram>

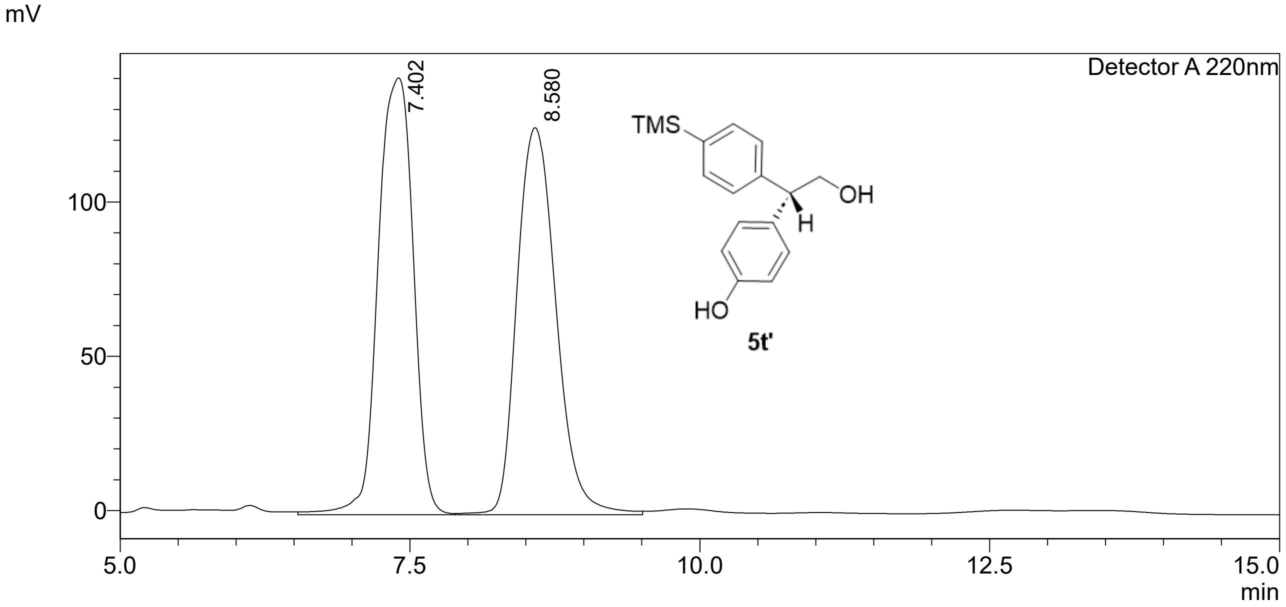

<Peak Table>

Detector A 220nm

Peak#	Ret. Time	Area	Height	Conc.	Unit	Mark	Name
1	7.402	2991733	141572	50.349			
2	8.580	2950313	125447	49.651		V	
Total		5942046	267019				

SHIMADZU LabSolutions

Analysis Report

<Sample Information>

Sample Name	:		
Sample ID	:		
Data Filename	: 373-3-chiral-IE-10%.lcd		
Method Filename	: 10%1ml-220nm-20min.lcm		
Batch Filename	: 373-3-ie-10%.lcb		
Vial #	: 1-4	Sample Type	: Unknown
Injection Volume	: 10 uL		
Date Acquired	: 5/5/2023 3:49:07 PM	Acquired by	: System Administrator
Date Processed	: 5/5/2023 4:09:40 PM	Processed by	: System Administrator

<Chromatogram>

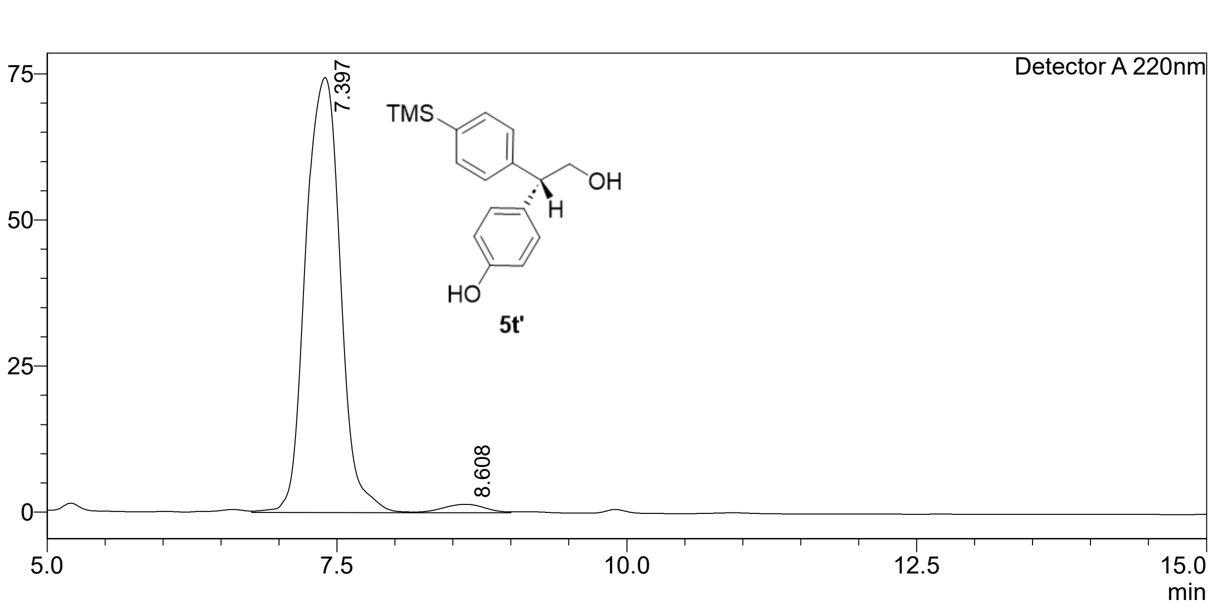

<Peak Table>

Detector A 220nm

Peak#	Ret. Time	Area	Height	Conc.	Unit	Mark	Name
1	7.397	1549778	74407	97.665			
2	8.608	37048	1459	2.335		V	
Total		1586826	75866				

SHIMADZU LabSolutions

Analysis Report

<Sample Information>

Sample Name	:		
Sample ID	:		
Data Filename	: s3-19-1-rac-IE-20%-1ml.lcd		
Method Filename	: 20%-1ml-220nm-20min.lcm		
Batch Filename	: s3-19-1-rac-IE-20%-1ml.lcb		
Vial #	: 1-1	Sample Type	: Unknown
Injection Volume	: 10 uL		
Date Acquired	: 4/19/2023 6:52:40 PM	Acquired by	: System Administrator
Date Processed	: 4/19/2023 7:18:09 PM	Processed by	: System Administrator

<Chromatogram>

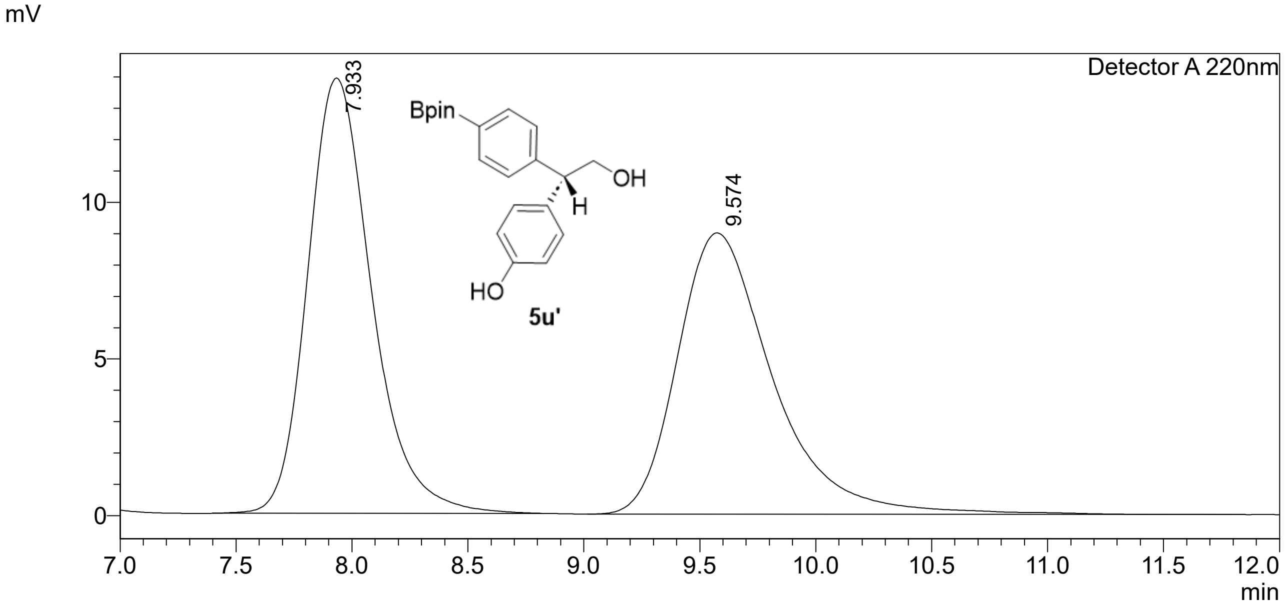

<Peak Table>

Detector A 220nm

Peak#	Ret. Time	Area	Height	Conc.	Unit	Mark	Name
1	7.933	266523	13888	50.929		M	
2	9.574	256798	8972	49.071		M	
Total		523321	22860				

SHIMADZU LabSolutions

Analysis Report

<Sample Information>

Sample Name	:		
Sample ID	:		
Data Filename	: s3-19-1-asy-IE-20%-1ml.lcd		
Method Filename	: 20%-1ml-220nm-20min.lcm		
Batch Filename	: s3-19-1-asy-IE-20%-1ml.lcb		
Vial #	: 1-1	Sample Type	: Unknown
Injection Volume	: 10 uL		
Date Acquired	: 4/19/2023 7:07:15 PM	Acquired by	: System Administrator
Date Processed	: 4/19/2023 7:21:52 PM	Processed by	: System Administrator

<Chromatogram>

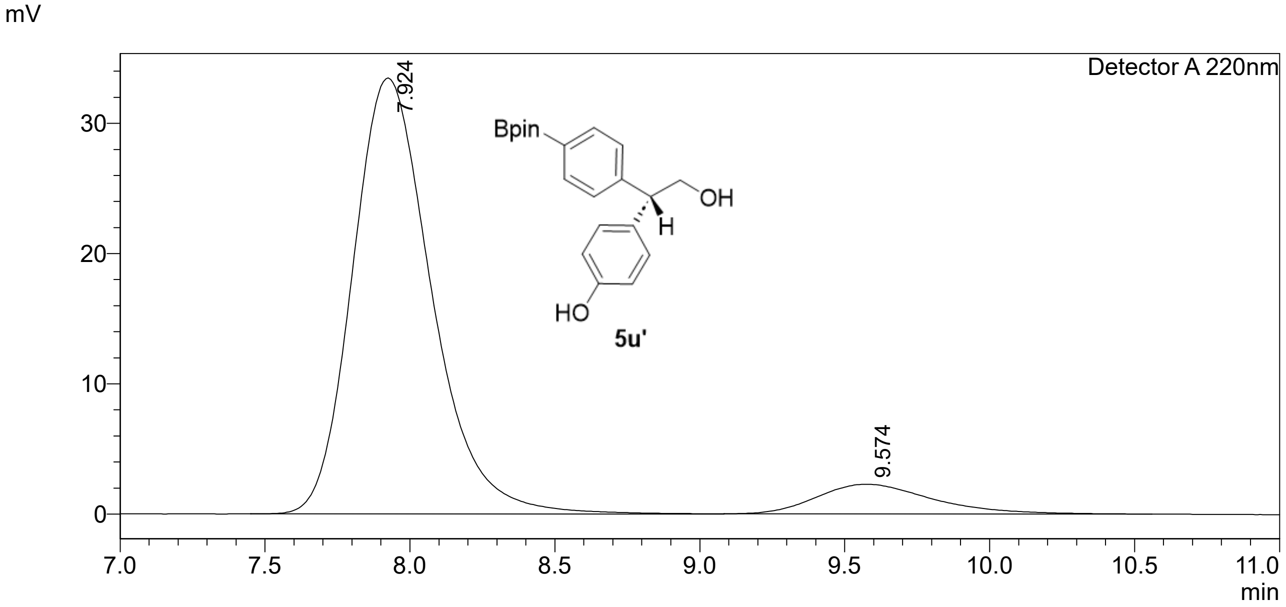

<Peak Table>

Detector A 220nm

Peak#	Ret. Time	Area	Height	Conc.	Unit	Mark	Name
1	7.924	635103	33475	90.963			
2	9.574	63098	2278	9.037		M	
Total		698202	35752				

SHIMADZU LabSolutions

Analysis Report

<Sample Information>

Sample Name	:		
Sample ID	:		
Data Filename	: 373-5(re)-RAC-IE-10%.lcd		
Method Filename	: 10%1ml-220nm-20min.lcm		
Batch Filename	: 373-5-ie-10%.lcb		
Vial #	: 1-1	Sample Type	: Unknown
Injection Volume	: 10 uL		
Date Acquired	: 5/5/2023 4:09:33 PM	Acquired by	: System Administrator
Date Processed	: 5/5/2023 4:31:41 PM	Processed by	: System Administrator

<Chromatogram>

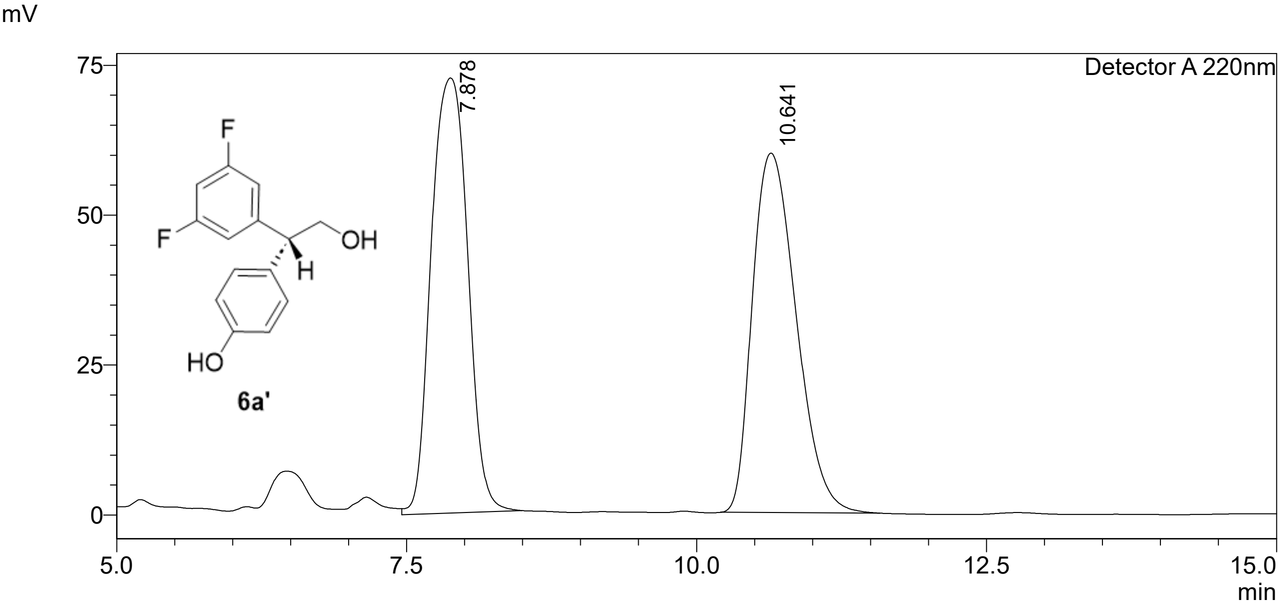

<Peak Table>

Detector A 220nm

Peak#	Ret. Time	Area	Height	Conc.	Unit	Mark	Name
1	7.878	1588514	72552	50.175		M	
2	10.641	1577428	59972	49.825		M	
Total		3165941	132524				

SHIMADZU LabSolutions

Analysis Report

<Sample Information>

Sample Name	:		
Sample ID	:		
Data Filename	: 373-5-chiral-IE-10%.lcd		
Method Filename	: 10%1ml-220nm-20min.lcm		
Batch Filename	: 373-5-ie-10%.lcb		
Vial #	: 1-2	Sample Type	: Unknown
Injection Volume	: 10 uL		
Date Acquired	: 5/5/2023 4:29:57 PM	Acquired by	: System Administrator
Date Processed	: 5/5/2023 4:51:11 PM	Processed by	: System Administrator

<Chromatogram>

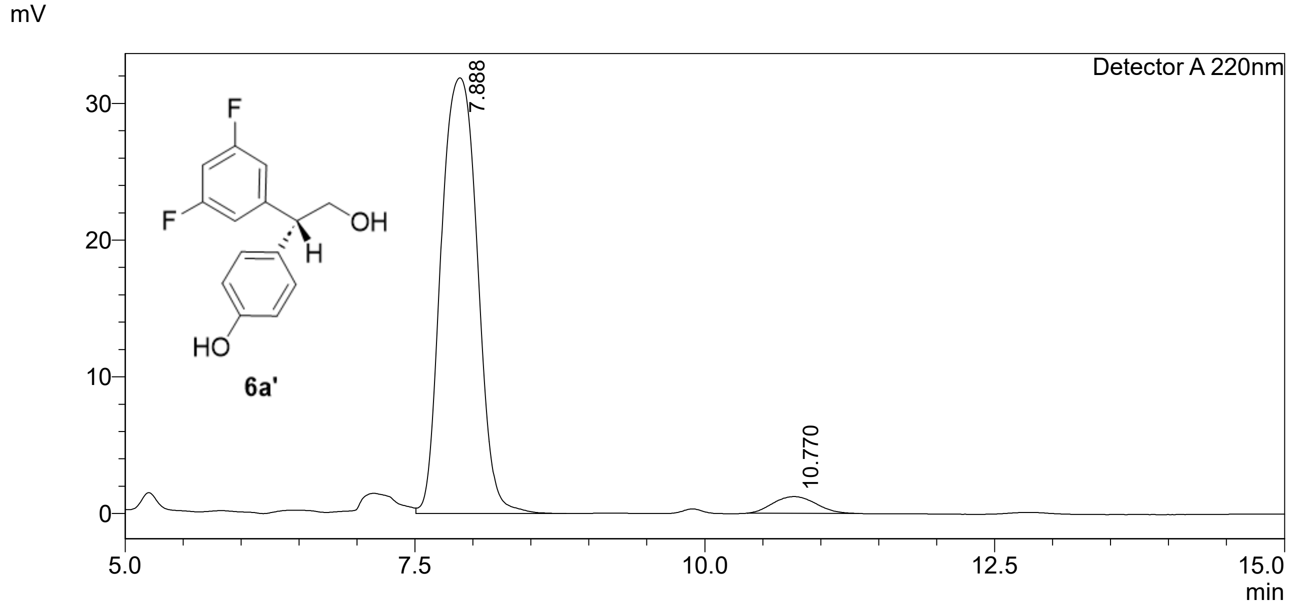

<Peak Table>

Detector A 220nm

Peak#	Ret. Time	Area	Height	Conc.	Unit	Mark	Name
1	7.888	693933	31858	95.656			
2	10.770	31516	1223	4.344		M	
Total		725450	33081				

SHIMADZU LabSolutions

Analysis Report

<Sample Information>

Sample Name	:		
Sample ID	:		
Data Filename	: 375-3-RAC-IE-5%.lcd		
Method Filename	: 5%-1ml-220nm-60min.lcm		
Batch Filename	: 375-3-RAC-IE-5%.lcd.lcb		
Vial #	: 1-1	Sample Type	: Unknown
Injection Volume	: 10 uL		
Date Acquired	: 5/8/2023 3:08:08 PM	Acquired by	: System Administrator
Date Processed	: 5/8/2023 4:12:30 PM	Processed by	: System Administrator

<Chromatogram>

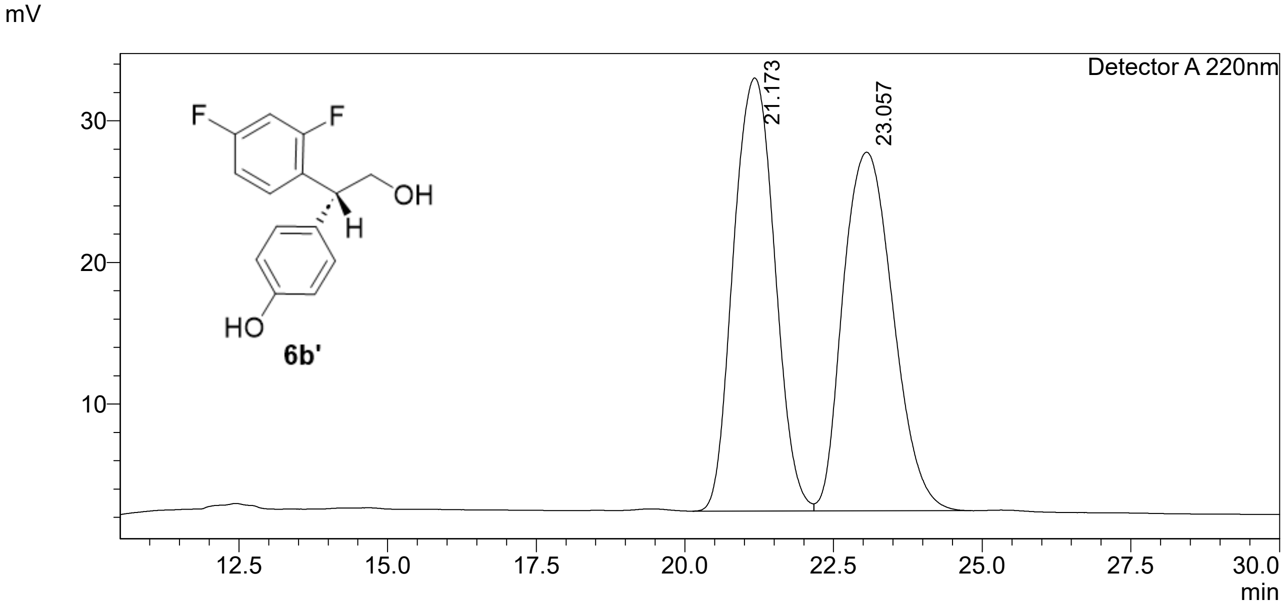

<Peak Table>

Detector A 220nm

Peak#	Ret. Time	Area	Height	Conc.	Unit	Mark	Name
1	21.173	1484204	30581	50.010			
2	23.057	1483590	25326	49.990		V	
Total		2967795	55906				

SHIMADZU LabSolutions

Analysis Report

<Sample Information>

Sample Name	:		
Sample ID	:		
Data Filename	: 375-3-CHIRAL-IE-5%.lcd		
Method Filename	: 5%-1ml-220nm-30min.lcm		
Batch Filename	: 375-3-CHIRAL-IE-5%.lcd.lcb		
Vial #	: 1-2	Sample Type	: Unknown
Injection Volume	: 10 uL		
Date Acquired	: 5/8/2023 4:08:34 PM	Acquired by	: System Administrator
Date Processed	: 5/11/2023 3:45:46 PM	Processed by	: System Administrator

<Chromatogram>

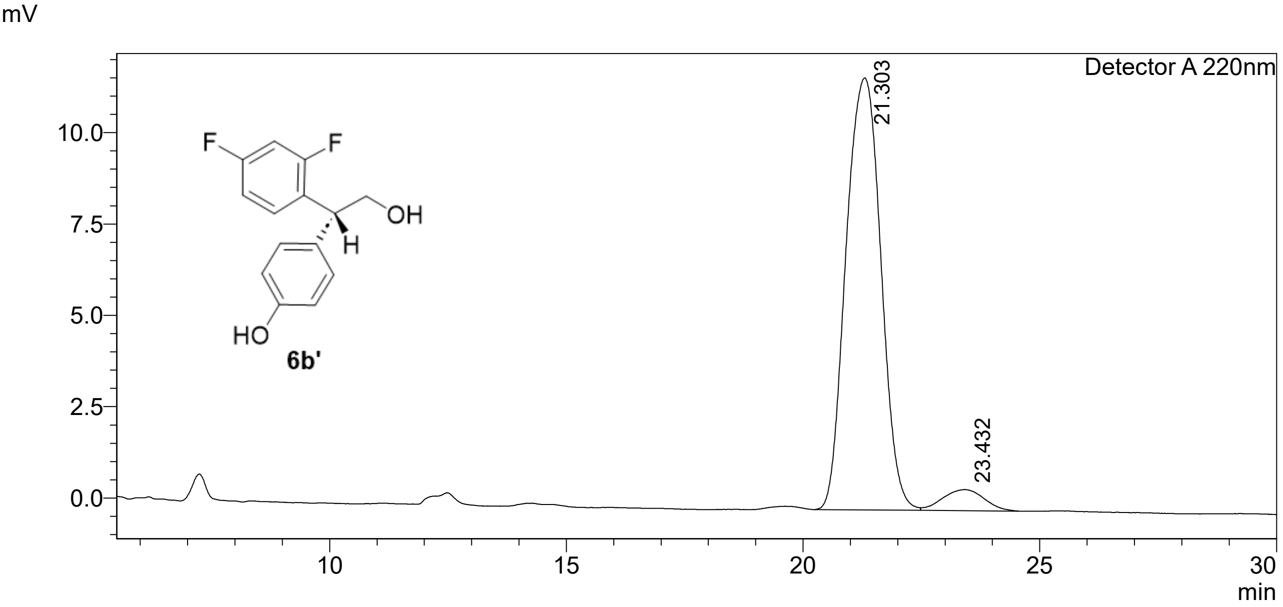

<Peak Table>

Detector A 220nm

Peak#	Ret. Time	Area	Height	Conc.	Unit	Mark	Name
1	21.303	606682	11822	94.524			
2	23.432	35148	577	5.476		V	
Total		641830	12399				

SHIMADZU LabSolutions

Analysis Report

<Sample Information>

Sample Name	:		
Sample ID	:		
Data Filename	: S3-22-3-RAC-IE-10%-1ML.lcd		
Method Filename	: 10%1ml-220nm-40min.lcm		
Batch Filename	: S3-22-3-RAC-IE-10%-1ML.lcb		
Vial #	: 1-1	Sample Type	: Unknown
Injection Volume	: 10 uL		
Date Acquired	: 5/11/2023 3:24:08 PM	Acquired by	: System Administrator
Date Processed	: 5/11/2023 4:02:36 PM	Processed by	: System Administrator

<Chromatogram>

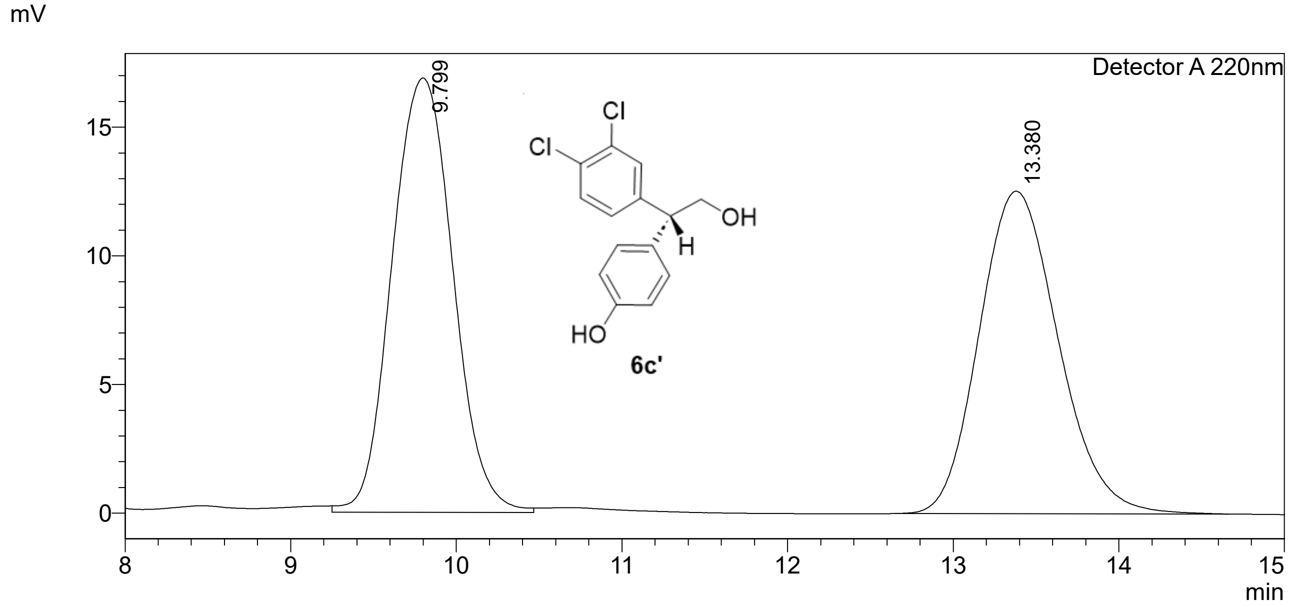

<Peak Table>

Detector A 220nm

Peak#	Ret. Time	Area	Height	Conc.	Unit	Mark	Name
1	9.799	432214	16877	50.947			
2	13.380	416143	12533	49.053			
Total		848357	29409				

SHIMADZU LabSolutions

Analysis Report

<Sample Information>

Sample Name	:		
Sample ID	:		
Data Filename	: S3-39-1-ASY-IE-10%-1ML.lcd		
Method Filename	: 10%1ml-220nm-40min.lcm		
Batch Filename	: S3-39-1-ASY-IE-10%-1ML.lcb		
Vial #	: 1-1	Sample Type	: Unknown
Injection Volume	: 10 uL		
Date Acquired	: 7/25/2023 4:28:47 PM	Acquired by	: System Administrator
Date Processed	: 10/3/2023 4:59:01 PM	Processed by	: System Administrator

<Chromatogram>

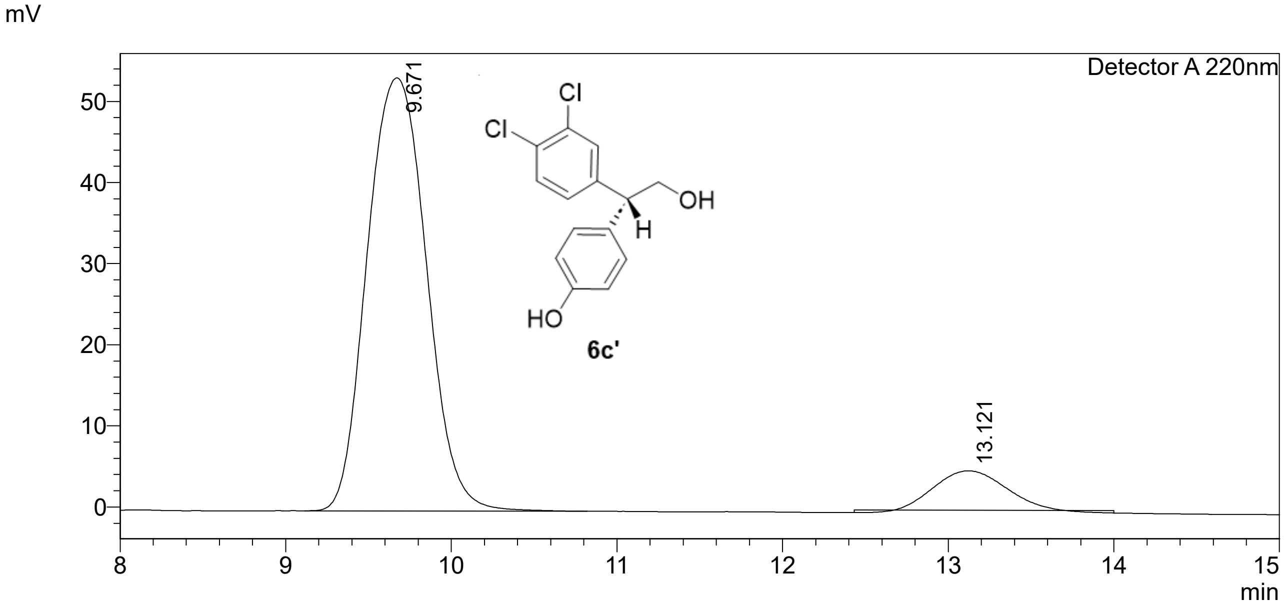

<Peak Table>

Detector A 220nm

Peak#	Ret. Time	Area	Height	Conc.	Unit	Mark	Name
1	9.671	1329959	53372	90.512			
2	13.121	139411	4864	9.488		M	
Total		1469370	58236				

SHIMADZU LabSolutions

Analysis Report

<Sample Information>

Sample Name	:		
Sample ID	:		
Data Filename	: s3-21-6-rac-IA-20%-1ml.lcd		
Method Filename	: 20%-1ml-220nm-30min.lcm		
Batch Filename	: s3-21-6-rac-IA-20%-1ml.lcb		
Vial #	: 1-1	Sample Type	: Unknown
Injection Volume	: 10 uL		
Date Acquired	: 5/10/2023 4:26:22 PM	Acquired by	: System Administrator
Date Processed	: 5/10/2023 5:01:15 PM	Processed by	: System Administrator

<Chromatogram>

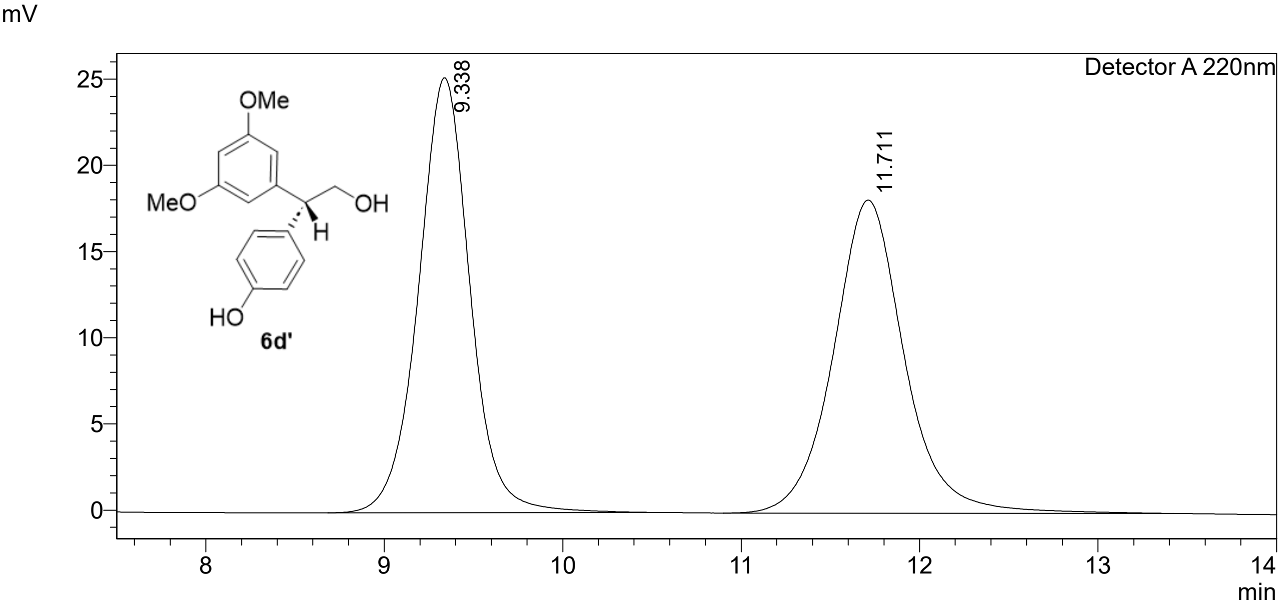

<Peak Table>

Detector A 220nm

Peak#	Ret. Time	Area	Height	Conc.	Unit	Mark	Name
1	9.338	506985	25223	50.186			
2	11.711	503229	18163	49.814			
Total		1010214	43386				

SHIMADZU LabSolutions

Analysis Report

<Sample Information>

Sample Name	:		
Sample ID	:		
Data Filename	: s3-21-6-asy-IA-20%-1ml.lcd		
Method Filename	: 20%-1ml-220nm-30min.lcm		
Batch Filename	: s3-21-6-asy-IA-20%-1ml.lcb		
Vial #	: 1-2	Sample Type	: Unknown
Injection Volume	: 10 uL		
Date Acquired	: 5/10/2023 4:56:46 PM	Acquired by	: System Administrator
Date Processed	: 5/10/2023 5:11:39 PM	Processed by	: System Administrator

<Chromatogram>

mV

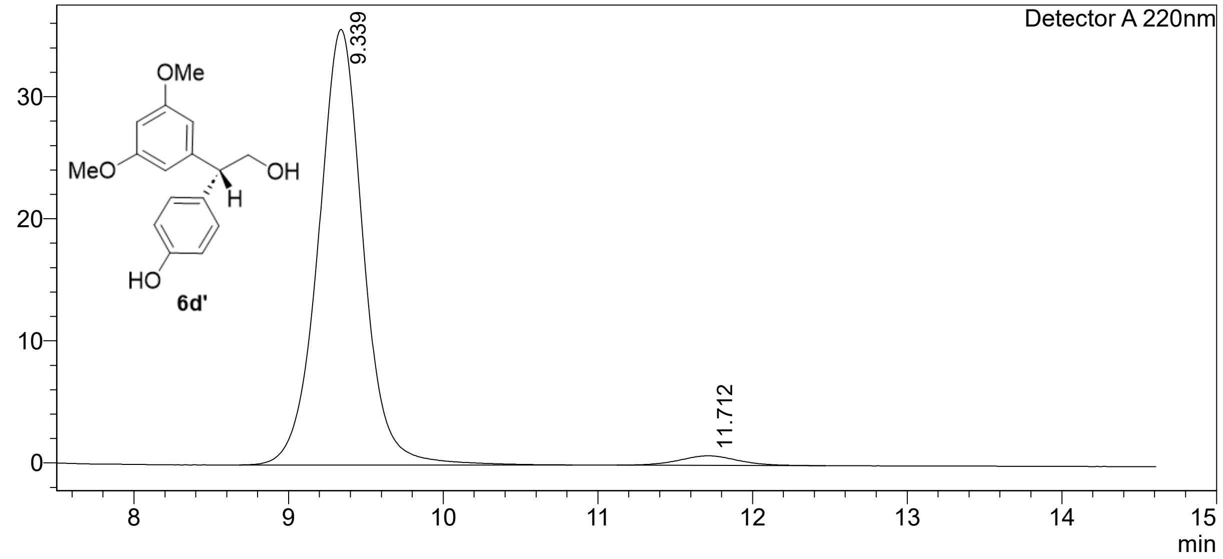

<Peak Table>

Detector A 220nm

Peak#	Ret. Time	Area	Height	Conc.	Unit	Mark	Name
1	9.339	719560	35665	97.195			
2	11.712	20768	786	2.805			
Total		740327	36451				

SHIMADZU LabSolutions

Analysis Report

<Sample Information>

Sample Name	:		
Sample ID	:		
Data Filename	: 368-3-RAC-IE-20%.lcd		
Method Filename	: 20%1ml-220nm-60min.lcm		
Batch Filename	: 368-3-RAC-IE-20%.lcd.lcb		
Vial #	: 1-1	Sample Type	: Unknown
Injection Volume	: 10 uL		
Date Acquired	: 4/21/2023 3:17:20 PM	Acquired by	: System Administrator
Date Processed	: 4/21/2023 3:30:18 PM	Processed by	: System Administrator

<Chromatogram>

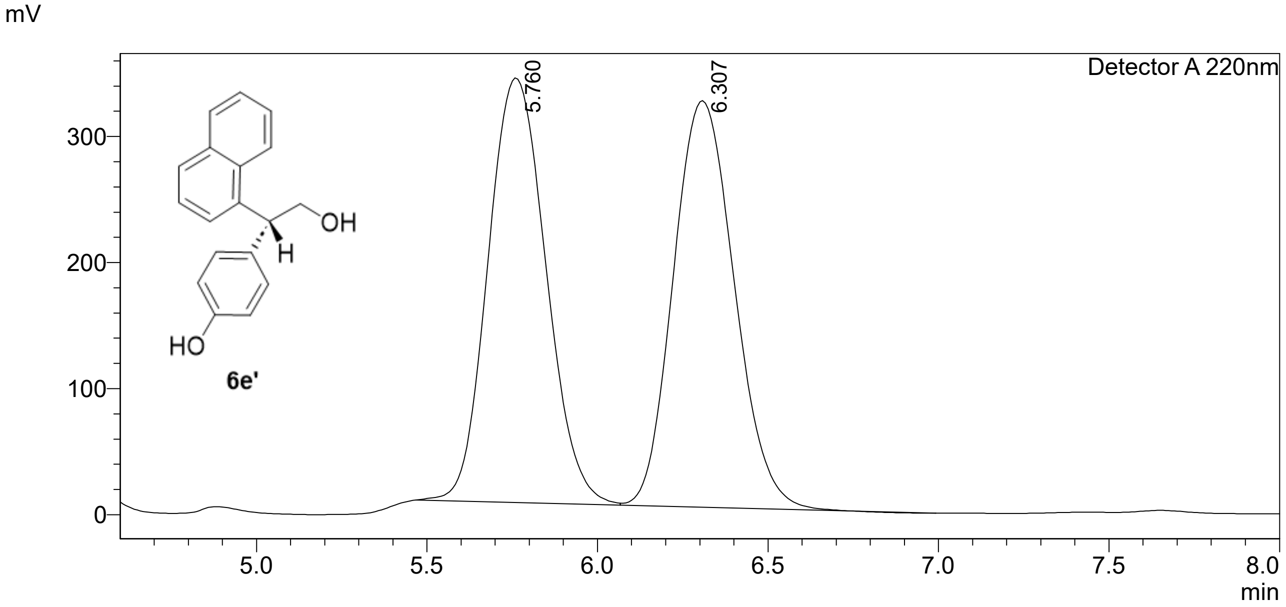

<Peak Table>

Detector A 220nm

Peak#	Ret. Time	Area	Height	Conc.	Unit	Mark	Name
1	5.760	3984193	336762	49.837		M	
2	6.307	4010279	322639	50.163		V M	
Total		7994472	659402				

SHIMADZU LabSolutions

Analysis Report

<Sample Information>

Sample Name	:		
Sample ID	:		
Data Filename	: 368-3-chiral-IE-20%.lcd		
Method Filename	: 20%-1ml-220nm-10min.lcm		
Batch Filename	: 368-3-RAC-IE-20%.lcd.lcb		
Vial #	: 1-2	Sample Type	: Unknown
Injection Volume	: 10 uL		
Date Acquired	: 4/21/2023 3:30:14 PM	Acquired by	: System Administrator
Date Processed	: 5/11/2023 3:41:07 PM	Processed by	: System Administrator

<Chromatogram>

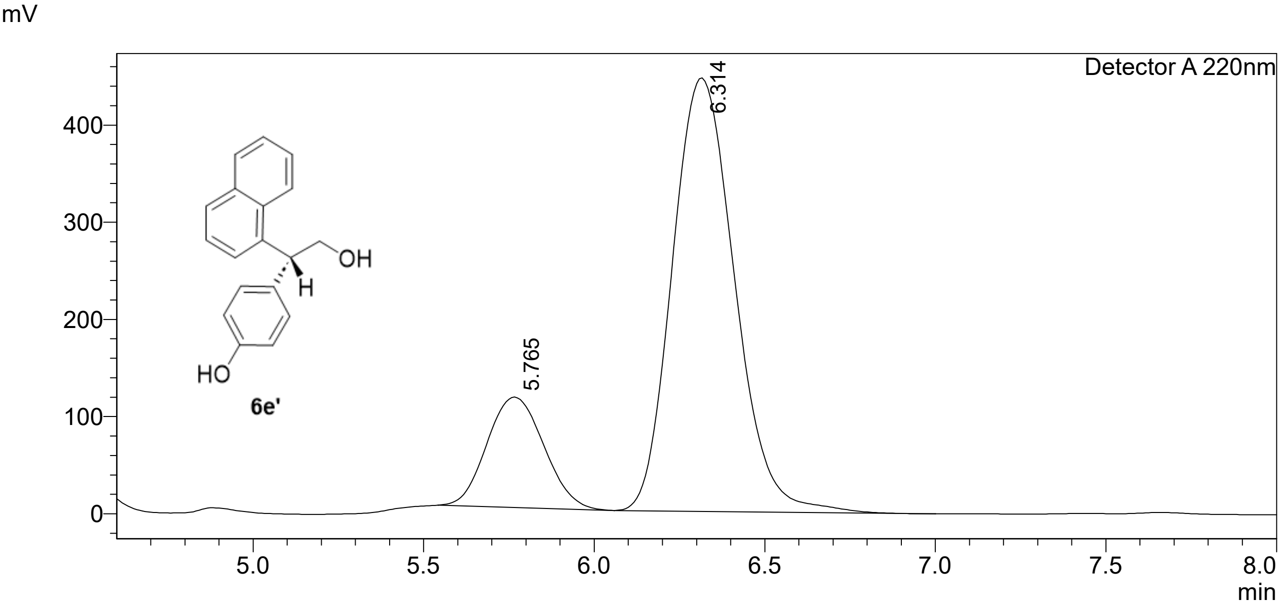

<Peak Table>

Detector A 220nm

Peak#	Ret. Time	Area	Height	Conc.	Unit	Mark	Name
1	5.765	1316405	113560	18.873		M	
2	6.314	5658625	445966	81.127		M	
Total		6975030	559526				

SHIMADZU LabSolutions

Analysis Report

<Sample Information>

Sample Name	:		
Sample ID	:		
Data Filename	: 368-4-RAC-IE-20%.lcd		
Method Filename	: 20%1ml-220nm-60min.lcm		
Batch Filename	: 368-4-RAC-IE-20%.lcd.lcb		
Vial #	: 1-1	Sample Type	: Unknown
Injection Volume	: 10 uL		
Date Acquired	: 4/21/2023 3:40:39 PM	Acquired by	: System Administrator
Date Processed	: 4/21/2023 4:13:21 PM	Processed by	: System Administrator

<Chromatogram>

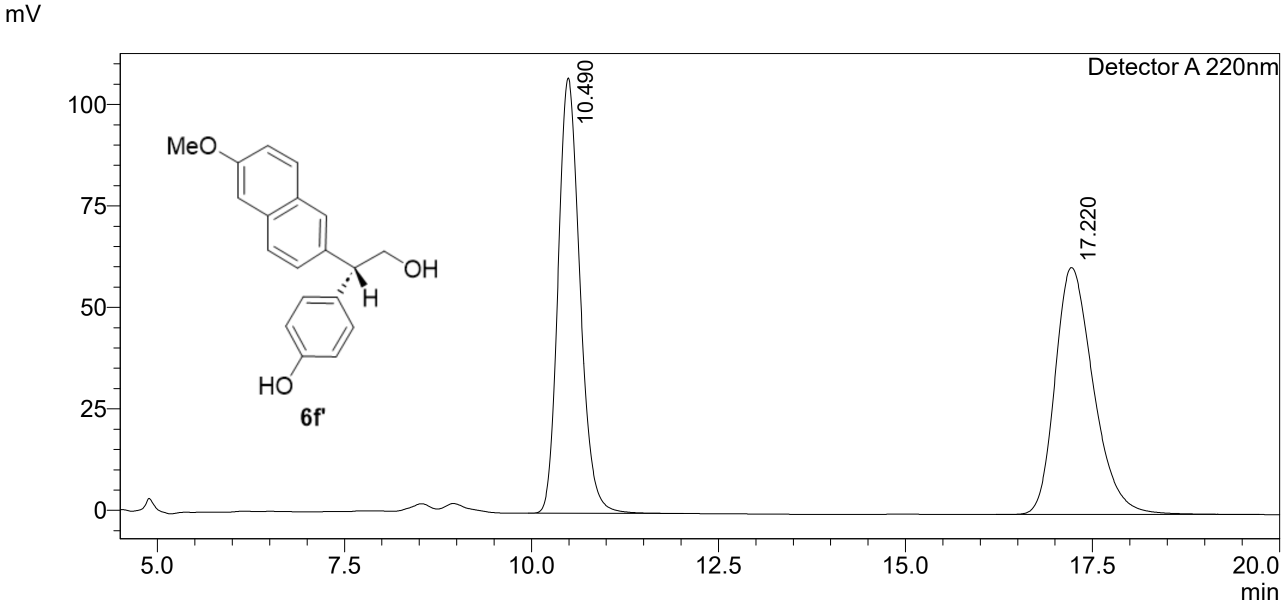

<Peak Table>

Detector A 220nm

Peak#	Ret. Time	Area	Height	Conc.	Unit	Mark	Name
1	10.490	2174138	107230	50.018			
2	17.220	2172585	60854	49.982		S	
Total		4346723	168084				

SHIMADZU LabSolutions

Analysis Report

<Sample Information>

Sample Name	:		
Sample ID	:		
Data Filename	: 368-4-chiral-IE-20%.lcd		
Method Filename	: 20%-1ml-220nm-20min.lcm		
Batch Filename	: 368-4-RAC-IE-20%.lcd.lcb		
Vial #	: 1-2	Sample Type	: Unknown
Injection Volume	: 10 uL		
Date Acquired	: 4/21/2023 4:02:07 PM	Acquired by	: System Administrator
Date Processed	: 5/11/2023 3:42:33 PM	Processed by	: System Administrator

<Chromatogram>

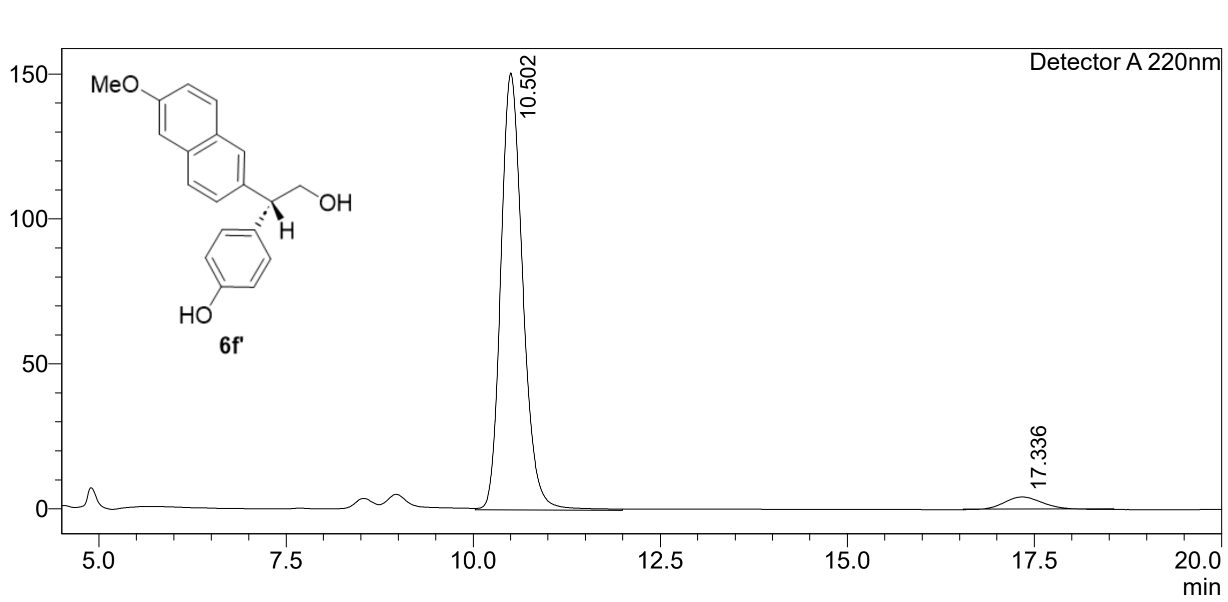

<Peak Table>

Detector A 220nm

Peak#	Ret. Time	Area	Height	Conc.	Unit	Mark	Name
1	10.502	3076745	150745	95.796		M	
2	17.336	135023	4165	4.204		M	
Total		3211768	154911				

SHIMADZU LabSolutions

Analysis Report

<Sample Information>

Sample Name	:		
Sample ID	:		
Data Filename	: s3-21-1-rac-IA-20%-1ml-re.lcd		
Method Filename	: 20%-1ml-220nm-40min.lcm		
Batch Filename	: s3-21-1-asy-IA-20%-1m.lcb		
Vial #	: 1-1	Sample Type	: Unknown
Injection Volume	: 10 uL		
Date Acquired	: 5/10/2023 5:11:49 PM	Acquired by	: System Administrator
Date Processed	: 5/10/2023 6:06:35 PM	Processed by	: System Administrator

<Chromatogram>

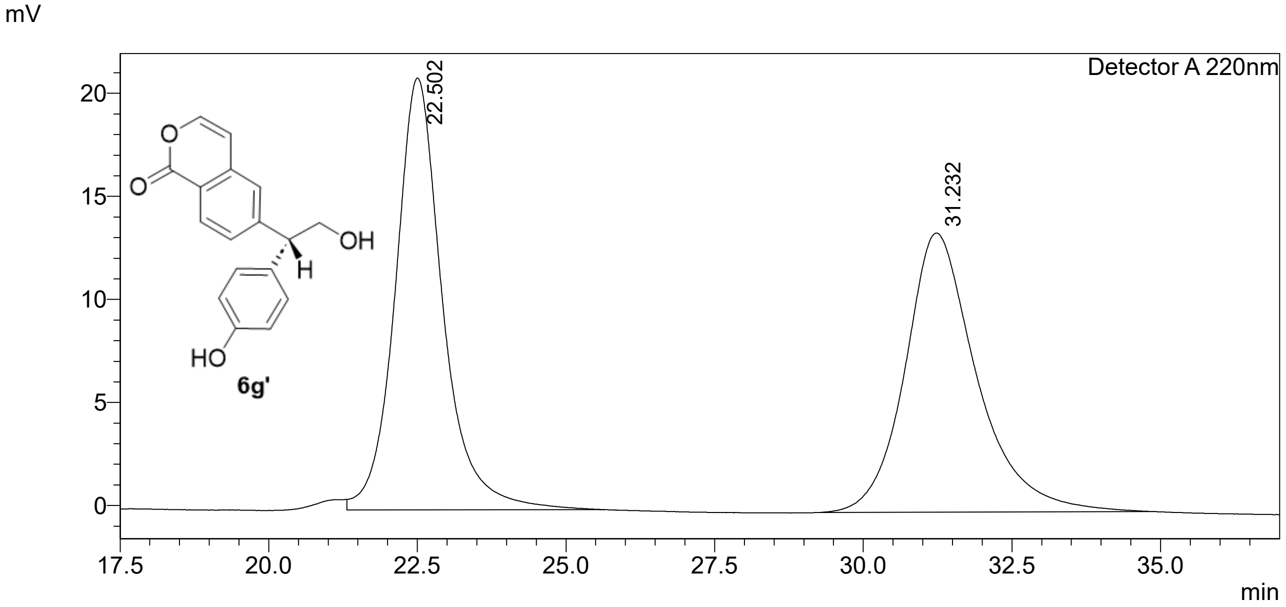

<Peak Table>

Detector A 220nm

Peak#	Ret. Time	Area	Height	Conc.	Unit	Mark	Name
1	22.502	1165634	20961	50.580			
2	31.232	1138881	13547	49.420			
Total		2304515	34508				

SHIMADZU LabSolutions

Analysis Report

<Sample Information>

Sample Name	:		
Sample ID	:		
Data Filename	: s3-21-1-asy-IA-20%-1ml.lcd		
Method Filename	: 20%-1ml-220nm-40min.lcm		
Batch Filename	: s3-21-1-asy-IA-20%-1m.lcb		
Vial #	: 1-2	Sample Type	: Unknown
Injection Volume	: 10 uL		
Date Acquired	: 5/10/2023 3:45:55 PM	Acquired by	: System Administrator
Date Processed	: 5/10/2023 5:01:29 PM	Processed by	: System Administrator

<Chromatogram>

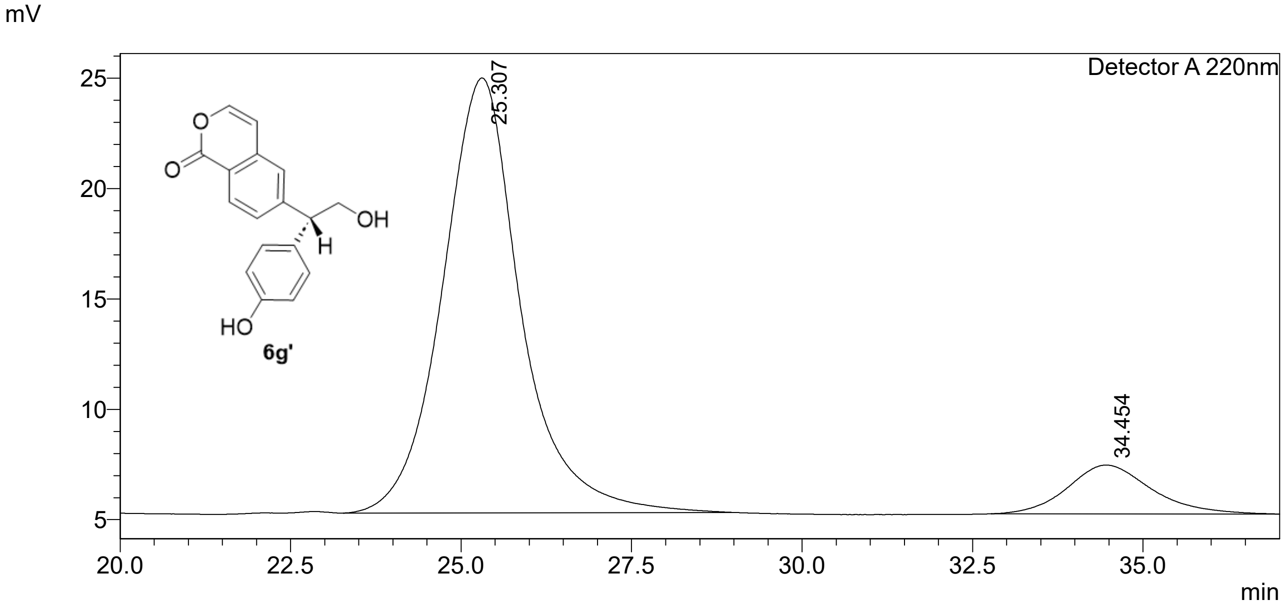

<Peak Table>

Detector A 220nm

Peak#	Ret. Time	Area	Height	Conc.	Unit	Mark	Name
1	25.307	1569901	19699	89.219			
2	34.454	189705	2213	10.781		S	
Total		1759607	21913				

SHIMADZU LabSolutions

Analysis Report

<Sample Information>

Sample Name	:		
Sample ID	:		
Data Filename	: 414-rac-40%-ie-1ML.lcd		
Method Filename	: 40%1ml-220nm-10min.lcm		
Batch Filename	: 414-1.lcb		
Vial #	: 1-1	Sample Type	: Unknown
Injection Volume	: 10 uL		
Date Acquired	: 8/4/2023 2:52:57 PM	Acquired by	: System Administrator
Date Processed	: 8/4/2023 3:04:50 PM	Processed by	: System Administrator

<Chromatogram>

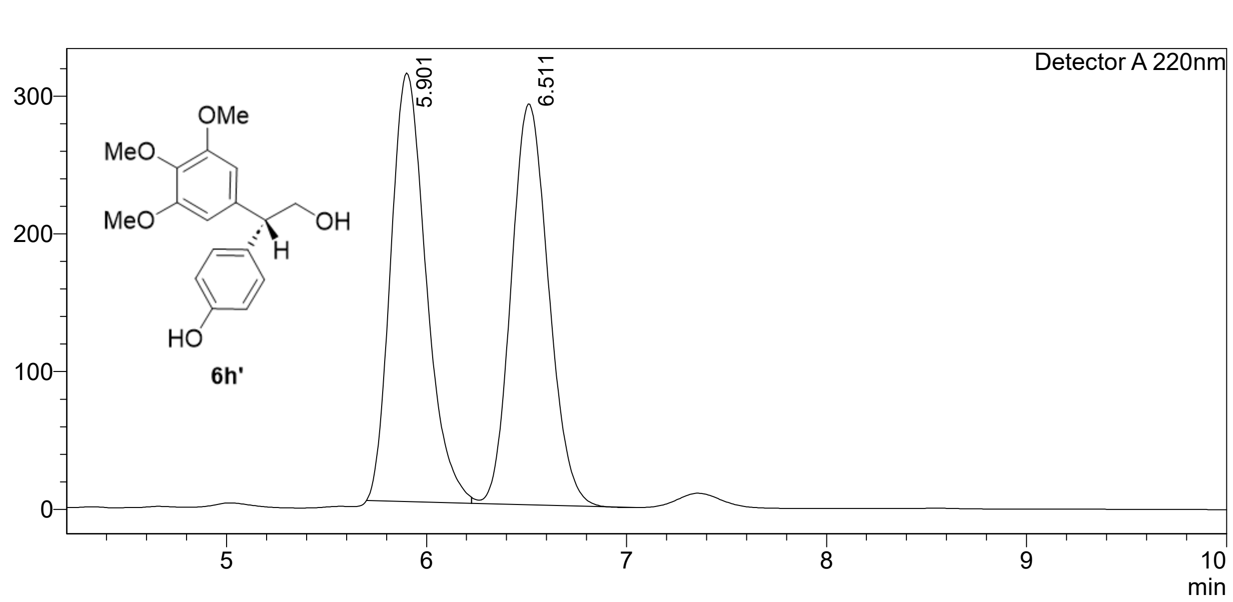

<Peak Table>

Detector A 220nm

Peak#	Ret. Time	Area	Height	Conc.	Unit	Mark	Name
1	5.901	3828203	311269	50.271		M	
2	6.511	3786965	291215	49.729		V M	
Total		7615168	602484				

SHIMADZU LabSolutions

Analysis Report

<Sample Information>

Sample Name	:		
Sample ID	:		
Data Filename	: 414-1-CHIRAL-40%-ie-1ML.lcd		
Method Filename	: 40%1ml-220nm-10min.lcm		
Batch Filename	: 414-1.lcb		
Vial #	: 1-2	Sample Type	: Unknown
Injection Volume	: 10 uL		
Date Acquired	: 8/4/2023 3:03:21 PM	Acquired by	: System Administrator
Date Processed	: 8/4/2023 3:30:53 PM	Processed by	: System Administrator

<Chromatogram>

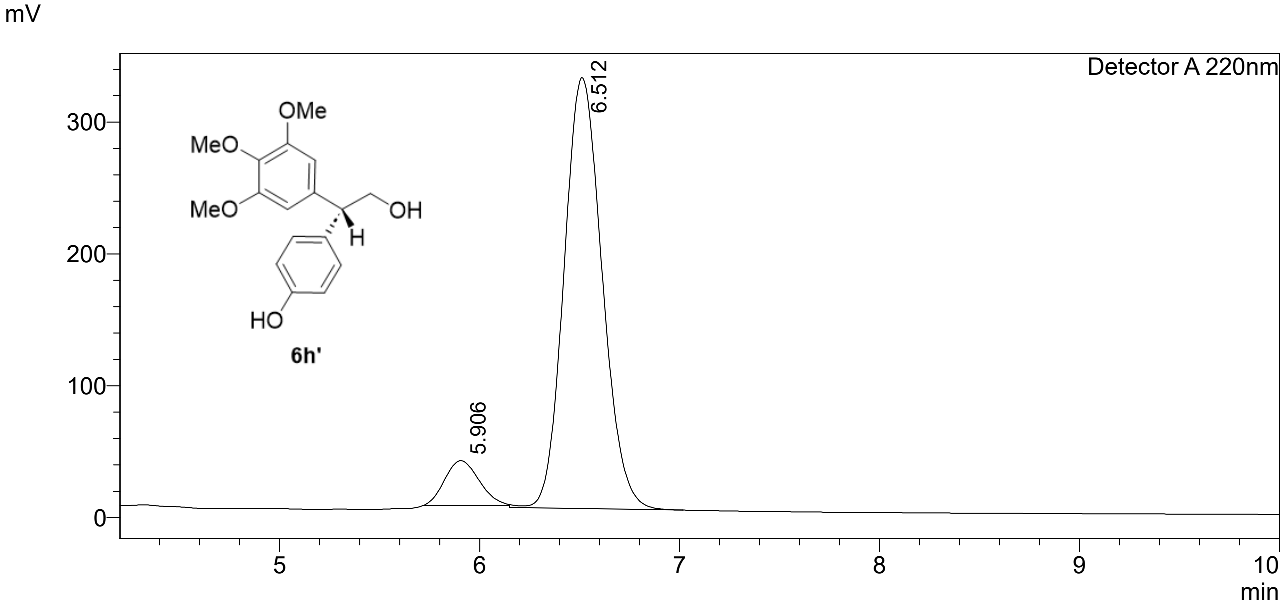

<Peak Table>

Detector A 220nm

Peak#	Ret. Time	Area	Height	Conc.	Unit	Mark	Name
1	5.906	409790	34068	8.631		M	
2	6.512	4338092	326684	91.369		M	
Total		4747882	360752				

SHIMADZU LabSolutions

Analysis Report

<Sample Information>

Sample Name	:		
Sample ID	:		
Data Filename	: s3-30-3-rac-IE-10%-1ml004.lcd		
Method Filename	: 10%1ml-220nm-40min.lcm		
Batch Filename	: 30-1 30-3.lcb		
Vial #	: 1-16	Sample Type	: Unknown
Injection Volume	: 10 uL		
Date Acquired	: 7/25/2023 11:39:00 PM	Acquired by	: System Administrator
Date Processed	: 7/26/2023 10:32:10 AM	Processed by	: System Administrator

<Chromatogram>

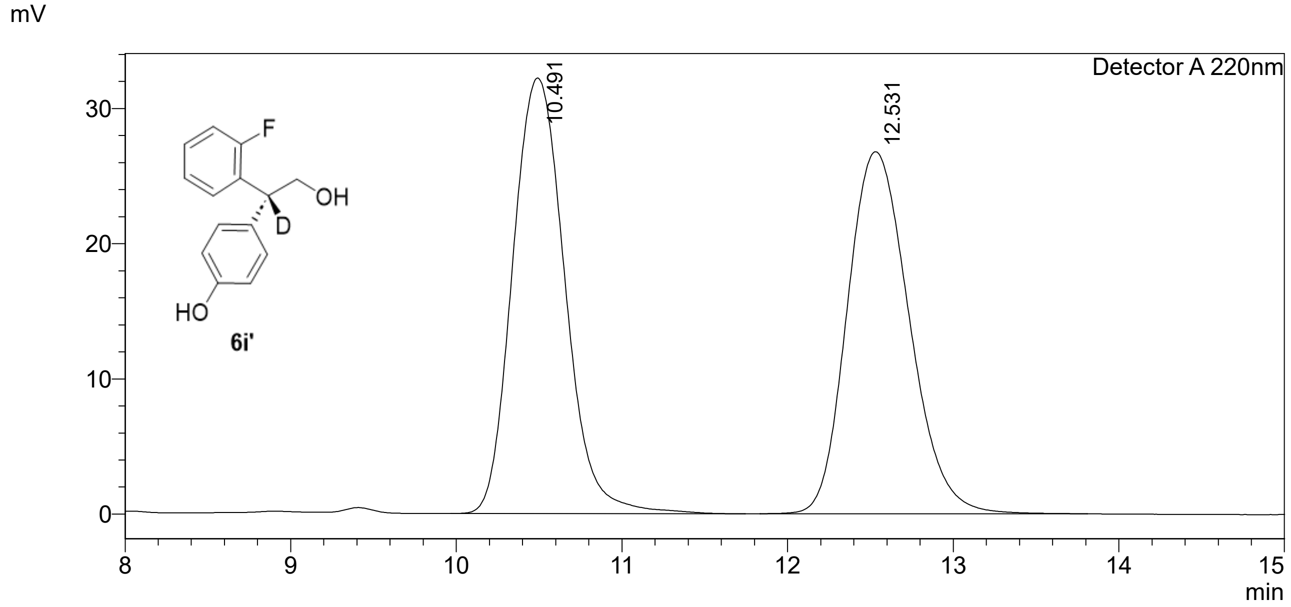

<Peak Table>

Detector A 220nm

Peak#	Ret. Time	Area	Height	Conc.	Unit	Mark	Name
1	10.491	708593	32222	50.443			
2	12.531	696158	26788	49.557			
Total		1404751	59010				

SHIMADZU LabSolutions

Analysis Report

<Sample Information>

Sample Name	:		
Sample ID	:		
Data Filename	: s3-30-3-asy-IE-10%-1ml005.lcd		
Method Filename	: 10%1ml-220nm-40min.lcm		
Batch Filename	: 30-1 30-3.lcb		
Vial #	: 1-17	Sample Type	: Unknown
Injection Volume	: 10 uL		
Date Acquired	: 7/26/2023 12:19:24 AM	Acquired by	: System Administrator
Date Processed	: 7/26/2023 10:32:18 AM	Processed by	: System Administrator

<Chromatogram>

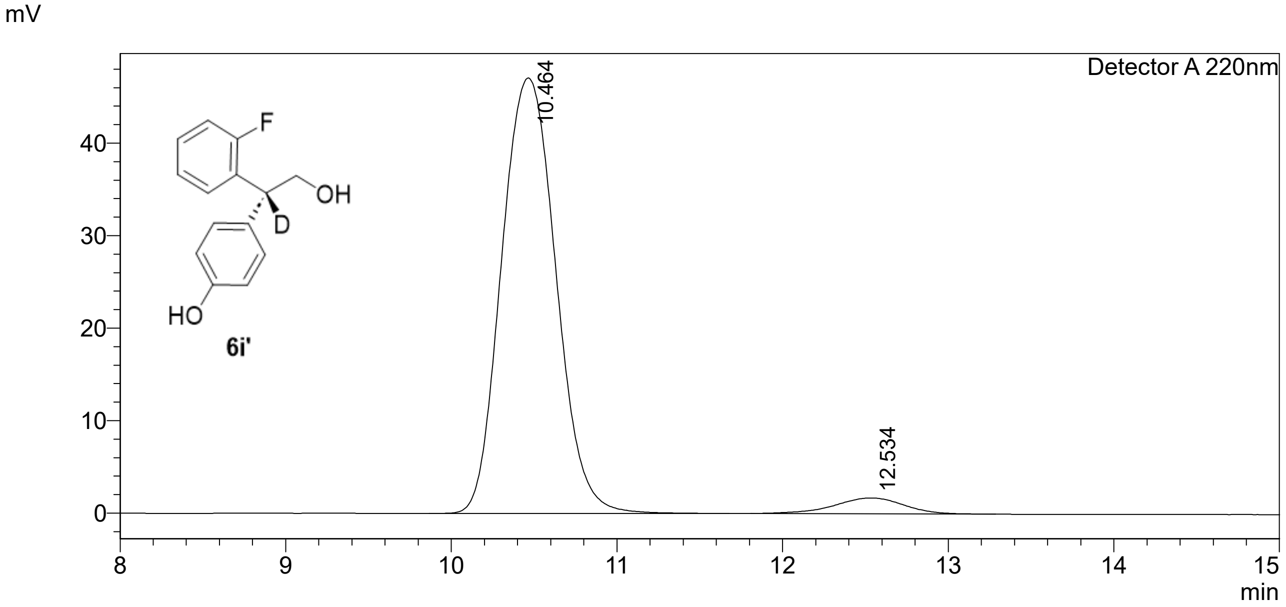

<Peak Table>

Detector A 220nm

Peak#	Ret. Time	Area	Height	Conc.	Unit	Mark	Name
1	10.464	1073554	47086	95.537			
2	12.534	50151	1711	4.463			
Total		1123705	48797				

SHIMADZU LabSolutions

Analysis Report

<Sample Information>

Sample Name	:		
Sample ID	:		
Data Filename	: S3-39-1-RAC-RE-IE-10%-1ML.lcd		
Method Filename	: 10%1ml-220nm-40min.lcm		
Batch Filename	: S3-22-3-RAC-RE-IE-10%-1ML.lcb		
Vial #	: 1-1	Sample Type	: Unknown
Injection Volume	: 10 uL		
Date Acquired	: 7/25/2023 4:11:36 PM	Acquired by	: System Administrator
Date Processed	: 10/4/2023 10:06:55 AM	Processed by	: System Administrator

<Chromatogram>

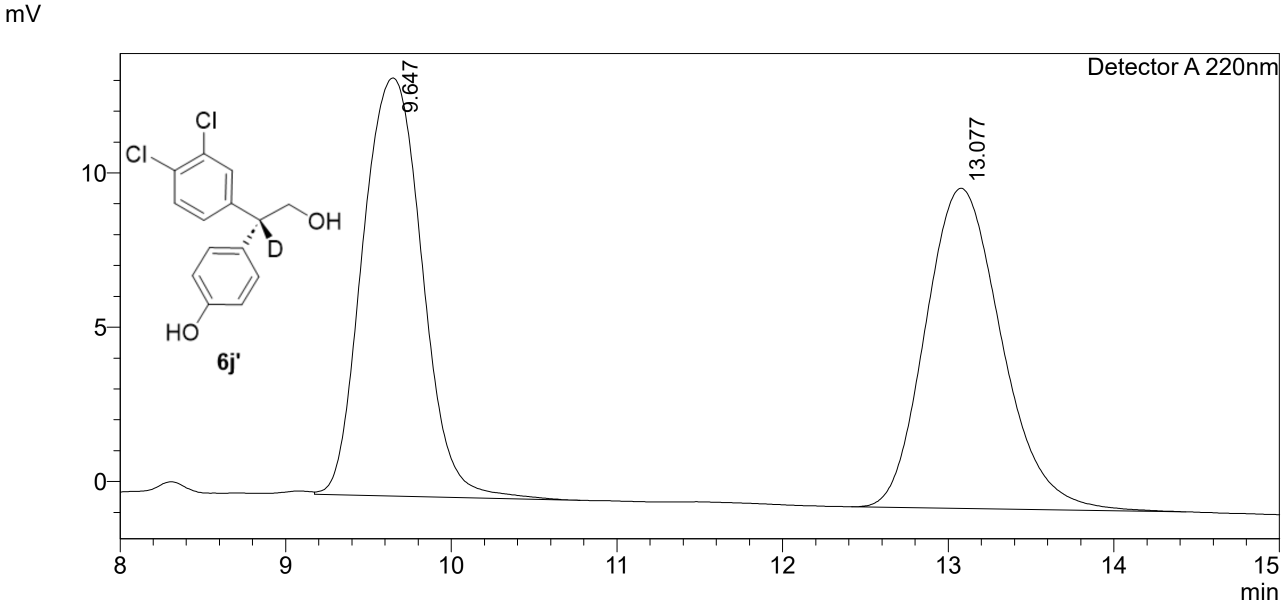

<Peak Table>

Detector A 220nm

Peak#	Ret. Time	Area	Height	Conc.	Unit	Mark	Name
1	9.647	338529	13547	50.529			
2	13.077	331435	10373	49.471			
Total		669964	23920				

SHIMADZU LabSolutions

Analysis Report

<Sample Information>

Sample Name	:		
Sample ID	:		
Data Filename	: s3-1,2-Cl-re-asy-IE-10%-1ml.lcd		
Method Filename	: 10%1ml-220nm-20min.lcm		
Batch Filename	: s3-1,2-Cl-re-asy-IE-10%-1ml.lcb		
Vial #	: 1-2	Sample Type	: Unknown
Injection Volume	: 10 uL		
Date Acquired	: 8/11/2023 11:20:20 AM	Acquired by	: System Administrator
Date Processed	: 10/6/2023 6:41:47 PM	Processed by	: System Administrator

<Chromatogram>

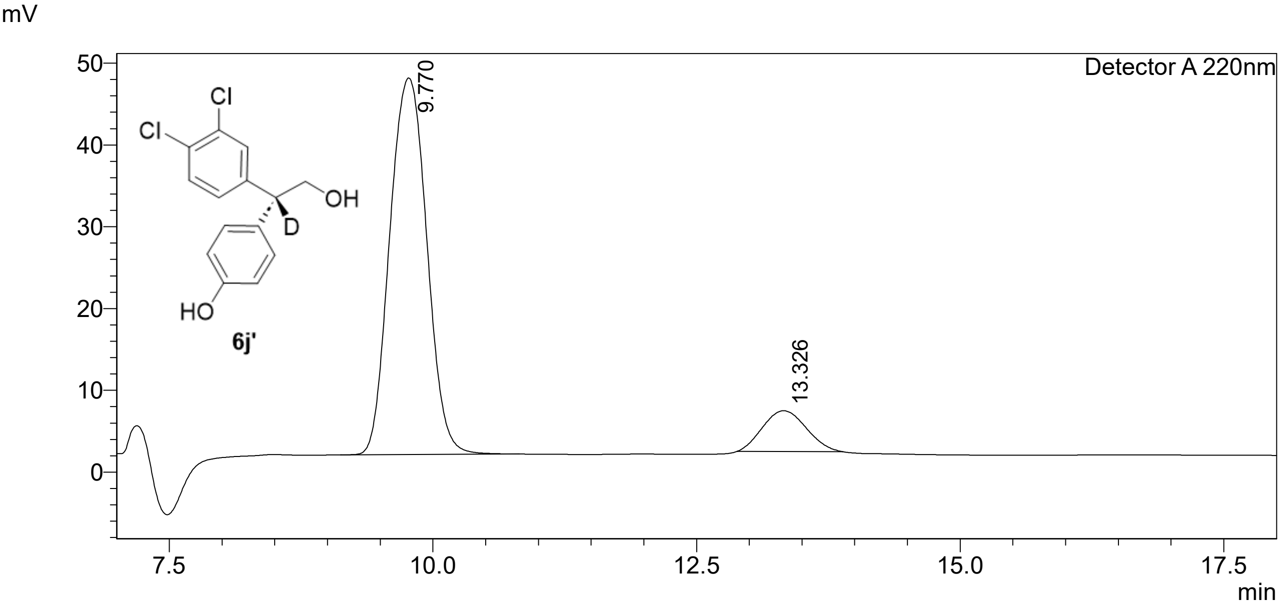

<Peak Table>

Detector A 220nm

Peak#	Ret. Time	Area	Height	Conc.	Unit	Mark	Name
1	9.770	1126904	46052	88.653			
2	13.326	144241	4984	11.347		M	
Total		1271144	51036				

SHIMADZU LabSolutions

Analysis Report

<Sample Information>

Sample Name	:		
Sample ID	:		
Data Filename	: 364-rac-1ml-5%-ia.lcd		
Method Filename	: 5%-1ml-220nm-60min.lcm		
Batch Filename	: 364-rac-1ml-5%-ia.lcd.lcb		
Vial #	: 1-1	Sample Type	: Unknown
Injection Volume	: 10 uL		
Date Acquired	: 4/12/2023 4:44:57 PM	Acquired by	: System Administrator
Date Processed	: 4/13/2023 10:32:06 AM	Processed by	: System Administrator

<Chromatogram>

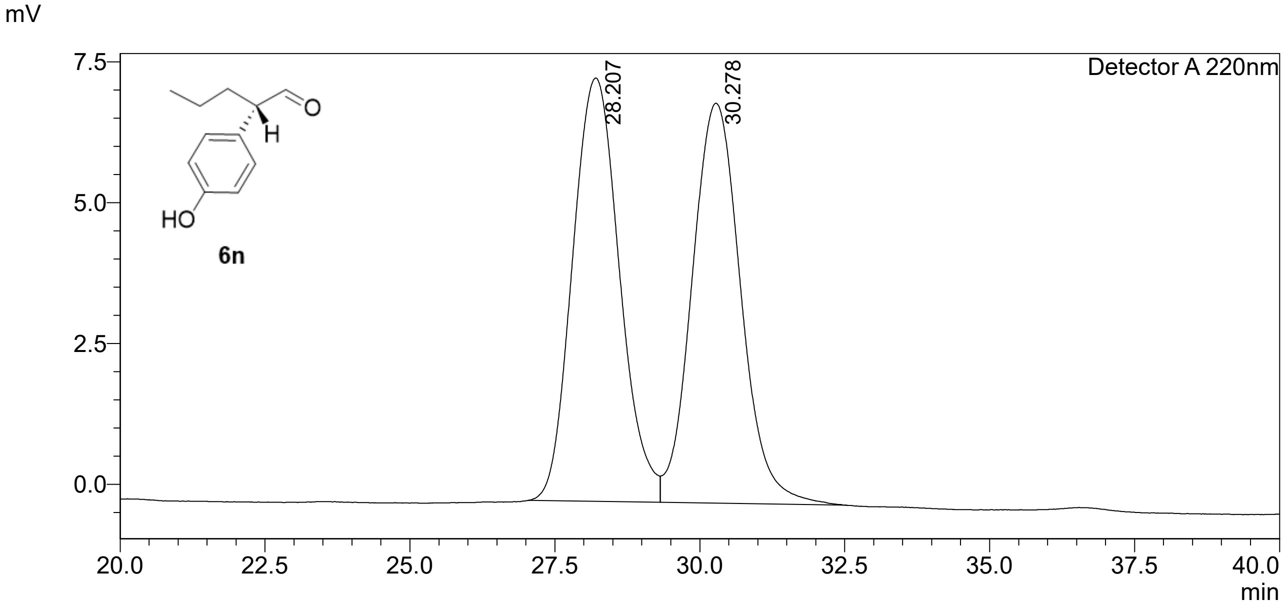

<Peak Table>

Detector A 220nm

Peak#	Ret. Time	Area	Height	Conc.	Unit	Mark	Name
1	28.207	424316	7519	50.481			
2	30.278	416226	7103	49.519		V	
Total		840541	14622				

SHIMADZU LabSolutions

Analysis Report

<Sample Information>

Sample Name	:		
Sample ID	:		
Data Filename	: 364-4-chiral-1ml-5%-ia.lcd		
Method Filename	: 5%-1ml-220nm-40min.lcm		
Batch Filename	: 364-4-chiral-1ml-5%-ia.lcd.lcb		
Vial #	: 1-4	Sample Type	: Unknown
Injection Volume	: 10 uL		
Date Acquired	: 4/12/2023 10:03:38 PM	Acquired by	: System Administrator
Date Processed	: 4/13/2023 10:33:53 AM	Processed by	: System Administrator

<Chromatogram>

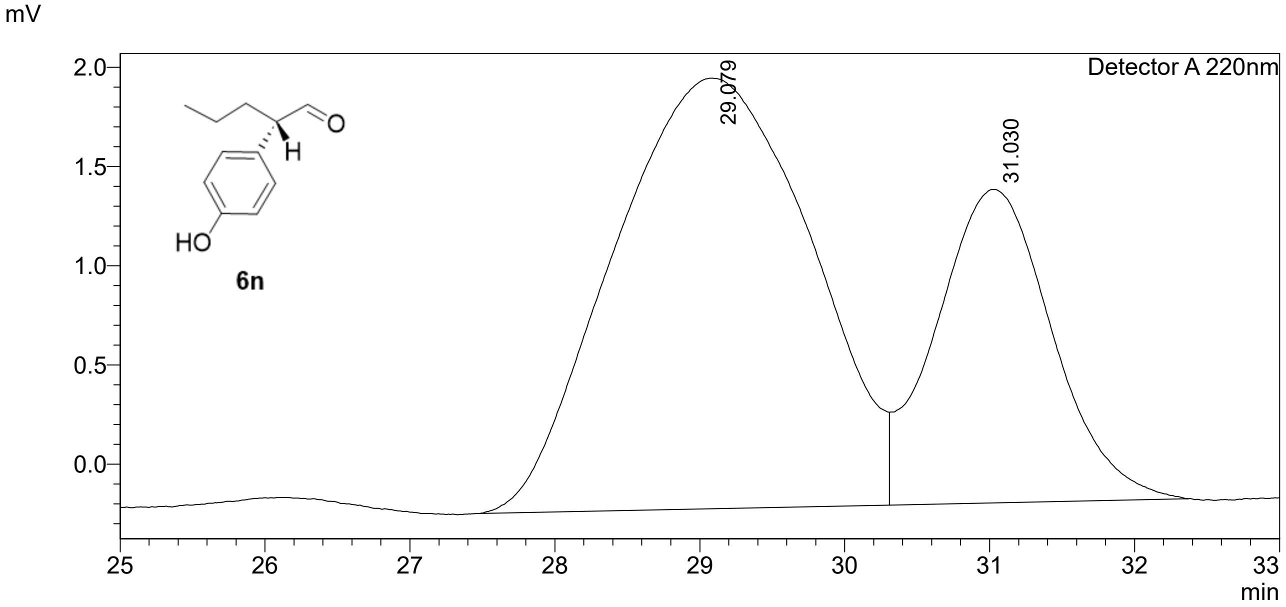

<Peak Table>

Detector A 220nm

Peak#	Ret. Time	Area	Height	Conc.	Unit	Mark	Name
1	29.079	204795	2171	69.921			
2	31.030	88099	1580	30.079		V	
Total		292895	3750				

SHIMADZU LabSolutions

Analysis Report

<Sample Information>

Sample Name	:		
Sample ID	:		
Data Filename	: 0122-10%-1ml-IA-RAC.lcd		
Method Filename	: WMY-20%-220-60mins.lcm		
Batch Filename	: 0122-10%-1ml-IA-RAC.lcb		
Vial #	: 1-1	Sample Type	: Unknown
Injection Volume	: 10 uL		
Date Acquired	: 17/1/2024 4:22:30 PM	Acquired by	: System Administrator
Date Processed	: 17/1/2024 4:32:53 PM	Processed by	: System Administrator

<Chromatogram>

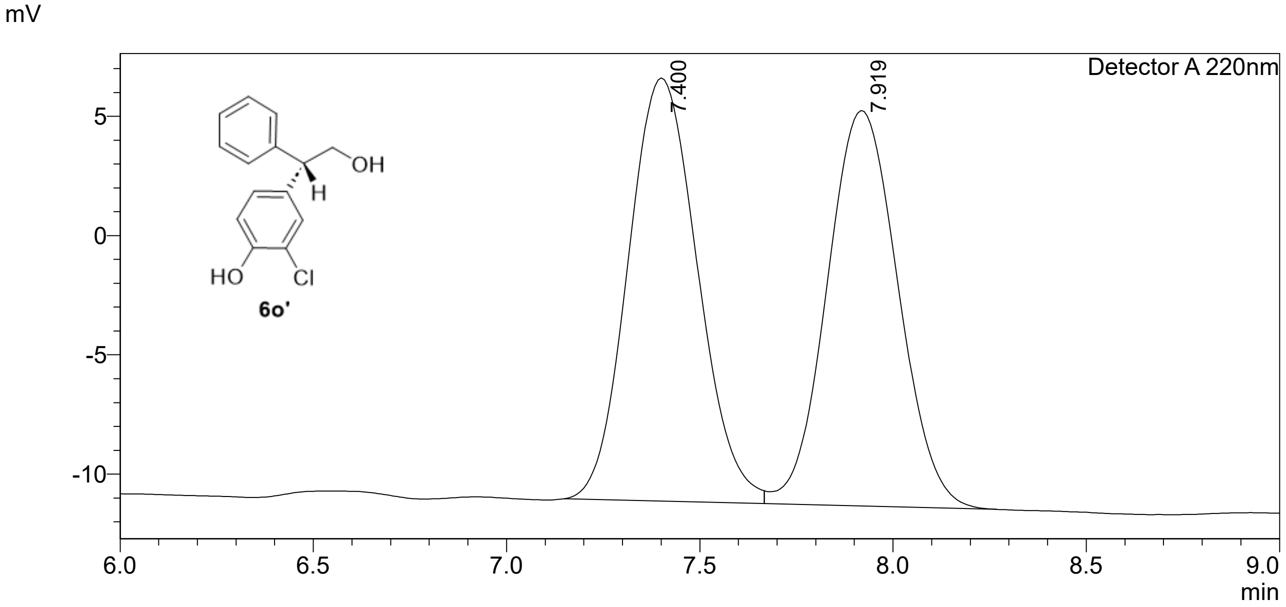

<Peak Table>

Detector A 220nm

Peak#	Ret. Time	Area	Height	Conc.	Unit	Mark	Name
1	7.400	221568	17735	51.105		M	
2	7.919	211984	16575	48.895		V M	
Total		433552	34310				

SHIMADZU LabSolutions

Analysis Report

<Sample Information>

Sample Name	:		
Sample ID	:		
Data Filename	: 0122-10%-1ml-IA-ASY.lcd		
Method Filename	: WMY-20%-220-60mins.lcm		
Batch Filename	: 15341.lcb		
Vial #	: 1-1	Sample Type	: Unknown
Injection Volume	: 10 uL		
Date Acquired	: 17/1/2024 4:33:18 PM	Acquired by	: System Administrator
Date Processed	: 17/1/2024 4:42:26 PM	Processed by	: System Administrator

<Chromatogram>

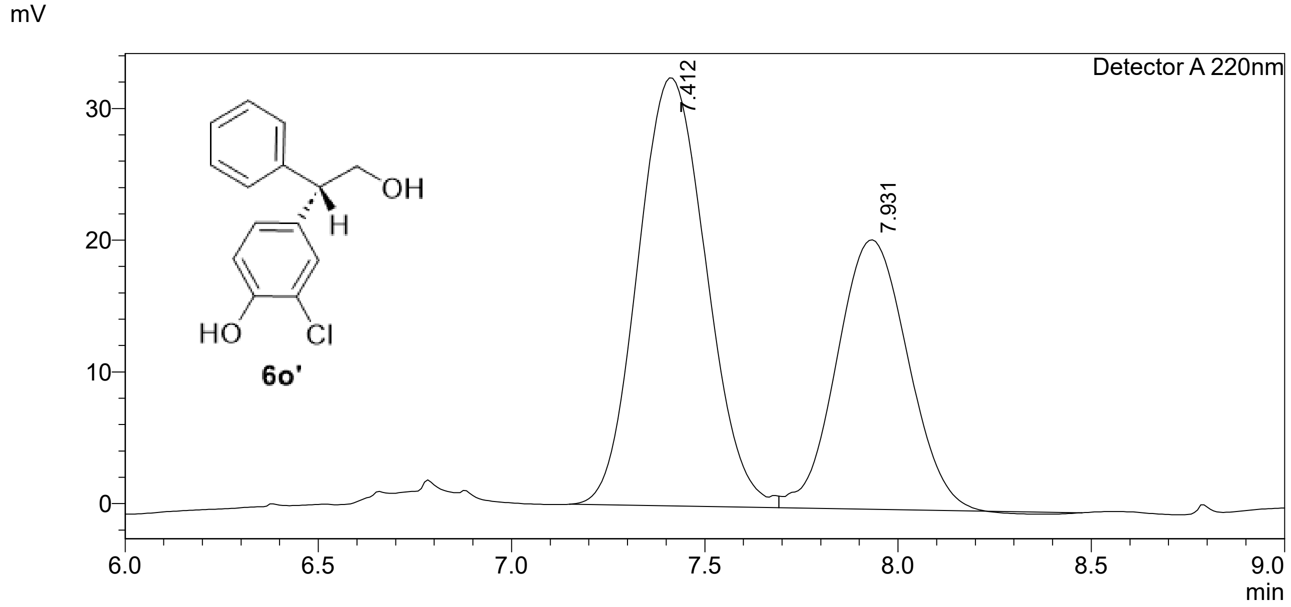

<Peak Table>

Detector A 220nm

Peak#	Ret. Time	Area	Height	Conc.	Unit	Mark	Name
1	7.412	400439	32495	60.625		M	
2	7.931	260080	20465	39.375		V M	
Total		660519	52960				

9. Copies of NMR spectra

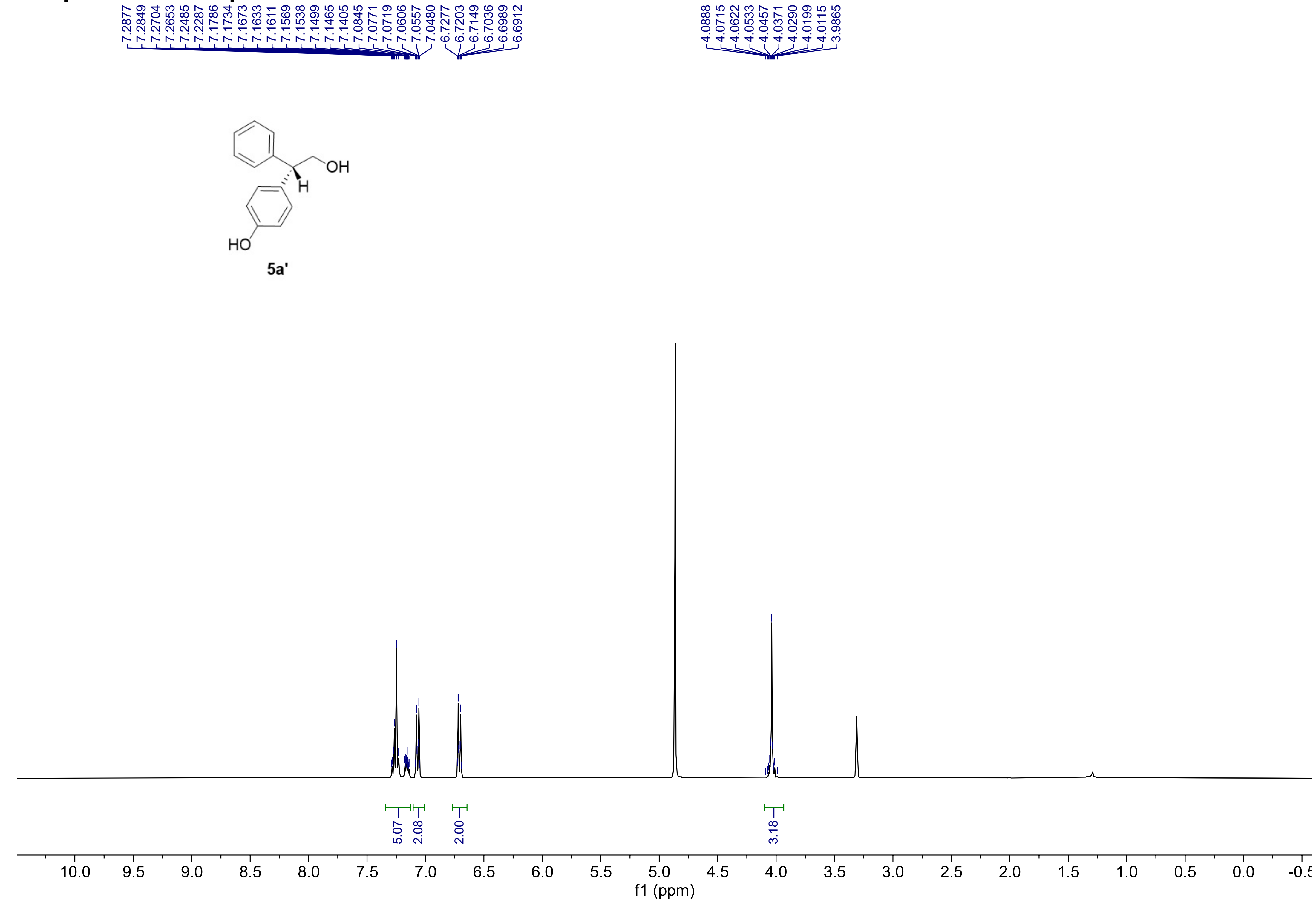

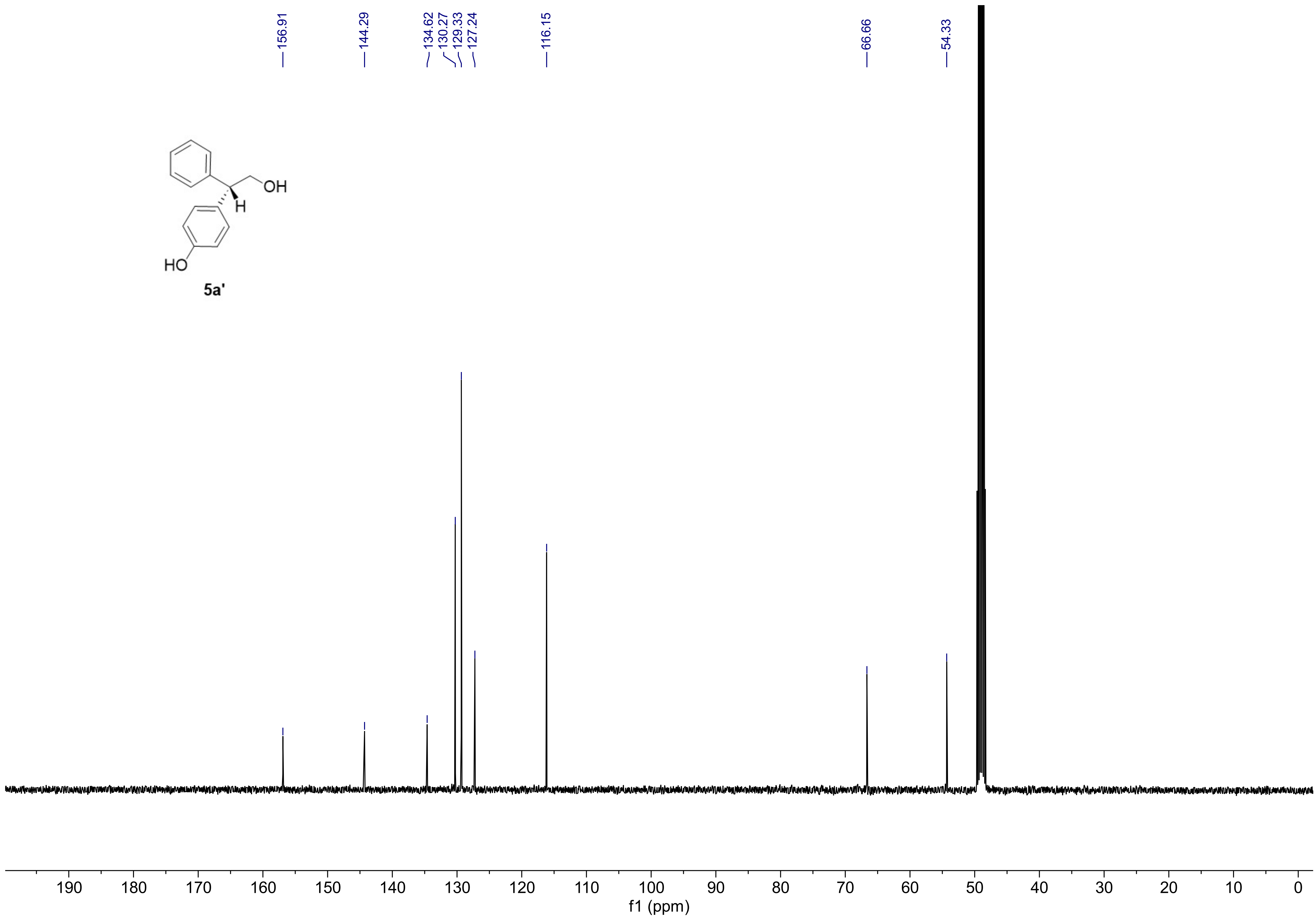

156.91
144.29
134.62
130.27
129.33
127.24
116.15
66.66
54.33
OH
H
HO
5a'
190
180
170
160
150
140
130
120
110
100
90
80
70
60
50
40
30
20
10
0
f1 (ppm)

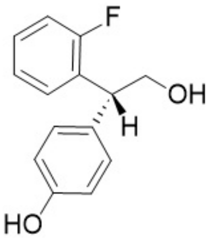
F
OH
H
HO

5b'

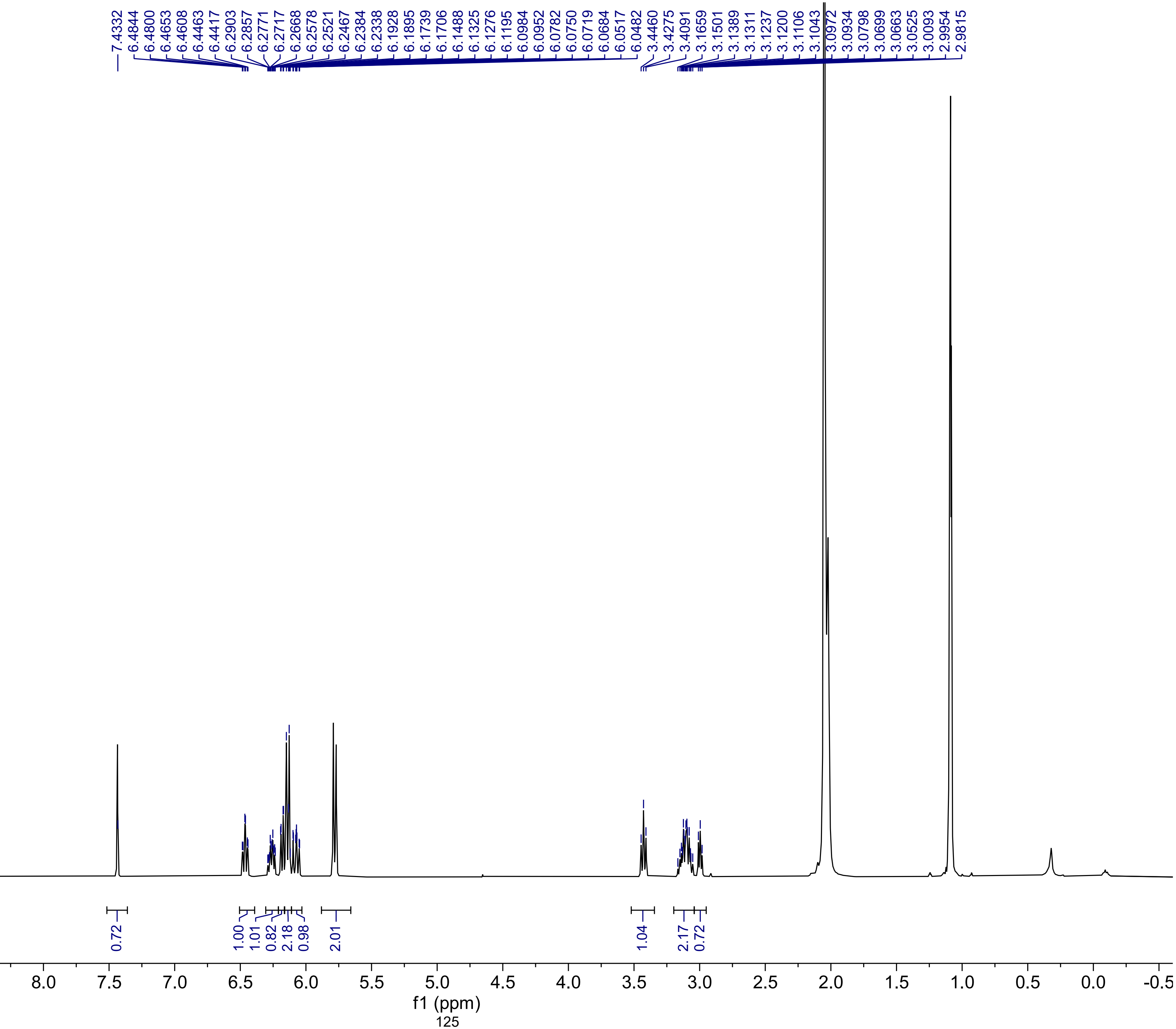
7.4332
6.4844
6.4800
6.4653
6.4608
6.4463
6.4417
6.2903
6.2857
6.2771
6.2717
6.2668
6.2578
6.2521
6.2467
6.2384
6.2338
6.1928
6.1895
6.1739
6.1706
6.1488
6.1325
6.1276
6.1195
6.0984
6.0952
6.0782
6.0750
6.0719
6.0684
6.0517
6.0482
3.4460
3.4275
3.4091
3.1659
3.1501
3.1389
3.1311
3.1237
3.1200
3.1106
3.1043
3.0972
3.0934
3.0798
3.0699
3.0663
3.0525
3.0093
2.9954
2.9815
0.72
1.00
1.01
0.82
2.18
0.98
2.01
1.04
2.17
0.72
10.0
9.5
9.0
8.5
8.0
7.5
7.0
6.5
6.0
5.5
5.0
4.5
4.0
3.5
3.0
2.5
2.0
1.5
1.0
0.5
0.0
-0.5
f1 (ppm)

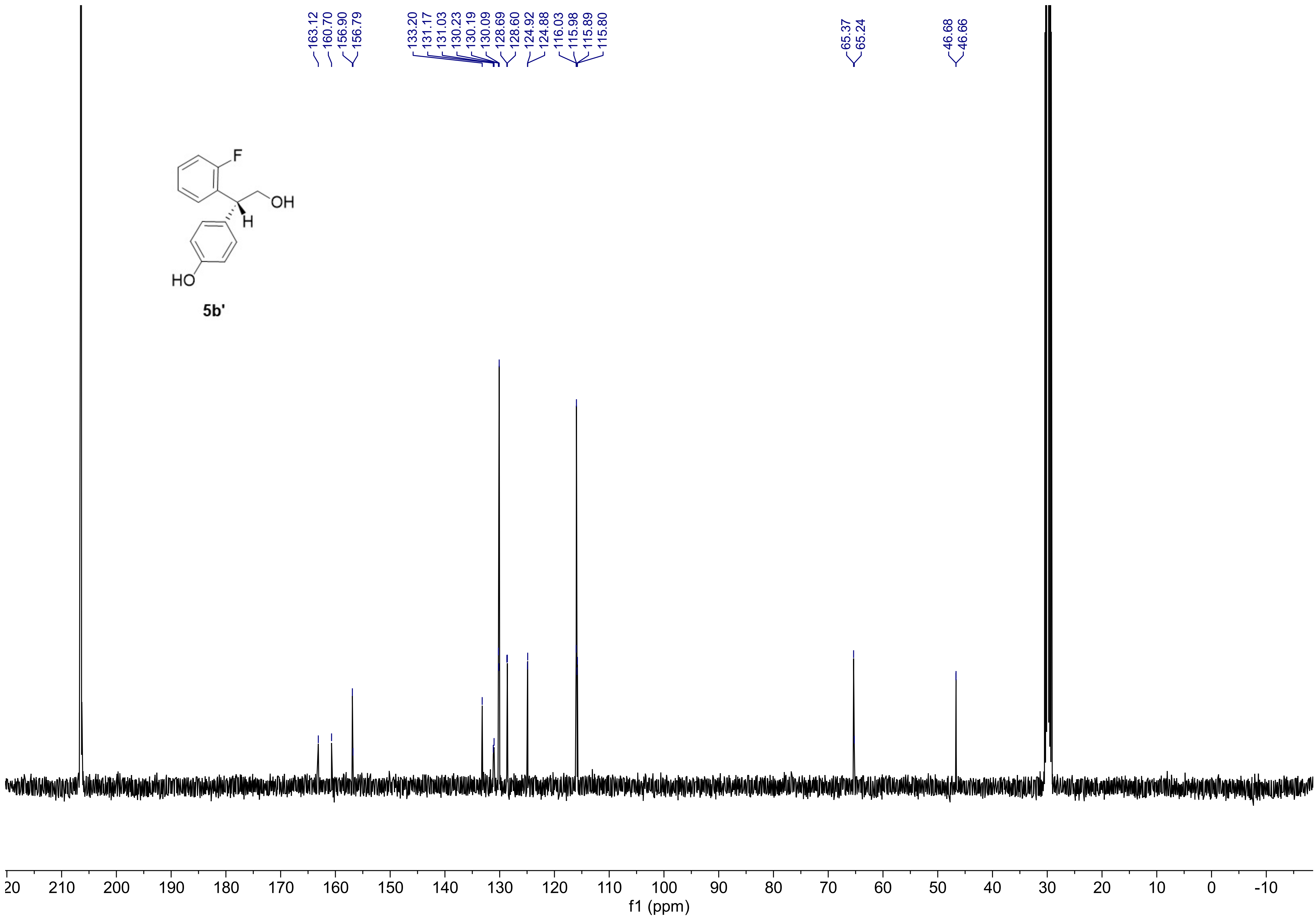
163.12
160.70
156.90
156.79
133.20
131.17
131.03
130.23
130.19
130.09
128.69
128.60
124.92
124.88
116.03
115.98
115.89
115.80
65.37
65.24
46.68
46.66
F
OH
H
HO
5b'
f1 (ppm)

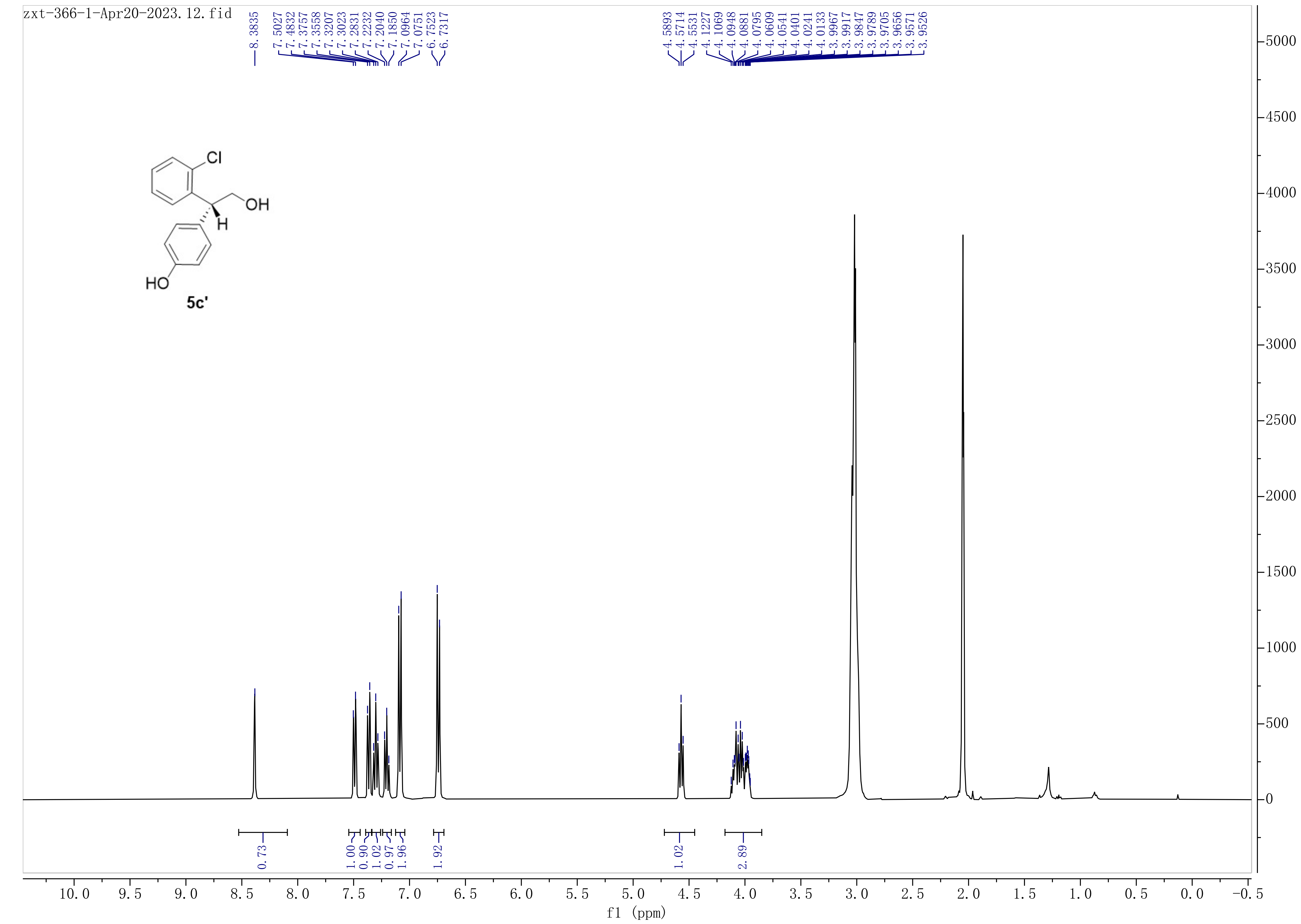
zxt-366-1-Apr20-2023.12.fid
8.3835
7.5027
7.4832
7.3757
7.3558
7.3207
7.3023
7.2831
7.2232
7.2040
7.1850
7.0964
7.0751
6.7523
6.7317
4.5893
4.5714
4.5531
4.1227
4.1069
4.0948
4.0881
4.0795
4.0609
4.0541
4.0401
4.0241
4.0133
3.9967
3.9917
3.9847
3.9789
3.9705
3.9656
3.9571
3.9526
Cl
OH
H
HO
5c'
0.73
1.00
0.90
1.02
0.97
1.96
1.92
1.02
2.89
f1 (ppm)

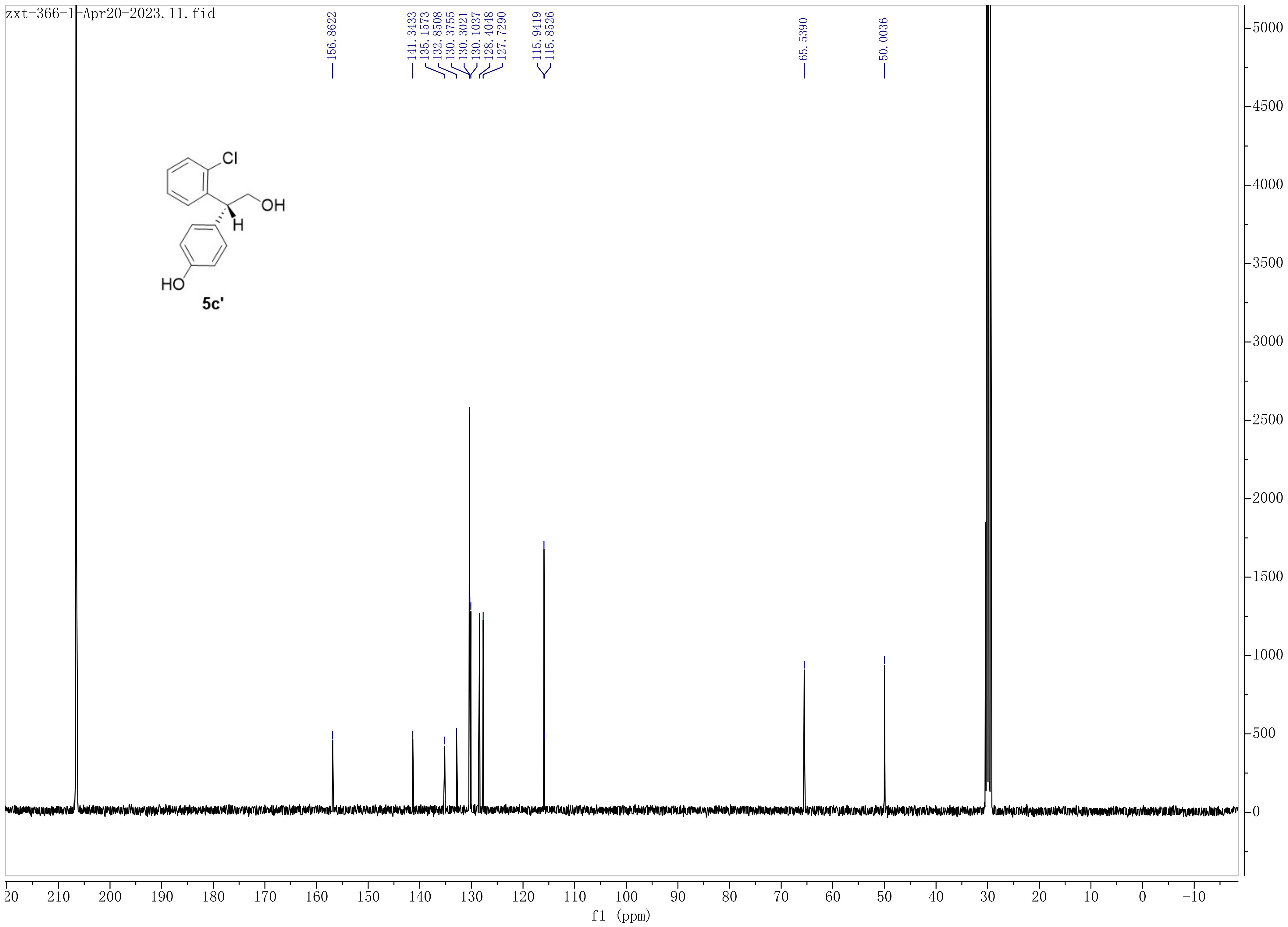
zxt-366-1-Apr20-2023.11.fid
156.8622
141.3433
135.1573
132.8508
130.3755
130.3021
130.1037
128.4048
127.7290
115.9419
115.8526
65.5390
50.0036
Cl
OH
H
HO
5c'
f1 (ppm)

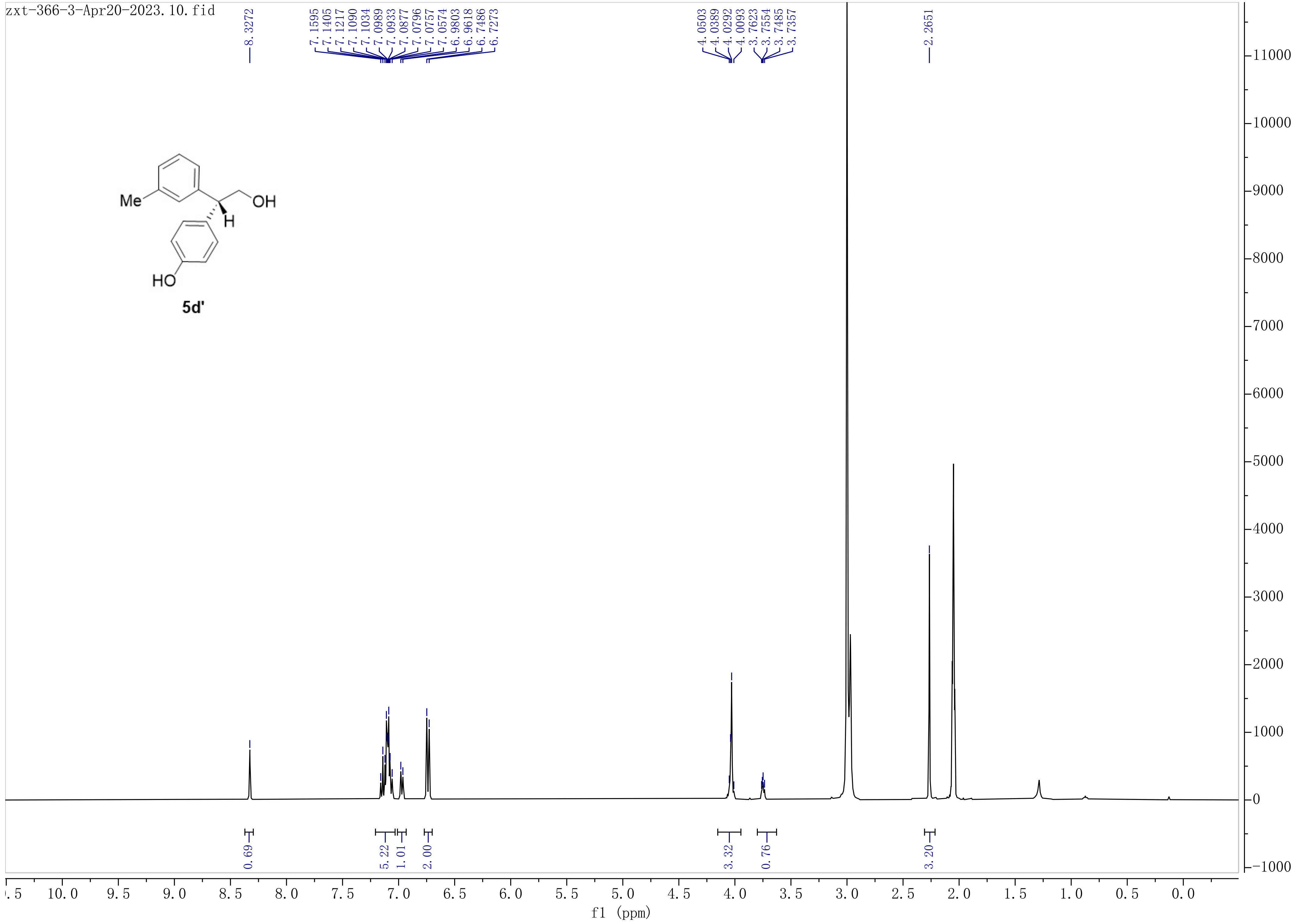
zxt-366-3-Apr20-2023.10.fid
8.3272
7.1595
7.1405
7.1217
7.1090
7.1034
7.0989
7.0933
7.0877
7.0796
7.0757
7.0574
6.9803
6.9618
6.7486
6.7273
4.0503
4.0389
4.0292
4.0093
3.7623
3.7554
3.7485
3.7357
2.2651
Me
OH
H
HO
5d'
0.69
5.22
1.01
2.00
3.32
0.76
3.20
f1 (ppm)

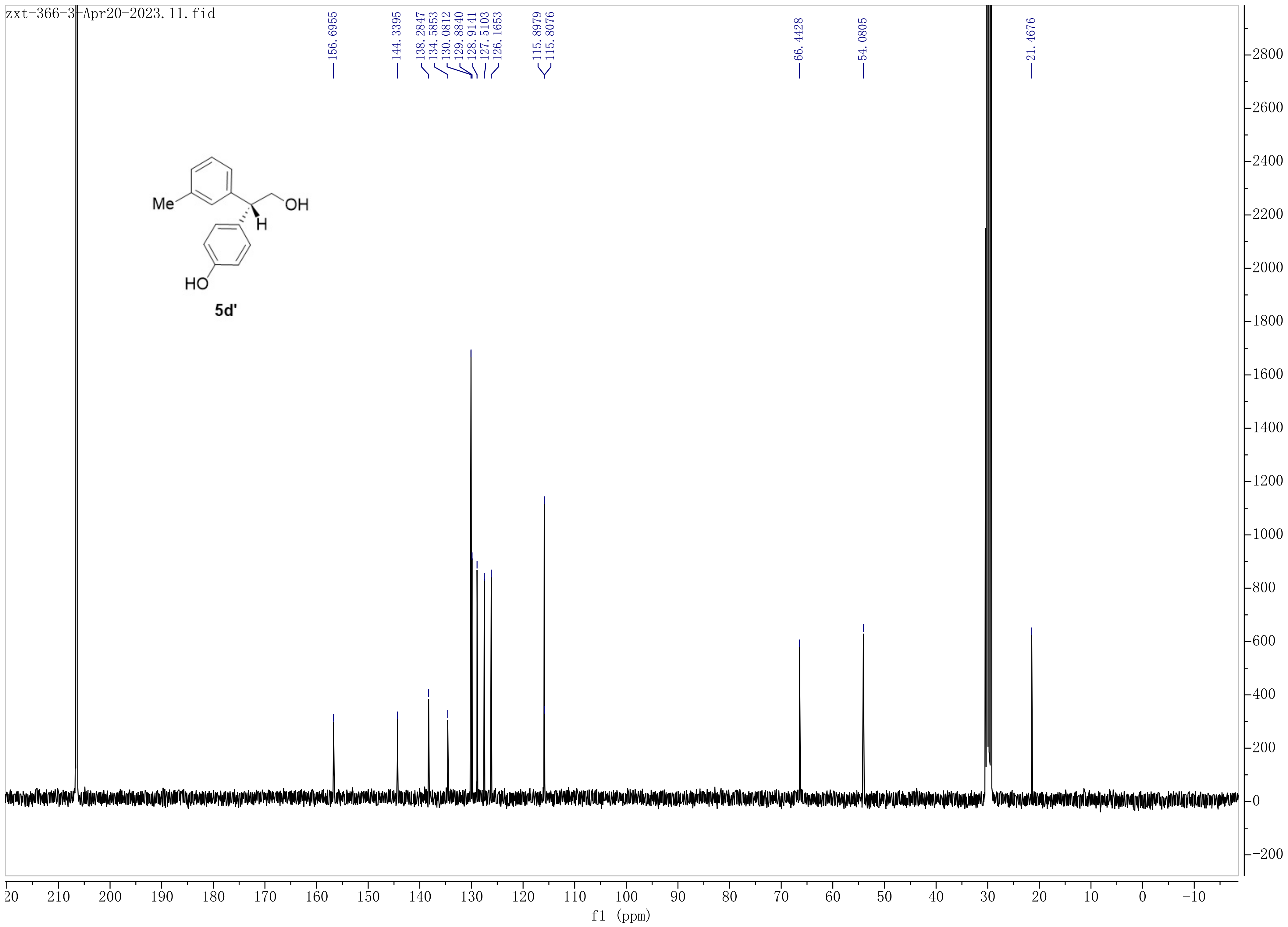
zxt-366-3-Apr20-2023.11.fid
156.6955
144.3395
138.2847
134.5853
130.0812
129.8840
128.9141
127.5103
126.1653
115.8979
115.8076
66.4428
54.0805
21.4676
Me
OH
H
HO
5d'
f1 (ppm)

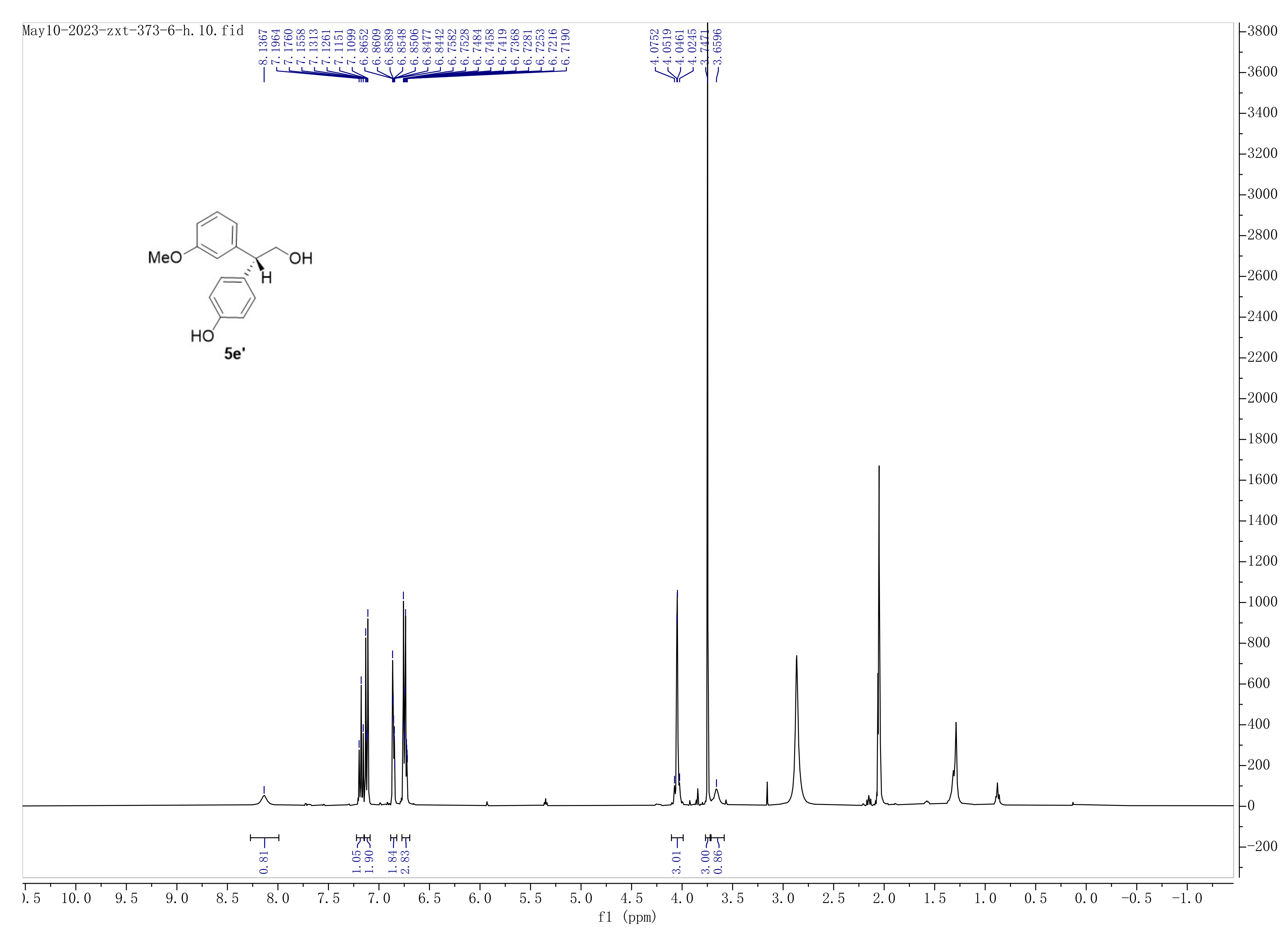
May10-2023-zxt-373-6-h.10.fid
8.1367
7.1964
7.1760
7.1558
7.1313
7.1261
7.1151
7.1099
6.8652
6.8609
6.8589
6.8548
6.8506
6.8477
6.8442
6.7582
6.7528
6.7484
6.7458
6.7419
6.7368
6.7281
6.7253
6.7216
6.7190
4.0752
4.0519
4.0461
4.0245
3.7471
3.6596
MeO
OH
H
HO
5e'
0.81
1.05
1.90
1.84
2.83
3.01
3.00
0.86
f1 (ppm)

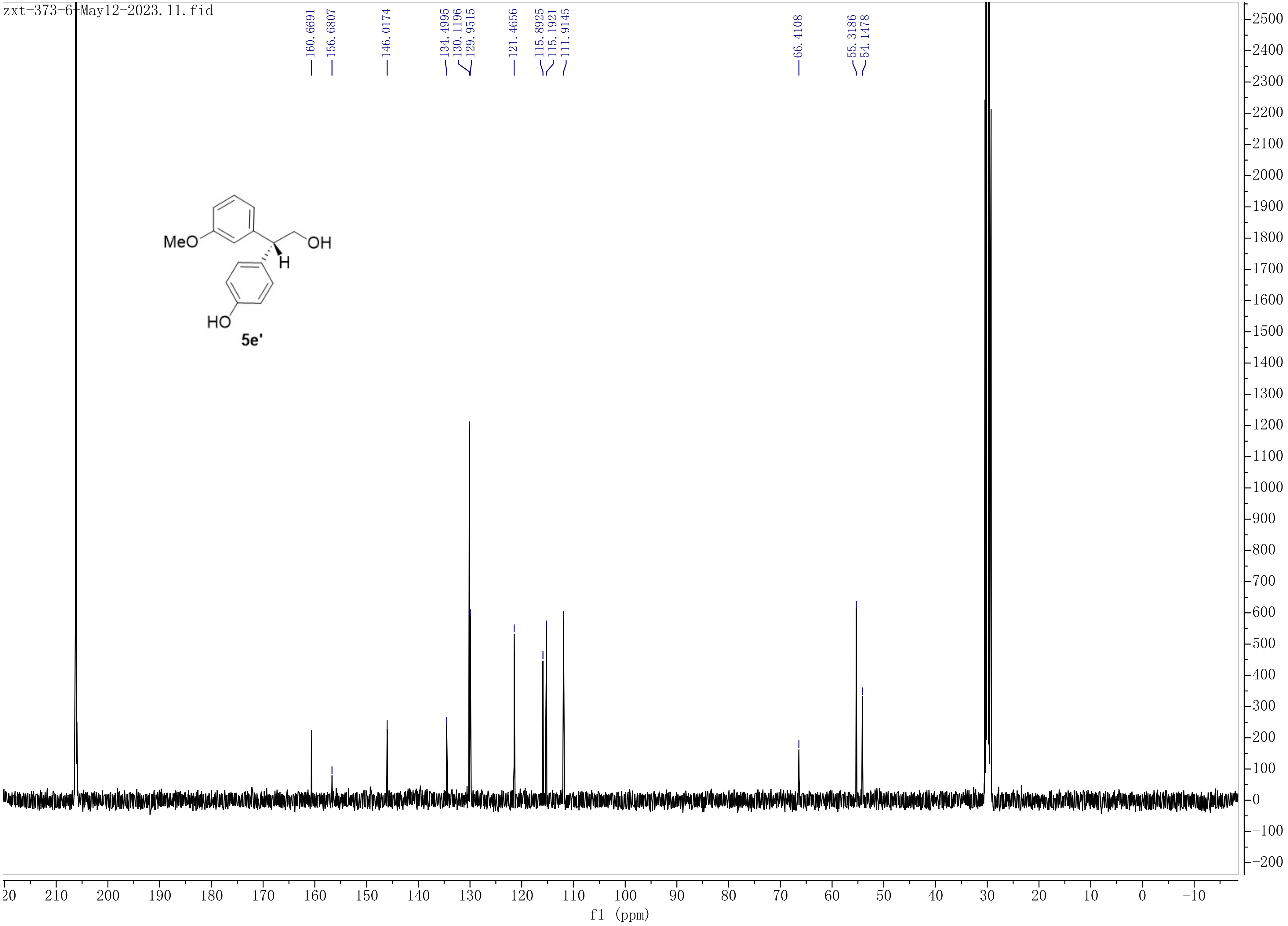
zxt-373-6-May12-2023.11.fid
160.6691
156.6807
146.0174
134.4995
130.1196
129.9515
121.4656
115.8925
115.1921
111.9145
66.4108
55.3186
54.1478
MeO
OH
H
HO
5e'
f1 (ppm)

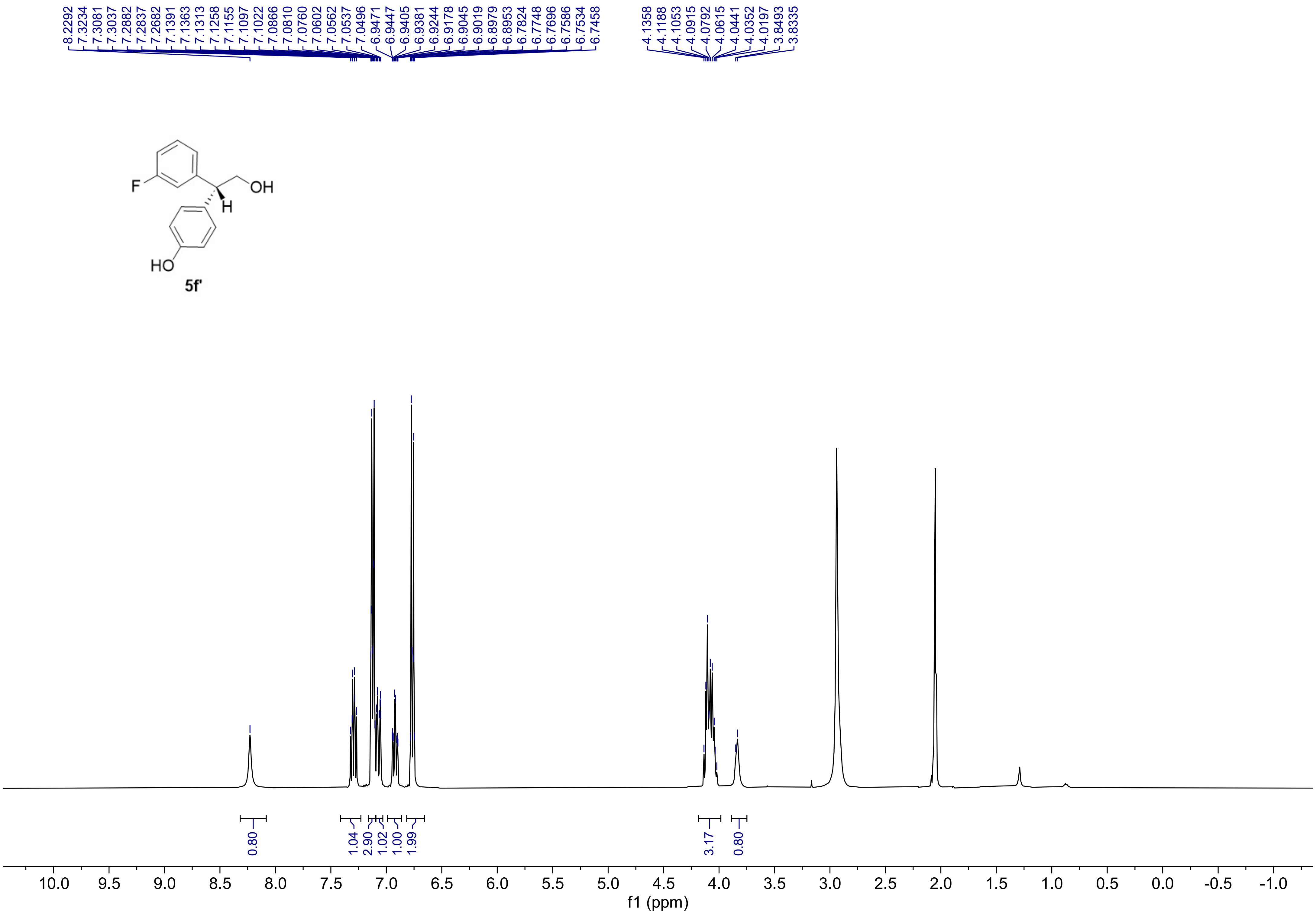

8.2292
7.3234
7.3081
7.3037
7.2882
7.2837
7.2682
7.1391
7.1363
7.1313
7.1258
7.1155
7.1097
7.1022
7.0866
7.0810
7.0760
7.0602
7.0562
7.0537
7.0496
6.9471
6.9447
6.9405
6.9381
6.9244
6.9178
6.9045
6.9019
6.8979
6.8953
6.7824
6.7748
6.7696
6.7586
6.7534
6.7458
4.1358
4.1188
4.1053
4.0915
4.0792
4.0615
4.0441
4.0352
4.0197
3.8493
3.8335
F
OH
H
HO
5f'
0.80
1.04
2.90
1.02
1.00
1.99
3.17
0.80
10.0 9.5 9.0 8.5 8.0 7.5 7.0 6.5 6.0 5.5 5.0 4.5 4.0 3.5 3.0 2.5 2.0 1.5 1.0 0.5 0.0 -0.5 -1.0
f1 (ppm)

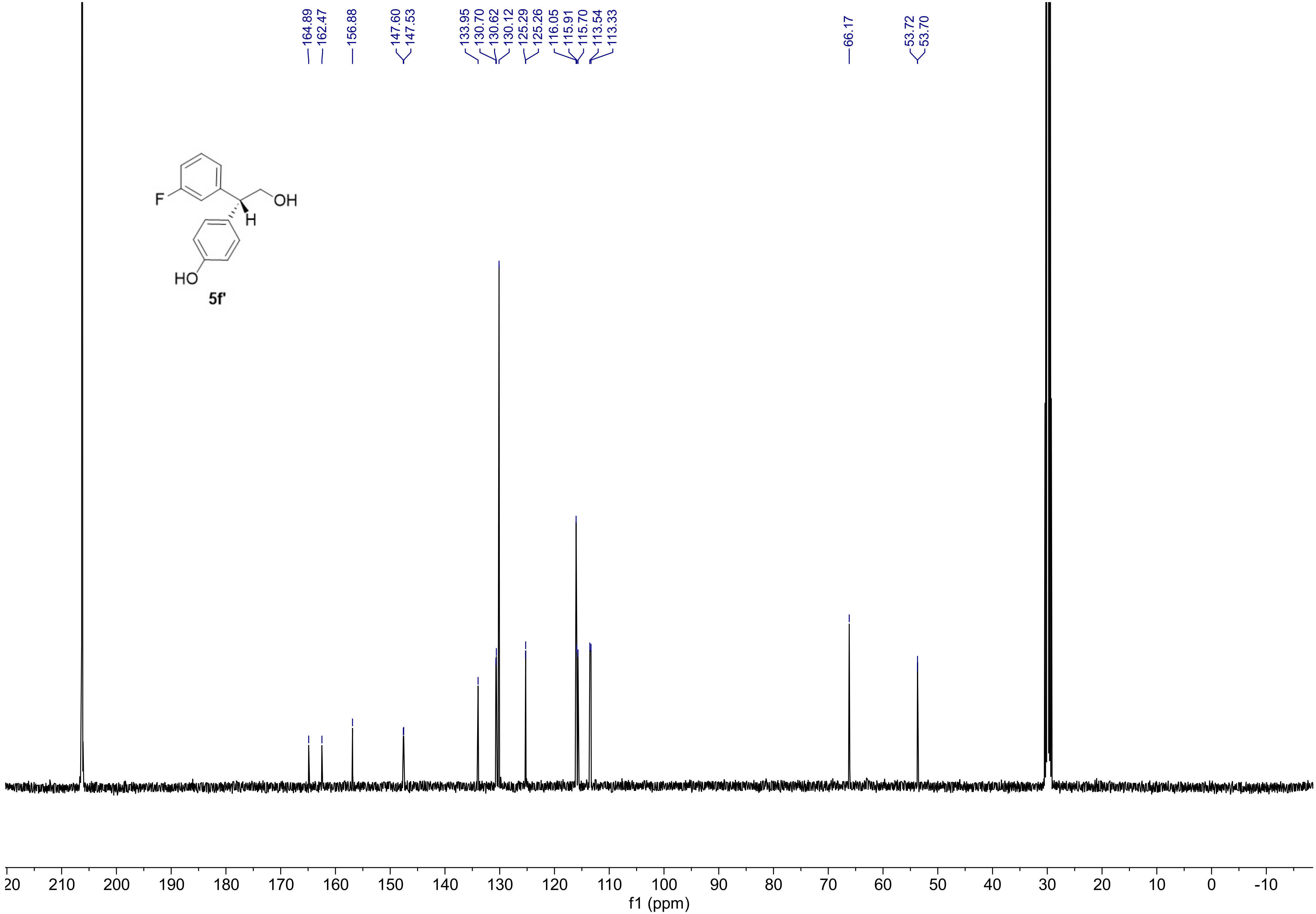
164.89
162.47
156.88
147.60
147.53
133.95
130.70
130.62
130.12
125.29
125.26
116.05
115.91
115.70
113.54
113.33
66.17
53.72
53.70
F
OH
H
HO
5f'
20 210 200 190 180 170 160 150 140 130 120 110 100 90 80 70 60 50 40 30 20 10 0 -10
f1 (ppm)

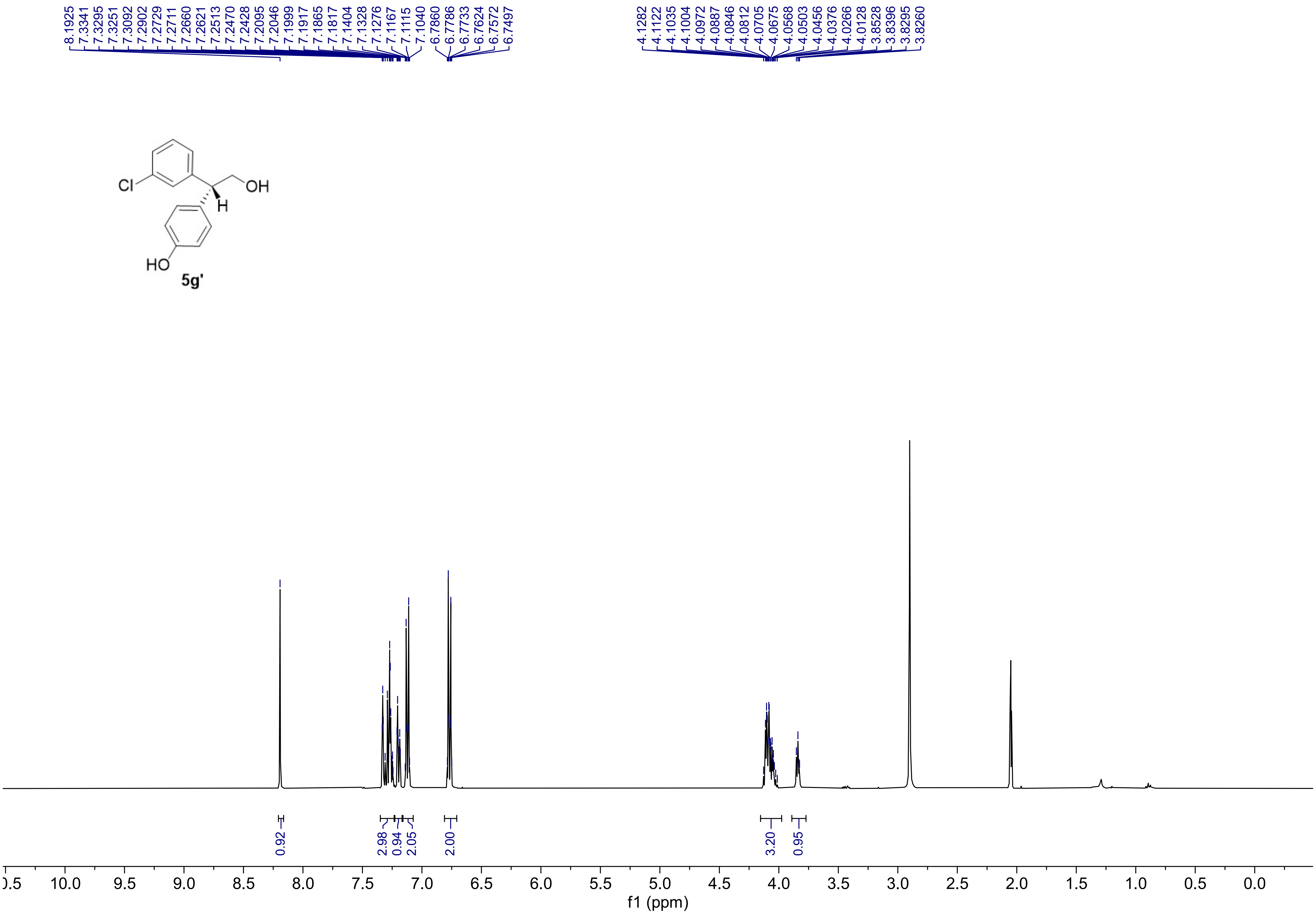
8.1925
7.3341
7.3295
7.3251
7.3092
7.2902
7.2729
7.2711
7.2660
7.2621
7.2513
7.2470
7.2428
7.2095
7.2046
7.1999
7.1917
7.1865
7.1817
7.1404
7.1328
7.1276
7.1167
7.1115
7.1040
6.7860
6.7786
6.7733
6.7624
6.7572
6.7497
4.1282
4.1122
4.1035
4.1004
4.0972
4.0887
4.0846
4.0812
4.0705
4.0675
4.0568
4.0503
4.0456
4.0376
4.0266
4.0128
3.8528
3.8396
3.8295
3.8260
Cl
OH
H
HO
5g'
0.92
2.98
0.94
2.05
2.00
3.20
0.95
10.0
9.5
9.0
8.5
8.0
7.5
7.0
6.5
6.0
5.5
5.0
4.5
4.0
3.5
3.0
2.5
2.0
1.5
1.0
0.5
0.0
f1 (ppm)

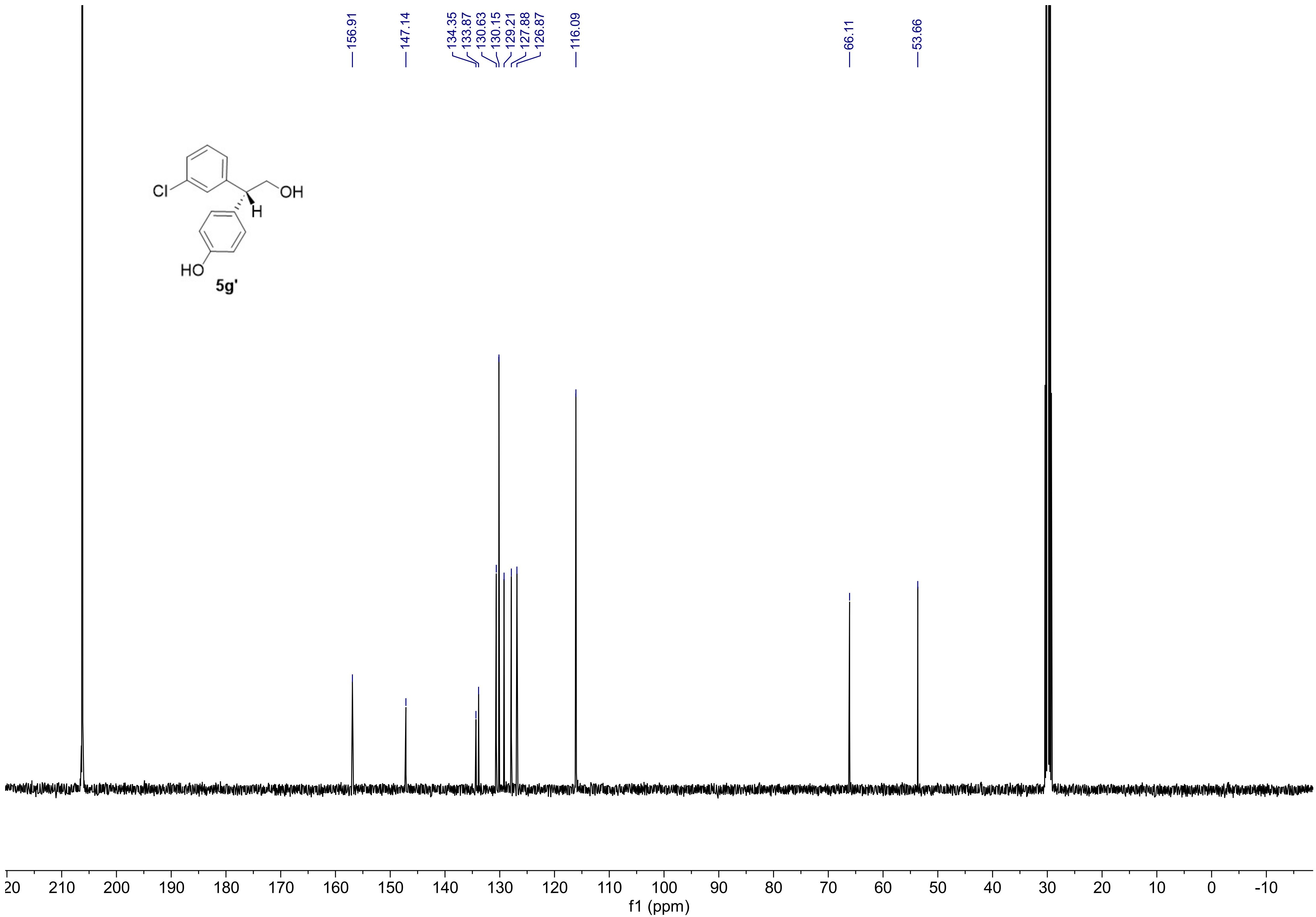

156.91
147.14
134.35
133.87
130.63
130.15
129.21
127.88
126.87
116.09
66.11
53.66
Cl
OH
H
HO
5g'
20
210
200
190
180
170
160
150
140
130
120
110
100
90
80
70
60
50
40
30
20
10
0
-10
f1 (ppm)

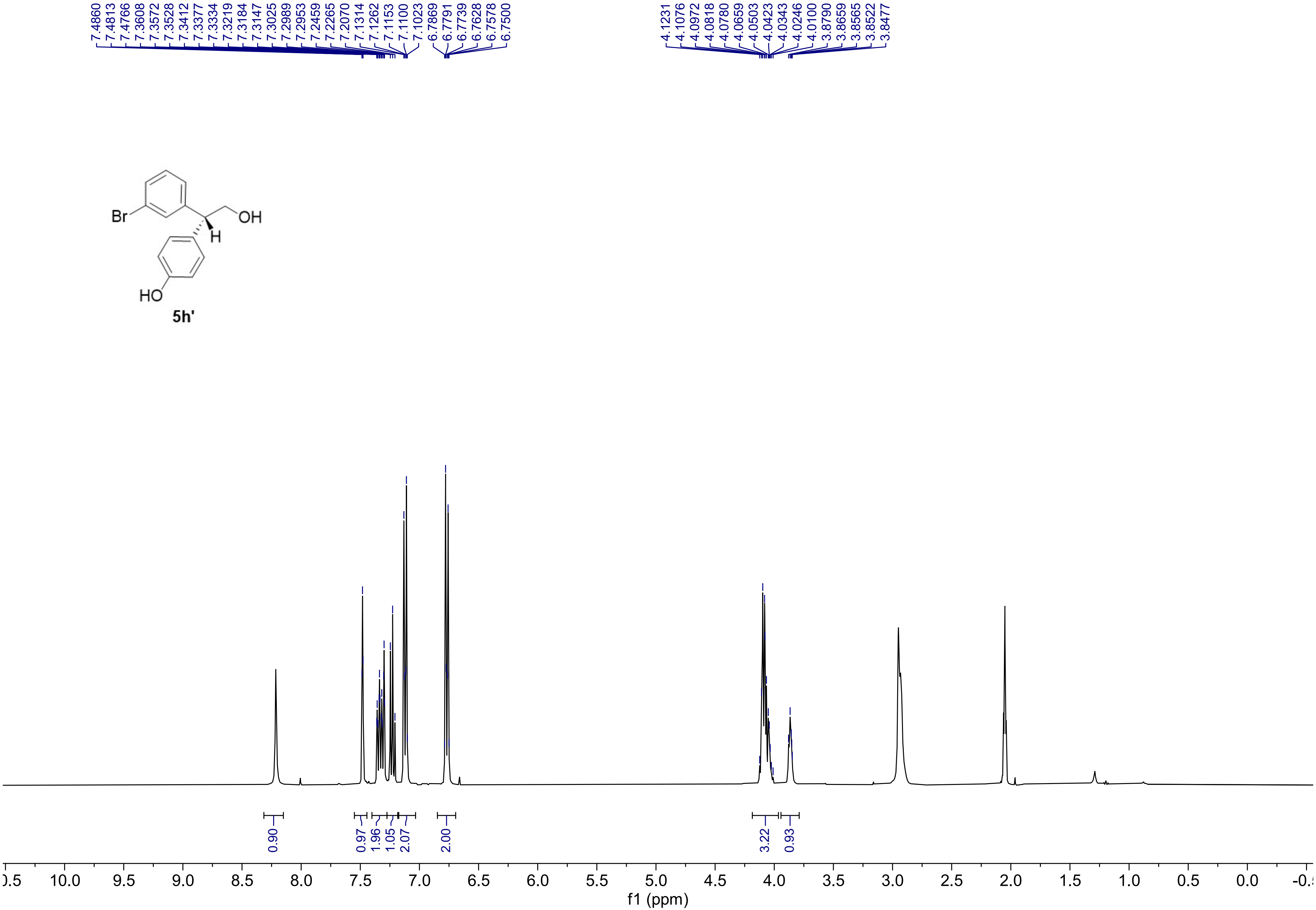
7.4860
7.4813
7.4766
7.3608
7.3572
7.3528
7.3412
7.3377
7.3334
7.3219
7.3184
7.3147
7.3025
7.2989
7.2953
7.2459
7.2265
7.2070
7.1314
7.1262
7.1153
7.1100
7.1023
6.7869
6.7791
6.7739
6.7628
6.7578
6.7500
4.1231
4.1076
4.0972
4.0818
4.0780
4.0659
4.0503
4.0423
4.0343
4.0246
4.0100
3.8790
3.8659
3.8565
3.8522
3.8477
Br
OH
H
HO
5h'
0.90
0.97
1.96
1.05
2.07
2.00
3.22
0.93
f1 (ppm)

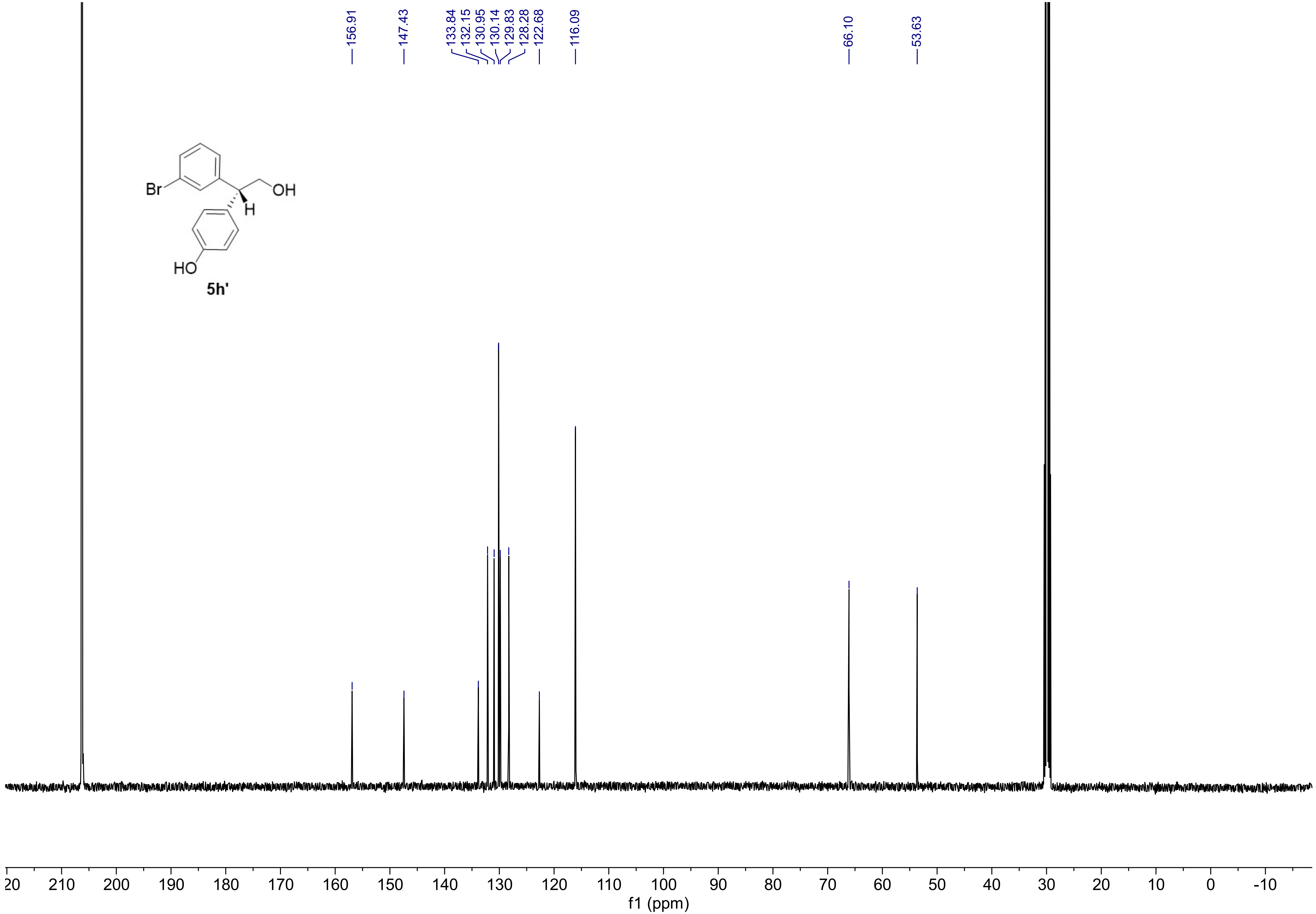
156.91
147.43
133.84
132.15
130.95
130.14
129.83
128.28
122.68
116.09
66.10
53.63
Br
OH
H
HO
5h'
20 210 200 190 180 170 160 150 140 130 120 110 100 90 80 70 60 50 40 30 20 10 0 -10
f1 (ppm)

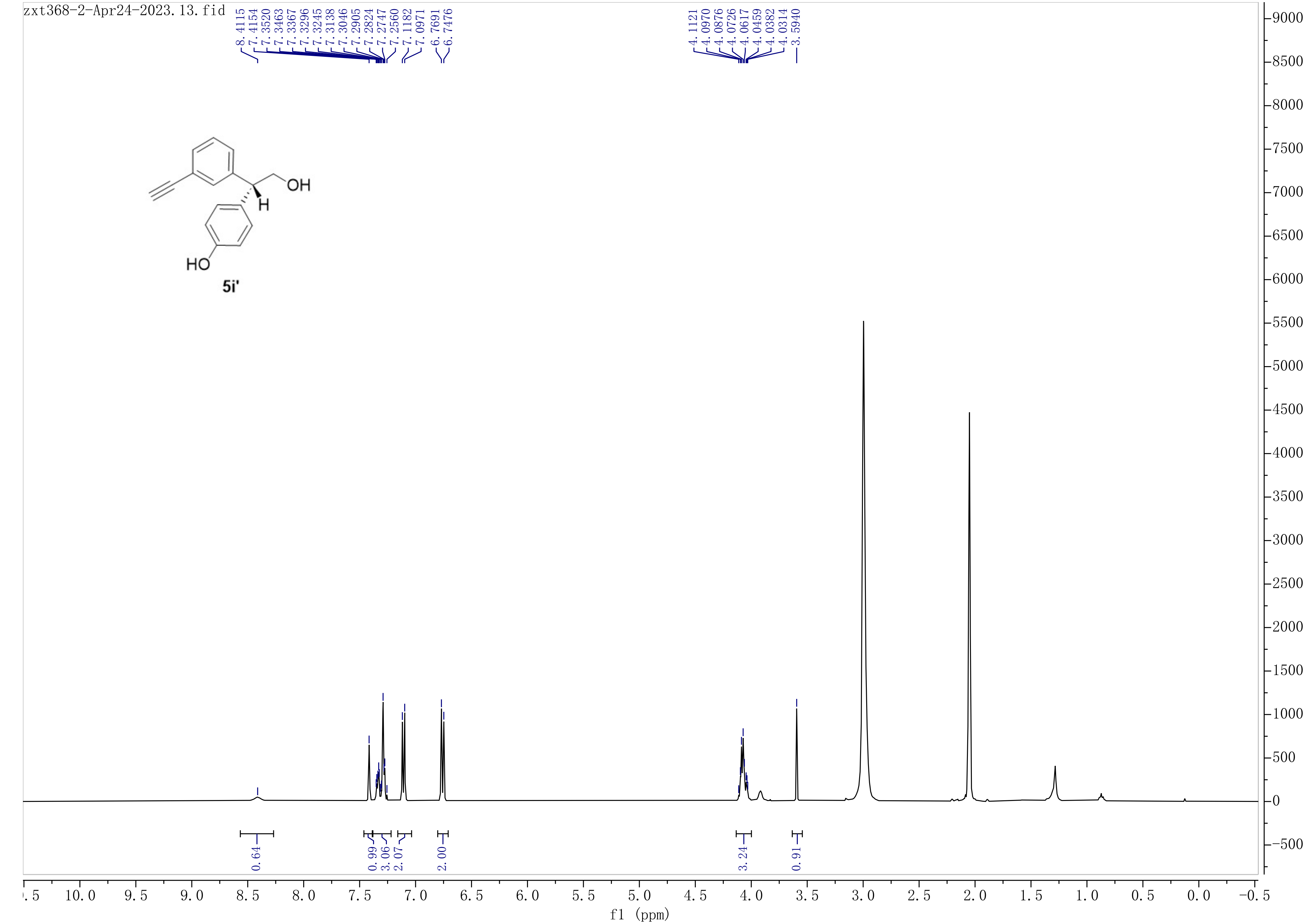
zxt368-2-Apr24-2023.13.fid
8.4115
7.4154
7.3520
7.3463
7.3367
7.3296
7.3245
7.3138
7.3046
7.2905
7.2824
7.2747
7.2560
7.1182
7.0971
6.7691
6.7476
4.1121
4.0970
4.0876
4.0726
4.0617
4.0459
4.0382
4.0314
3.5940
OH
H
HO
5i'
0.64
0.99
3.06
2.07
2.00
3.24
0.91
f1 (ppm)

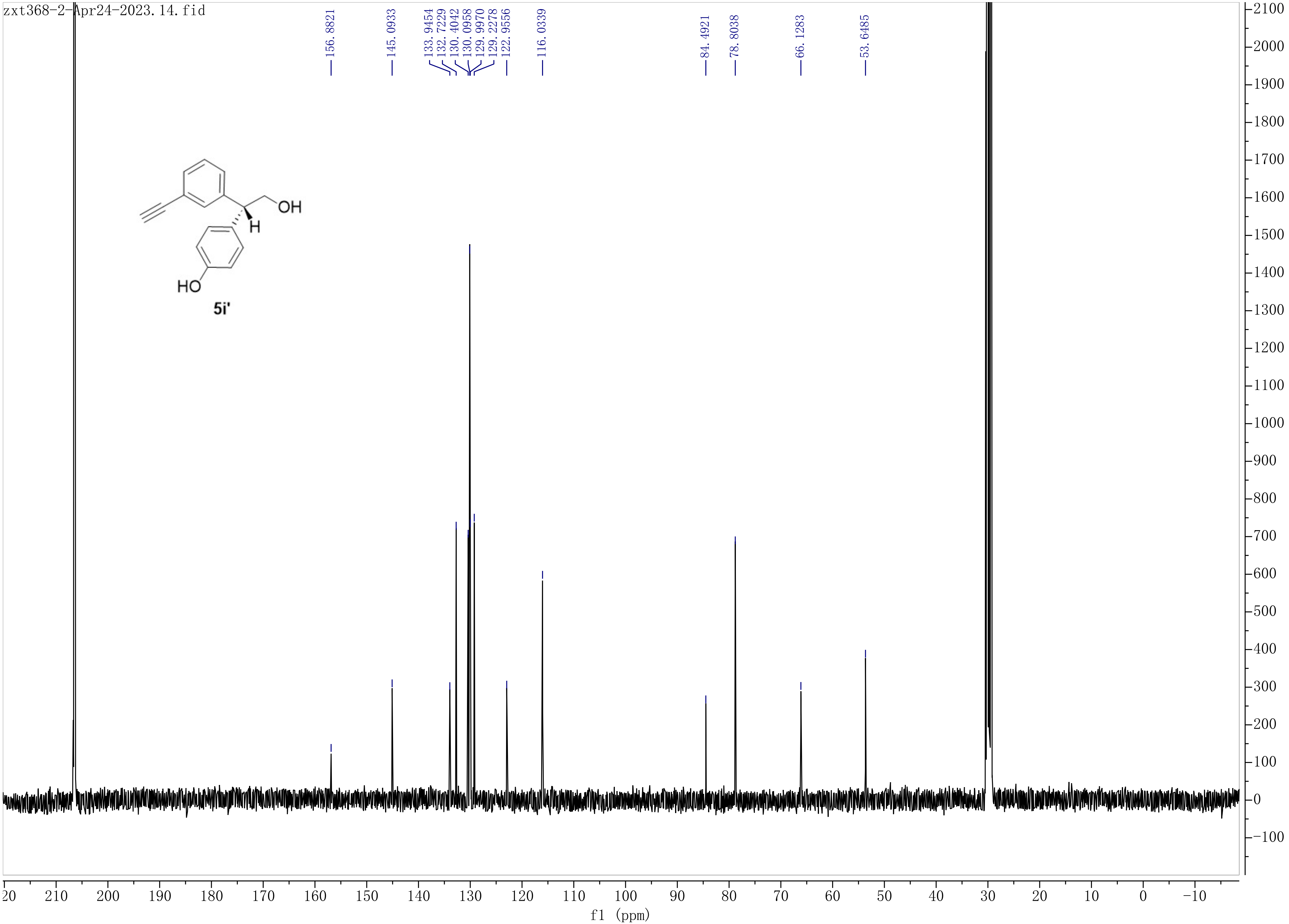
zxt368-2-Apr24-2023.14.fid
156.8821
145.0933
133.9454
132.7229
130.4042
130.0958
129.9970
129.2278
122.9556
116.0339
84.4921
78.8038
66.1283
53.6485
OH
H
HO
5i'
f1 (ppm)

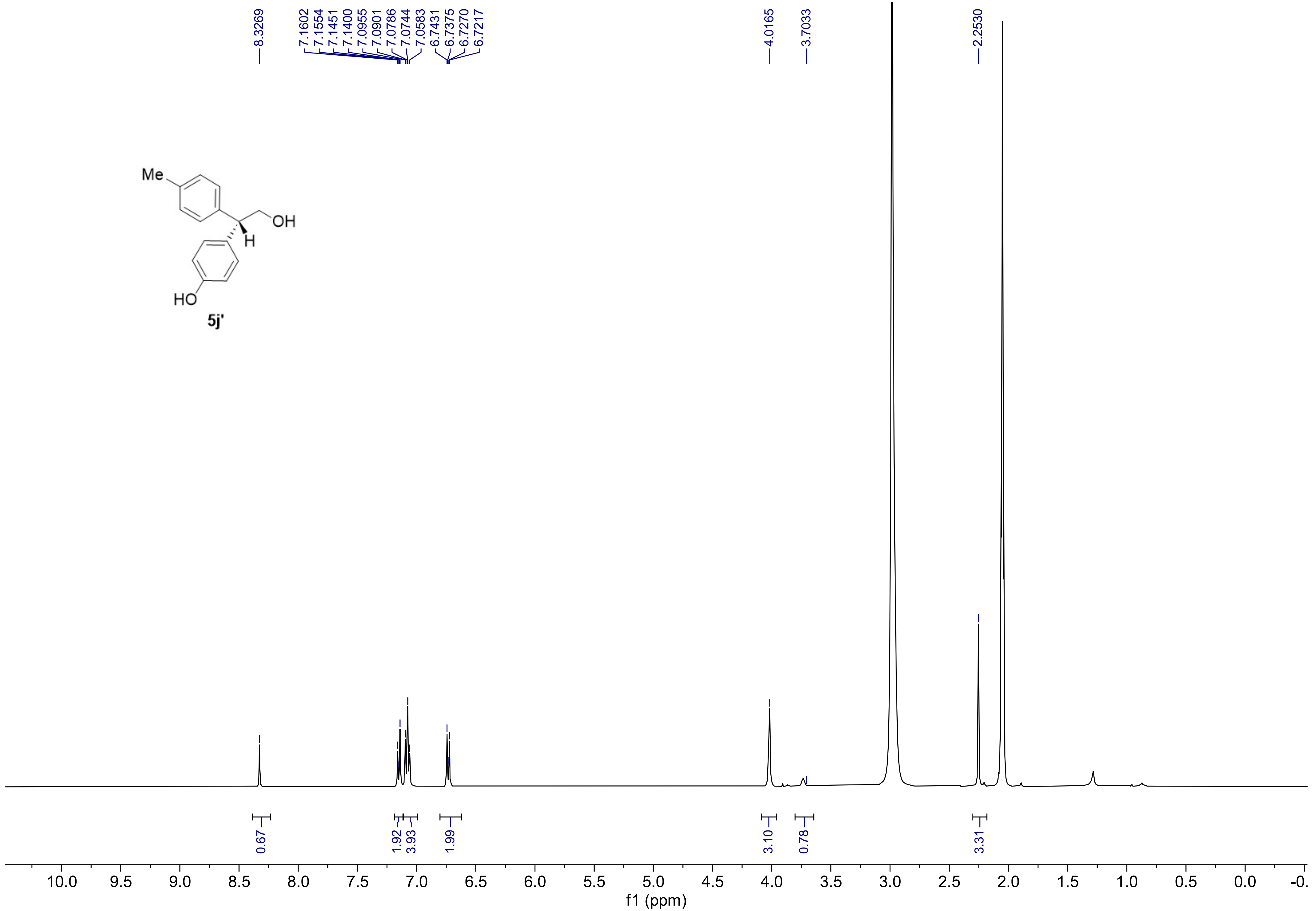

8.3269
7.1602
7.1554
7.1451
7.1400
7.0955
7.0901
7.0786
7.0744
7.0583
6.7431
6.7375
6.7270
6.7217
4.0165
3.7033
2.2530
Me
OH
H
HO
5j'
0.67
1.92
3.93
1.99
3.10
0.78
3.31
10.0 9.5 9.0 8.5 8.0 7.5 7.0 6.5 6.0 5.5 5.0 4.5 4.0 3.5 3.0 2.5 2.0 1.5 1.0 0.5 0.0
f1 (ppm)

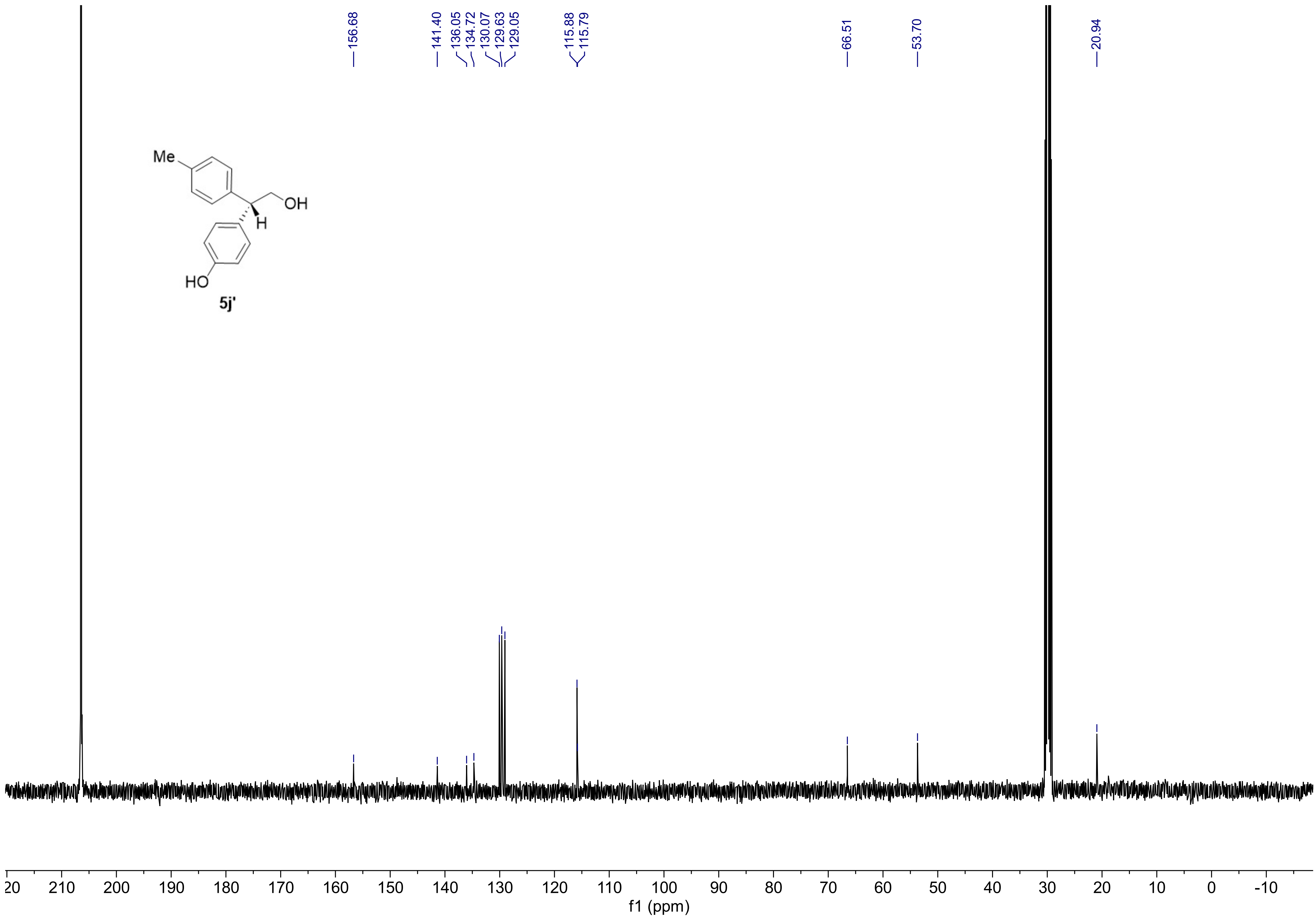
156.68
141.40
136.05
134.72
130.07
129.63
129.05
115.88
115.79
66.51
53.70
20.94
Me
OH
H
HO
5j'
20
210
200
190
180
170
160
150
140
130
120
110
100
90
80
70
60
50
40
30
20
10
0
-10
f1 (ppm)

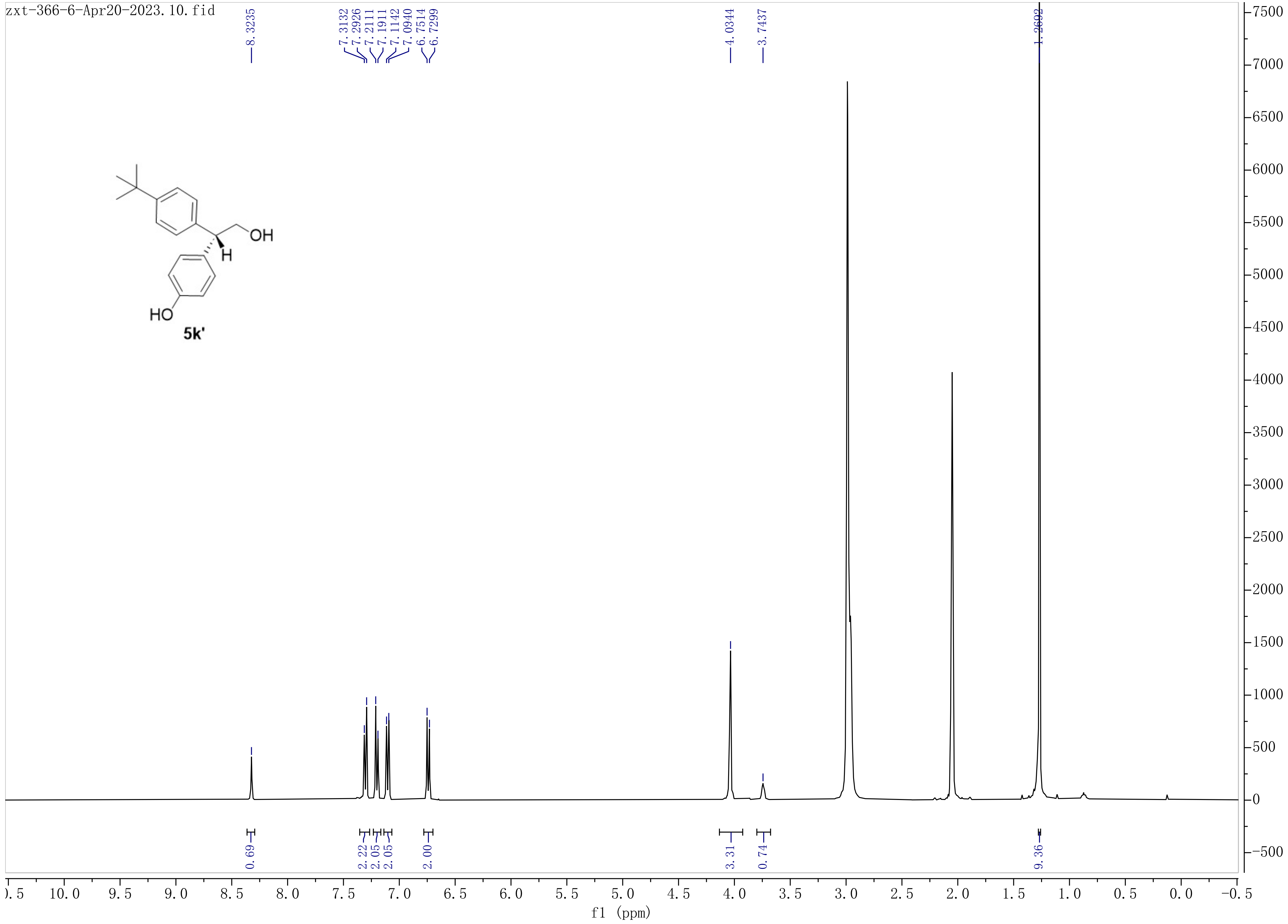
zxt-366-6-Apr20-2023.10.fid
8.3235
7.3132
7.2926
7.2111
7.1911
7.1142
7.0940
6.7514
6.7299
4.0344
3.7437
1.2692
OH
H
HO
5k'
0.69
2.22
2.05
2.05
2.00
3.31
0.74
9.36
f1 (ppm)

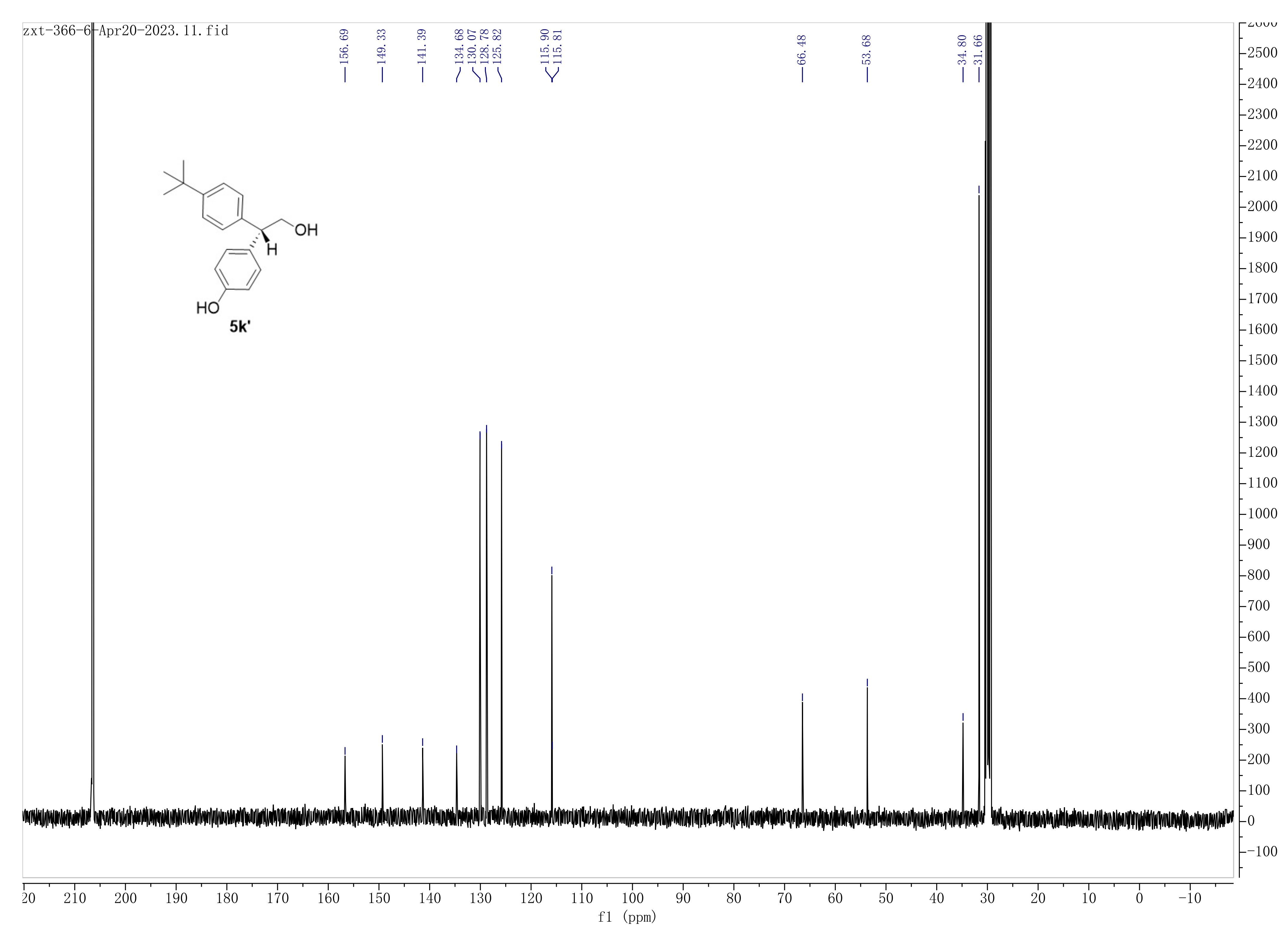
zxt-366-6-Apr20-2023.11.fid
156.69
149.33
141.39
134.68
130.07
128.78
125.82
115.90
115.81
66.48
53.68
34.80
31.66
OH
H
HO
5k'
f1 (ppm)

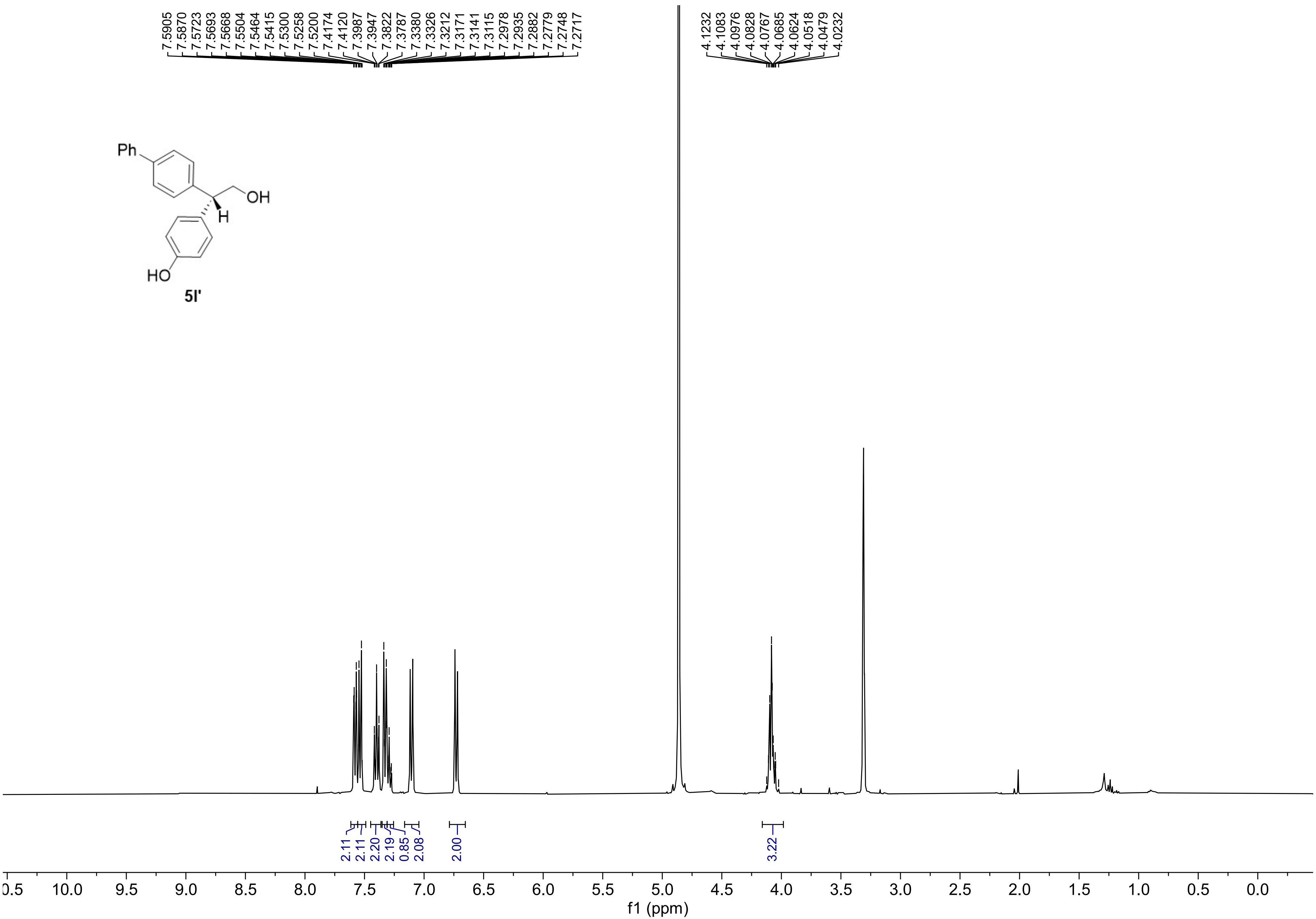
7.5905
7.5870
7.5723
7.5693
7.5668
7.5504
7.5464
7.5415
7.5300
7.5258
7.5200
7.4174
7.4120
7.3987
7.3947
7.3822
7.3787
7.3380
7.3326
7.3212
7.3171
7.3141
7.3115
7.2978
7.2935
7.2882
7.2779
7.2748
7.2717
4.1232
4.1083
4.0976
4.0828
4.0767
4.0685
4.0624
4.0518
4.0479
4.0232
Ph
OH
H
HO
5l'
2.11
2.11
2.20
2.19
0.85
2.08
2.00
3.22
0.5
10.0
9.5
9.0
8.5
8.0
7.5
7.0
6.5
6.0
5.5
5.0
4.5
4.0
3.5
3.0
2.5
2.0
1.5
1.0
0.5
0.0
f1 (ppm)

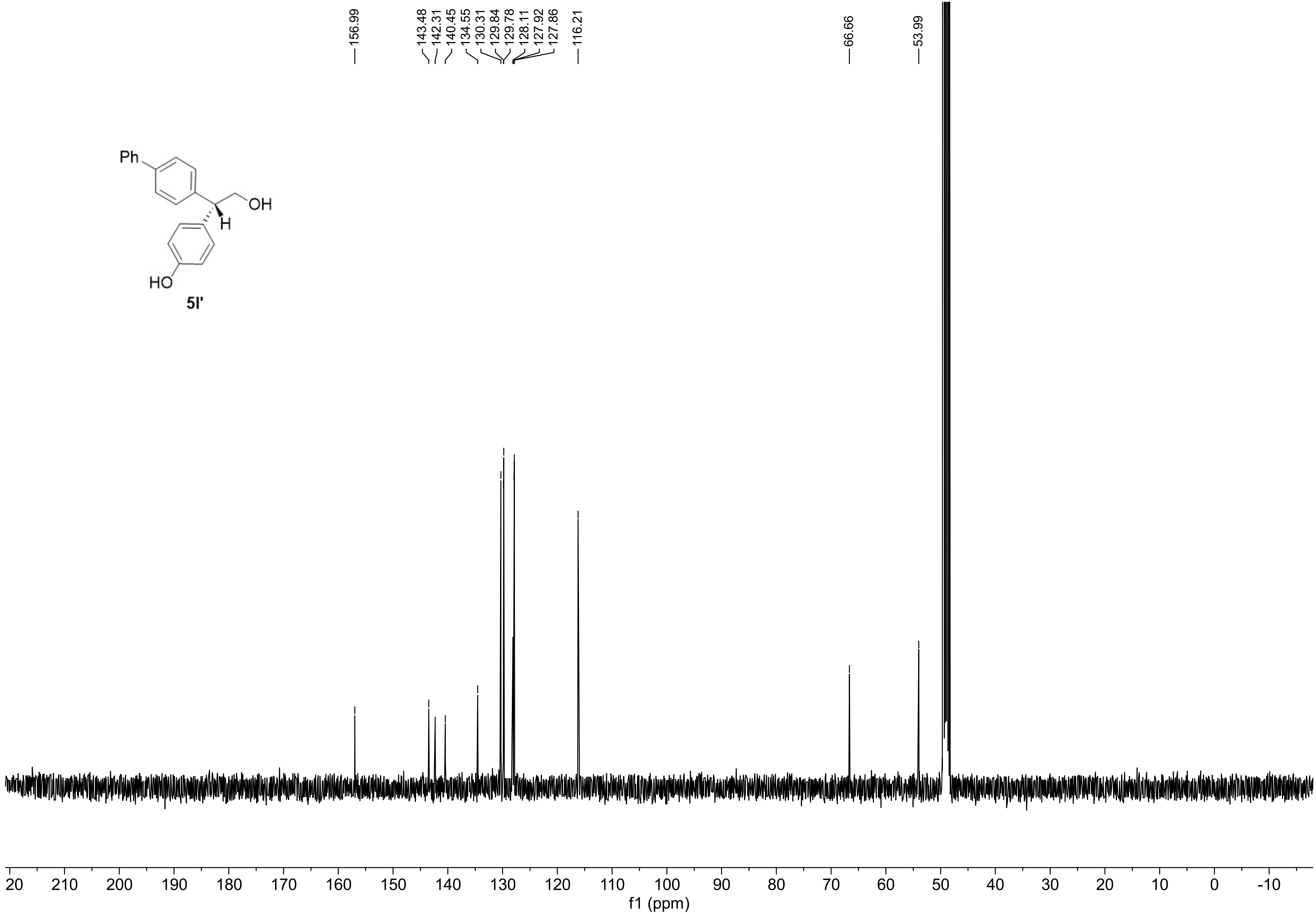
156.99
143.48
142.31
140.45
134.55
130.31
129.84
129.78
128.11
127.92
127.86
116.21
66.66
53.99
Ph
OH
H
HO
5l'
20 210 200 190 180 170 160 150 140 130 120 110 100 90 80 70 60 50 40 30 20 10 0 -10
f1 (ppm)

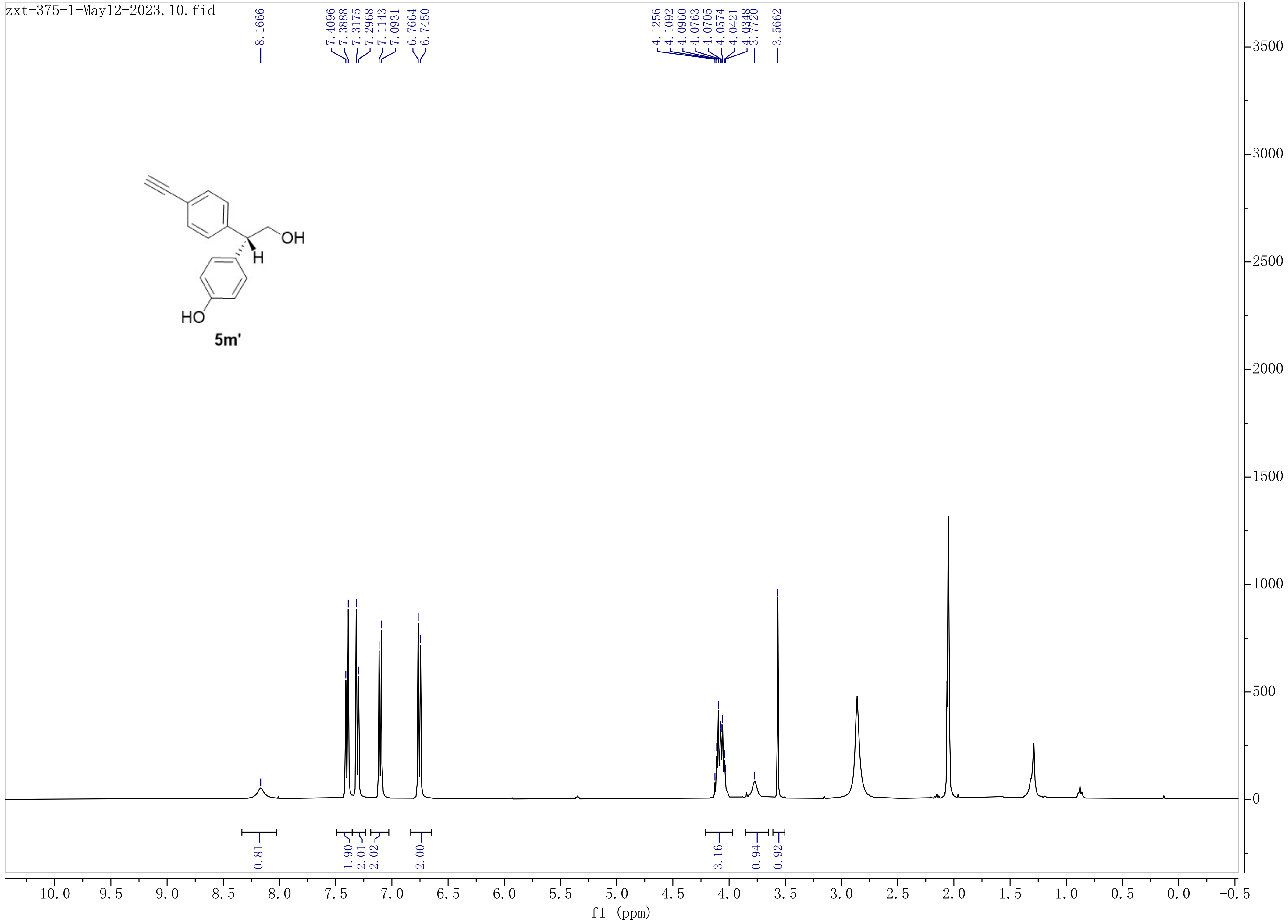
zxt-375-1-May12-2023.10.fid
8.1666
7.4096
7.3888
7.3175
7.2968
7.1143
7.0931
6.7664
6.7450
4.1256
4.1092
4.0960
4.0763
4.0705
4.0574
4.0421
4.0348
3.7720
3.5662
OH
H
HO
5m'
0.81
1.90
2.01
2.02
2.00
3.16
0.94
0.92
f1 (ppm)

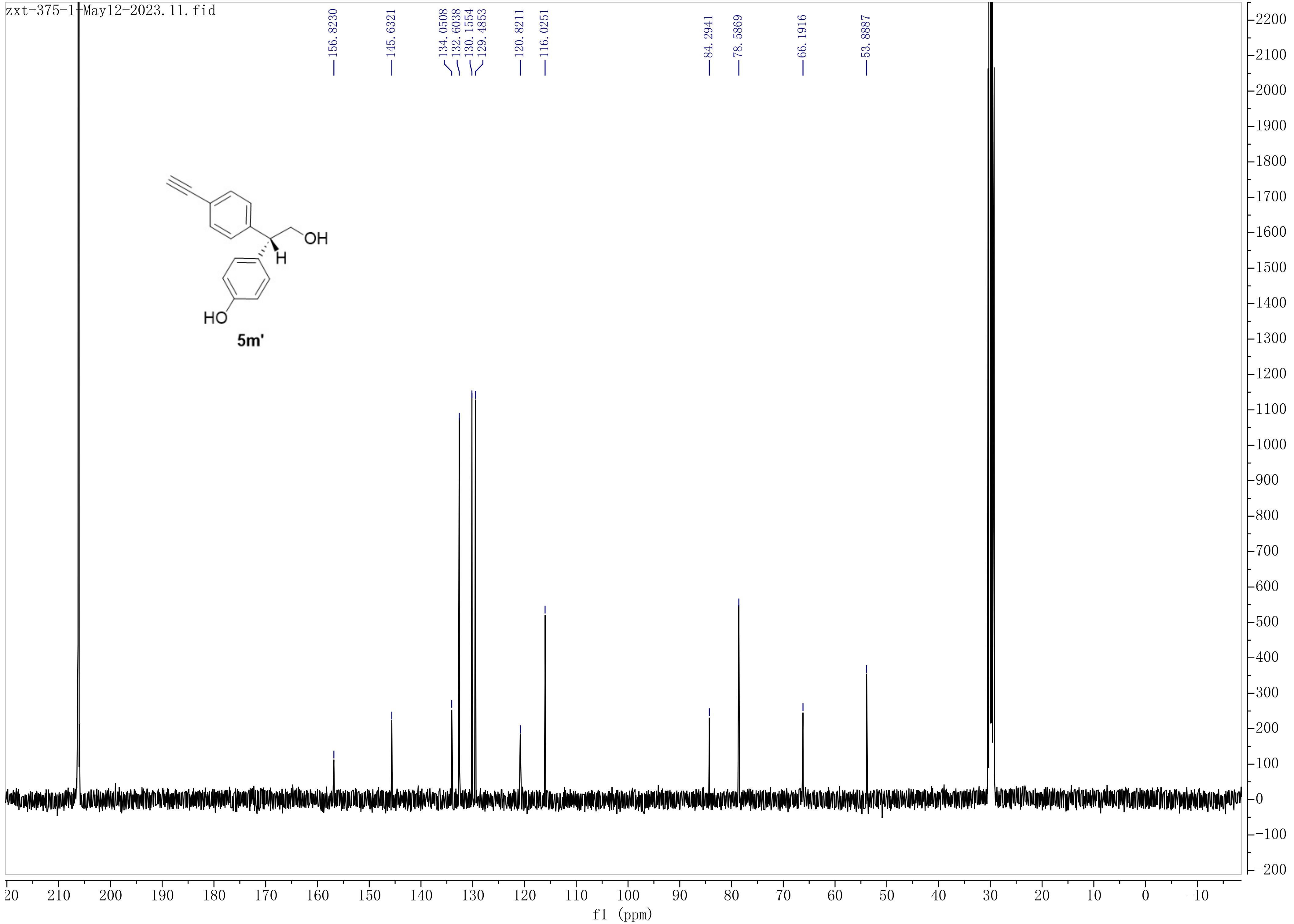
zxt-375-1-May12-2023.11.fid
156.8230
145.6321
134.0508
132.6038
130.1554
129.4853
120.8211
116.0251
84.2941
78.5869
66.1916
53.8887
OH
H
HO
5m'
f1 (ppm)

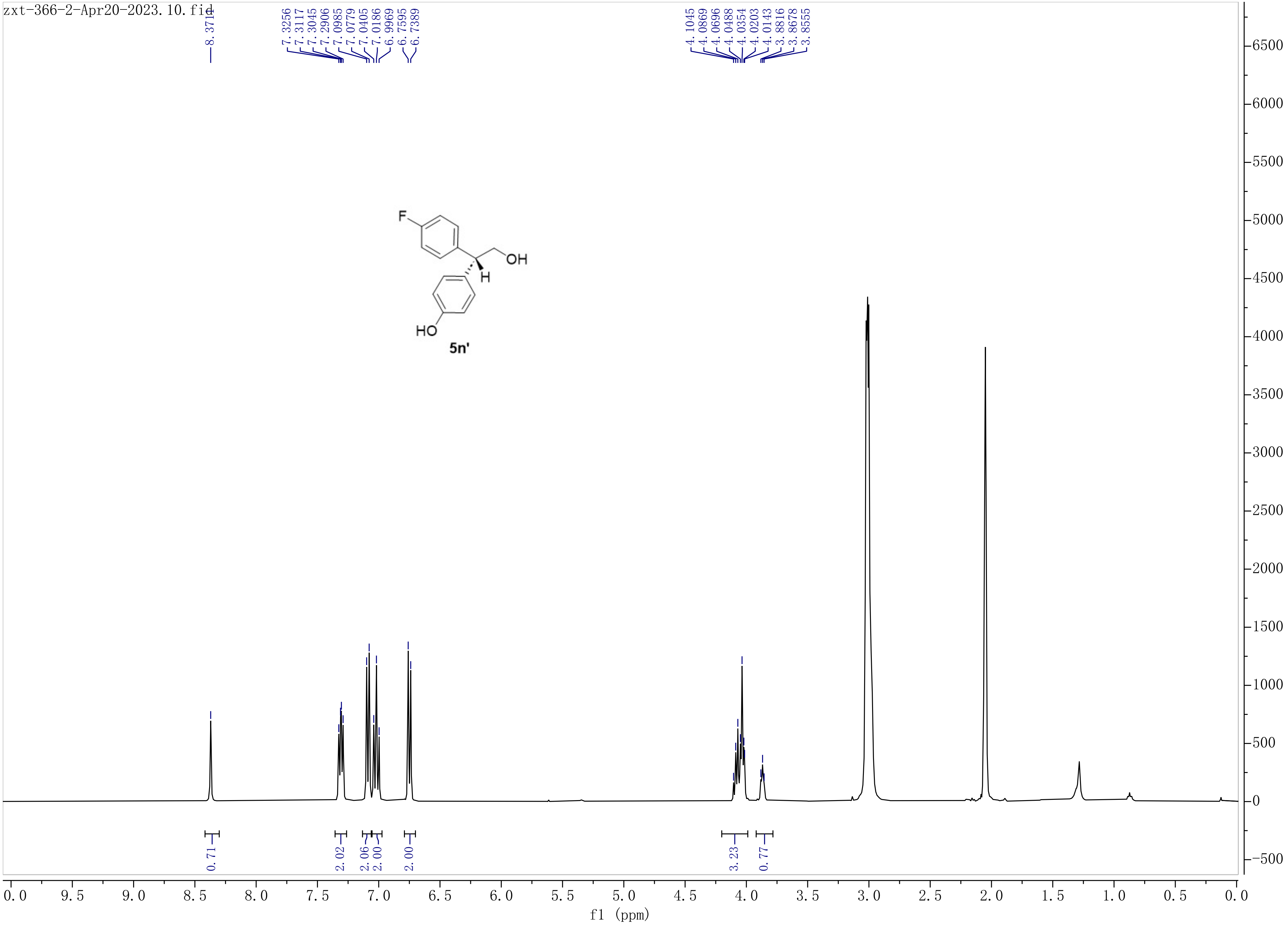

zxt-366-2-Apr20-2023.10.fid
8.3718
7.3256
7.3117
7.3045
7.2906
7.0985
7.0779
7.0405
7.0186
6.9969
6.7595
6.7389
4.1045
4.0869
4.0696
4.0488
4.0354
4.0203
4.0143
3.8816
3.8678
3.8555
F
OH
H
HO
5n'
0.71
2.02
2.06
2.00
2.00
3.23
0.77
f1 (ppm)

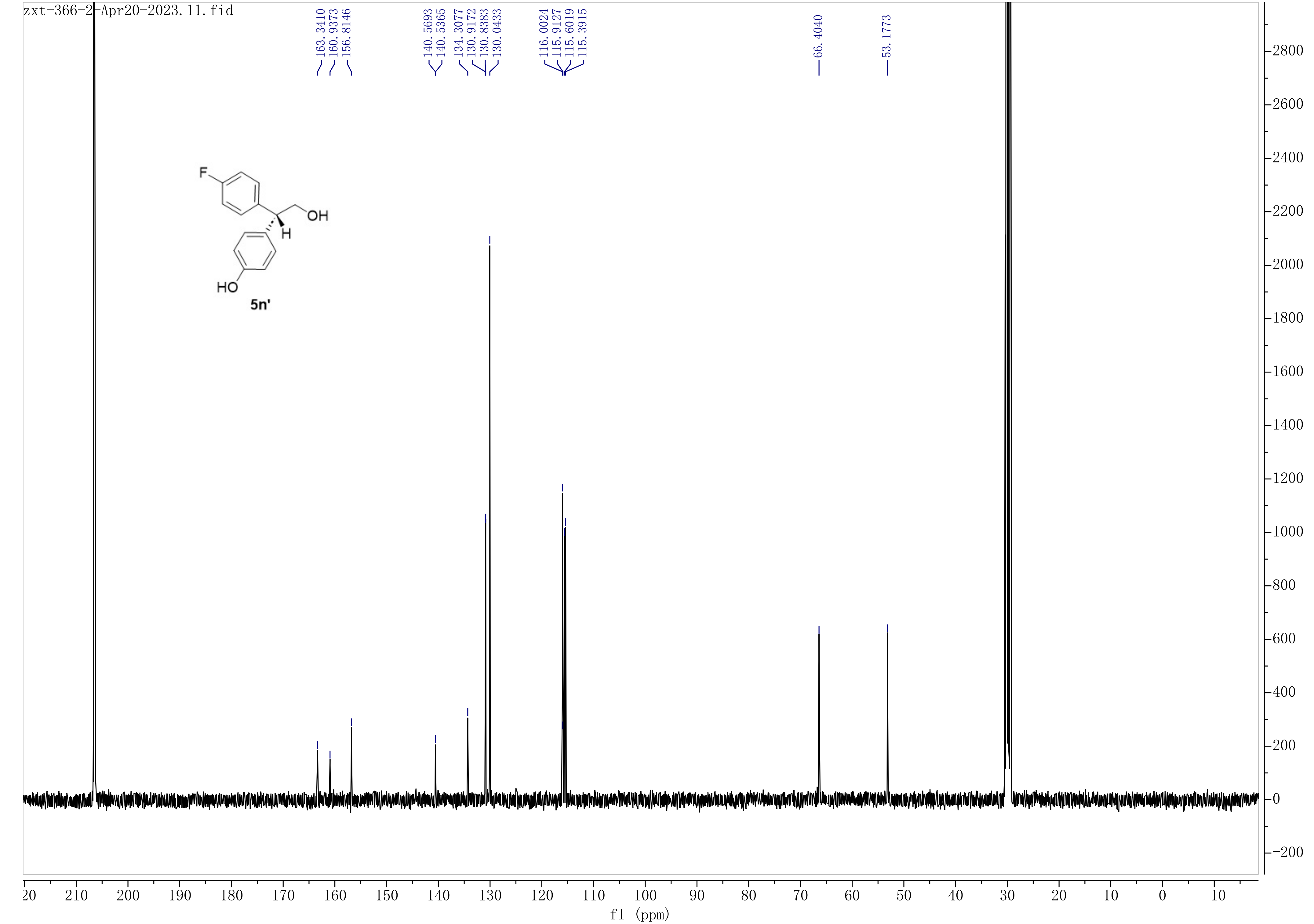
zxt-366-2-Apr20-2023.11.fid
163.3410
160.9373
156.8146
140.5693
140.5365
134.3077
130.9172
130.8383
130.0433
116.0024
115.9127
115.6019
115.3915
66.4040
53.1773
F
OH
H
HO
5n'
f1 (ppm)

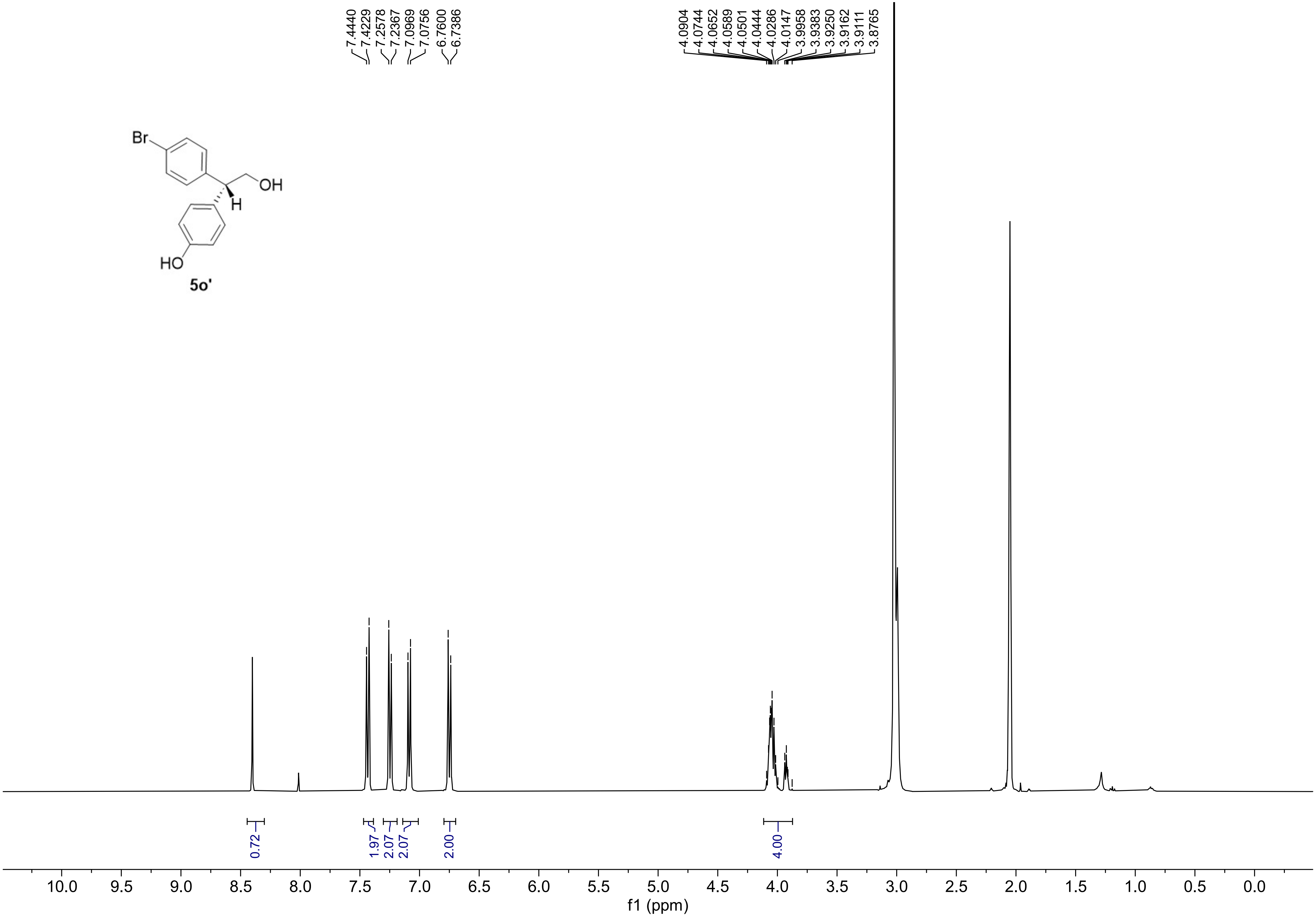
Br
OH
H
HO
5o'
7.4440
7.4229
7.2578
7.2367
7.0969
7.0756
6.7600
6.7386
4.0904
4.0744
4.0652
4.0589
4.0501
4.0444
4.0286
4.0147
3.9958
3.9383
3.9250
3.9162
3.9111
3.8765
0.72
1.97
2.07
2.07
2.00
4.00
10.0
9.5
9.0
8.5
8.0
7.5
7.0
6.5
6.0
5.5
5.0
4.5
4.0
3.5
3.0
2.5
2.0
1.5
1.0
0.5
0.0
f1 (ppm)

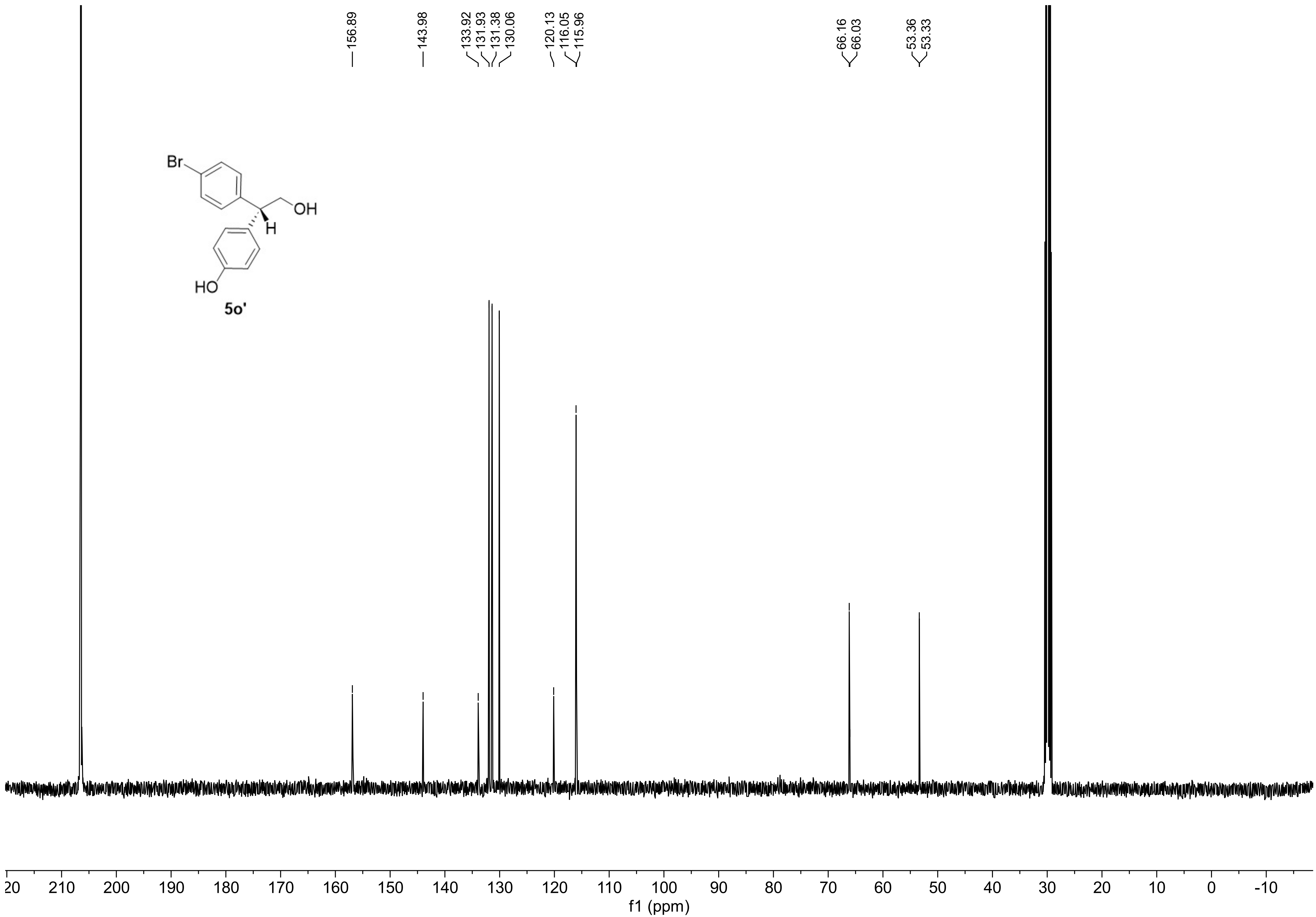
156.89
143.98
133.92
131.93
131.38
130.06
120.13
116.05
115.96
66.16
66.03
53.36
53.33
Br
OH
H
HO
5o'
20 210 200 190 180 170 160 150 140 130 120 110 100 90 80 70 60 50 40 30 20 10 0 -10
f1 (ppm)

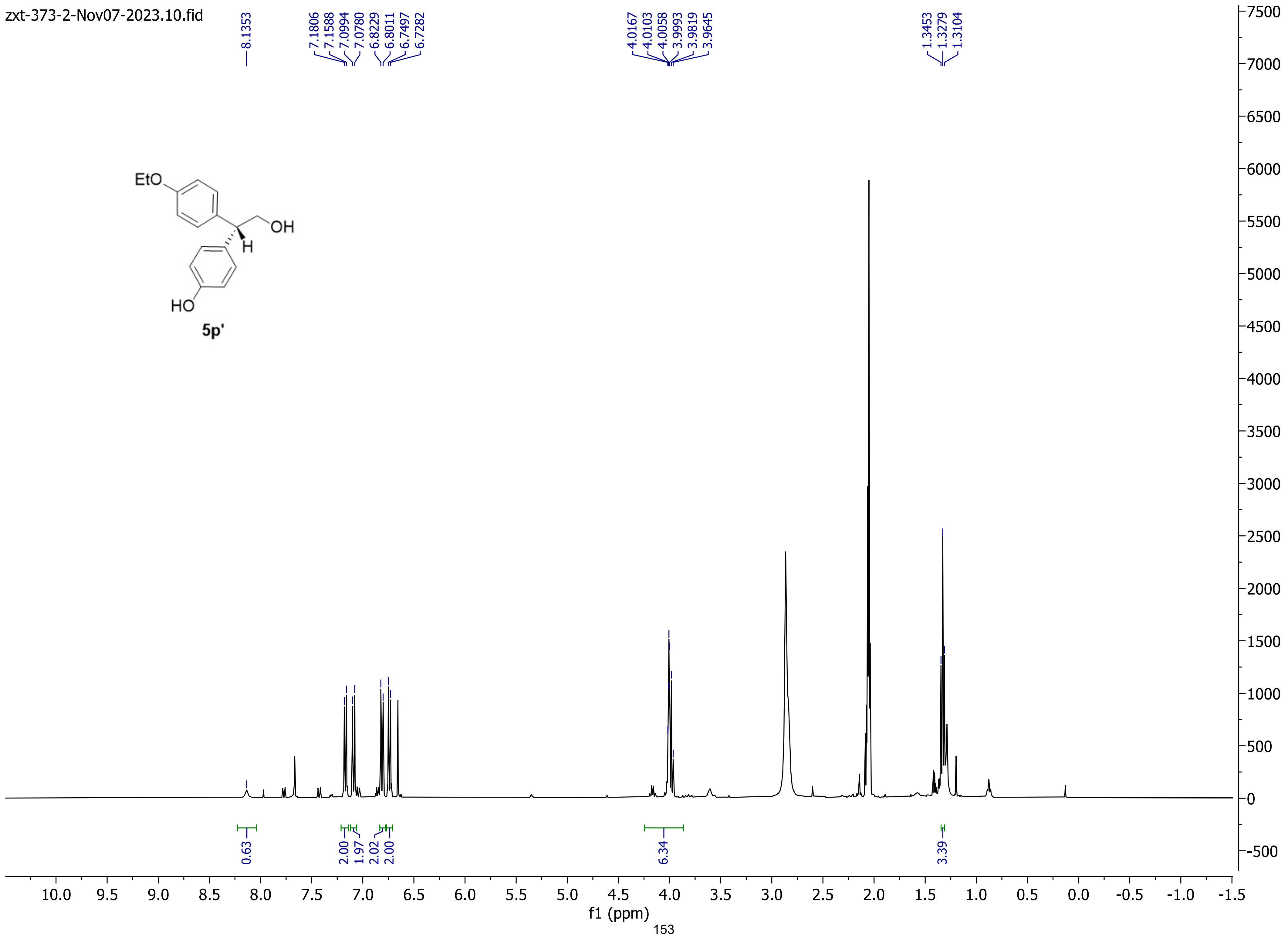

zxt-373-2-Nov07-2023.10.fid
8.1353
7.1806
7.1588
7.0994
7.0780
6.8229
6.8011
6.7497
6.7282
4.0167
4.0103
4.0058
3.9993
3.9819
3.9645
1.3453
1.3279
1.3104
EtO
OH
H
HO
5p'
0.63
2.00
1.97
2.02
2.00
6.34
3.39
f1 (ppm)

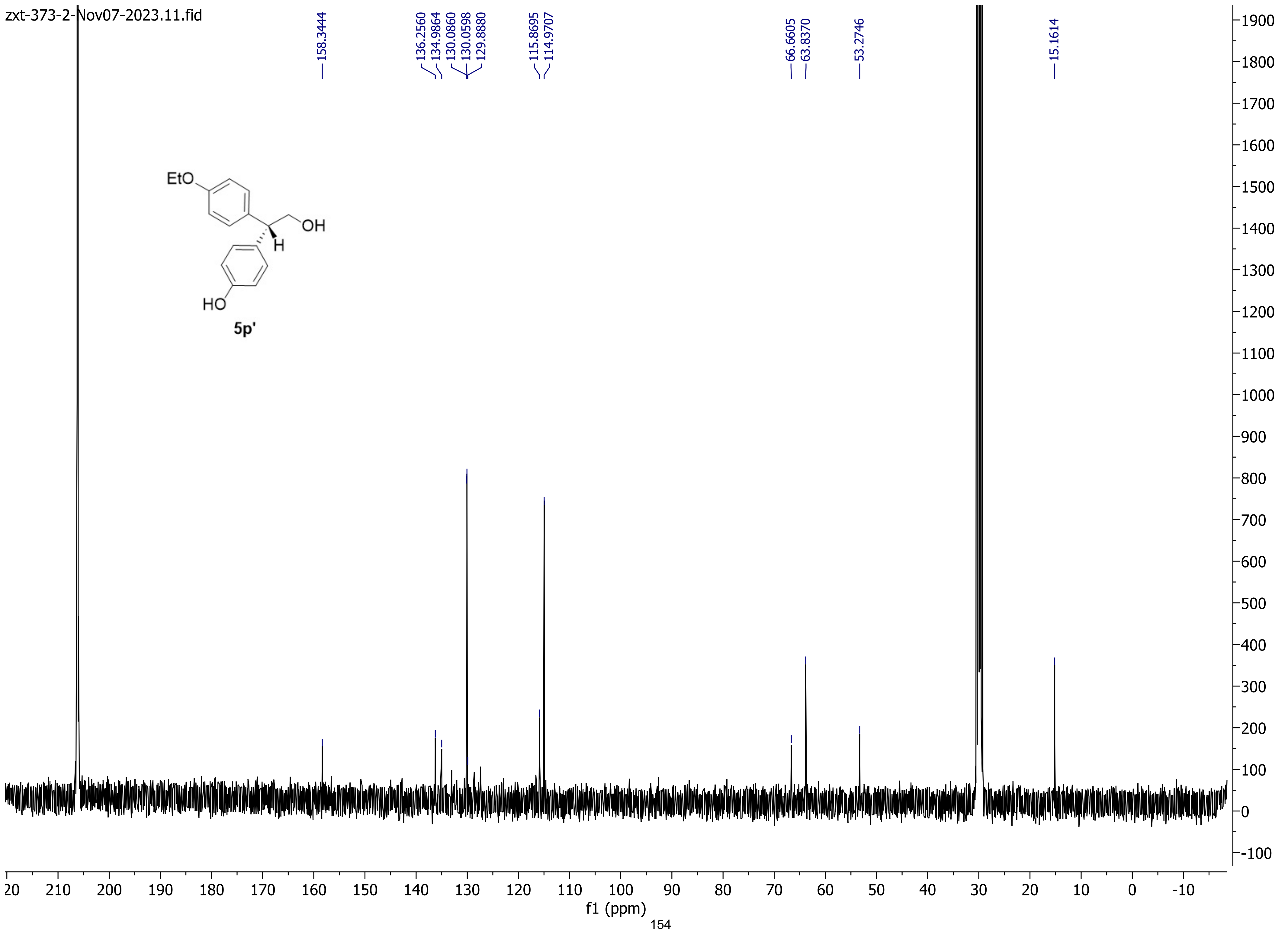
zxt-373-2-Nov07-2023.11.fid
158.3444
136.2560
134.9864
130.0860
130.0598
129.8880
115.8695
114.9707
66.6605
63.8370
53.2746
15.1614
EtO
OH
H
HO
5p'
f1 (ppm)

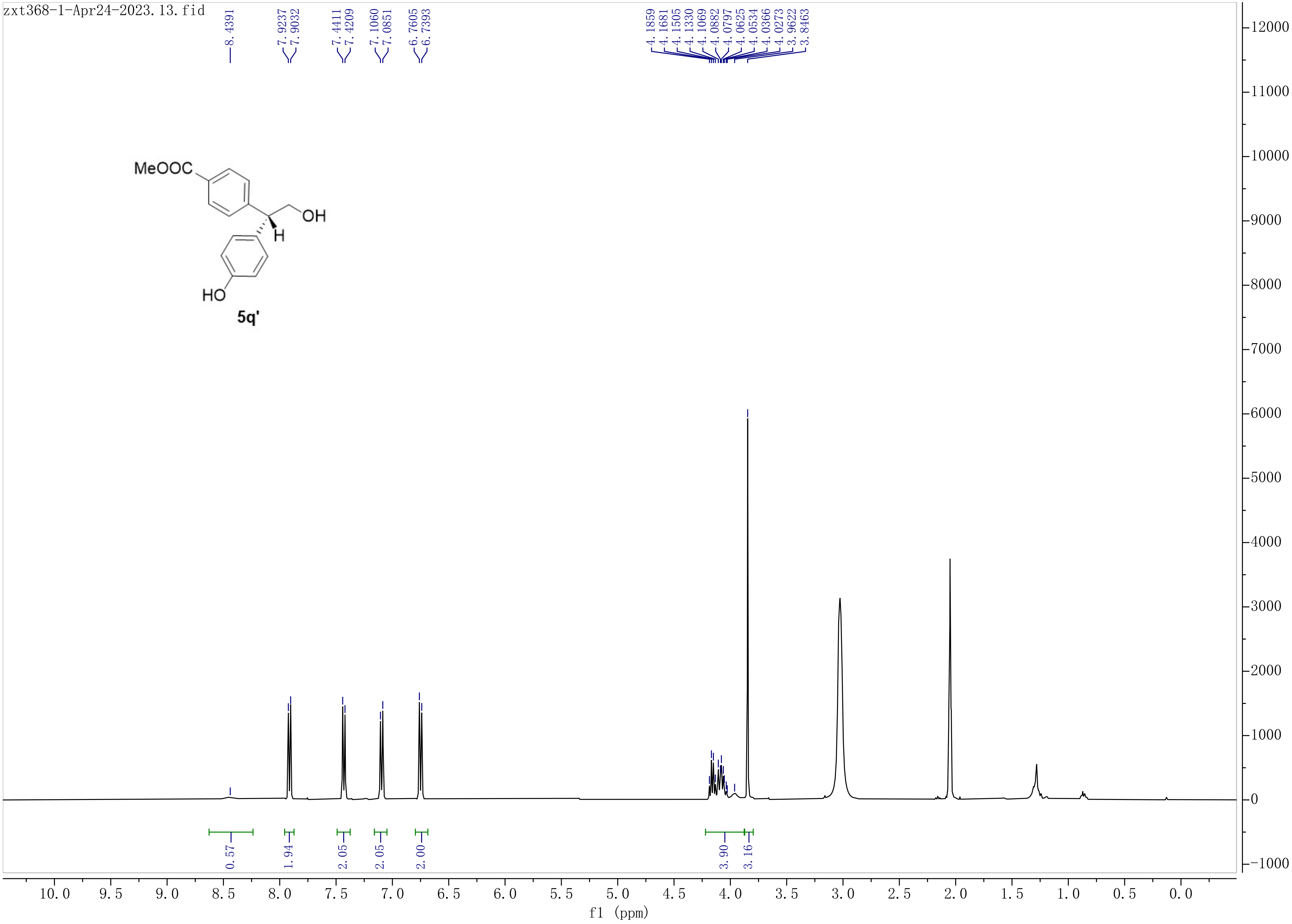
zxt368-1-Apr24-2023.13.fid
8.4391
7.9237
7.9032
7.4411
7.4209
7.1060
7.0851
6.7605
6.7393
4.1859
4.1681
4.1505
4.1330
4.1069
4.0882
4.0797
4.0625
4.0534
4.0366
4.0273
3.9622
3.8463
MeOOC
OH
H
HO
5q'
0.57
1.94
2.05
2.05
2.00
3.90
3.16
f1 (ppm)

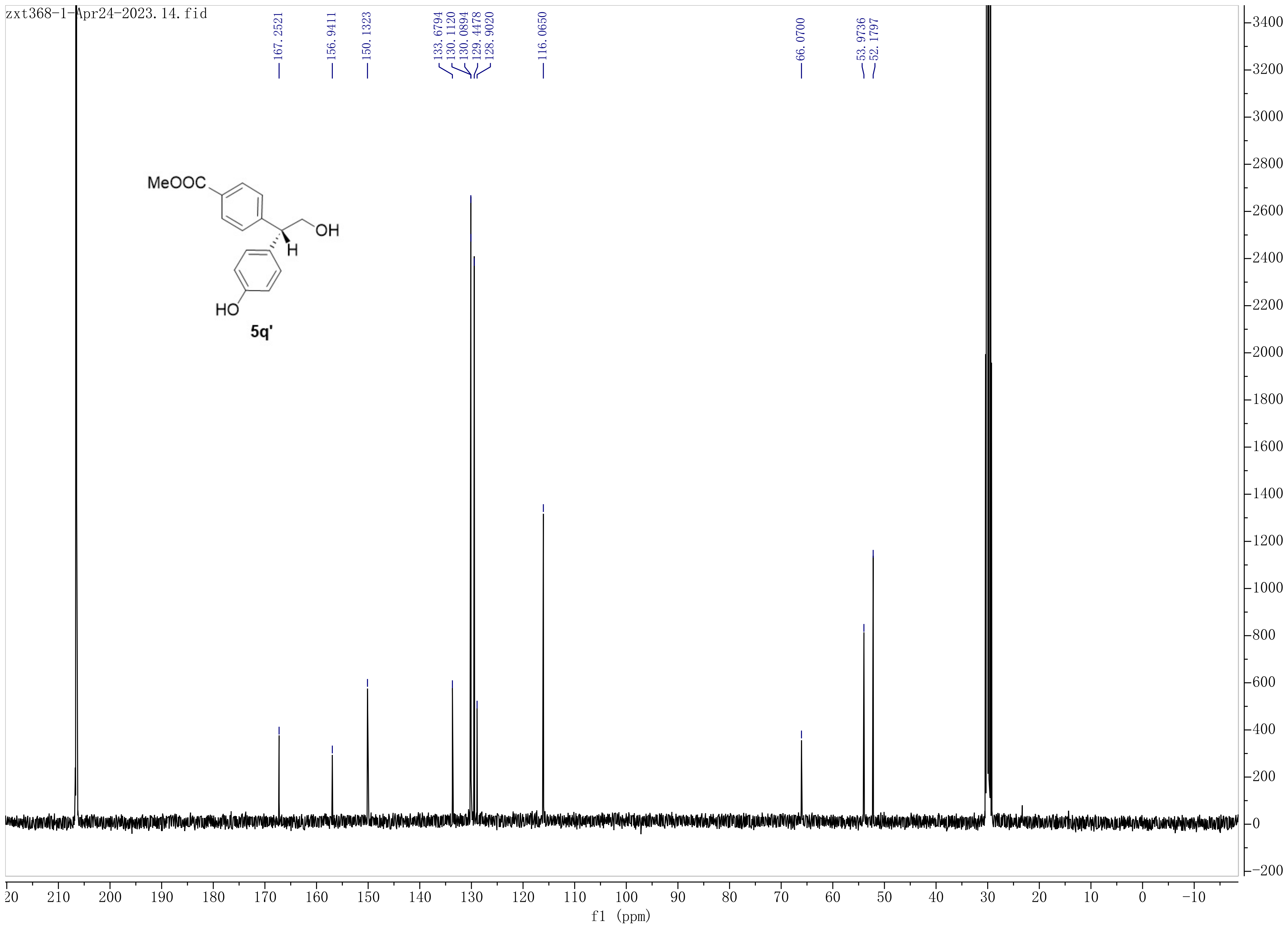
zxt368-1-Apr24-2023.14.fid
167.2521
156.9411
150.1323
133.6794
130.1120
130.0894
129.4478
128.9020
116.0650
66.0700
53.9736
52.1797
MeOOC
OH
H
HO
5q'
f1 (ppm)

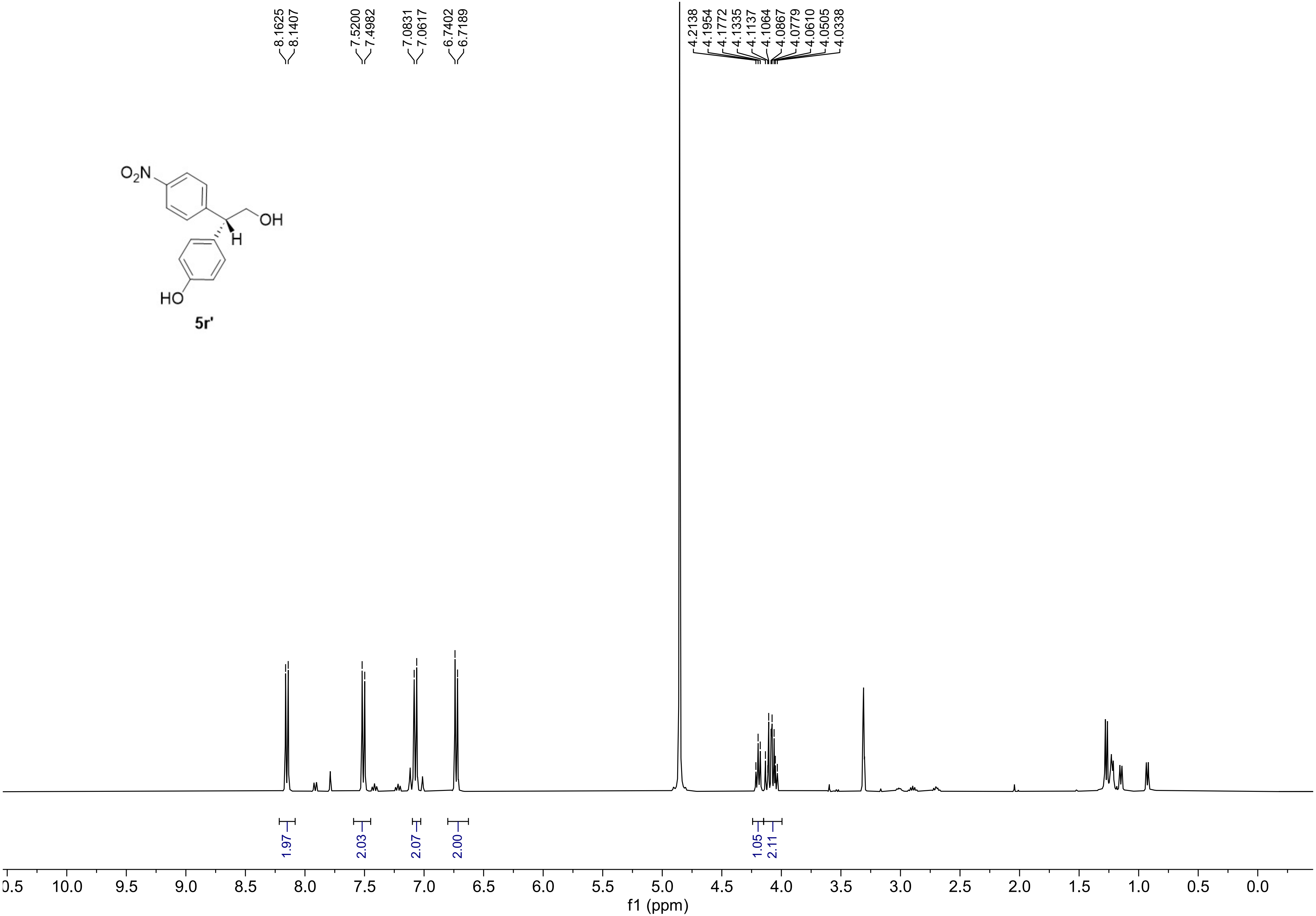
8.1625
8.1407
7.5200
7.4982
7.0831
7.0617
6.7402
6.7189
4.2138
4.1954
4.1772
4.1335
4.1137
4.1064
4.0867
4.0779
4.0610
4.0505
4.0338
O_2N
OH
H
HO
5r'
1.97
2.03
2.07
2.00
1.05
2.11
f1 (ppm)

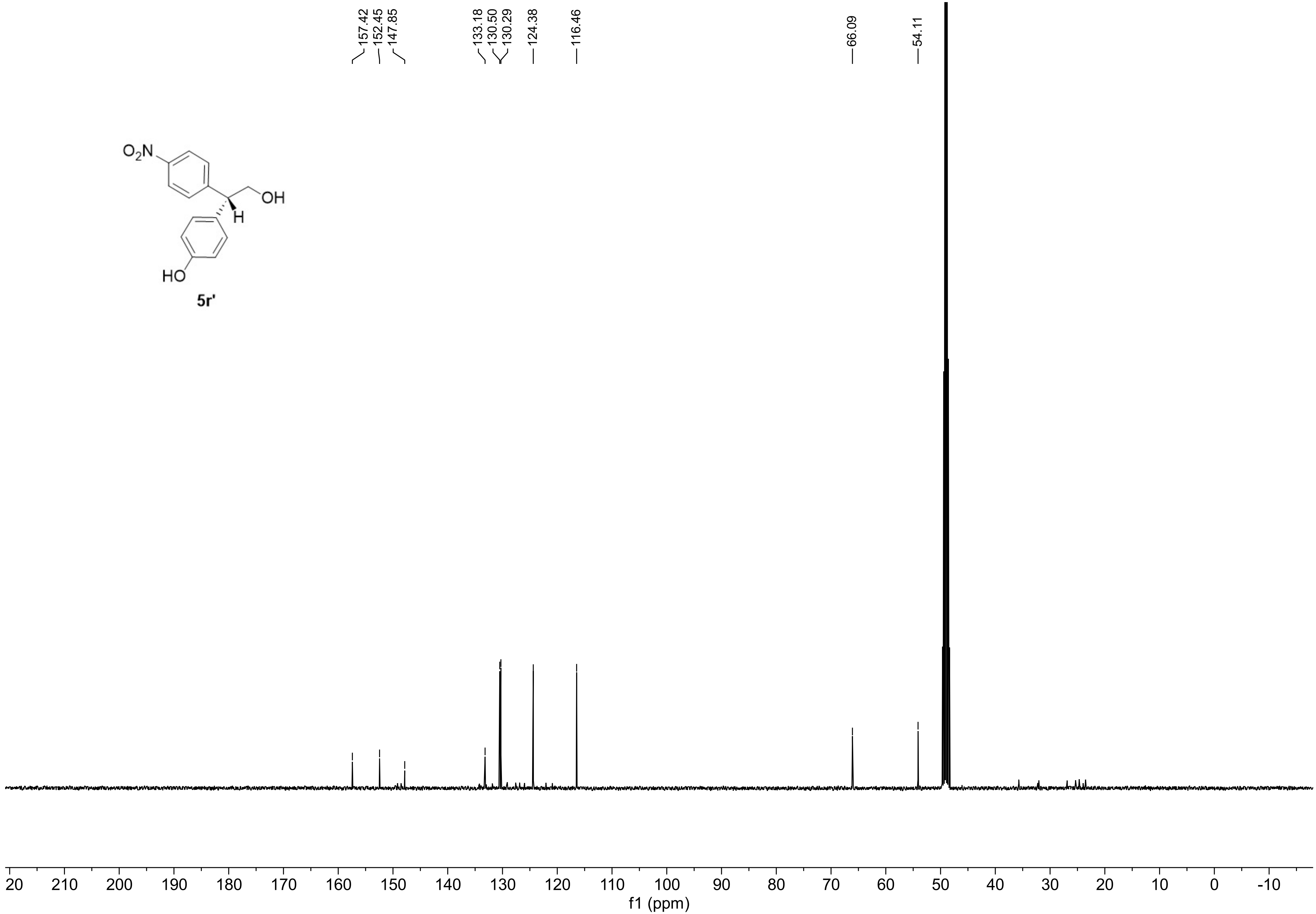
157.42
152.45
147.85
133.18
130.50
130.29
124.38
116.46
66.09
54.11
O2N
OH
H
HO
5r'
220 210 200 190 180 170 160 150 140 130 120 110 100 90 80 70 60 50 40 30 20 10 0 -10
f1 (ppm)

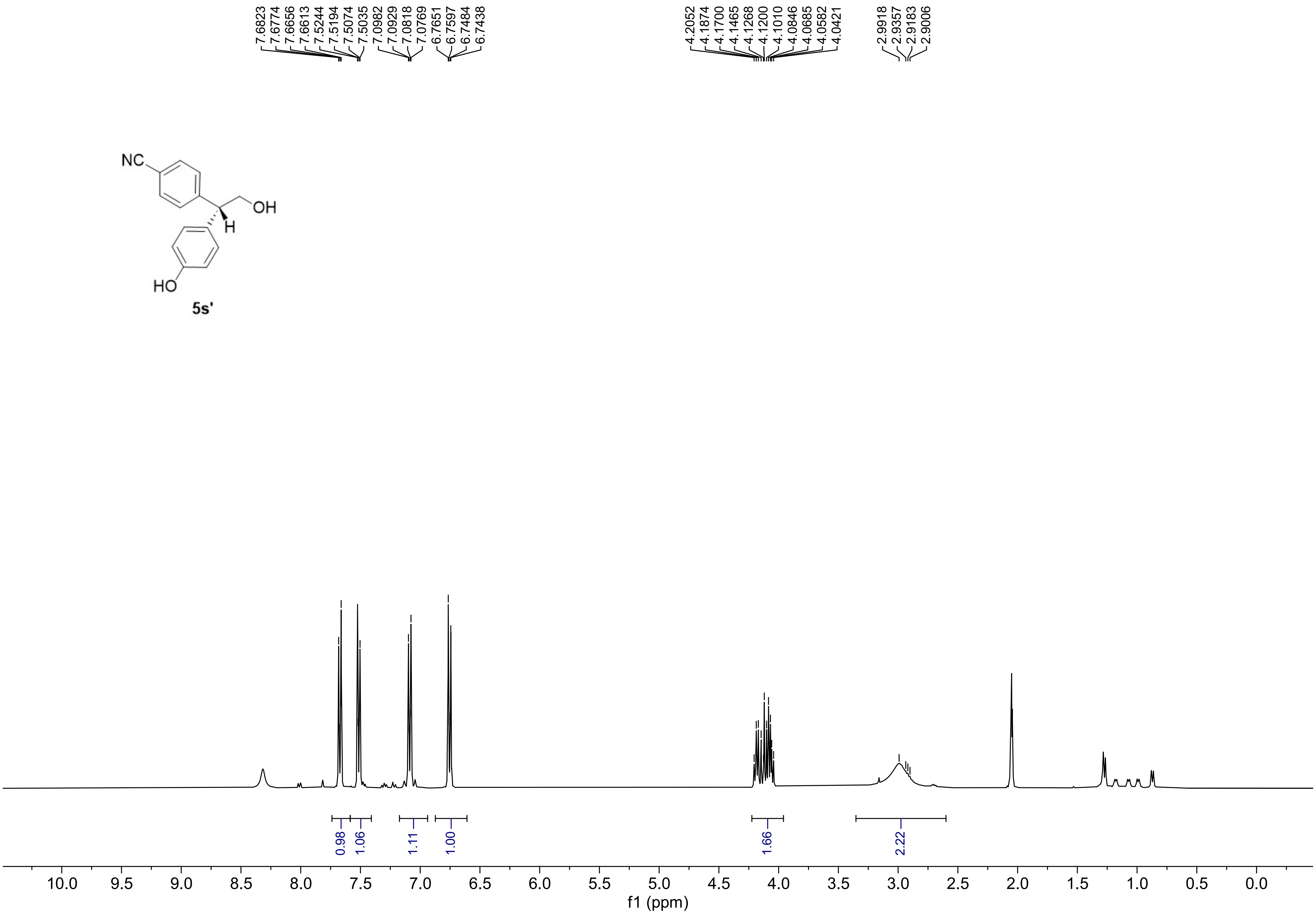
7.6823
7.6774
7.6656
7.6613
7.5244
7.5194
7.5074
7.5035
7.0982
7.0929
7.0818
7.0769
6.7651
6.7597
6.7484
6.7438
4.2052
4.1874
4.1700
4.1465
4.1268
4.1200
4.1010
4.0846
4.0685
4.0582
4.0421
2.9918
2.9357
2.9183
2.9006
NC
OH
H
HO
5s'
0.98
1.06
1.11
1.00
1.66
2.22
10.0
9.5
9.0
8.5
8.0
7.5
7.0
6.5
6.0
5.5
5.0
4.5
4.0
3.5
3.0
2.5
2.0
1.5
1.0
0.5
0.0
f1 (ppm)

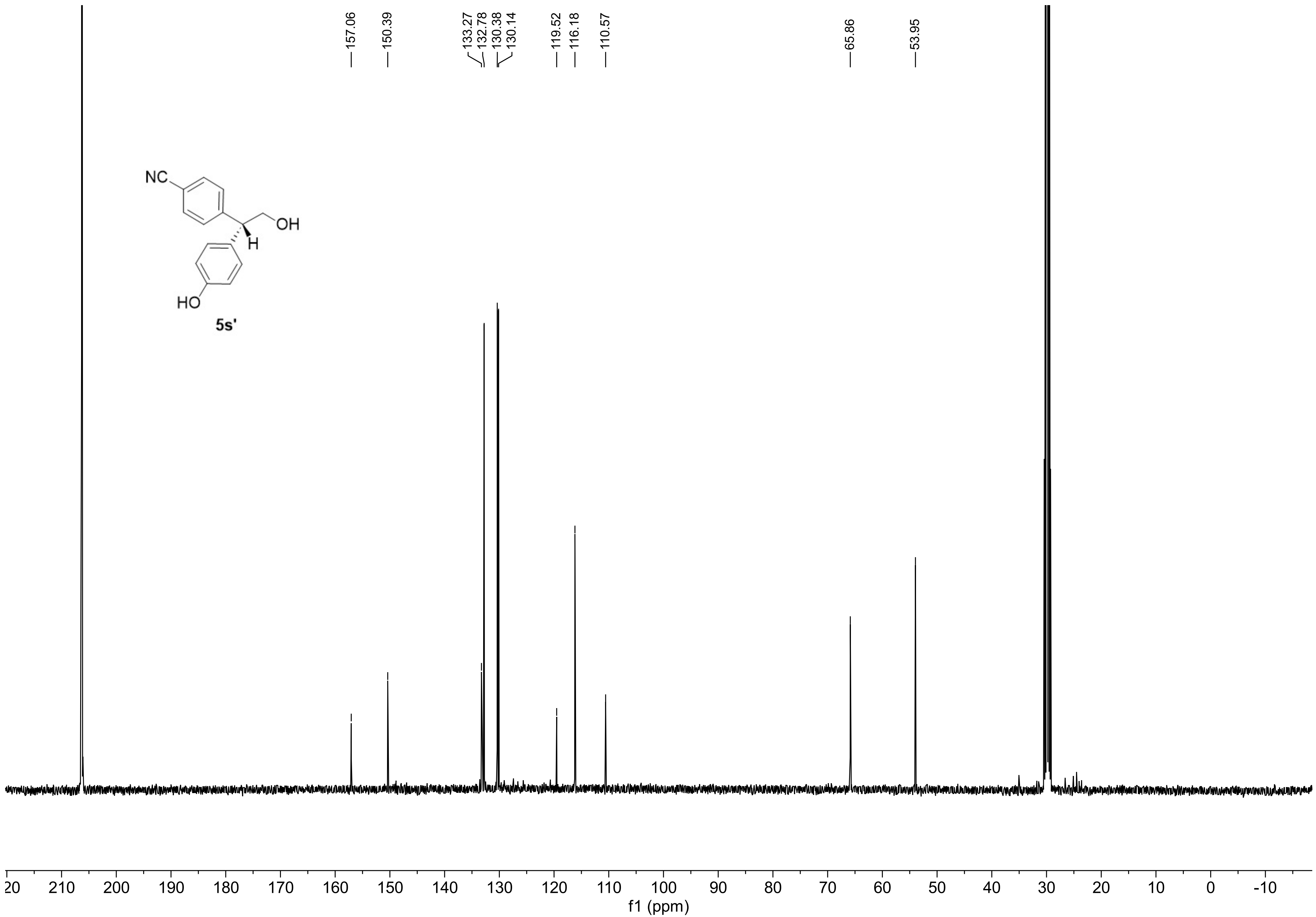
157.06
150.39
133.27
132.78
130.38
130.14
119.52
116.18
110.57
65.86
53.95
NC
OH
H
HO
5s'
20
210
200
190
180
170
160
150
140
130
120
110
100
90
80
70
60
50
40
30
20
10
0
-10
f1 (ppm)

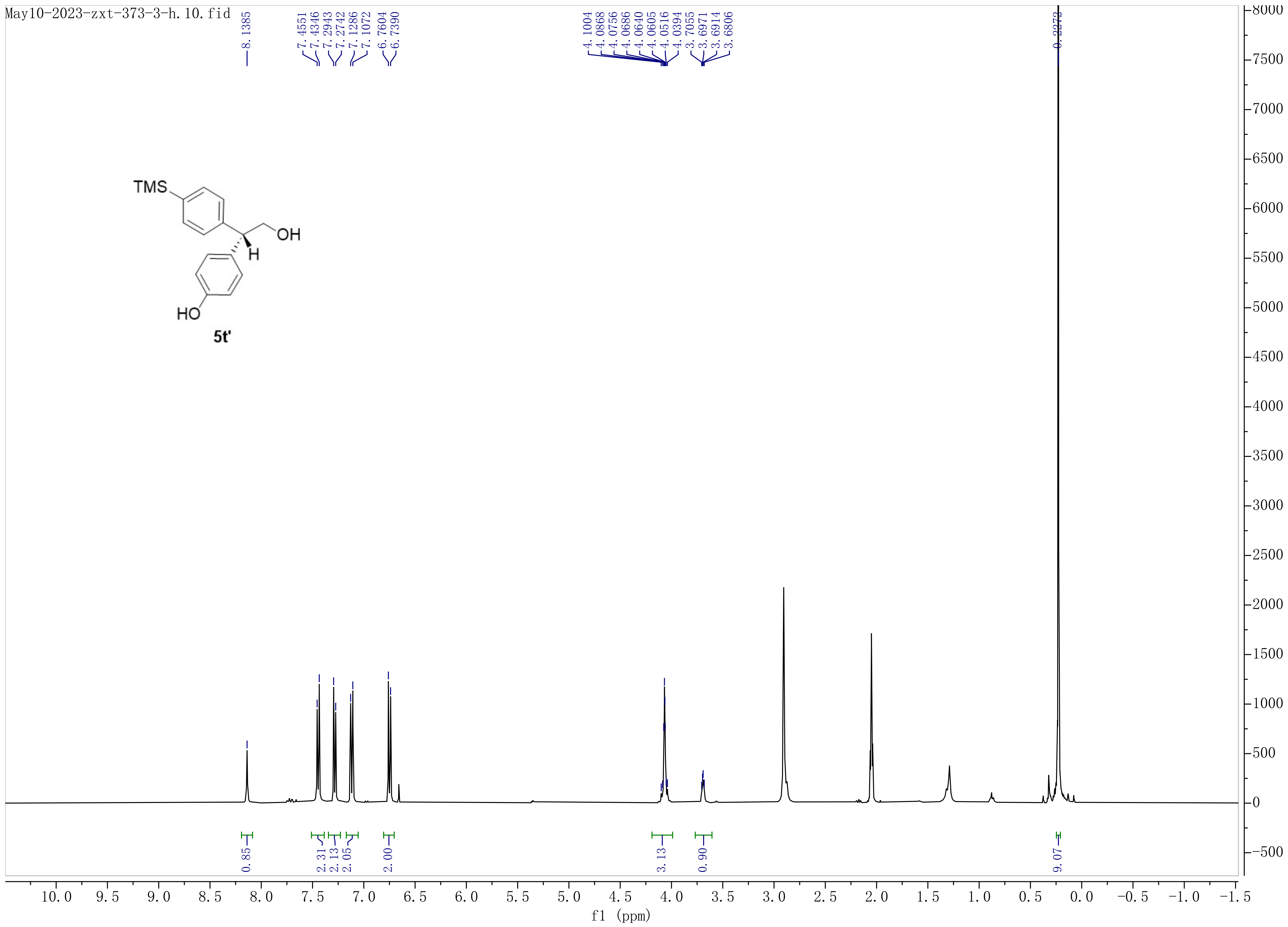
May10-2023-zxt-373-3-h.10.fid
8.1385
7.4551
7.4346
7.2943
7.2742
7.1286
7.1072
6.7604
6.7390
4.1004
4.0868
4.0756
4.0686
4.0640
4.0605
4.0516
4.0394
3.7055
3.6971
3.6914
3.6806
0.2272
TMS
OH
H
HO
5t'
0.85
2.31
2.13
2.05
2.00
3.13
0.90
9.07
f1 (ppm)

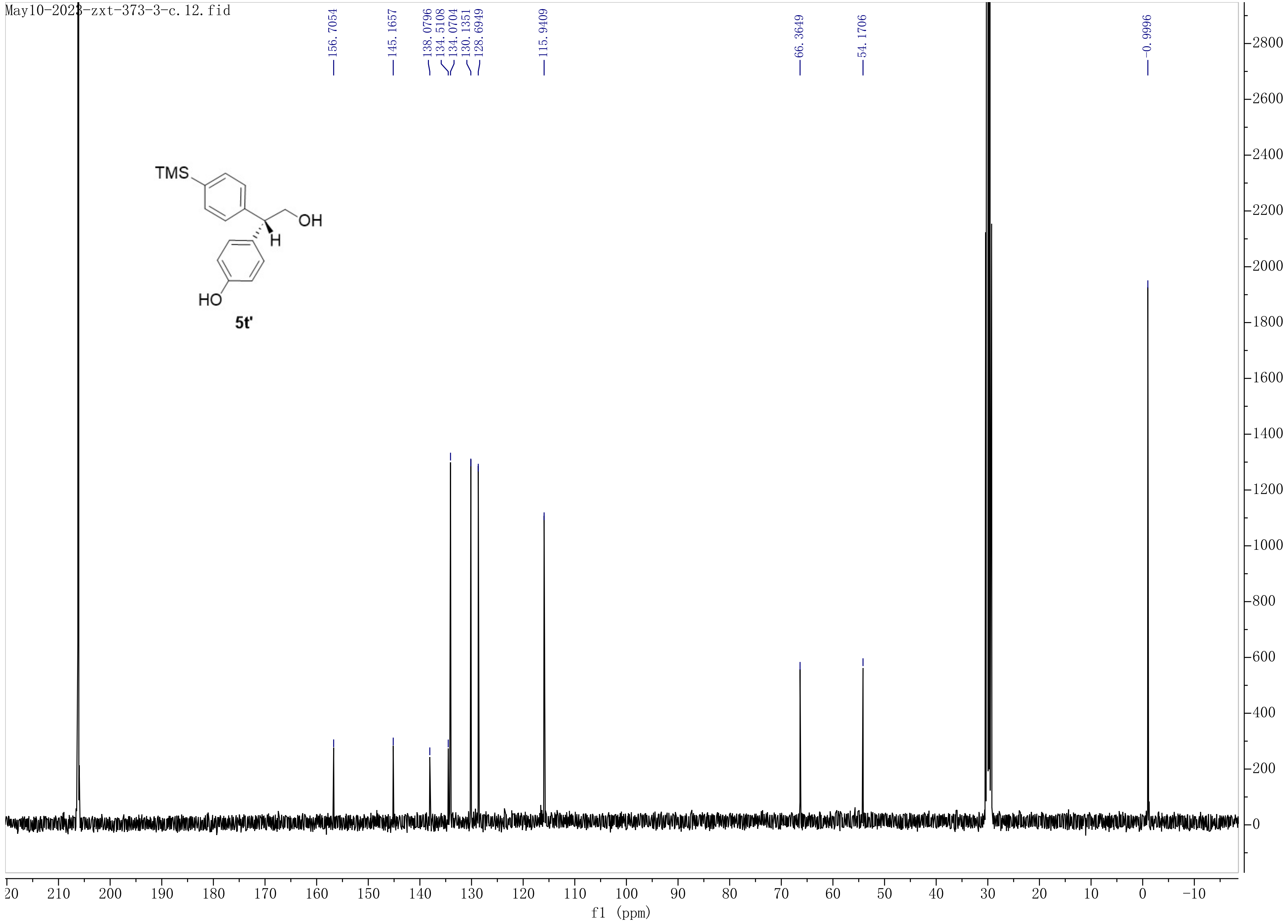
May10-2023-zxt-373-3-c.12.fid
156.7054
145.1657
138.0796
134.5108
134.0704
130.1351
128.6949
115.9409
66.3649
54.1706
-0.9996
TMS
OH
H
HO
5t'
f1 (ppm)

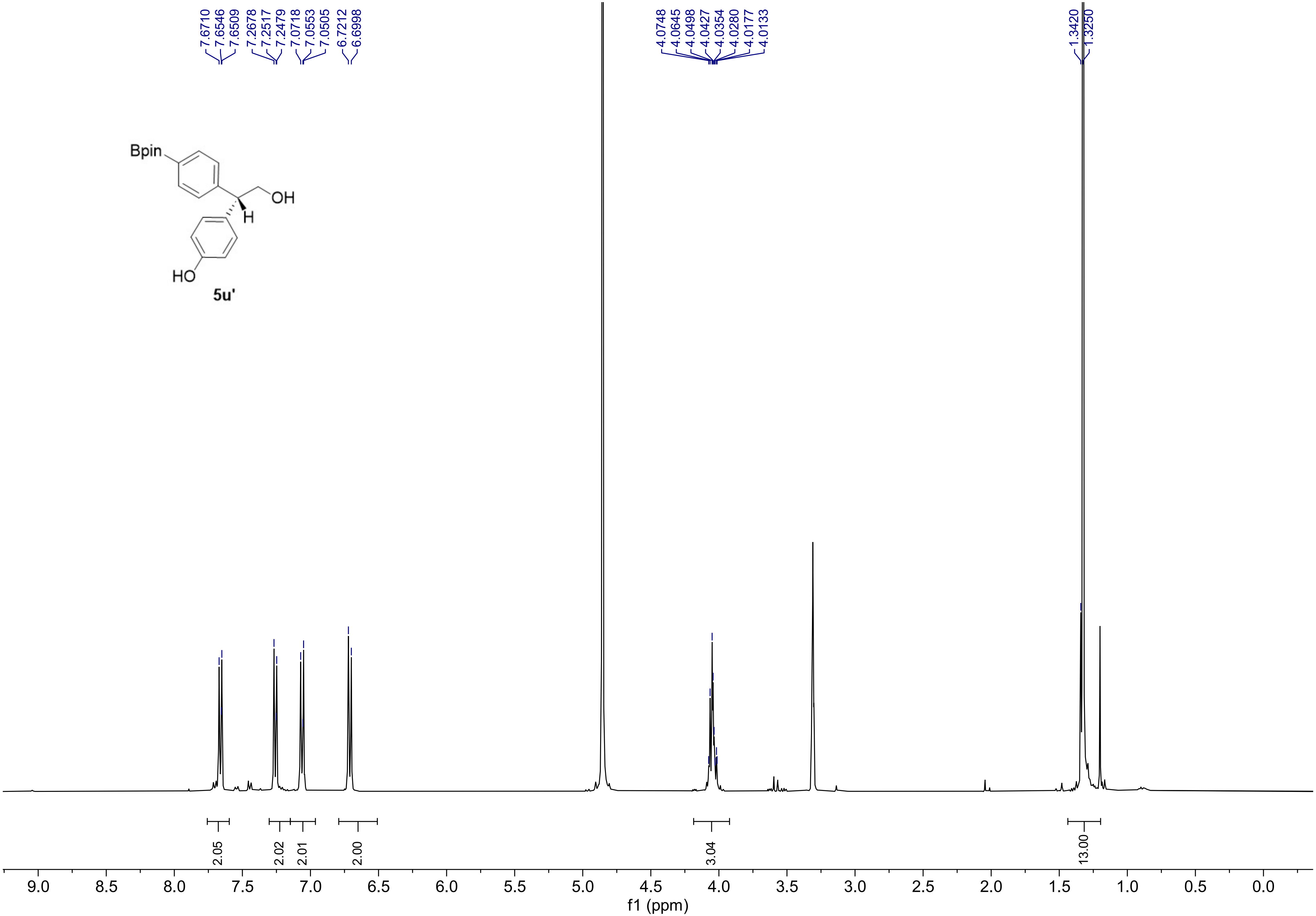

7.6710
7.6546
7.6509
7.2678
7.2517
7.2479
7.0718
7.0553
7.0505
6.7212
6.6998
4.0748
4.0645
4.0498
4.0427
4.0354
4.0280
4.0177
4.0133
1.3420
1.3250
Bpin
OH
H
HO
5u'
2.05
2.02
2.01
2.00
3.04
13.00
9.0
8.5
8.0
7.5
7.0
6.5
6.0
5.5
5.0
4.5
4.0
3.5
3.0
2.5
2.0
1.5
1.0
0.5
0.0
f1 (ppm)

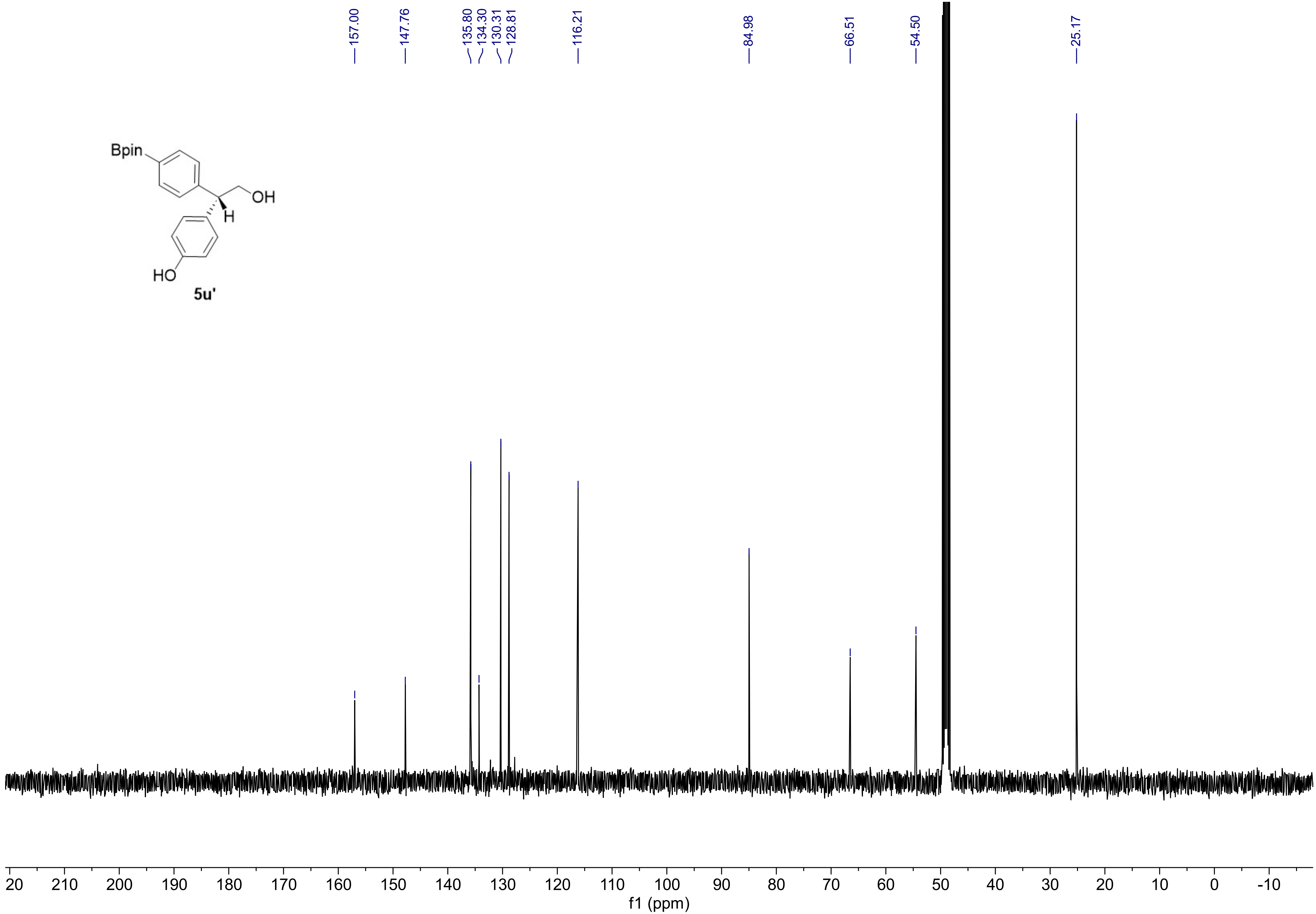
157.00
147.76
135.80
134.30
130.31
128.81
116.21
84.98
66.51
54.50
25.17
Bpin
OH
H
HO
5u'
20 210 200 190 180 170 160 150 140 130 120 110 100 90 80 70 60 50 40 30 20 10 0 -10
f1 (ppm)

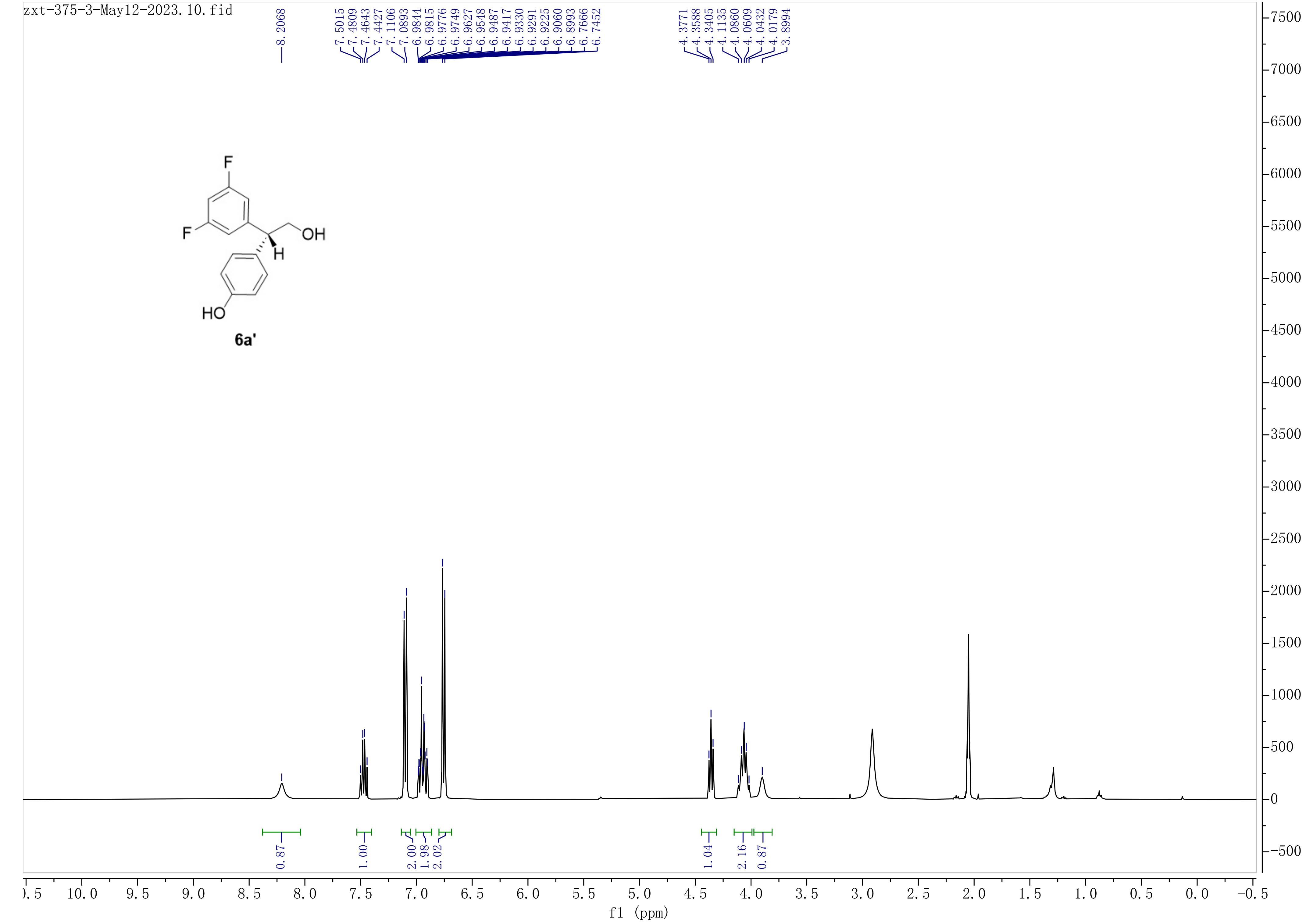
zxt-375-3-May12-2023.10.fid
8.2068
7.5015
7.4809
7.4643
7.4427
7.1106
7.0893
6.9844
6.9815
6.9776
6.9749
6.9627
6.9548
6.9487
6.9417
6.9330
6.9291
6.9225
6.9060
6.8993
6.7666
6.7452
4.3771
4.3588
4.3405
4.1135
4.0860
4.0609
4.0432
4.0179
3.8994
F
F
OH
H
HO
6a'
0.87
1.00
2.00
1.98
2.02
1.04
2.16
0.87
f1 (ppm)

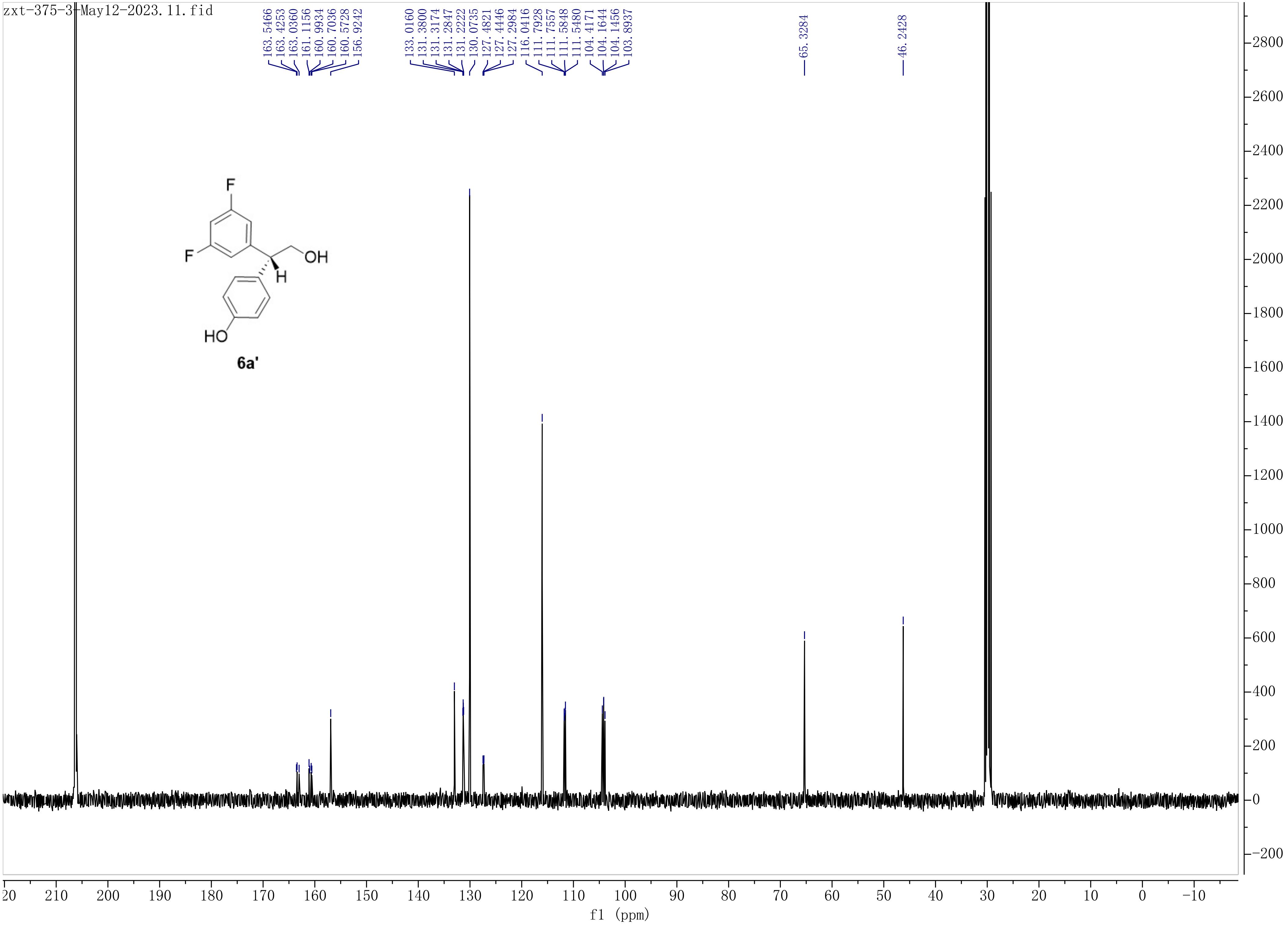
zxt-375-3-May12-2023.11.fid
163.5466
163.4253
163.0360
161.1156
160.9934
160.7036
160.5728
156.9242
133.0160
131.3800
131.3174
131.2847
131.2222
130.0735
127.4821
127.4446
127.2984
116.0416
111.7928
111.7557
111.5848
111.5480
104.4171
104.1644
104.1456
103.8937
65.3284
46.2428
F
F
OH
H
HO
6a'
f1 (ppm)

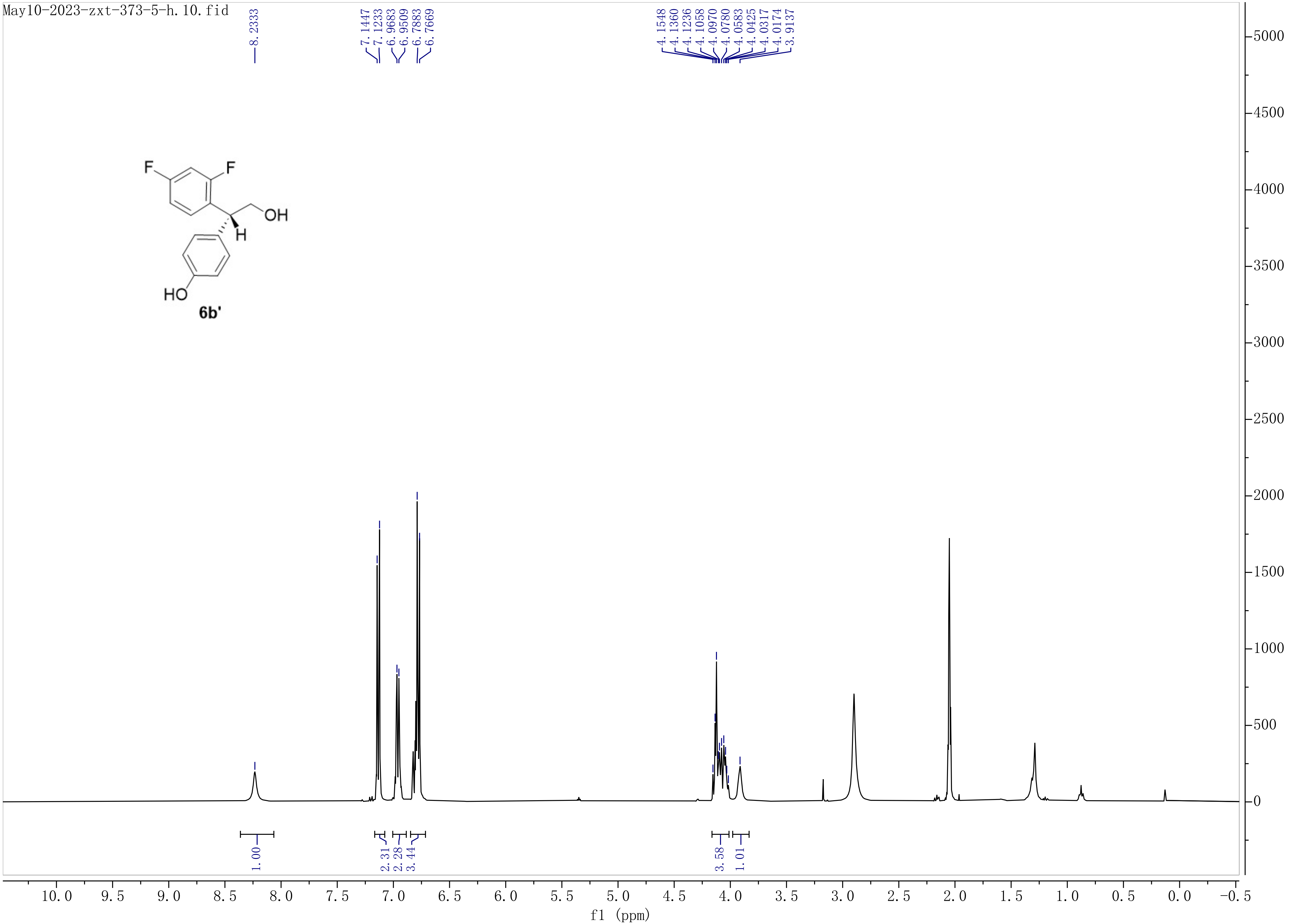
May10-2023-zxt-373-5-h.10.fid
8.2333
7.1447
7.1233
6.9683
6.9509
6.7883
6.7669
4.1548
4.1360
4.1236
4.1058
4.0970
4.0780
4.0583
4.0425
4.0317
4.0174
3.9137
F
F
OH
H
HO
6b'
1.00
2.31
2.28
3.44
3.58
1.01
f1 (ppm)

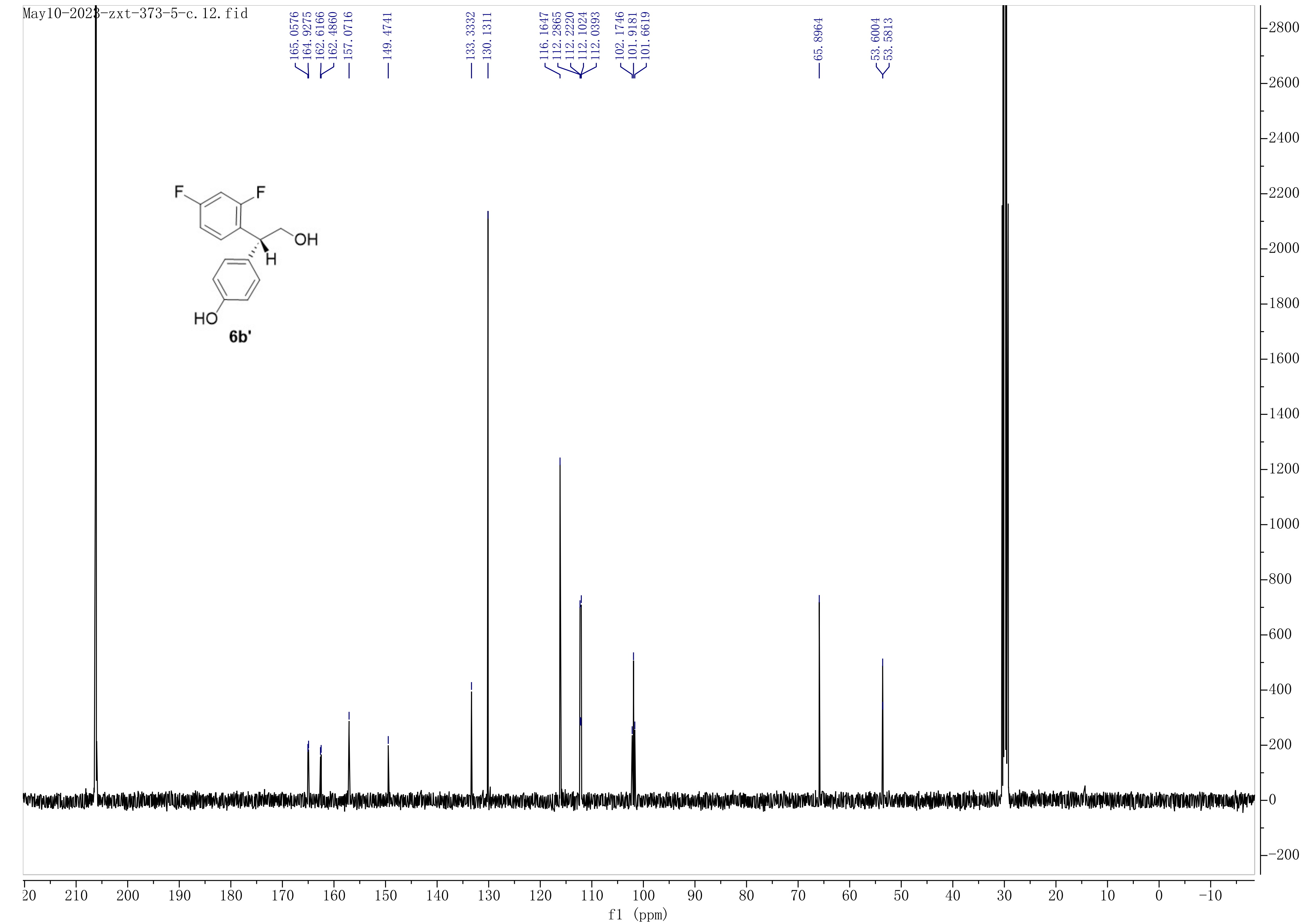
May10-2023-zxt-373-5-c.12.fid
165.0576
164.9275
162.6166
162.4860
157.0716
149.4741
133.3332
130.1311
116.1647
112.2865
112.2220
112.1024
112.0393
102.1746
101.9181
101.6619
65.8964
53.6004
53.5813
F
F
OH
H
HO
6b'
f1 (ppm)

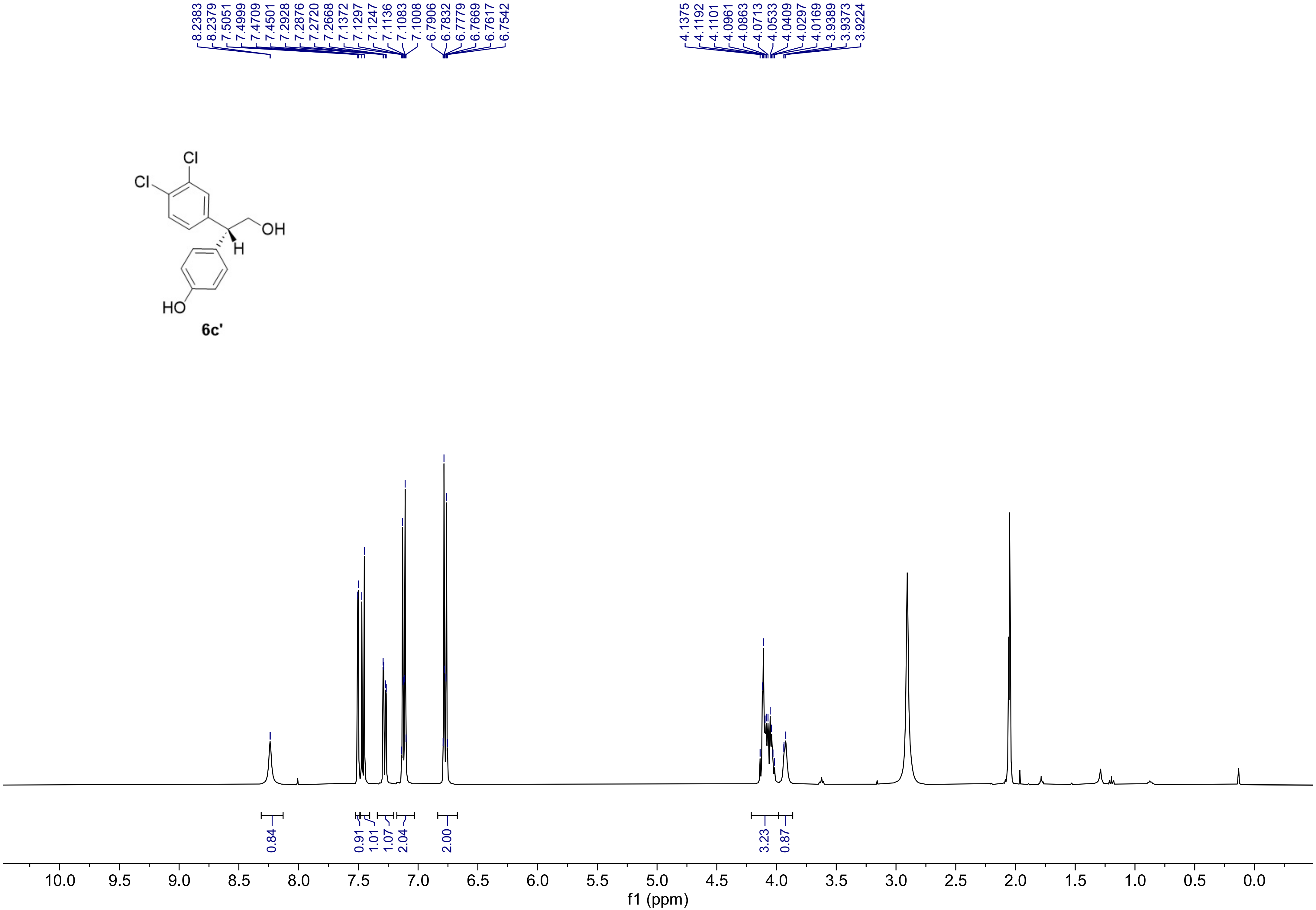
8.2383
8.2379
7.5051
7.4999
7.4709
7.4501
7.2928
7.2876
7.2720
7.2668
7.1372
7.1297
7.1247
7.1136
7.1083
7.1008
6.7906
6.7832
6.7779
6.7669
6.7617
6.7542
4.1375
4.1192
4.1101
4.0961
4.0863
4.0713
4.0533
4.0409
4.0297
4.0169
3.9389
3.9373
3.9224
Cl
Cl
OH
H
HO
6c'
0.84
0.91
1.01
1.07
2.04
2.00
3.23
0.87
10.0 9.5 9.0 8.5 8.0 7.5 7.0 6.5 6.0 5.5 5.0 4.5 4.0 3.5 3.0 2.5 2.0 1.5 1.0 0.5 0.0
f1 (ppm)

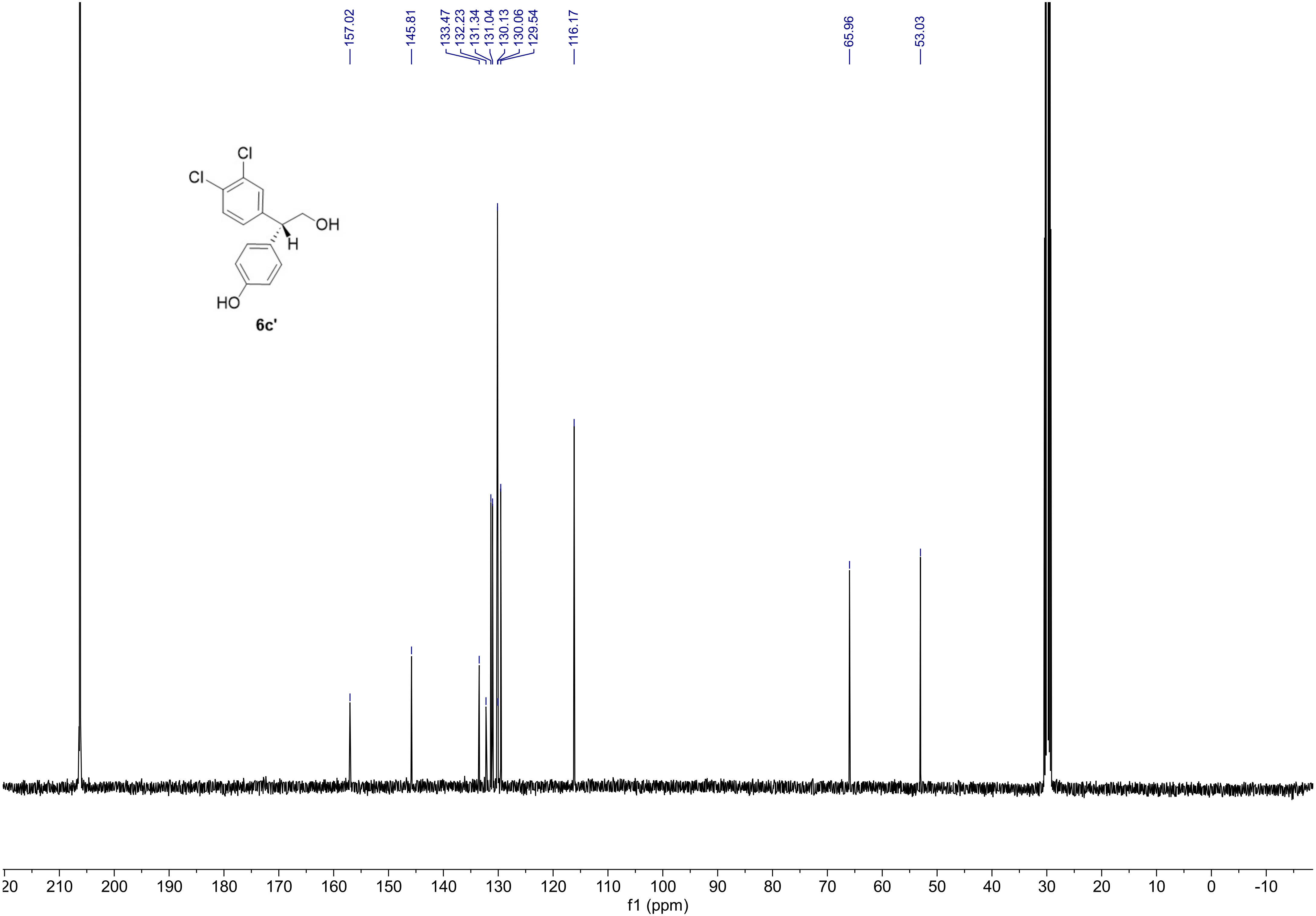
157.02
145.81
133.47
132.23
131.34
131.04
130.13
130.06
129.54
116.17
65.96
53.03
Cl
Cl
OH
H
HO
6c'
20 210 200 190 180 170 160 150 140 130 120 110 100 90 80 70 60 50 40 30 20 10 0 -10
f1 (ppm)

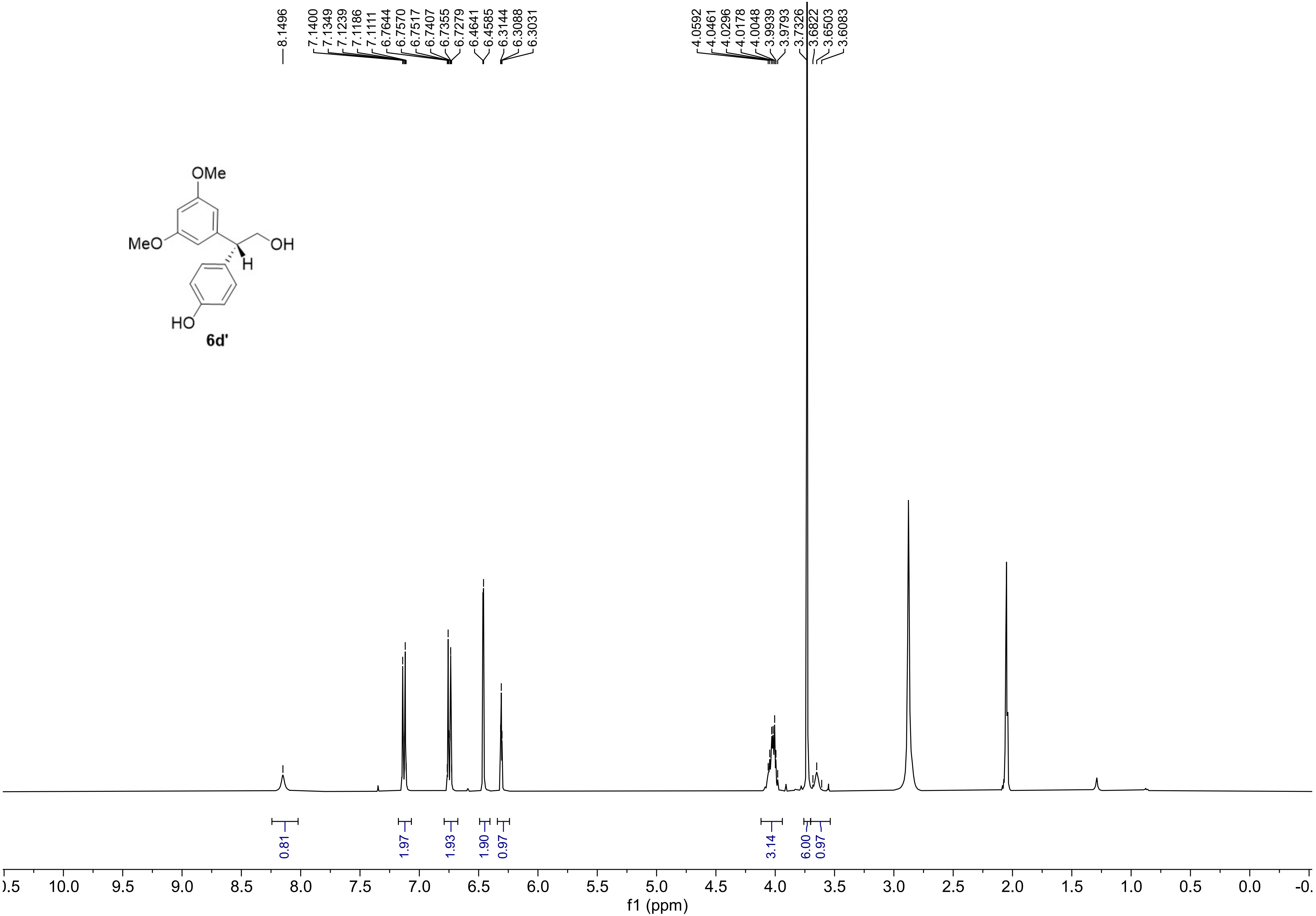
8.1496
7.1400
7.1349
7.1239
7.1186
7.1111
6.7644
6.7570
6.7517
6.7407
6.7355
6.7279
6.4641
6.4585
6.3144
6.3088
6.3031
4.0592
4.0461
4.0296
4.0178
4.0048
3.9939
3.9793
3.7326
3.6822
3.6503
3.6083
OMe
MeO
OH
H
HO
6d'
0.81
1.97
1.93
1.90
0.97
3.14
6.00
0.97
10.0
9.5
9.0
8.5
8.0
7.5
7.0
6.5
6.0
5.5
5.0
4.5
4.0
3.5
3.0
2.5
2.0
1.5
1.0
0.5
0.0
f1 (ppm)

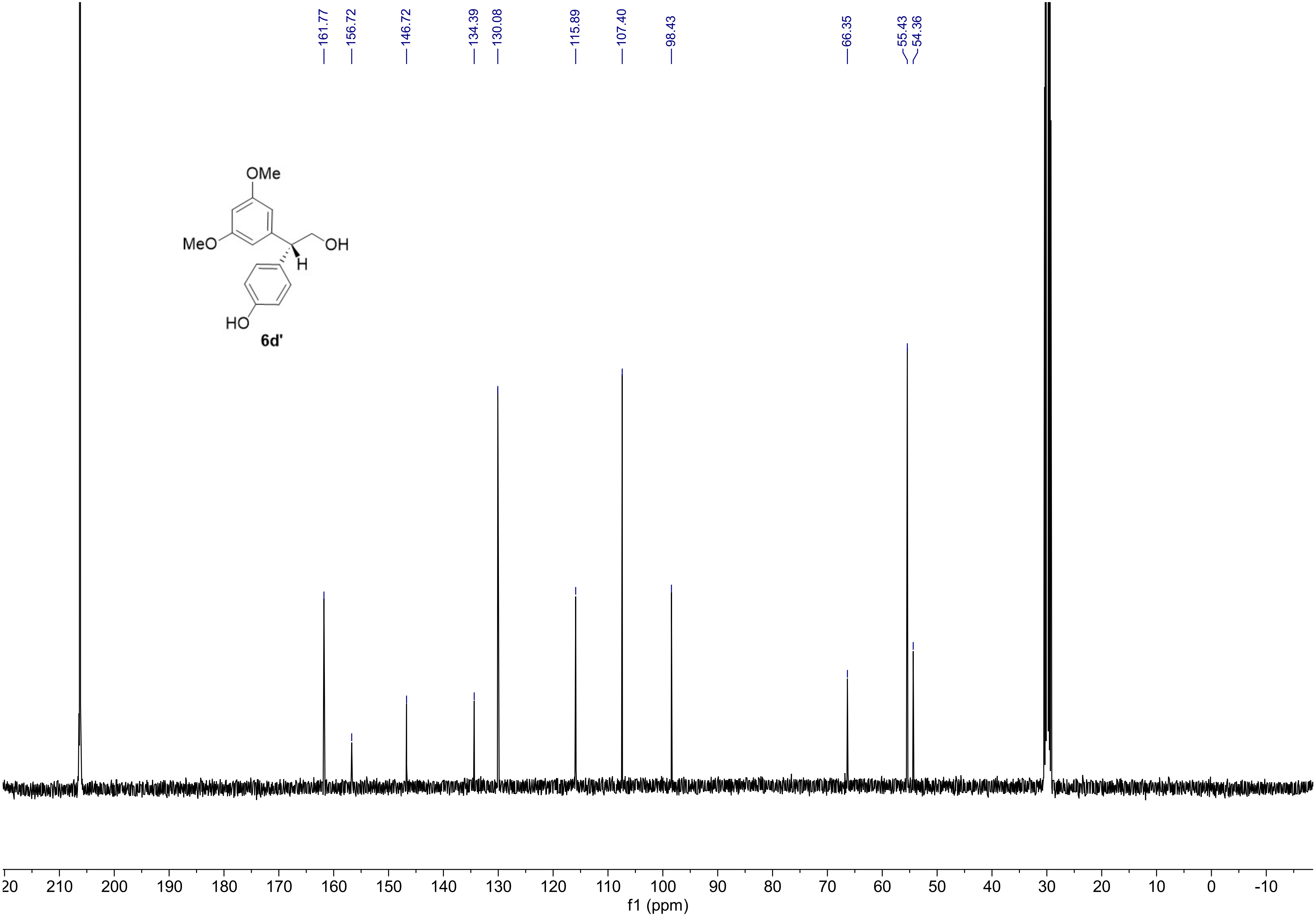
161.77
156.72
146.72
134.39
130.08
115.89
107.40
98.43
66.35
55.43
54.36
OMe
MeO
OH
H
HO
6d'
20
210
200
190
180
170
160
150
140
130
120
110
100
90
80
70
60
50
40
30
20
10
0
-10
f1 (ppm)

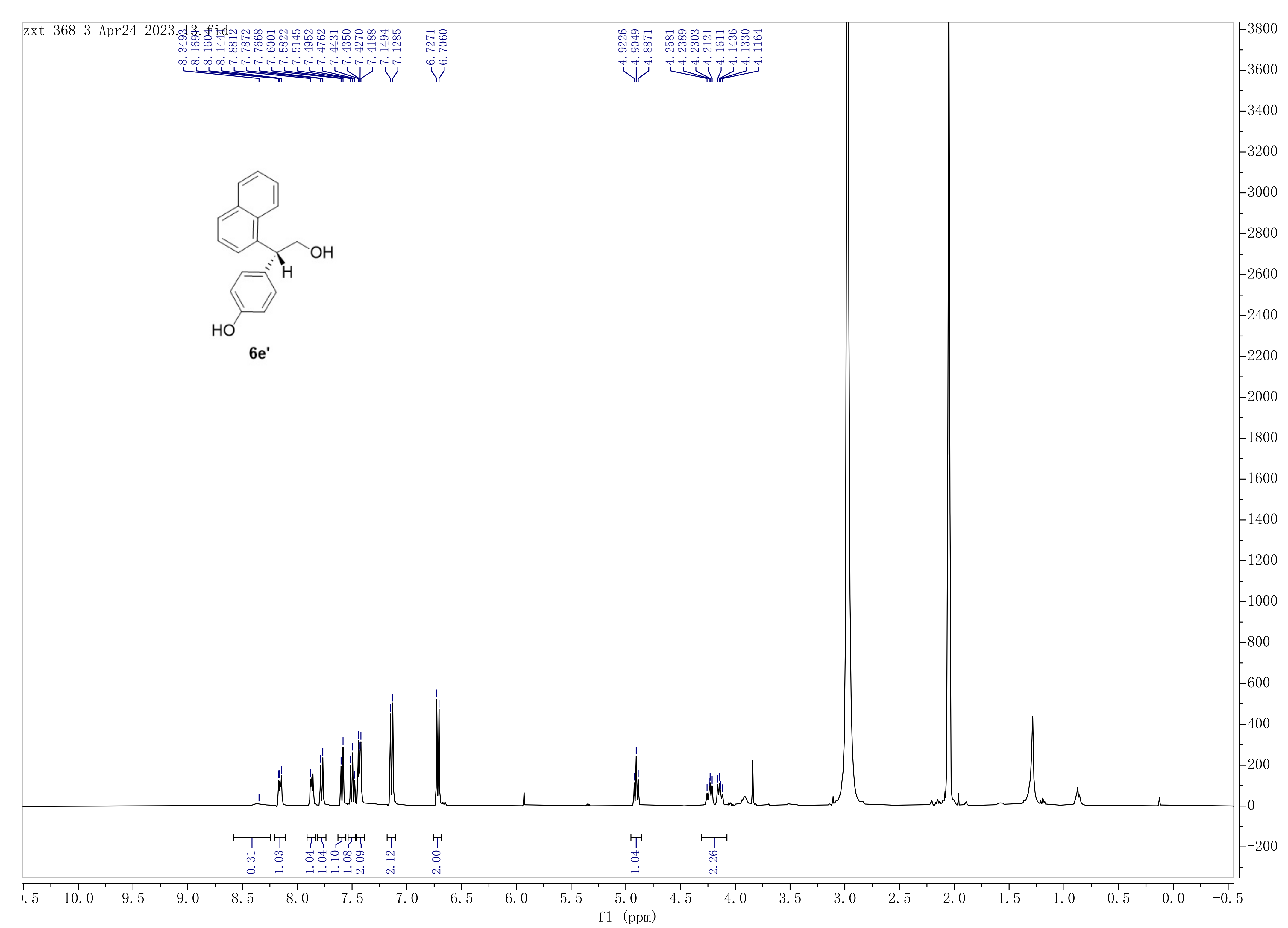
zxt-368-3-Apr24-2023.13.fid
6e'
OH
H
HO
f1 (ppm)

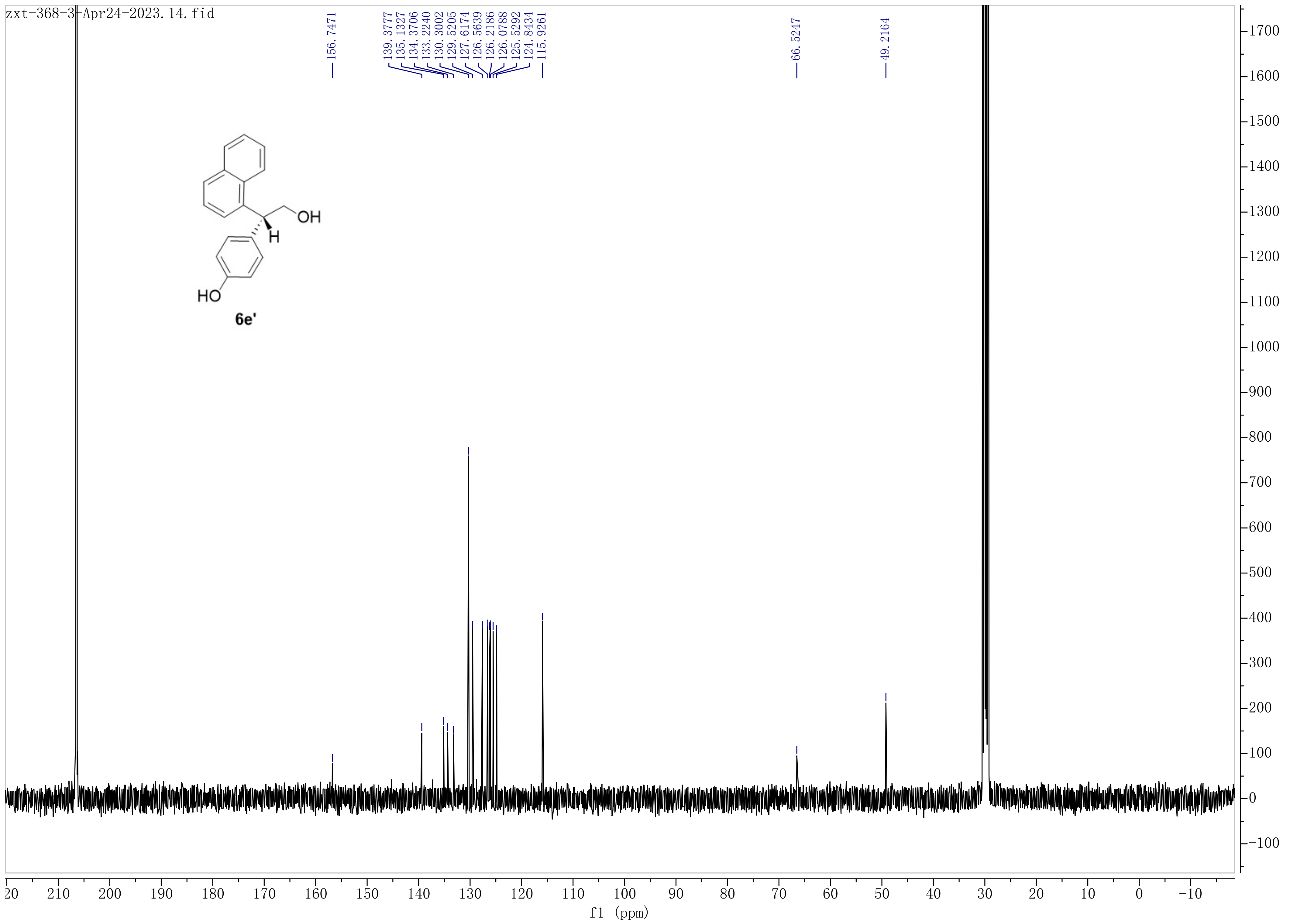
zxt-368-3-Apr24-2023.14.fid
156.7471
139.3777
135.1327
134.3706
133.2240
130.3002
129.5205
127.6174
126.5639
126.2186
126.0788
125.5292
124.8434
115.9261
66.5247
49.2164
OH
H
HO
6e'
f1 (ppm)

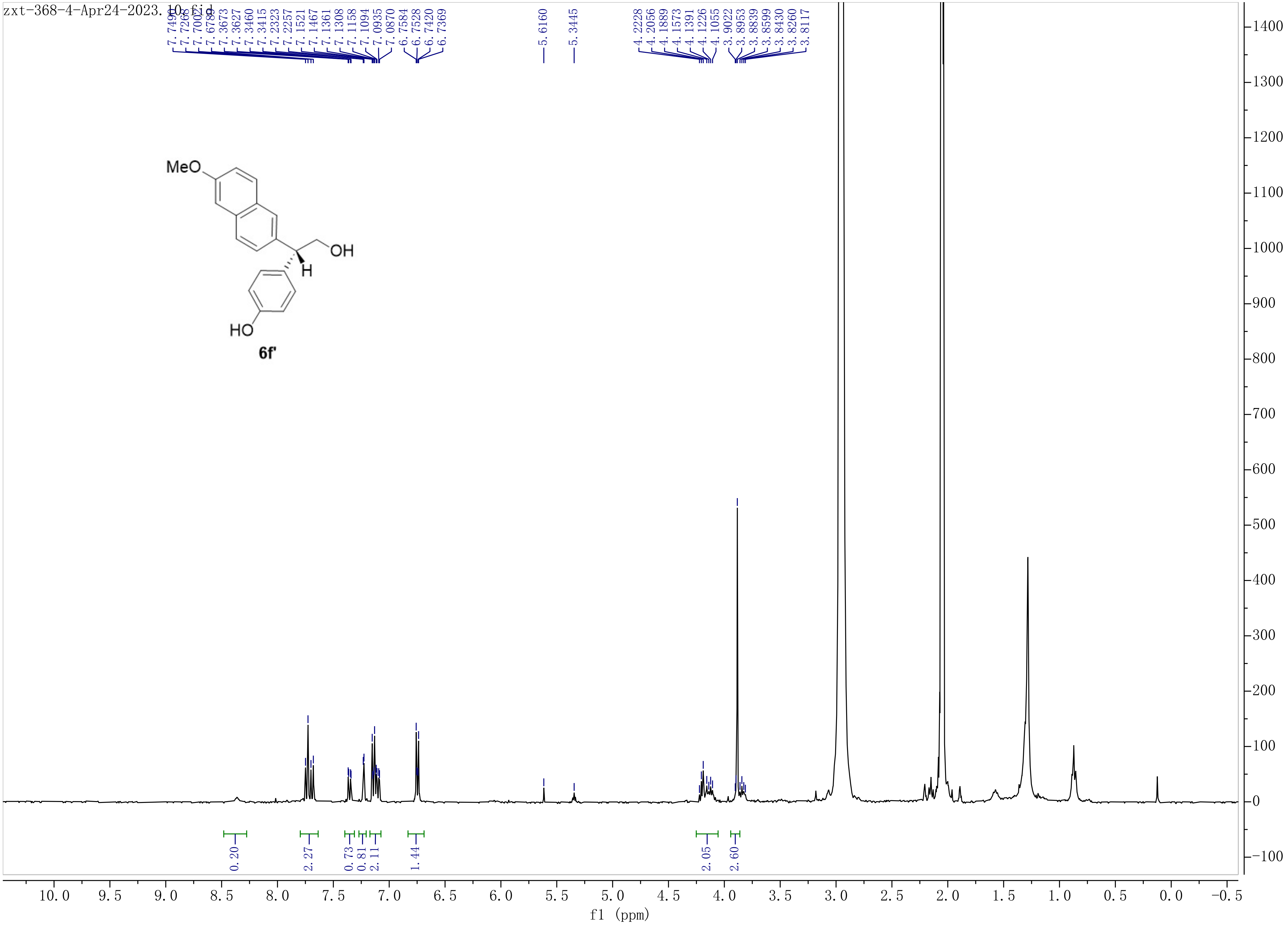
zxt-368-4-Apr24-2023.10.fid
7.6789
7.3673
7.3627
7.3460
7.3415
7.2323
7.2257
7.1521
7.1467
7.1361
7.1308
7.1158
7.1094
7.0935
7.0870
6.7584
6.7528
6.7420
6.7369
5.6160
5.3445
4.2228
4.2056
4.1889
4.1573
4.1391
4.1226
4.1055
3.9022
3.8953
3.8839
3.8599
3.8430
3.8260
3.8117
MeO
OH
H
HO
6f'
0.20
2.27
0.73
0.81
2.11
1.44
2.05
2.60
f1 (ppm)

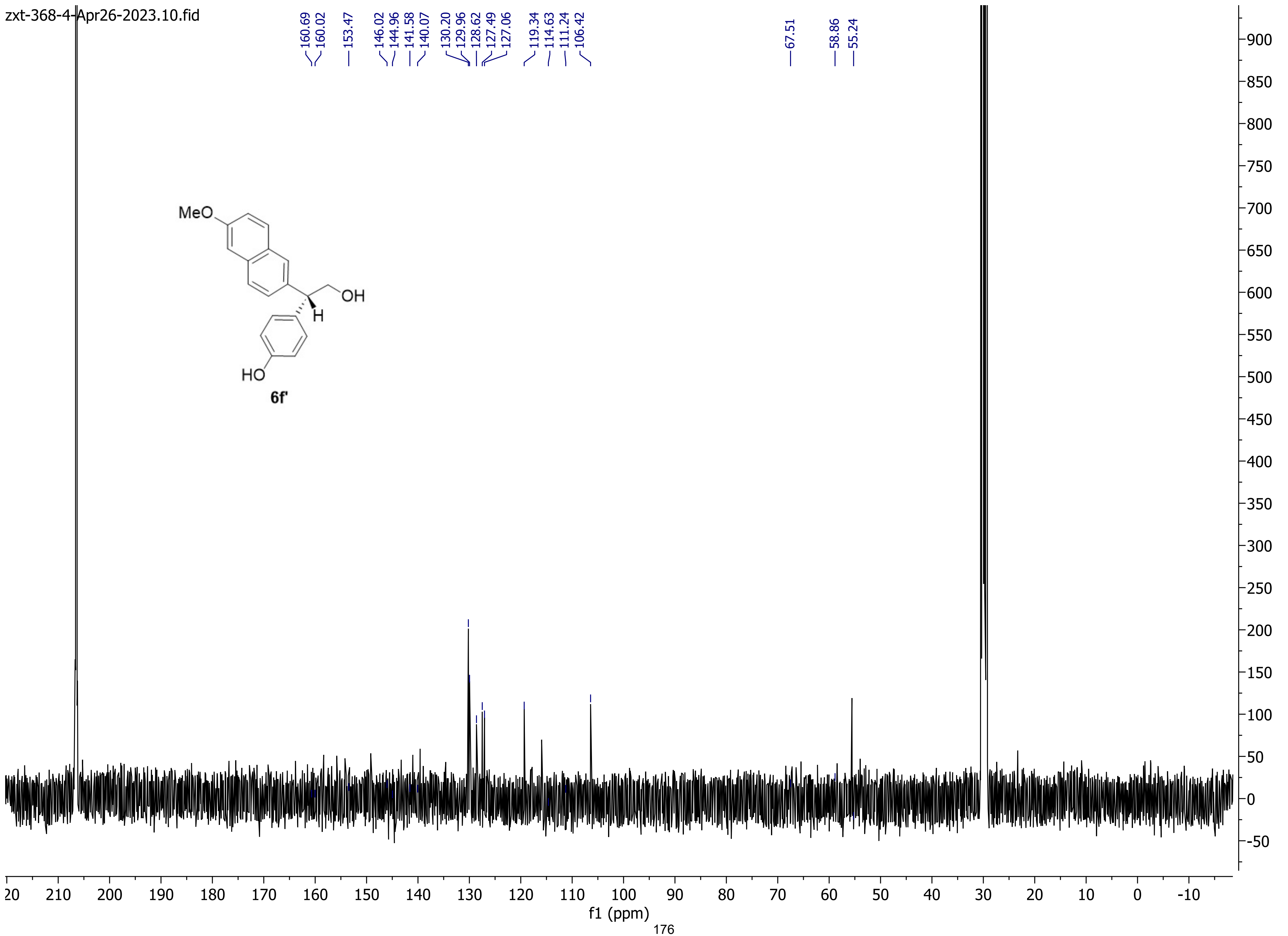
zxt-368-4-Apr26-2023.10.fid
160.69
160.02
153.47
146.02
144.96
141.58
140.07
130.20
129.96
128.62
127.49
127.06
119.34
114.63
111.24
106.42
67.51
58.86
55.24
MeO
OH
H
HO
6f'
f1 (ppm)

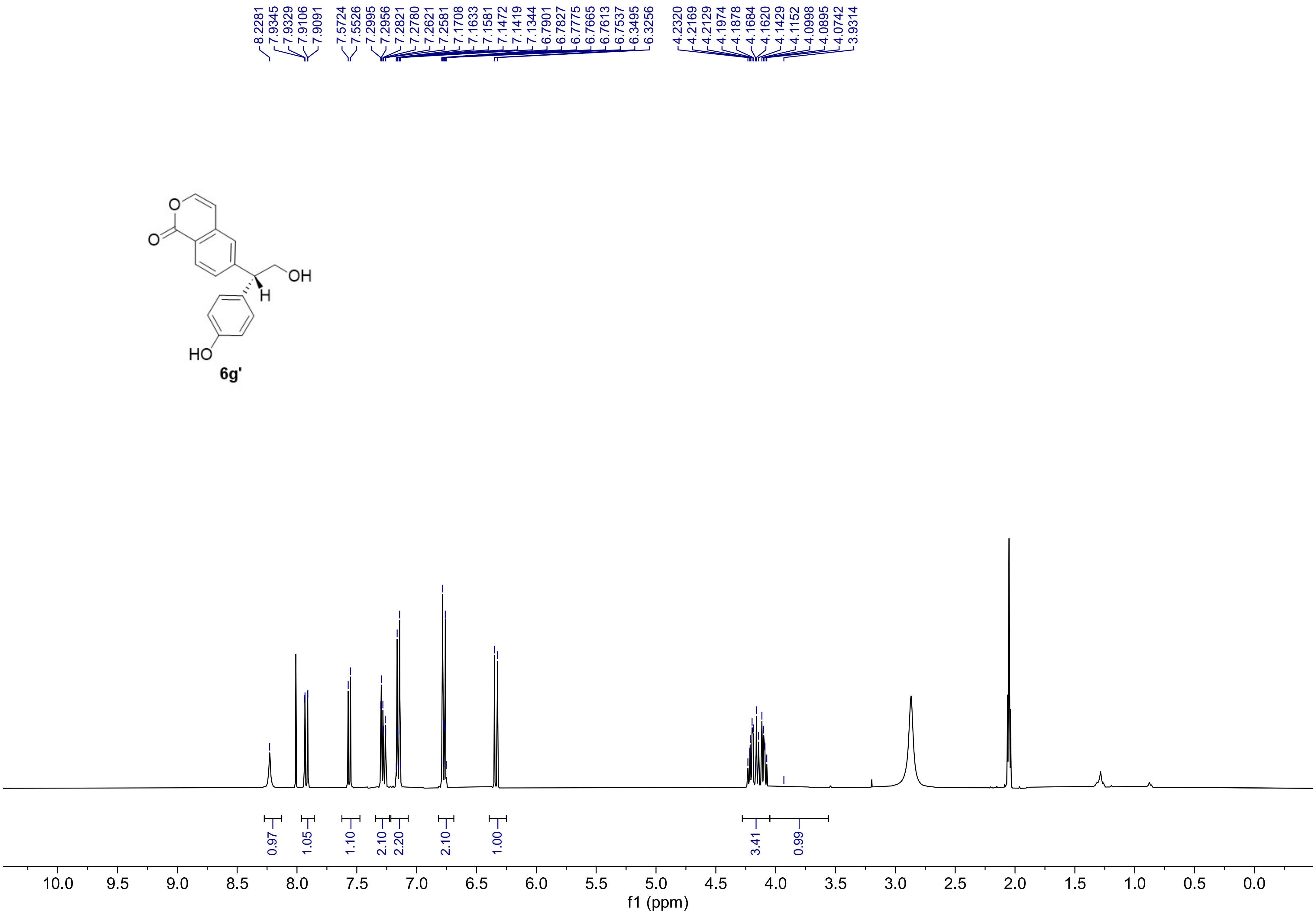

6g'
8.2281
7.9345
7.9329
7.9106
7.9091
7.5724
7.5526
7.2995
7.2956
7.2821
7.2780
7.2621
7.2581
7.1708
7.1633
7.1581
7.1472
7.1419
7.1344
6.7901
6.7827
6.7775
6.7665
6.7613
6.7537
6.3495
6.3256
4.2320
4.2169
4.2129
4.1974
4.1878
4.1684
4.1620
4.1429
4.1152
4.0998
4.0895
4.0742
3.9314
0.97
1.05
1.10
2.10
2.20
2.10
1.00
3.41
0.99
10.0
9.5
9.0
8.5
8.0
7.5
7.0
6.5
6.0
5.5
5.0
4.5
4.0
3.5
3.0
2.5
2.0
1.5
1.0
0.5
0.0
f1 (ppm)

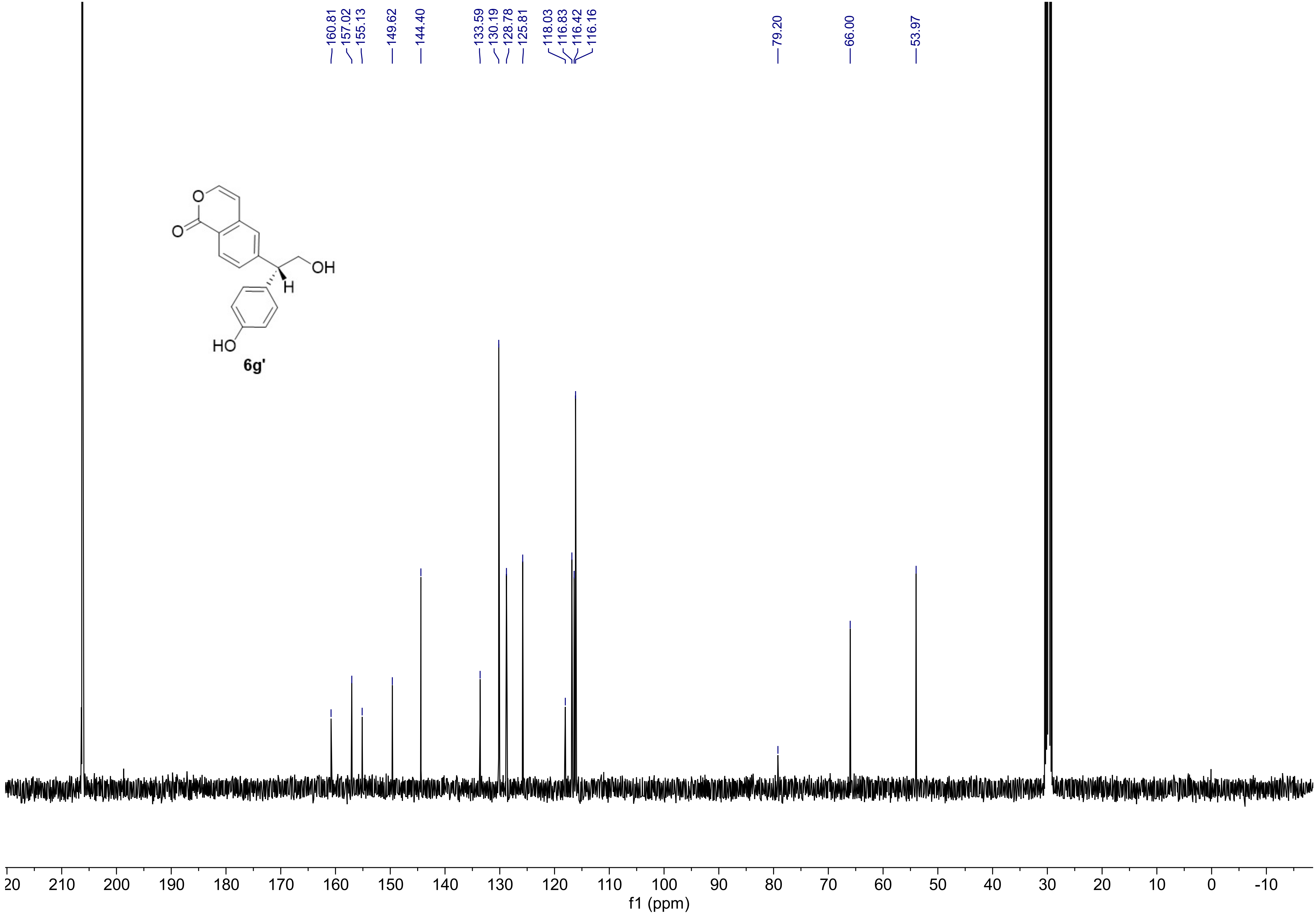
160.81
157.02
155.13
149.62
144.40
133.59
130.19
128.78
125.81
118.03
116.83
116.42
116.16
79.20
66.00
53.97
O
O
OH
H
HO
6g'
20
210
200
190
180
170
160
150
140
130
120
110
100
90
80
70
60
50
40
30
20
10
0
-10
f1 (ppm)

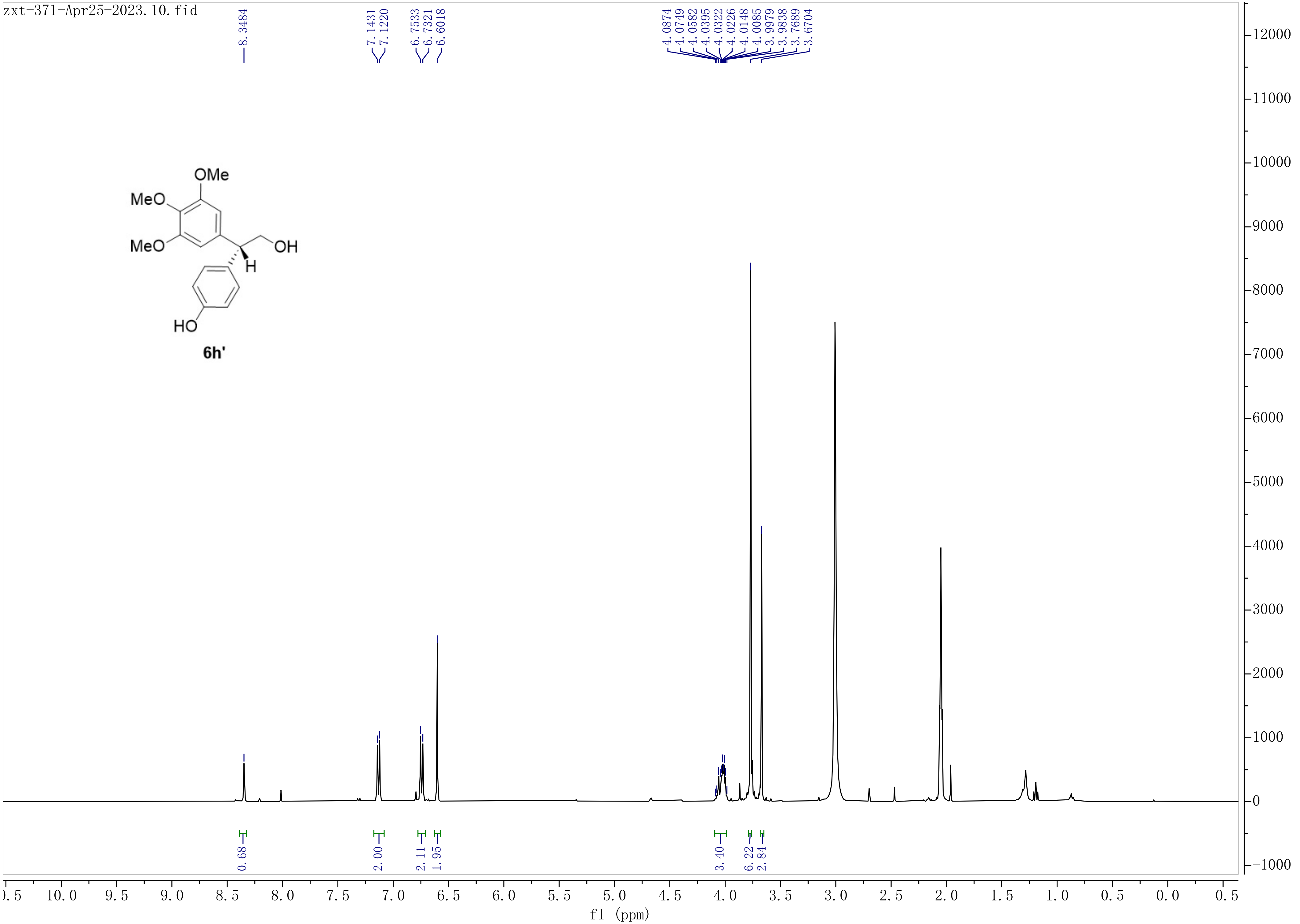
zxt-371-Apr25-2023.10.fid
8.3484
7.1431
7.1220
6.7533
6.7321
6.6018
4.0874
4.0749
4.0582
4.0395
4.0322
4.0226
4.0148
4.0085
3.9979
3.9838
3.7689
3.6704
OMe
MeO
MeO
OH
H
HO
6h'
0.68
2.00
2.11
1.95
3.40
6.22
2.84
f1 (ppm)

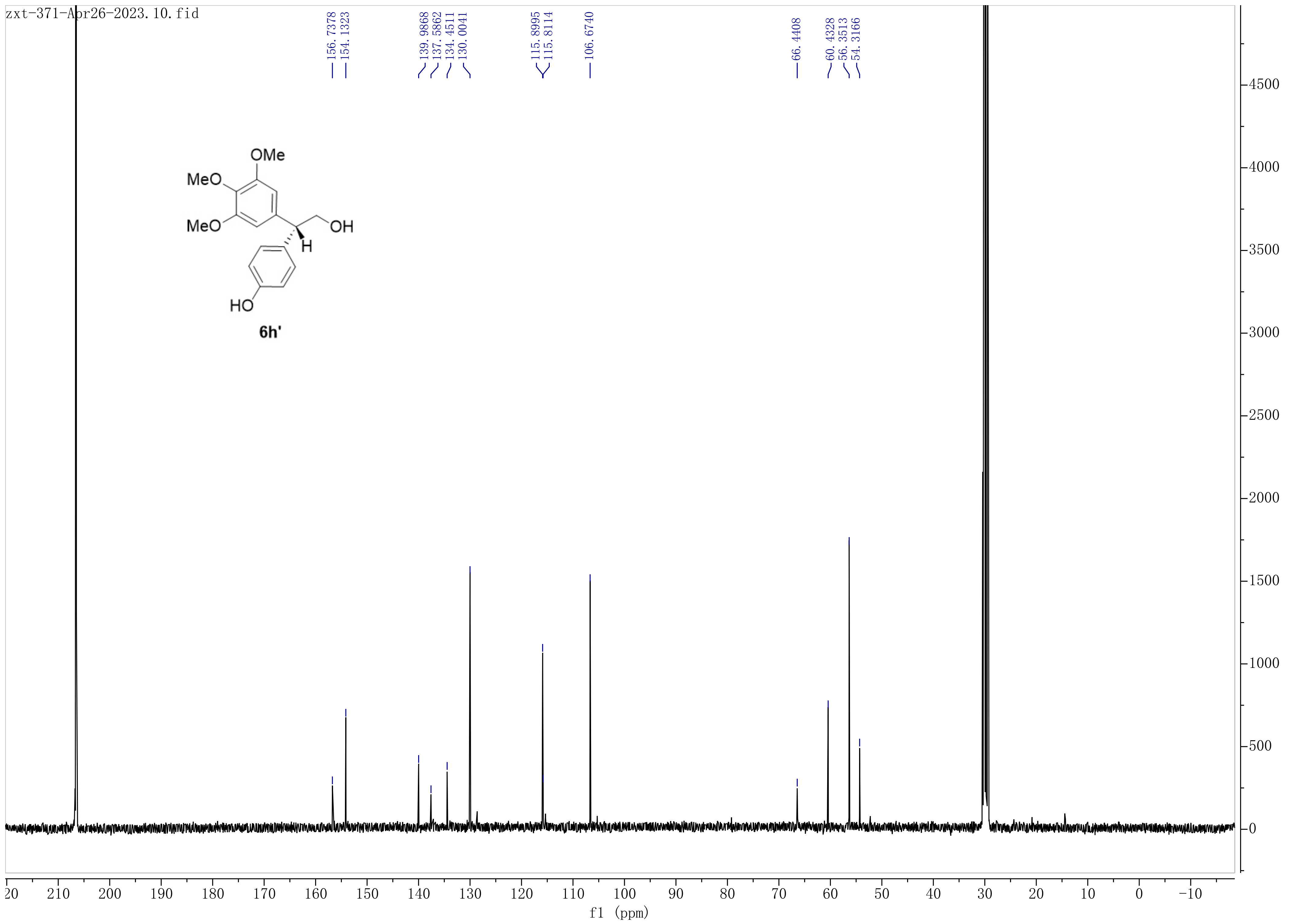
zxt-371-Apr26-2023.10.fid
156.7378
154.1323
139.9868
137.5862
134.4511
130.0041
115.8995
115.8114
106.6740
66.4408
60.4328
56.3513
54.3166
OMe
MeO
MeO
OH
H
HO
6h'
f1 (ppm)

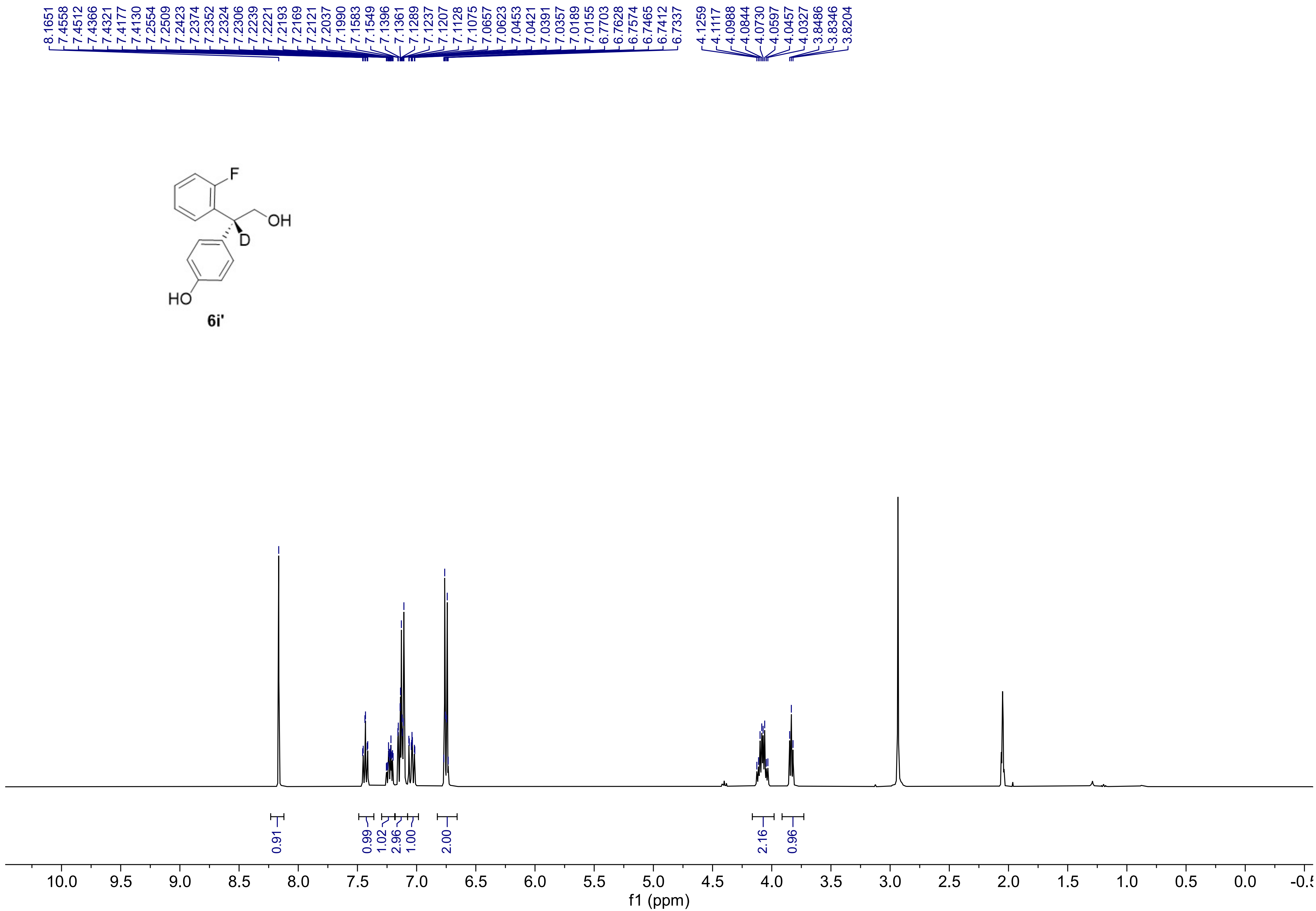
8.1651
7.4558
7.4512
7.4366
7.4321
7.4177
7.4130
7.2554
7.2509
7.2423
7.2374
7.2352
7.2324
7.2306
7.2239
7.2221
7.2193
7.2169
7.2121
7.2037
7.1990
7.1583
7.1549
7.1396
7.1361
7.1289
7.1237
7.1207
7.1128
7.1075
7.0657
7.0623
7.0453
7.0421
7.0391
7.0357
7.0189
7.0155
6.7703
6.7628
6.7574
6.7465
6.7412
6.7337
4.1259
4.1117
4.0988
4.0844
4.0730
4.0597
4.0457
4.0327
3.8486
3.8346
3.8204
F
OH
D
HO
0.91
0.99
1.02
2.96
1.00
2.00
2.16
0.96
f1 (ppm)
6i'

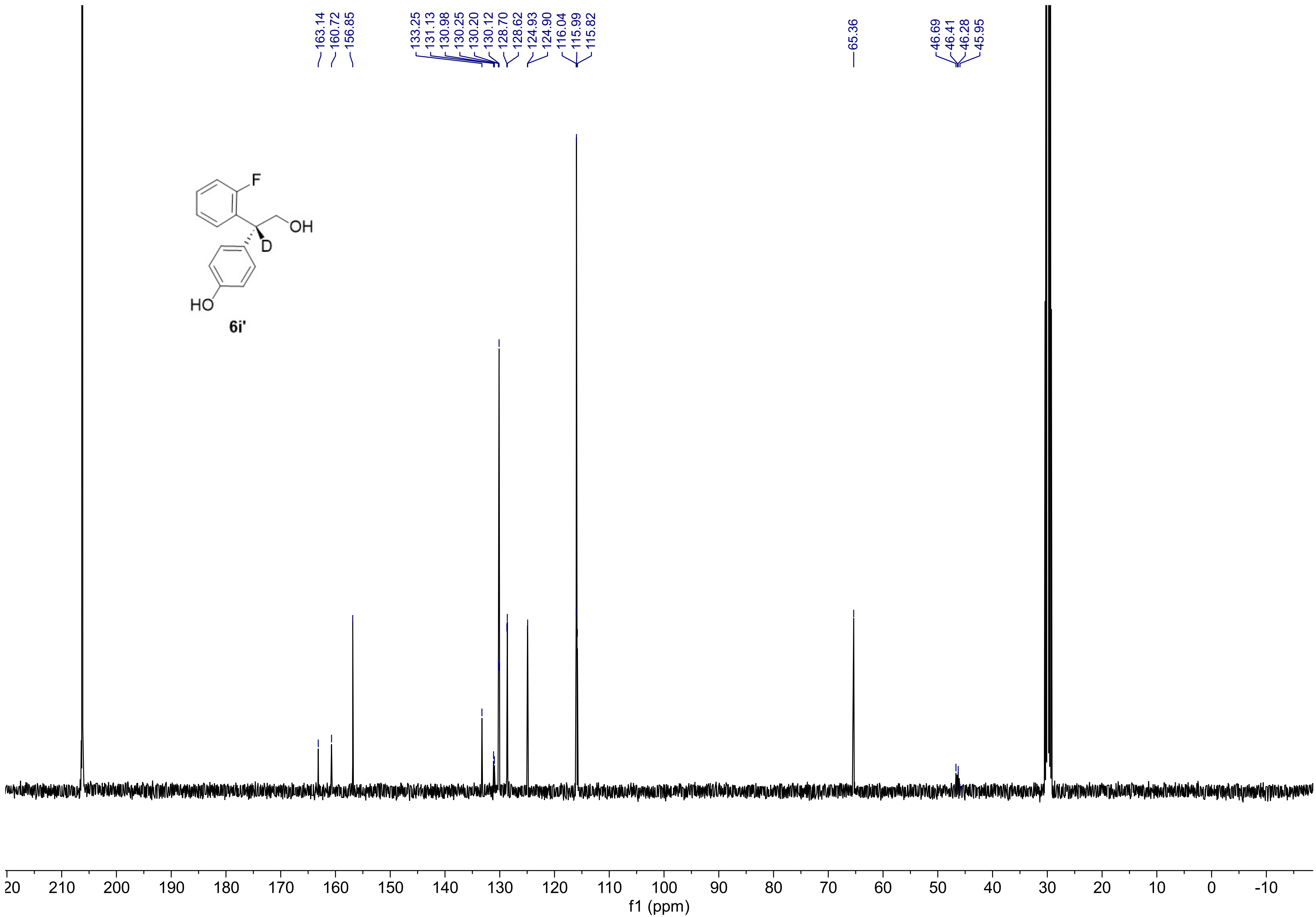
163.14
160.72
156.85
133.25
131.13
130.98
130.25
130.20
130.12
128.70
128.62
124.93
124.90
116.04
115.99
115.82
65.36
46.69
46.41
46.28
45.95
F
OH
D
HO
6i'
20
210
200
190
180
170
160
150
140
130
120
110
100
90
80
70
60
50
40
30
20
10
0
-10
f1 (ppm)

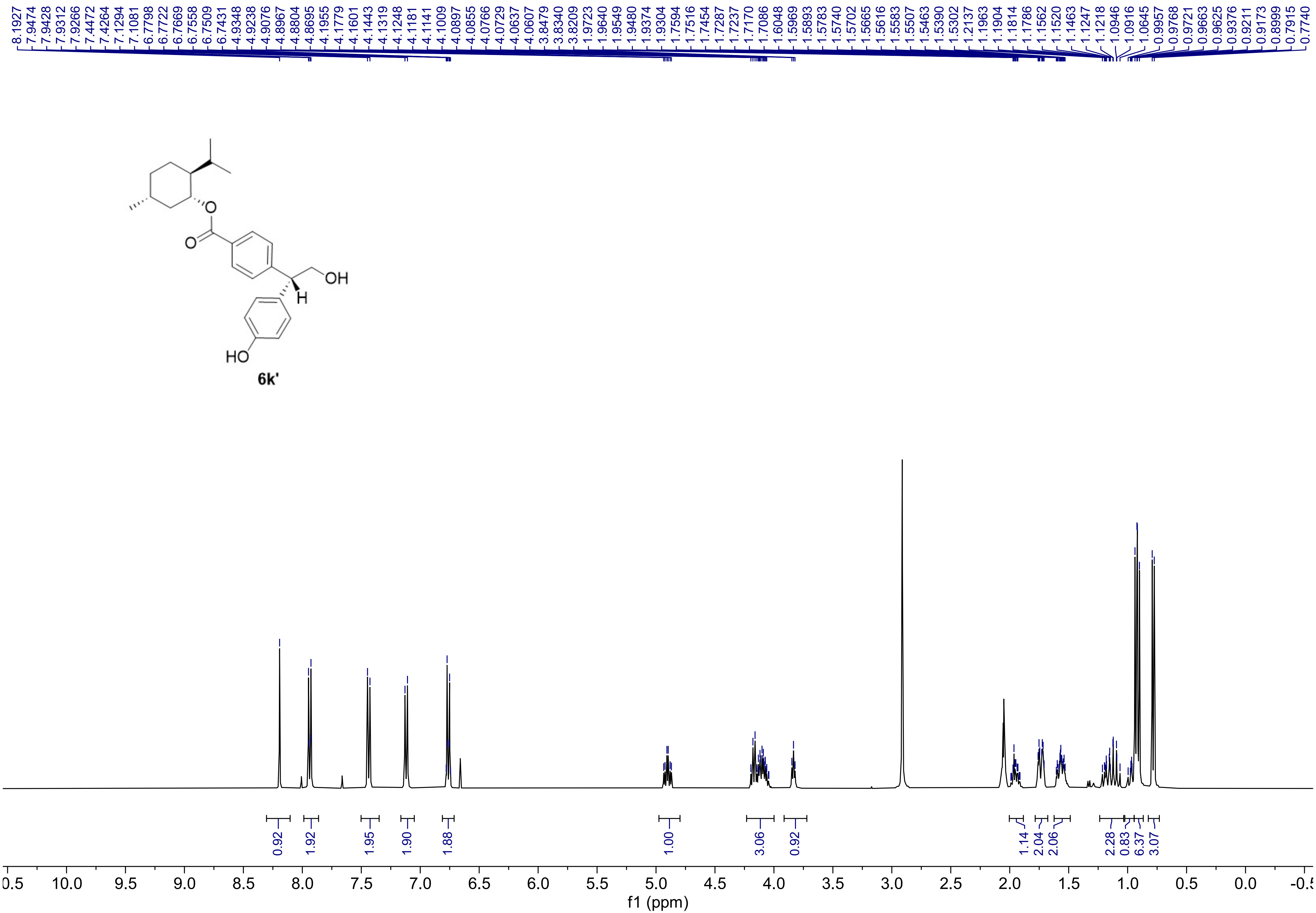
8.1927
7.9474
7.9428
7.9312
7.9266
7.4472
7.4264
7.1294
7.1081
6.7798
6.7722
6.7669
6.7558
6.7509
6.7431
4.9348
4.9238
4.9076
4.8967
4.8804
4.8695
4.1955
4.1779
4.1601
4.1443
4.1319
4.1248
4.1181
4.1141
4.1009
4.0897
4.0855
4.0766
4.0729
4.0637
4.0607
3.8479
3.8340
3.8209
1.9723
1.9640
1.9549
1.9480
1.9374
1.9304
1.7594
1.7516
1.7454
1.7287
1.7237
1.7170
1.7086
1.6048
1.5969
1.5893
1.5783
1.5740
1.5702
1.5665
1.5616
1.5583
1.5507
1.5463
1.5390
1.5302
1.2137
1.1963
1.1904
1.1814
1.1786
1.1562
1.1520
1.1463
1.1247
1.1218
1.0946
1.0916
1.0645
0.9957
0.9768
0.9721
0.9663
0.9625
0.9376
0.9211
0.9173
0.8999
0.7915
0.7740
O
O
OH
H
HO
6k'
0.92
1.92
1.95
1.90
1.88
1.00
3.06
0.92
1.14
2.04
2.06
2.28
0.83
6.37
3.07
f1 (ppm)

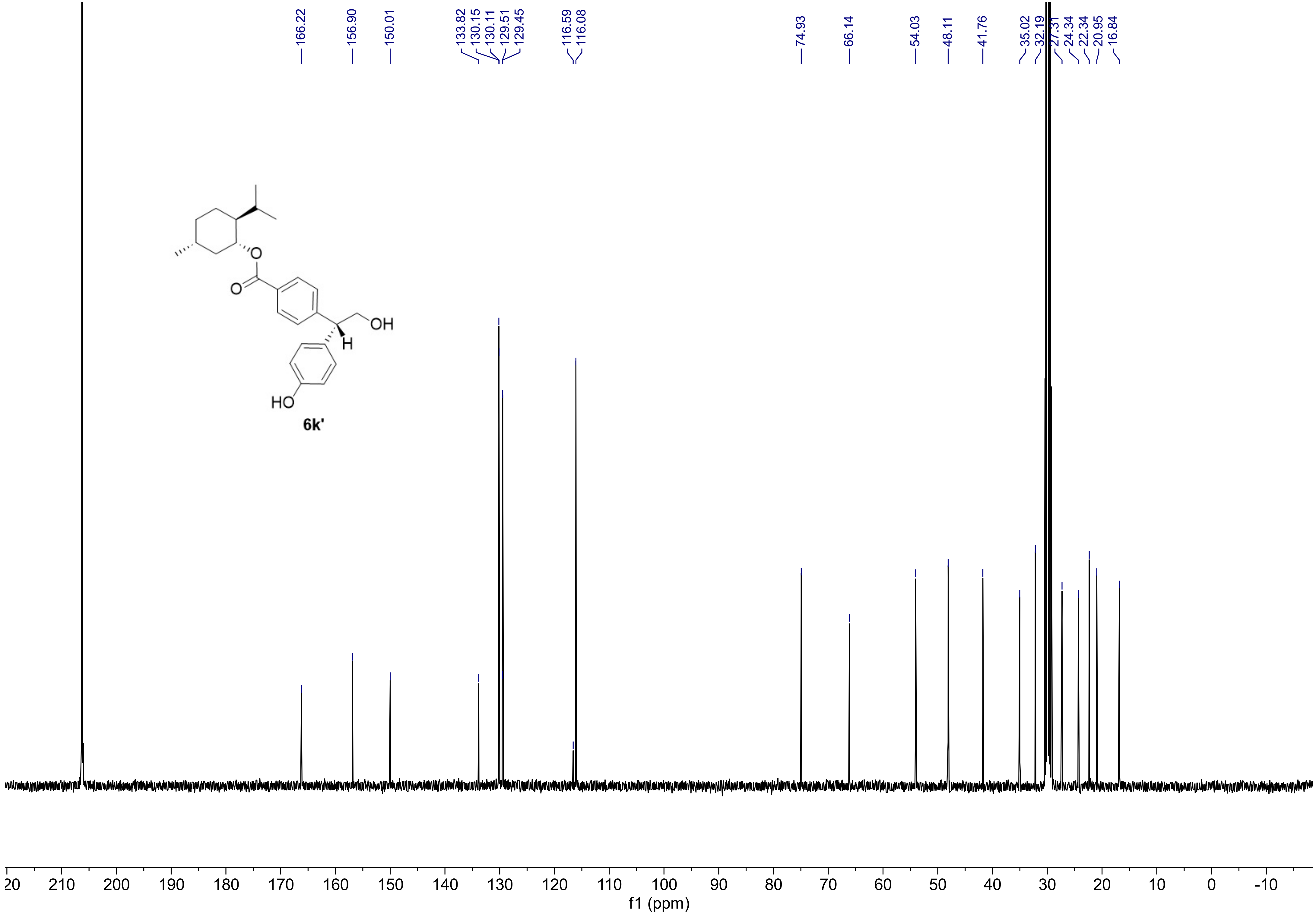
166.22
156.90
150.01
133.82
130.15
130.11
129.51
129.45
116.59
116.08
74.93
66.14
54.03
48.11
41.76
35.02
32.19
27.31
24.34
22.34
20.95
16.84
O
O
OH
H
HO
6k'
20 210 200 190 180 170 160 150 140 130 120 110 100 90 80 70 60 50 40 30 20 10 0 -10
f1 (ppm)

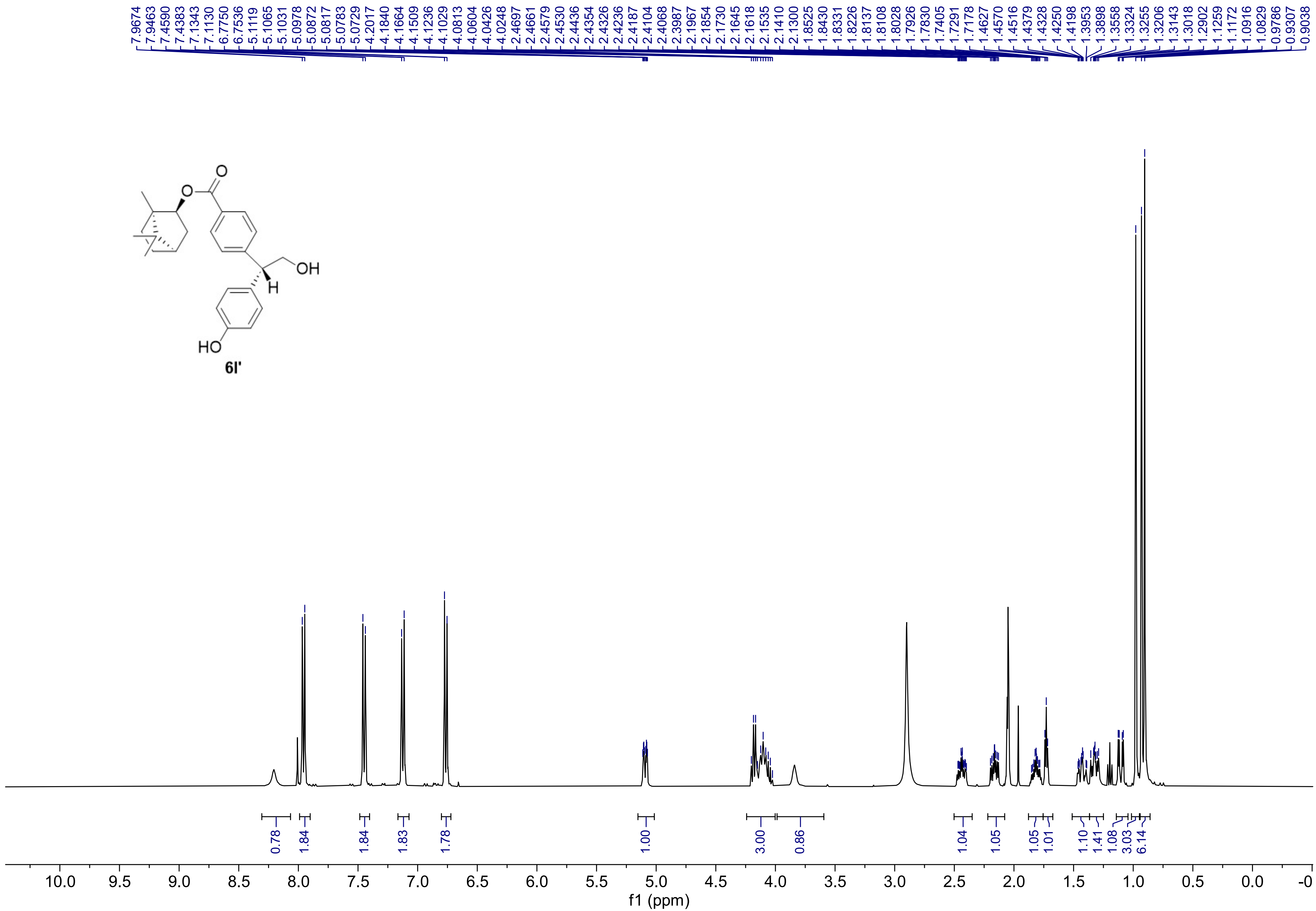
7.9674 7.9463 7.4590 7.4383 7.1343 7.1130 6.7750 6.7536 5.1119 5.1065 5.1031 5.0978 5.0872 5.0817 5.0783 5.0729 4.2017 4.1840 4.1664 4.1509 4.1236 4.1029 4.0813 4.0604 4.0426 4.0248 2.4697 2.4661 2.4579 2.4530 2.4436 2.4354 2.4326 2.4236 2.4187 2.4104 2.4068 2.3987 2.1967 2.1854 2.1730 2.1645 2.1618 2.1535 2.1410 2.1300 1.8525 1.8430 1.8331 1.8226 1.8137 1.8108 1.8028 1.7926 1.7830 1.7405 1.7291 1.7178 1.4627 1.4570 1.4516 1.4379 1.4328 1.4250 1.4198 1.3953 1.3898 1.3558 1.3324 1.3255 1.3206 1.3143 1.3018 1.2902 1.1259 1.1172 1.0916 1.0829 0.9786 0.9307 0.9038
O
O
OH
H
HO
6l'
0.78 1.84 1.84 1.83 1.78 1.00 3.00 0.86 1.04 1.05 1.05 1.01 1.10 1.41 1.08 3.03 6.14
10.0 9.5 9.0 8.5 8.0 7.5 7.0 6.5 6.0 5.5 5.0 4.5 4.0 3.5 3.0 2.5 2.0 1.5 1.0 0.5 0.0 -0
f1 (ppm)

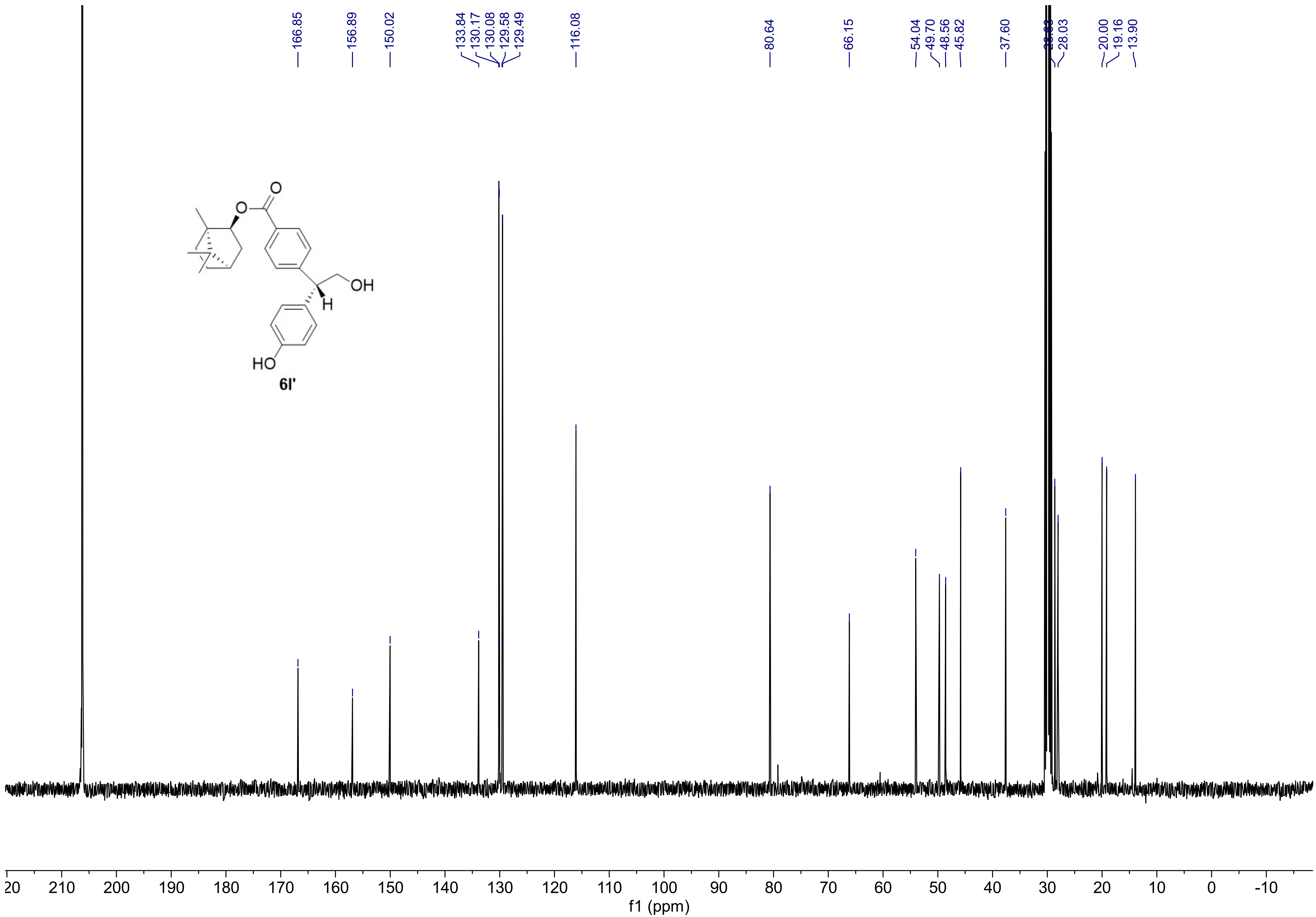

166.85
156.89
150.02
133.84
130.17
130.08
129.58
129.49
116.08
80.64
66.15
54.04
49.70
48.56
45.82
37.60
28.03
20.00
19.16
13.90
O
OH
H
HO
6l'
20
210
200
190
180
170
160
150
140
130
120
110
100
90
80
70
60
50
40
30
20
10
0
-10
f1 (ppm)

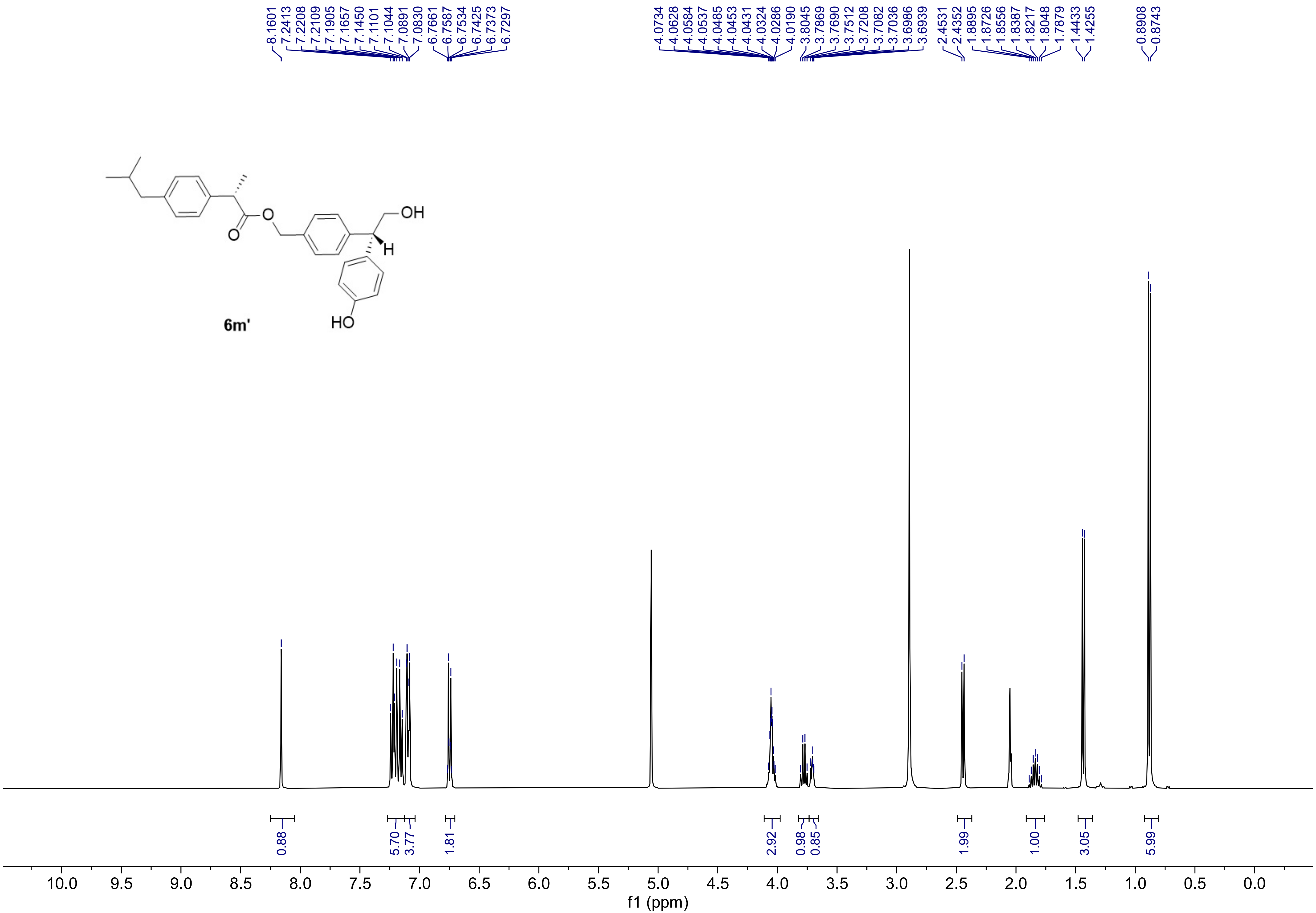
8.1601
7.2413
7.2208
7.2109
7.1905
7.1657
7.1450
7.1101
7.1044
7.0891
7.0830
6.7661
6.7587
6.7534
6.7425
6.7373
6.7297
4.0734
4.0628
4.0584
4.0537
4.0485
4.0453
4.0431
4.0324
4.0286
4.0190
3.8045
3.7869
3.7690
3.7512
3.7208
3.7082
3.7036
3.6986
3.6939
2.4531
2.4352
1.8895
1.8726
1.8556
1.8387
1.8217
1.8048
1.7879
1.4433
1.4255
0.8908
0.8743
OH
O
O
H
HO
6m'
0.88
5.70
3.77
1.81
2.92
0.98
0.85
1.99
1.00
3.05
5.99
10.0
9.5
9.0
8.5
8.0
7.5
7.0
6.5
6.0
5.5
5.0
4.5
4.0
3.5
3.0
2.5
2.0
1.5
1.0
0.5
0.0
f1 (ppm)

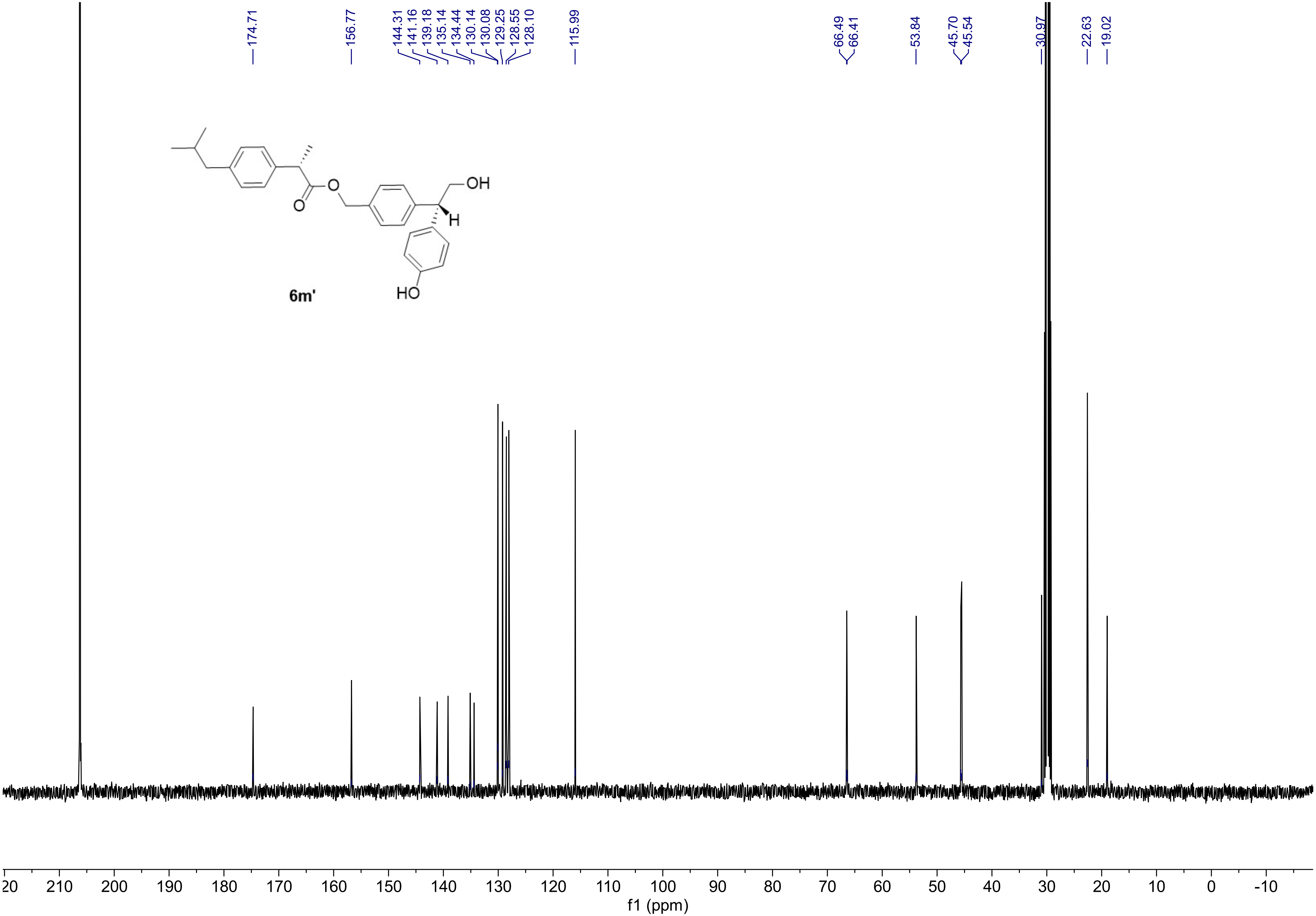
174.71
156.77
144.31
141.16
139.18
135.14
134.44
130.14
130.08
129.25
128.55
128.10
115.99
66.49
66.41
53.84
45.70
45.54
30.97
22.63
19.02
OH
O
O
H
HO
6m'
20 210 200 190 180 170 160 150 140 130 120 110 100 90 80 70 60 50 40 30 20 10 0 -10
f1 (ppm)

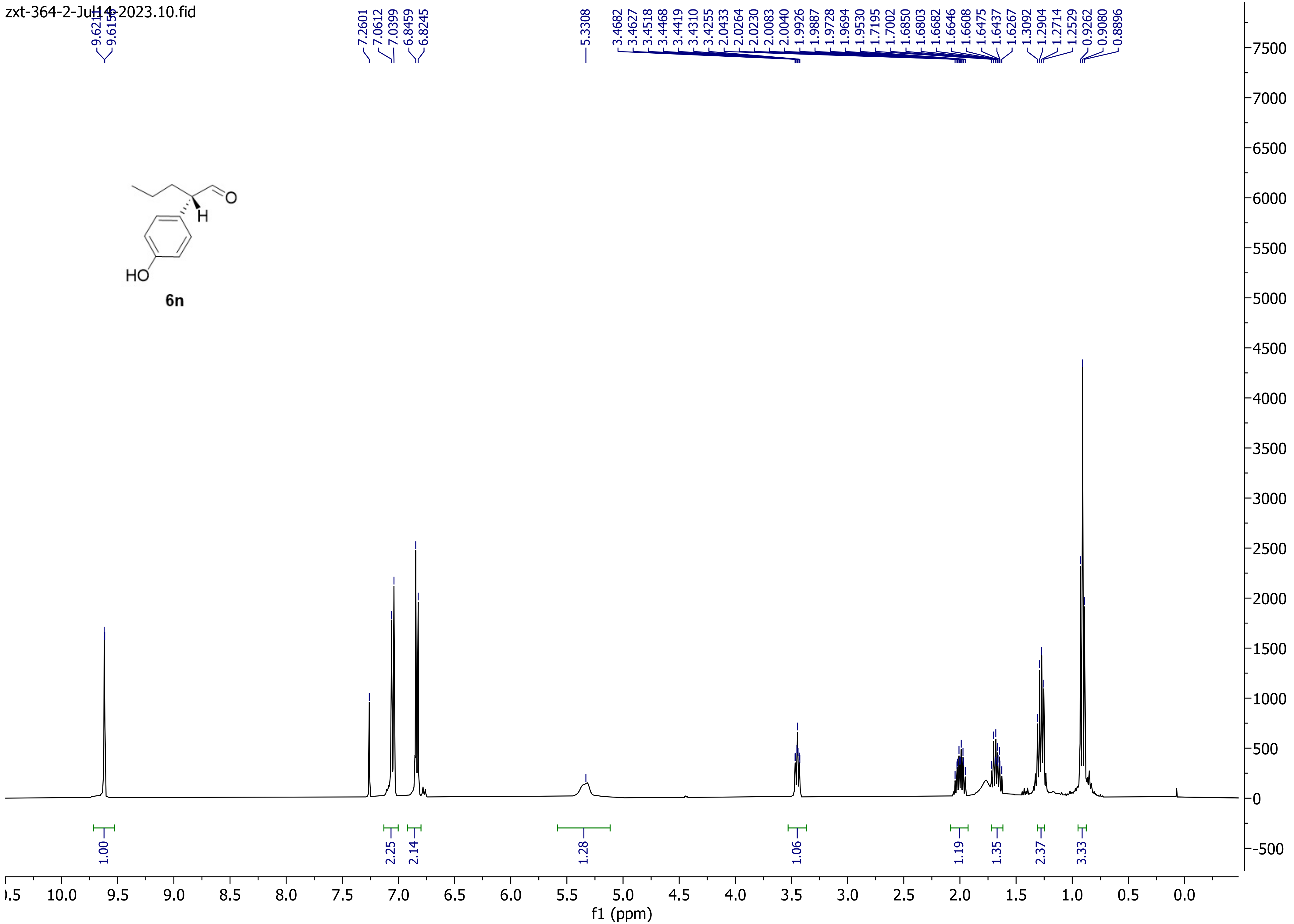
zxt-364-2-Jul14-2023.10.fid
9.6211
9.6154
7.2601
7.0612
7.0399
6.8459
6.8245
5.3308
3.4682
3.4627
3.4518
3.4468
3.4419
3.4310
3.4255
2.0433
2.0264
2.0230
2.0083
2.0040
1.9926
1.9887
1.9728
1.9694
1.9530
1.7195
1.7002
1.6850
1.6803
1.6682
1.6646
1.6608
1.6475
1.6437
1.6267
1.3092
1.2904
1.2714
1.2529
0.9262
0.9080
0.8896
O
H
HO
6n
1.00
2.25
2.14
1.28
1.06
1.19
1.35
2.37
3.33
f1 (ppm)

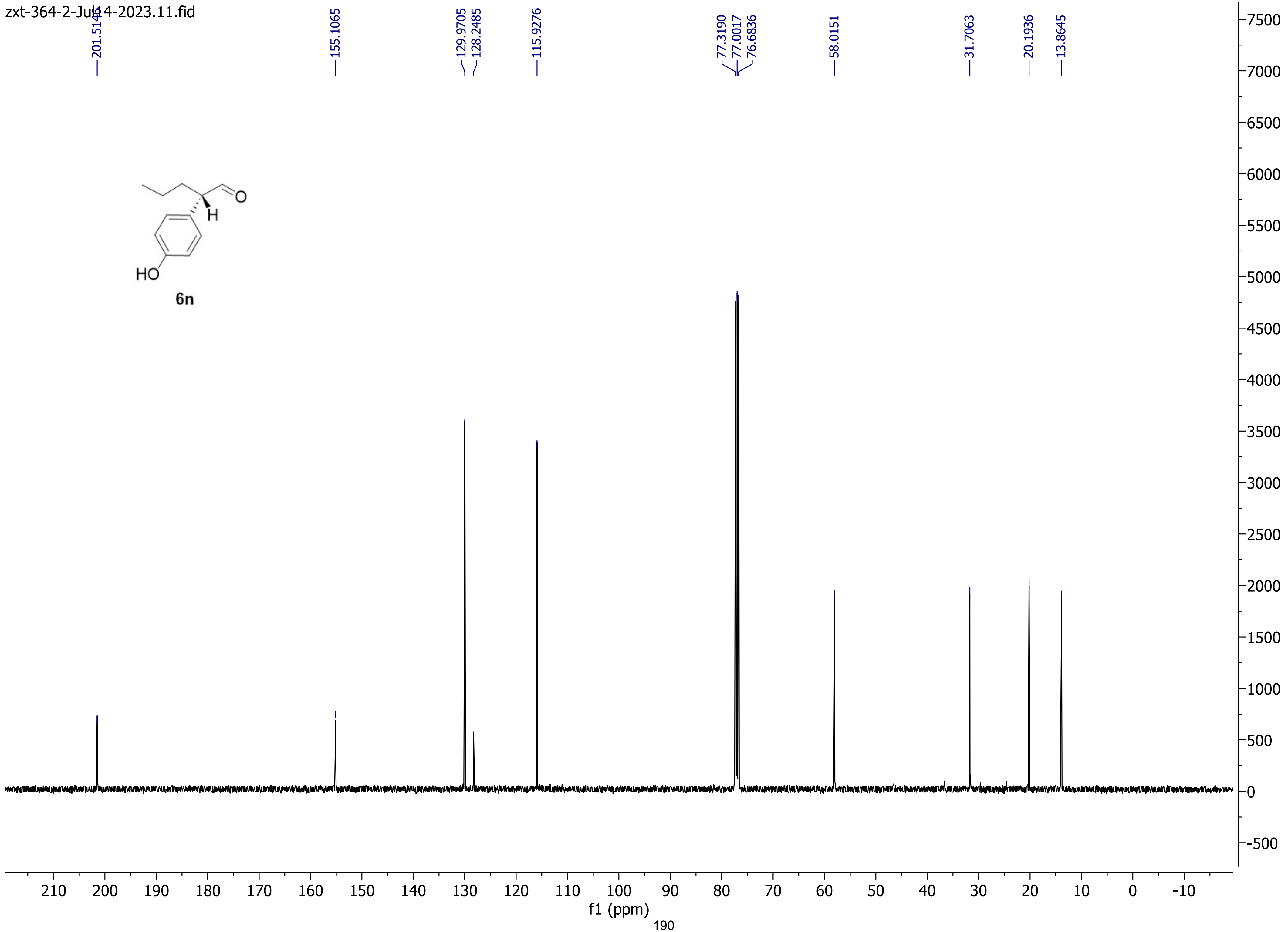
zxt-364-2-Jul14-2023.11.fid
201.514
155.1065
129.9705
128.2485
115.9276
77.3190
77.0017
76.6836
58.0151
31.7063
20.1936
13.8645
O
H
HO
6n
f1 (ppm)
210 200 190 180 170 160 150 140 130 120 110 100 90 80 70 60 50 40 30 20 10 0 -10
7500 7000 6500 6000 5500 5000 4500 4000 3500 3000 2500 2000 1500 1000 500 0 -500

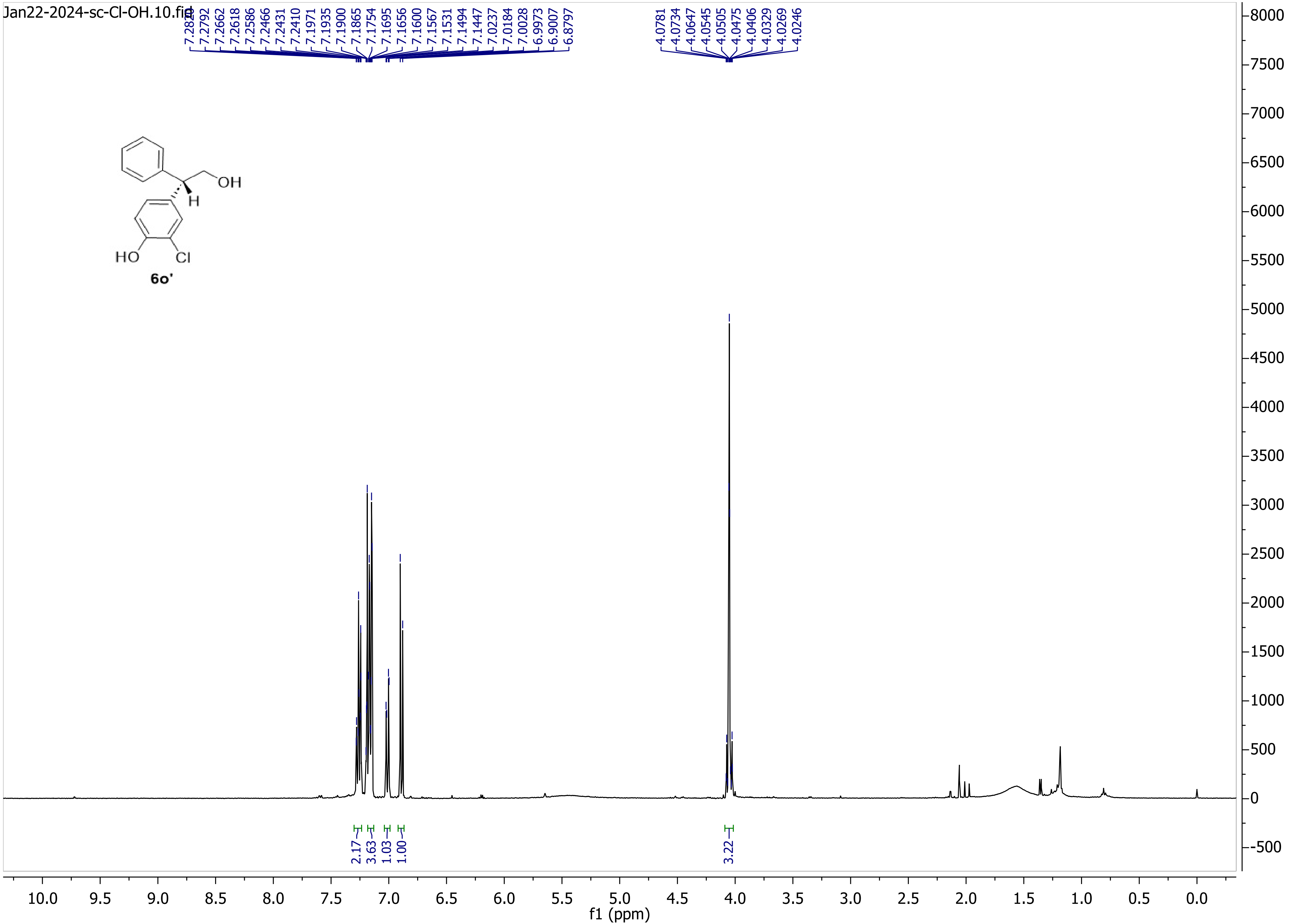
Jan22-2024-sc-Cl-OH.10.fid
7.2820 7.2792 7.2662 7.2618 7.2586 7.2466 7.2431 7.2410 7.1971 7.1935 7.1900 7.1865 7.1754 7.1695 7.1656 7.1600 7.1567 7.1531 7.1494 7.1447 7.0237 7.0184 7.0028 6.9973 6.9007 6.8797
4.0781 4.0734 4.0647 4.0545 4.0505 4.0475 4.0406 4.0329 4.0269 4.0246
OH
H
HO
Cl
6o'
2.17
3.63
1.03
1.00
3.22
f1 (ppm)

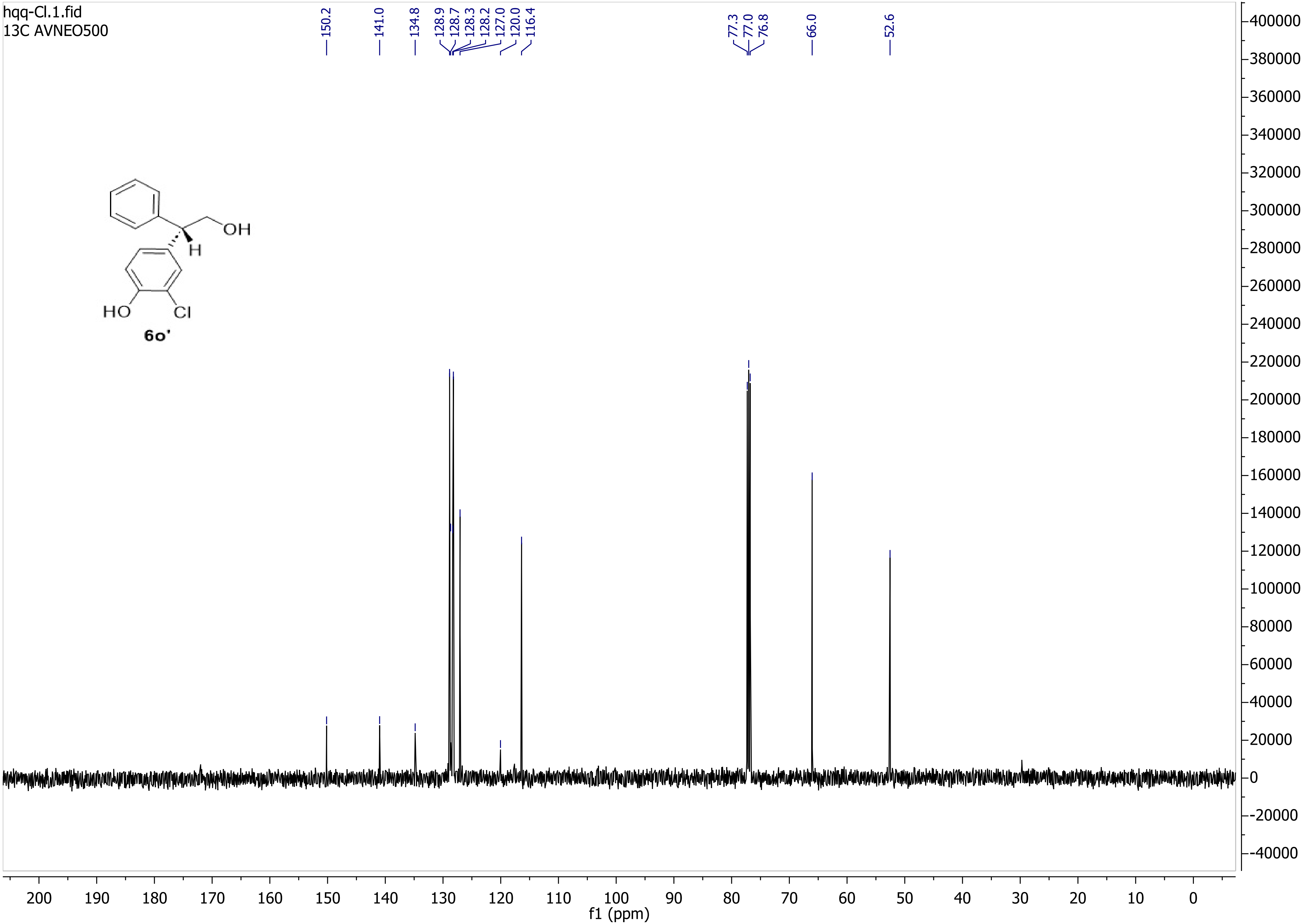
hqq-Cl.1.fid
13C AVNEO500
150.2
141.0
134.8
128.9
128.7
128.3
128.2
127.0
120.0
116.4
77.3
77.0
76.8
66.0
52.6
OH
H
HO
Cl
6o'
f1 (ppm)

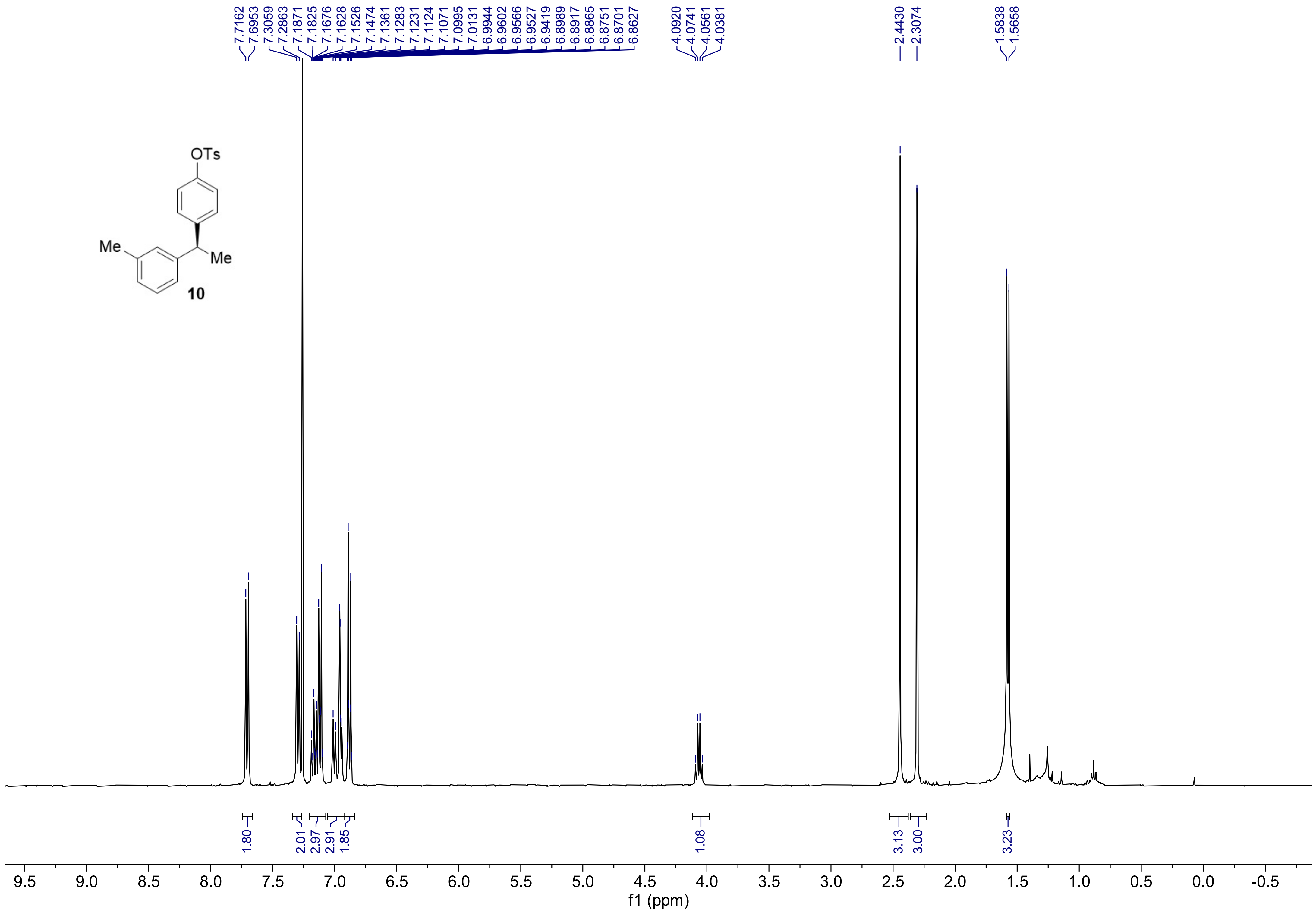

7.7162
7.6953
7.3059
7.2863
7.1871
7.1825
7.1676
7.1628
7.1526
7.1474
7.1361
7.1283
7.1231
7.1124
7.1071
7.0995
7.0131
6.9944
6.9602
6.9566
6.9527
6.9419
6.8989
6.8917
6.8865
6.8751
6.8701
6.8627
4.0920
4.0741
4.0561
4.0381
2.4430
2.3074
1.5838
1.5658
OTs
Me
Me
10
1.80
2.01
2.97
2.91
1.85
1.08
3.13
3.00
3.23
9.5
9.0
8.5
8.0
7.5
7.0
6.5
6.0
5.5
5.0
4.5
4.0
3.5
3.0
2.5
2.0
1.5
1.0
0.5
0.0
-0.5
f1 (ppm)

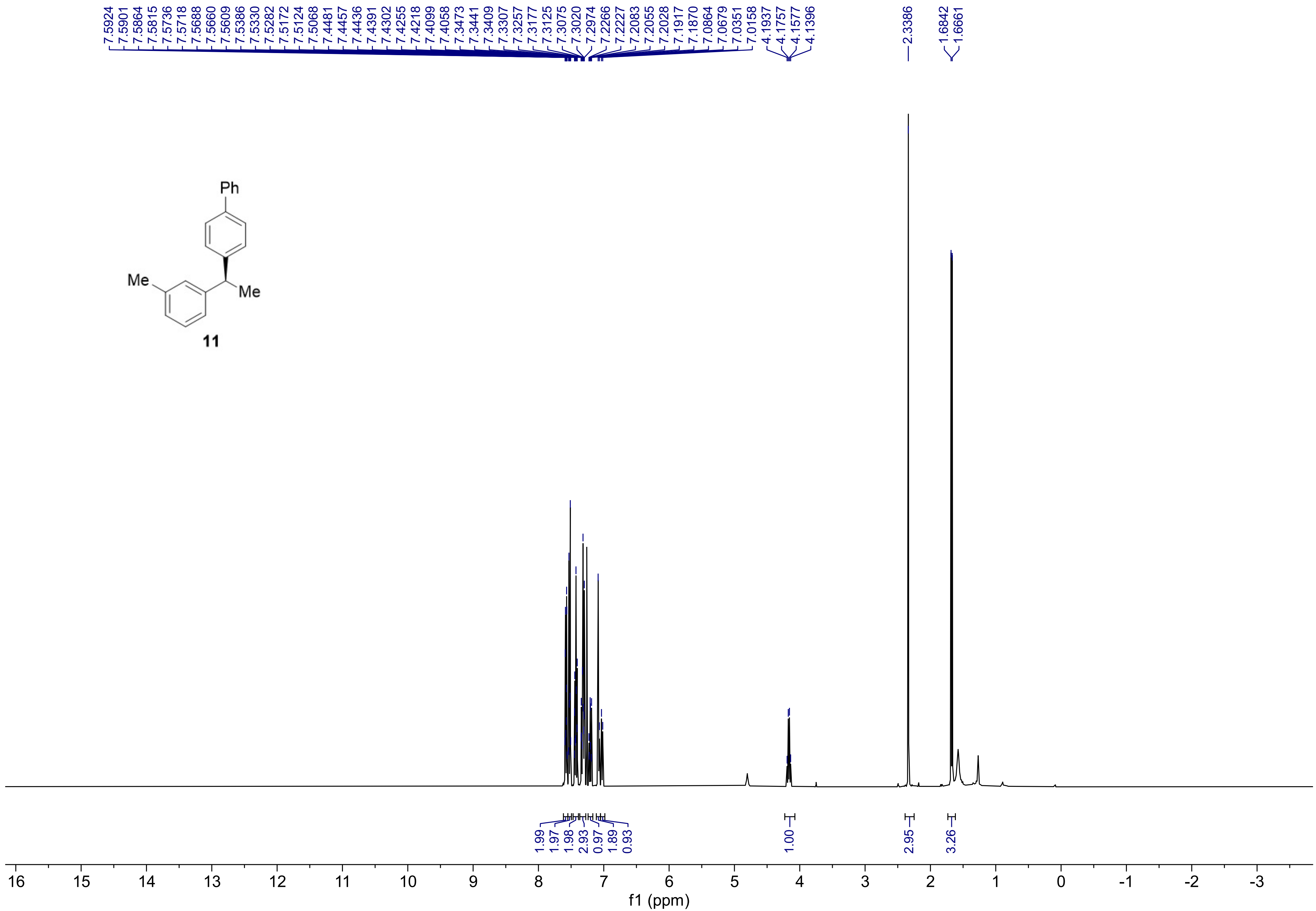
7.5924
7.5901
7.5864
7.5815
7.5736
7.5718
7.5688
7.5660
7.5609
7.5386
7.5330
7.5282
7.5172
7.5124
7.5068
7.4481
7.4457
7.4436
7.4391
7.4302
7.4255
7.4218
7.4099
7.4058
7.3473
7.3441
7.3409
7.3307
7.3257
7.3177
7.3125
7.3075
7.3020
7.2974
7.2266
7.2227
7.2083
7.2055
7.2028
7.1917
7.1870
7.0864
7.0679
7.0351
7.0158
4.1937
4.1757
4.1577
4.1396
2.3386
1.6842
1.6661
Ph
Me
Me
11
1.99
1.97
1.98
2.93
0.97
1.89
0.93
1.00
2.95
3.26
16
15
14
13
12
11
10
9
8
7
6
5
4
3
2
1
0
-1
-2
-3
f1 (ppm)

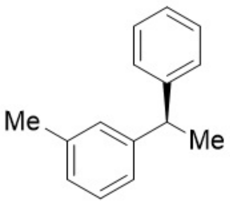
Me
Me

12

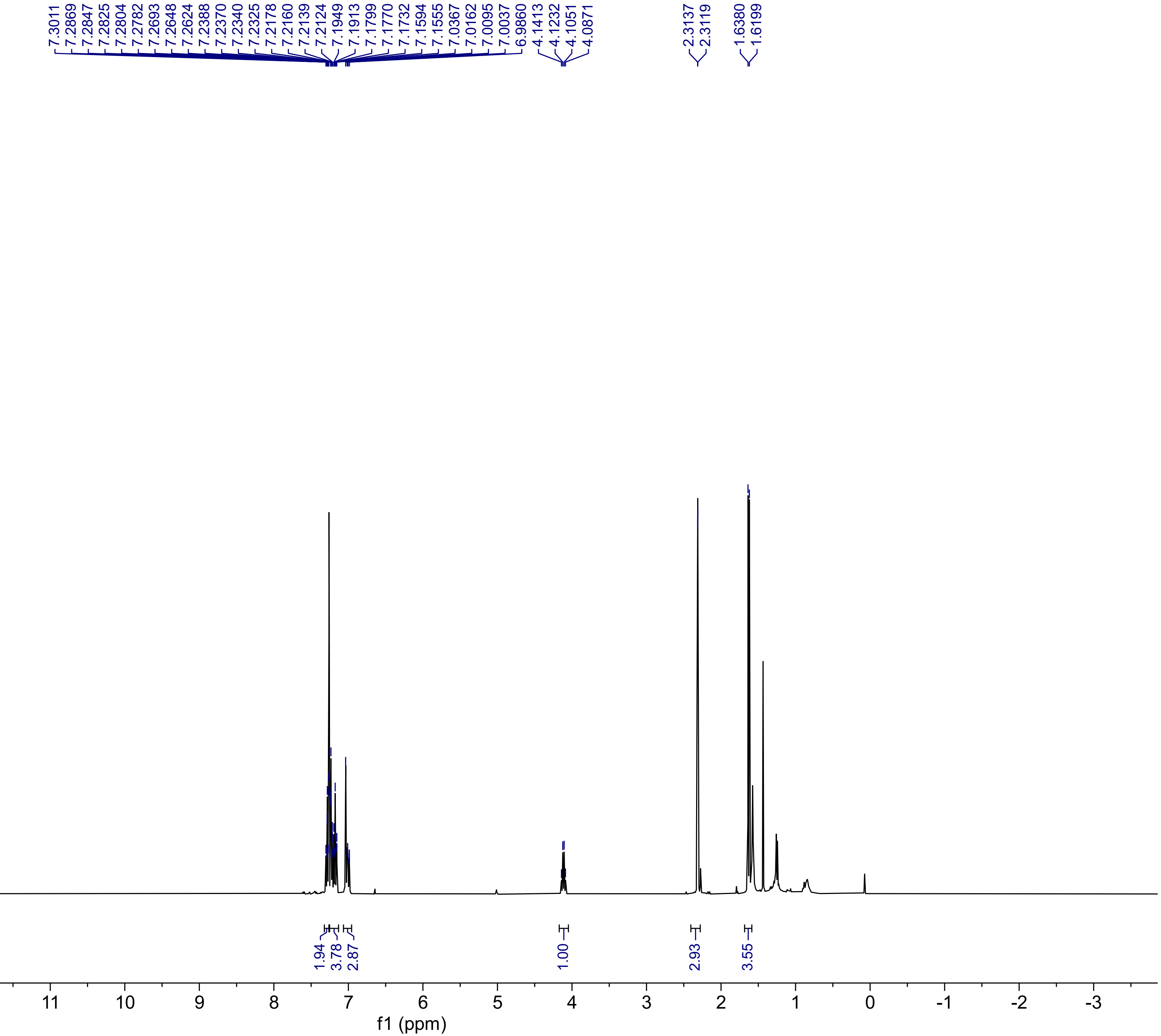
7.3011
7.2869
7.2847
7.2825
7.2804
7.2782
7.2693
7.2648
7.2624
7.2388
7.2370
7.2340
7.2325
7.2178
7.2160
7.2139
7.2124
7.1949
7.1913
7.1799
7.1770
7.1732
7.1594
7.1555
7.0367
7.0162
7.0095
7.0037
6.9860
4.1413
4.1232
4.1051
4.0871
2.3137
2.3119
1.6380
1.6199
1.94
3.78
2.87
1.00
2.93
3.55
16
15
14
13
12
11
10
9
8
7
6
5
4
3
2
1
0
-1
-2
-3
f1 (ppm)

zxt-377-Jun23-2023.10.fid

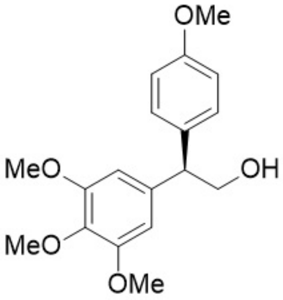

14

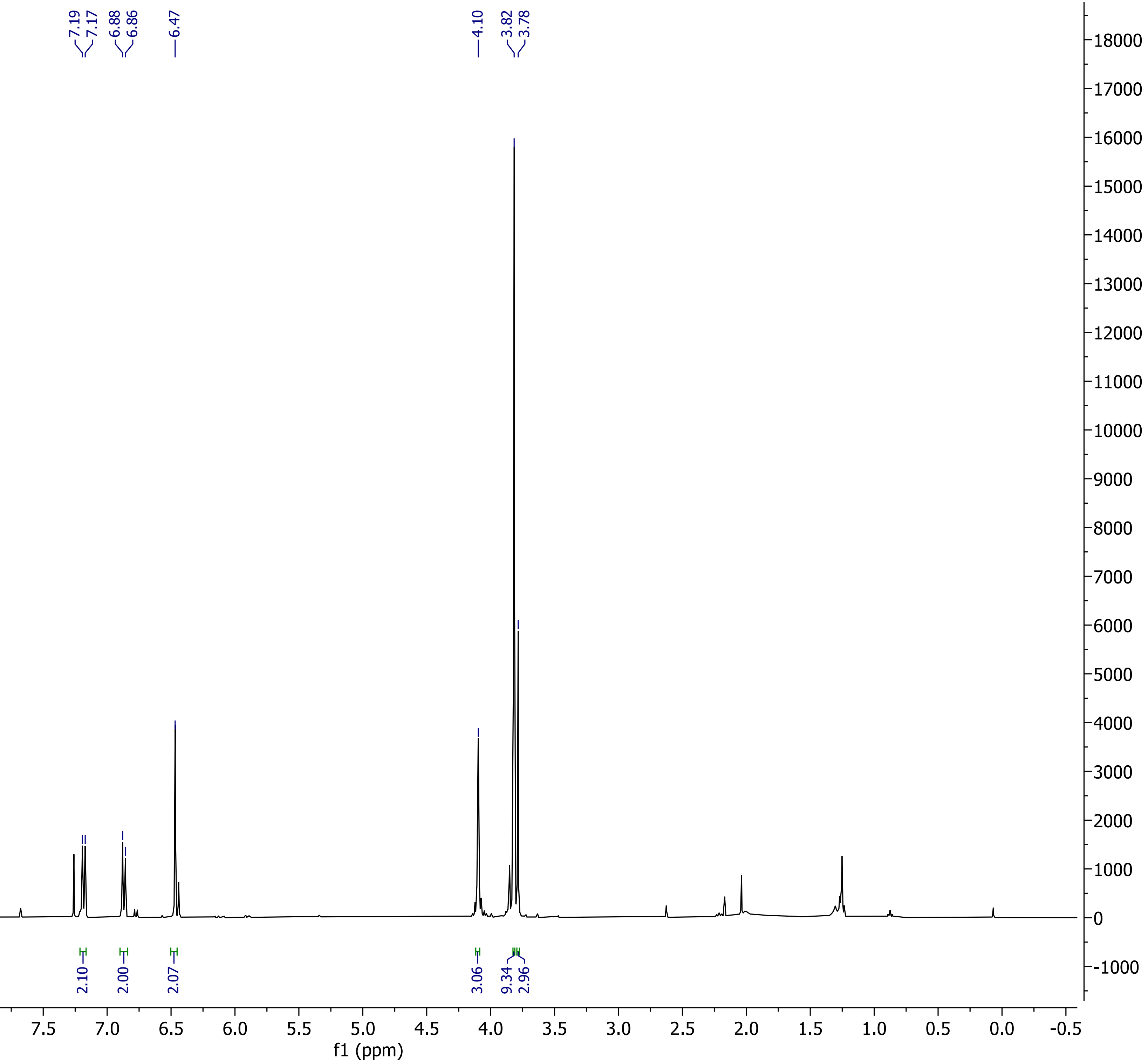

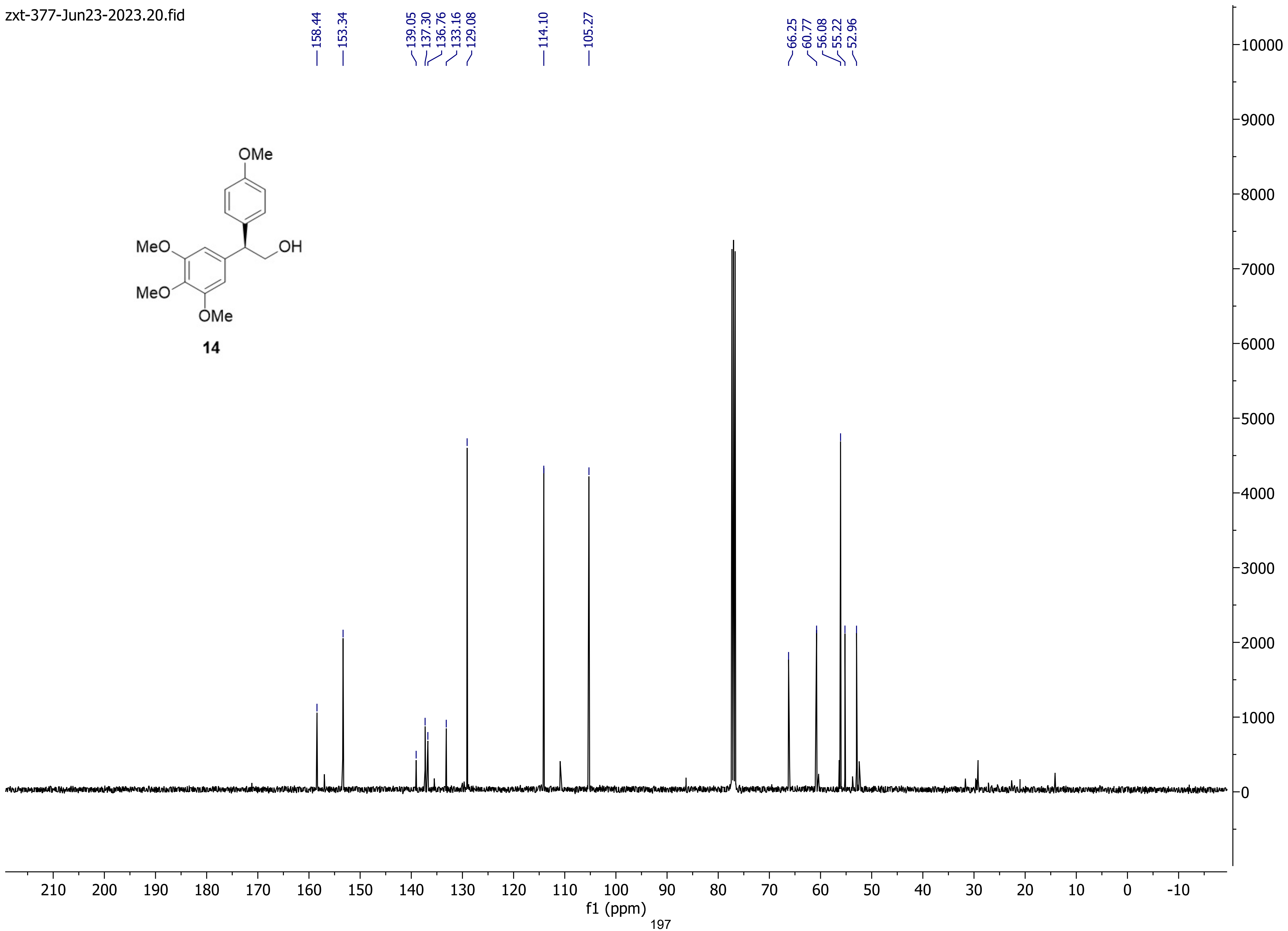
zxt-377-Jun23-2023.20.fid
158.44
153.34
139.05
137.30
136.76
133.16
129.08
114.10
105.27
66.25
60.77
56.08
55.22
52.96
OMe
MeO
OH
MeO
OMe
14
f1 (ppm)

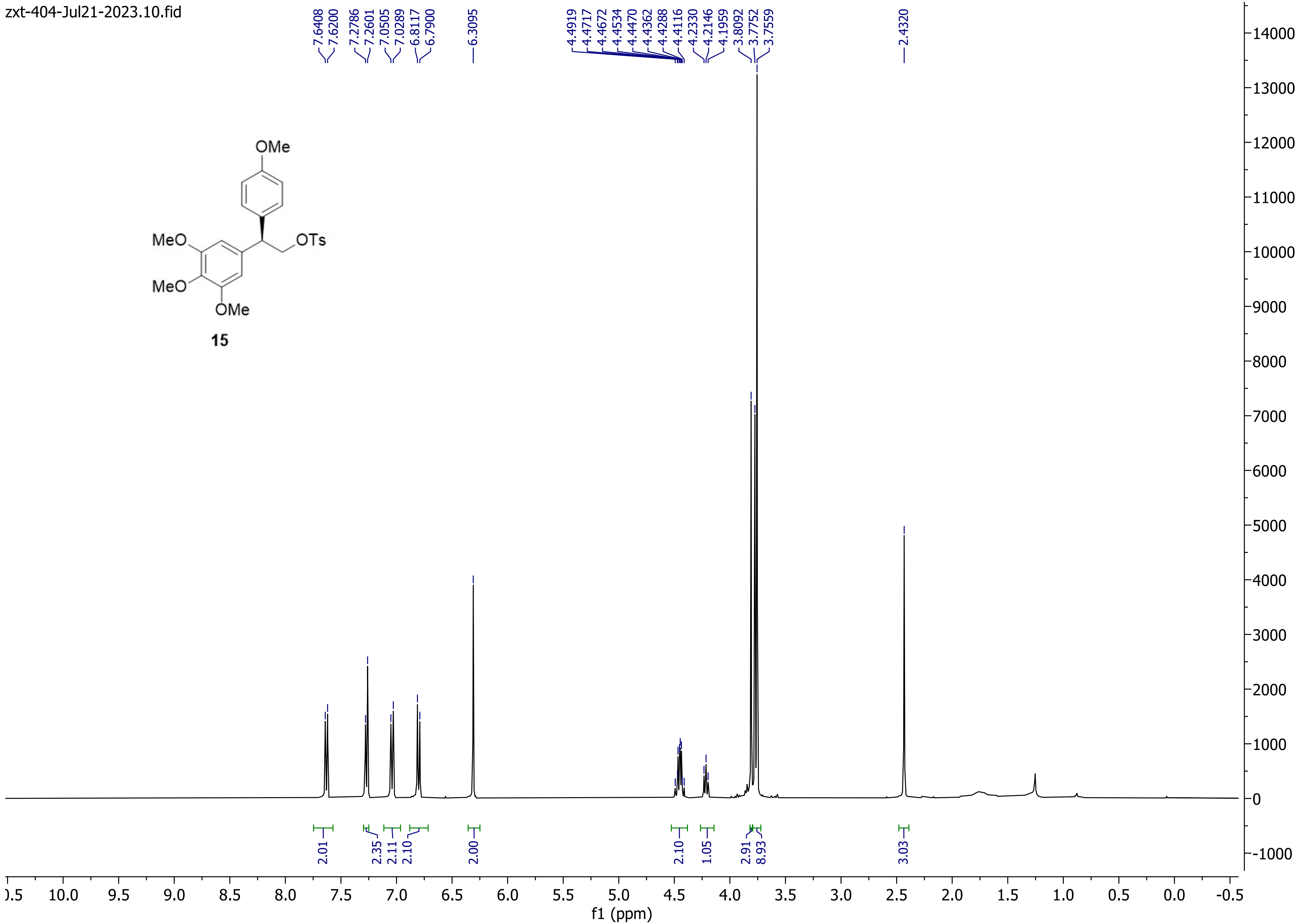
zxt-404-Jul21-2023.10.fid
OMe
MeO
OTs
MeO
OMe
15
7.6408
7.6200
7.2786
7.2601
7.0505
7.0289
6.8117
6.7900
6.3095
4.4919
4.4717
4.4672
4.4534
4.4470
4.4362
4.4288
4.4116
4.2330
4.2146
4.1959
3.8092
3.7752
3.7559
2.4320
2.01
2.35
2.11
2.10
2.00
2.10
1.05
2.91
8.93
3.03
f1 (ppm)

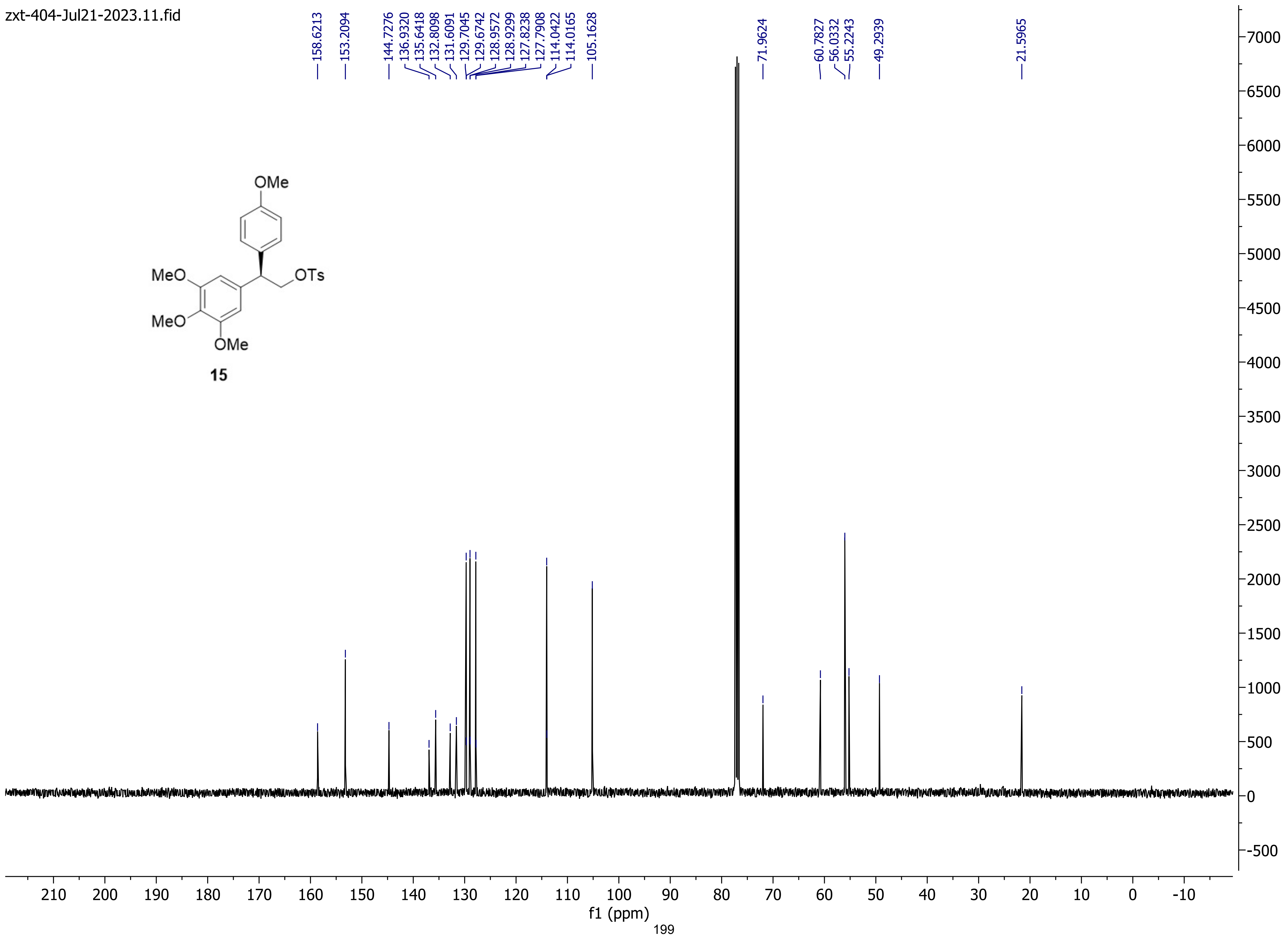
zxt-404-Jul21-2023.11.fid
158.6213
153.2094
144.7276
136.9320
135.6418
132.8098
131.6091
129.7045
129.6742
128.9572
128.9299
127.8238
127.7908
114.0422
114.0165
105.1628
71.9624
60.7827
56.0332
55.2243
49.2939
21.5965
OMe
MeO
OTs
MeO
OMe
15
f1 (ppm)

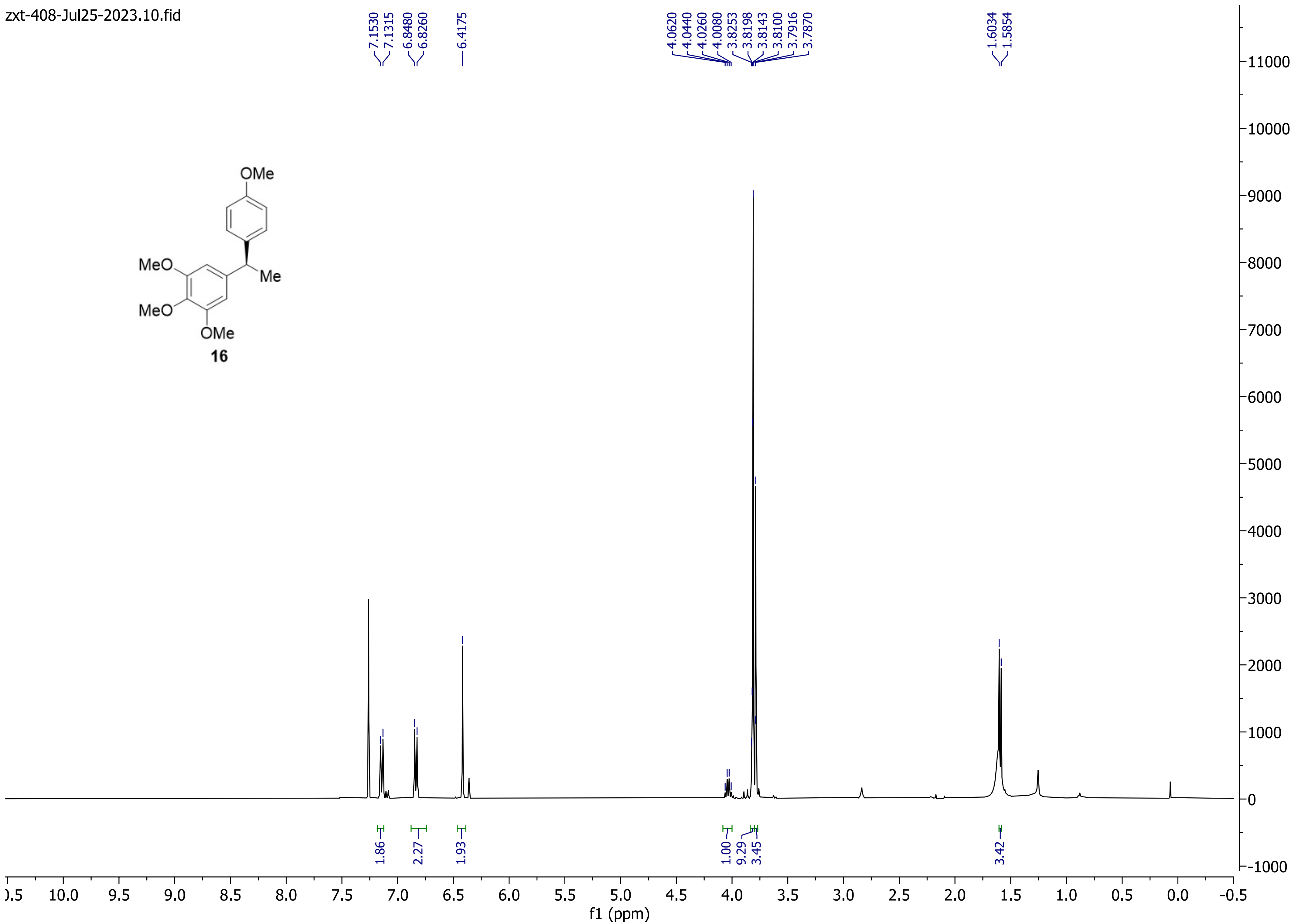
zxt-408-Jul25-2023.10.fid
7.1530
7.1315
6.8480
6.8260
6.4175
4.0620
4.0440
4.0260
4.0080
3.8253
3.8198
3.8143
3.8100
3.7916
3.7870
1.6034
1.5854
OMe
MeO
Me
MeO
OMe
16
1.86
2.27
1.93
1.00
9.29
3.45
3.42
11000
10000
9000
8000
7000
6000
5000
4000
3000
2000
1000
0
-1000
10.0 9.5 9.0 8.5 8.0 7.5 7.0 6.5 6.0 5.5 5.0 4.5 4.0 3.5 3.0 2.5 2.0 1.5 1.0 0.5 0.0 -0.5
f1 (ppm)

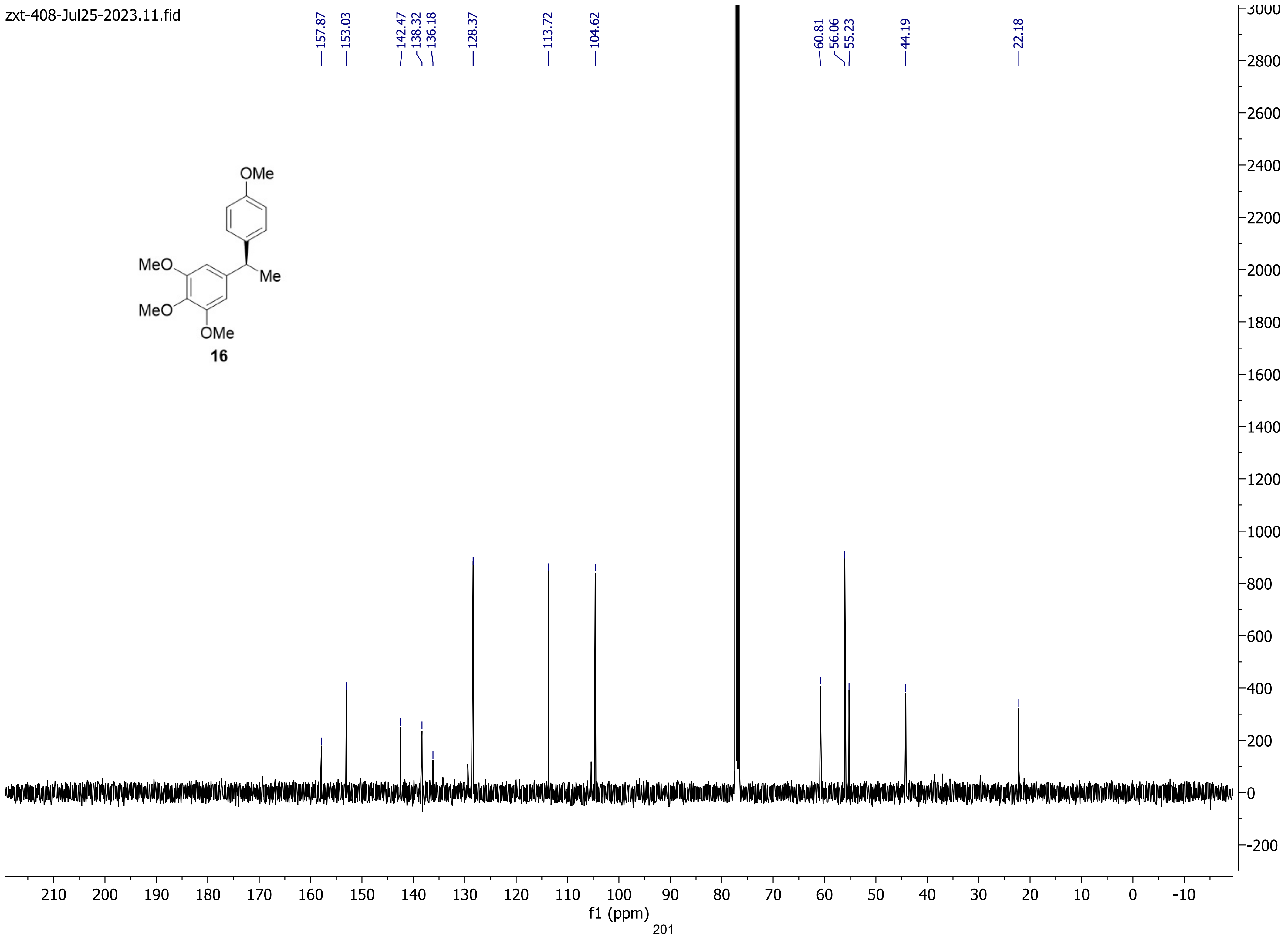
zxt-408-Jul25-2023.11.fid
157.87
153.03
142.47
138.32
136.18
128.37
113.72
104.62
60.81
56.06
55.23
44.19
22.18
OMe
MeO
Me
MeO
OMe
16
f1 (ppm)

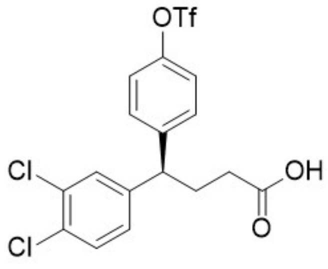

19

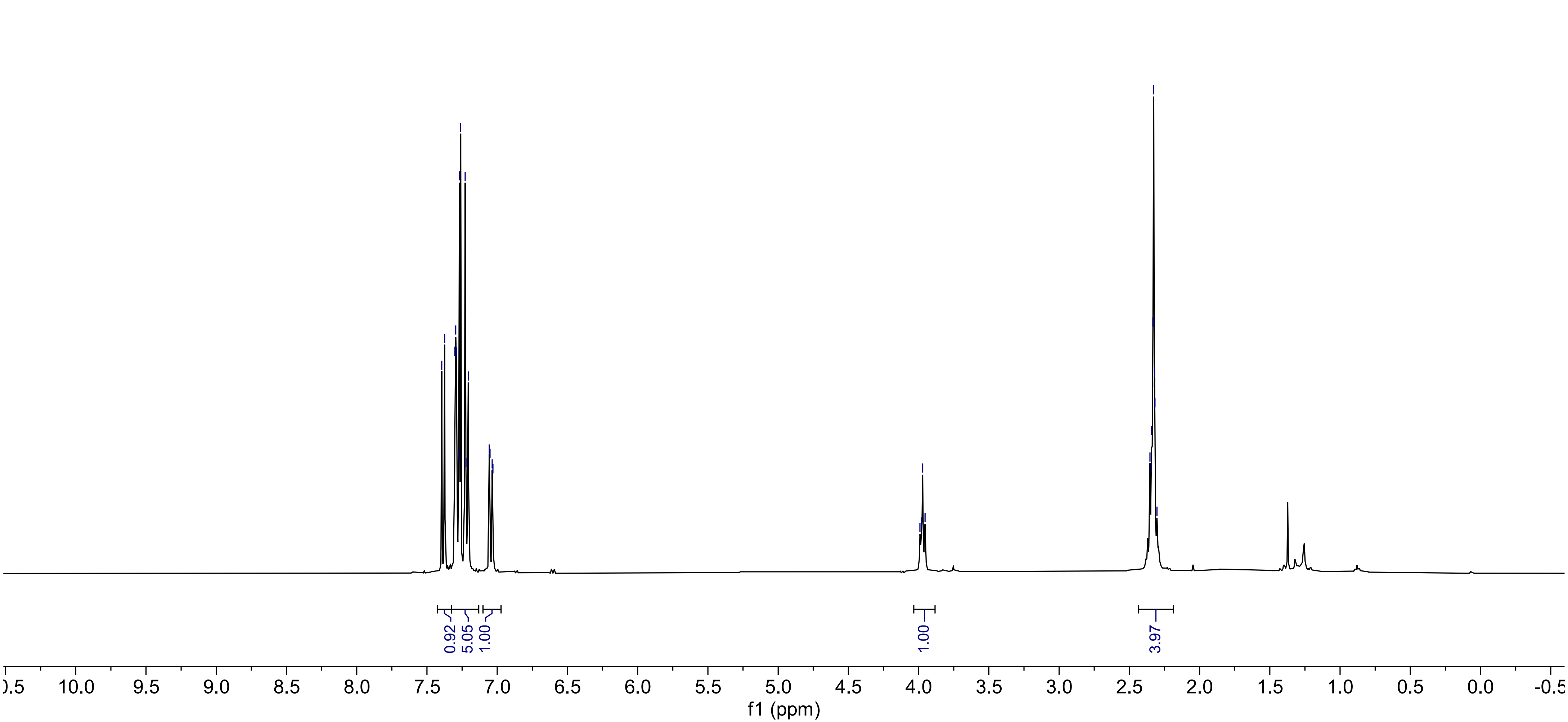

7.3947
7.3739
7.3006
7.2953
7.2907
7.2745
7.2686
7.2600
7.2281
7.2223
7.2060
7.0567
7.0512
7.0358
7.0305
3.9908
3.9796
3.9714
3.9543
2.3531
2.3441
2.3409
2.3323
2.3265
2.3204
2.3177
2.3035
0.92
5.05
1.00
1.00
3.97
f1 (ppm)

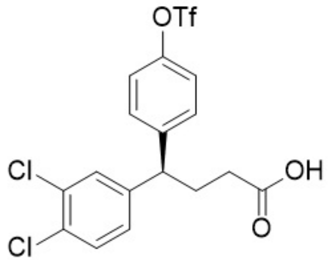

19

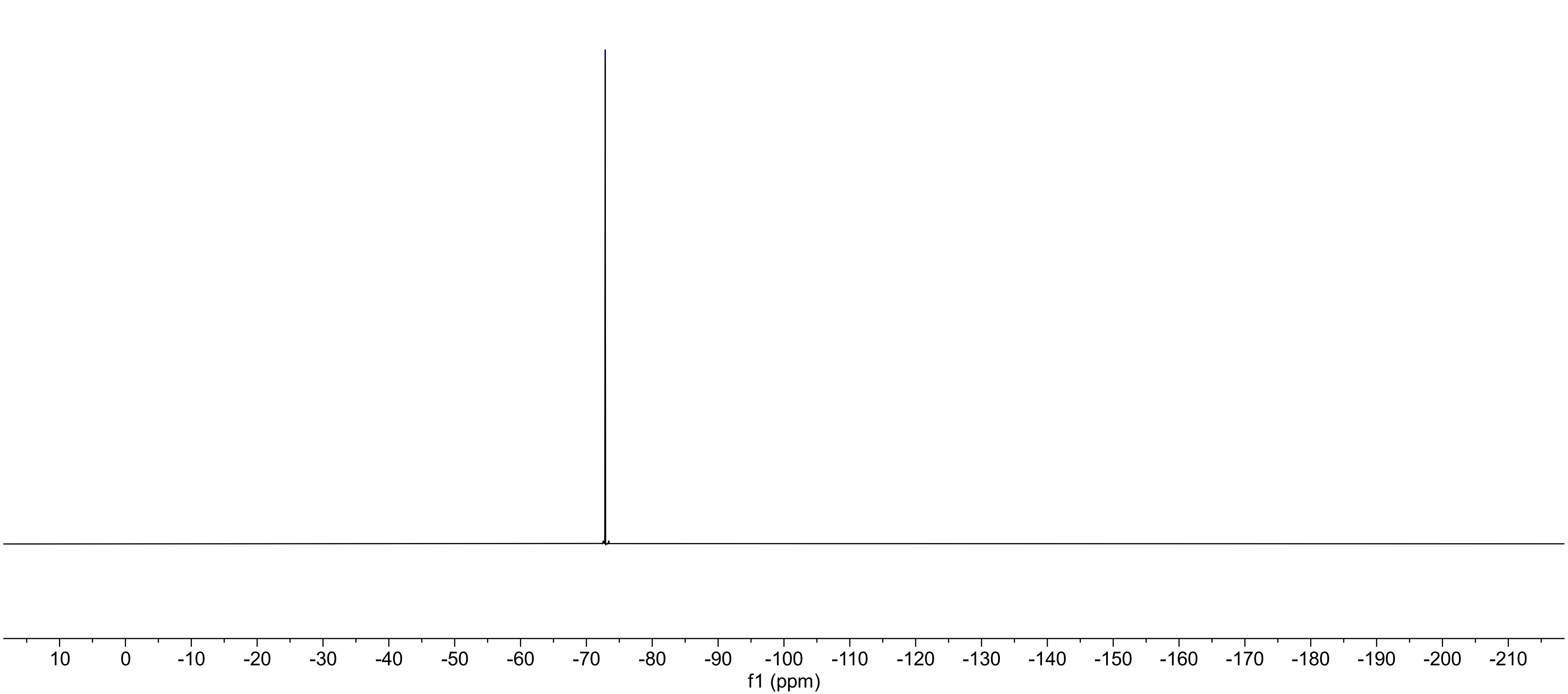
-72.85
10 0 -10 -20 -30 -40 -50 -60 -70 -80 -90 -100 -110 -120 -130 -140 -150 -160 -170 -180 -190 -200 -210
f1 (ppm)

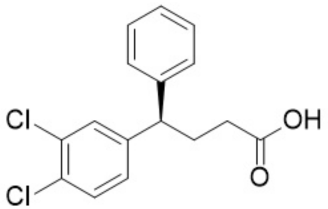
Cl
Cl
OH
O

20

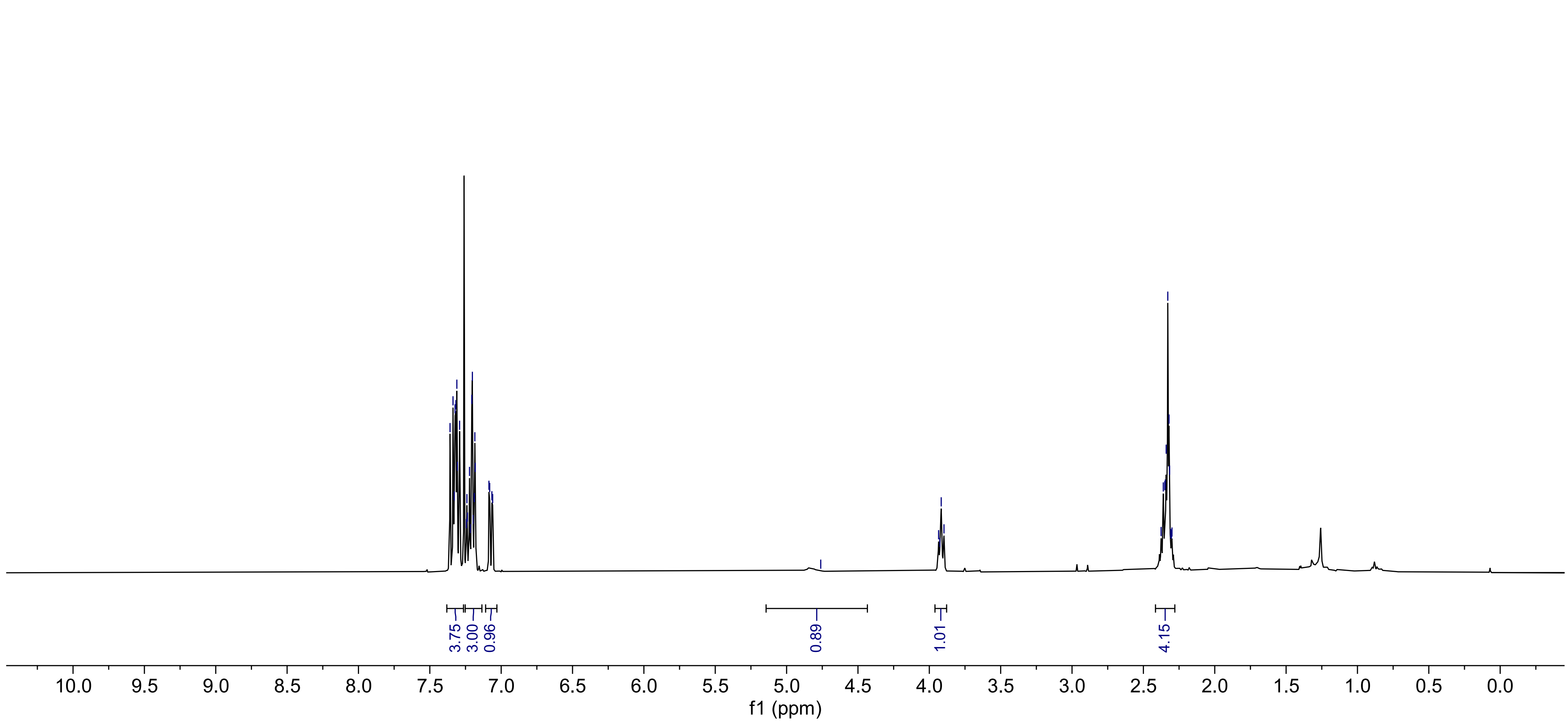
7.3584
7.3375
7.3291
7.3232
7.3176
7.3106
7.3071
7.2913
7.2435
7.2402
7.2367
7.2219
7.2161
7.2058
7.2017
7.1963
7.1887
7.1845
7.1816
7.0858
7.0804
7.0650
7.0597
4.7608
3.9350
3.9264
3.9166
3.8972
2.3759
2.3605
2.3499
2.3467
2.3402
2.3290
2.3204
2.3162
2.3095
2.3040
2.2997
3.75
3.00
0.96
0.89
1.01
4.15
10.0
9.5
9.0
8.5
8.0
7.5
7.0
6.5
6.0
5.5
5.0
4.5
4.0
3.5
3.0
2.5
2.0
1.5
1.0
0.5
0.0
f1 (ppm)

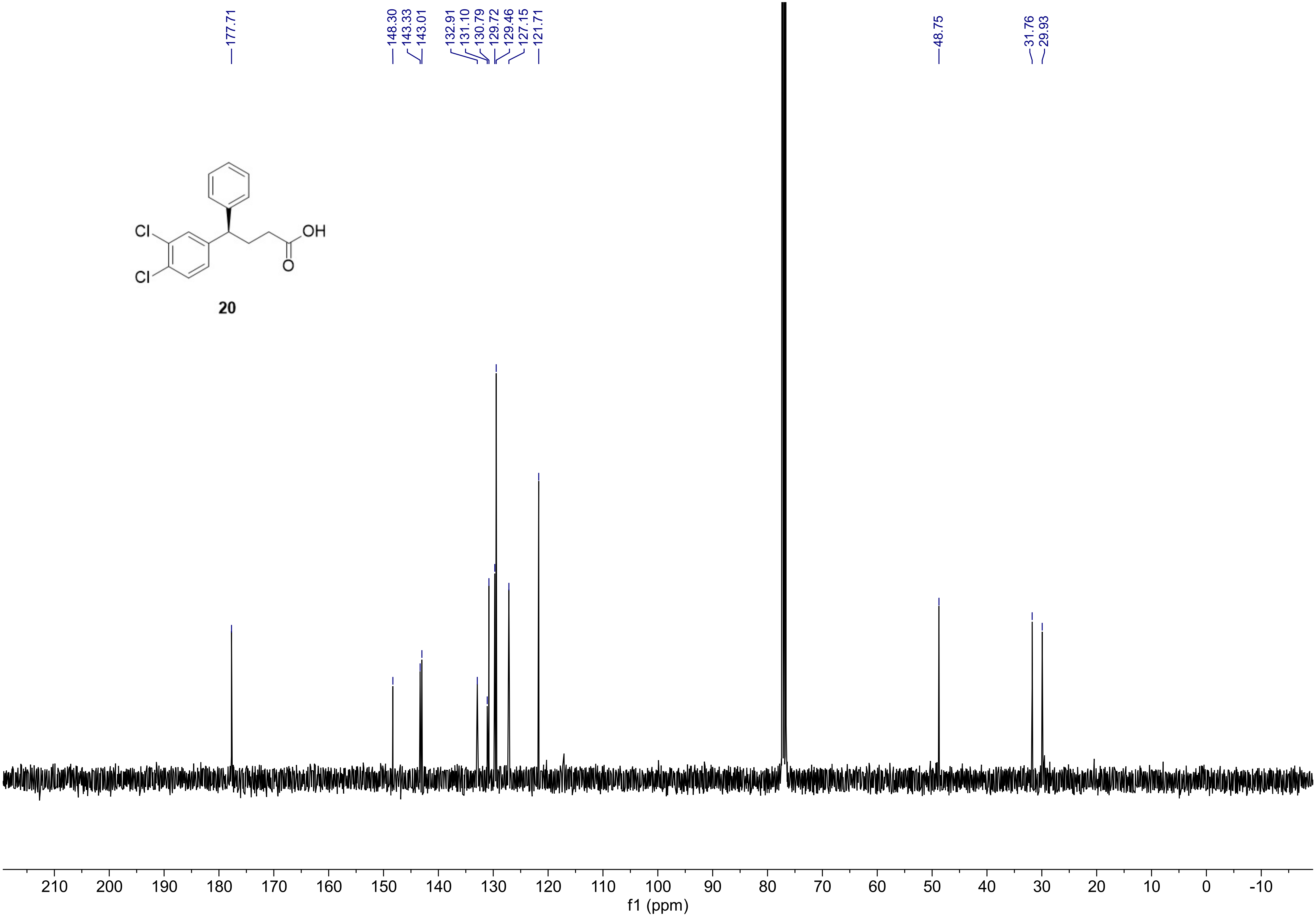
177.71
148.30
143.33
143.01
132.91
131.10
130.79
129.72
129.46
127.15
121.71
48.75
31.76
29.93
Cl
Cl
OH
O
20
210 200 190 180 170 160 150 140 130 120 110 100 90 80 70 60 50 40 30 20 10 0 -10
f1 (ppm)
